# Building a multi-scaled geospatial temporal ecology database from disparate data sources: fostering open science and data reuse

**DOI:** 10.1186/s13742-015-0067-4

**Published:** 2015-07-01

**Authors:** Patricia A. Soranno, Edward G. Bissell, Kendra S. Cheruvelil, Samuel T. Christel, Sarah M. Collins, C. Emi Fergus, Christopher T. Filstrup, Jean-Francois Lapierre, Noah R. Lottig, Samantha K. Oliver, Caren E. Scott, Nicole J. Smith, Scott Stopyak, Shuai Yuan, Mary Tate Bremigan, John A. Downing, Corinna Gries, Emily N. Henry, Nick K. Skaff, Emily H. Stanley, Craig A. Stow, Pang-Ning Tan, Tyler Wagner, Katherine E. Webster

**Affiliations:** 1 Department of Fisheries and Wildlife, Michigan State University, East Lansing, MI 48824 USA; 2 Center for Limnology, University of Wisconsin-Madison, Madison, WI 53706 USA; 3 Department of Ecology, Evolution, and Organismal Biology, Iowa State University, Ames, IA 50011 USA; 4 Center for Limnology Trout Lake Station, University of Wisconsin-Madison, Boulder Junction, WI 54512 USA; 5 Oregon State University, Tillamook County, Tillamook, OR 97141 USA; 6 NOAA Great Lakes Laboratory, Ann Arbor, MI 48108 USA; 7 Department of Computer Science and Engineering, Michigan State University, East Lansing, MI 48824 USA; 8 US Geological Survey, Pennsylvania Cooperative Fish and Wildlife Research Unit, Pennsylvania State University, University Park, PA 16802 USA; 9 School of Natural Sciences, Trinity College Dublin, Dublin, Ireland

**Keywords:** LAGOS, Integrated database, Data harmonization, Database documentation, Data reuse, Data sharing, Ecoinformatics, Macrosystems ecology, Landscape limnology, Water quality

## Abstract

Although there are considerable site-based data for individual or groups of ecosystems, these datasets are widely scattered, have different data formats and conventions, and often have limited accessibility. At the broader scale, national datasets exist for a large number of geospatial features of land, water, and air that are needed to fully understand variation among these ecosystems. However, such datasets originate from different sources and have different spatial and temporal resolutions. By taking an open-science perspective and by combining site-based ecosystem datasets and national geospatial datasets, science gains the ability to ask important research questions related to grand environmental challenges that operate at broad scales. Documentation of such complicated database integration efforts, through peer-reviewed papers, is recommended to foster reproducibility and future use of the integrated database. Here, we describe the major steps, challenges, and considerations in building an integrated database of lake ecosystems, called LAGOS (LAke multi-scaled GeOSpatial and temporal database), that was developed at the sub-continental study extent of 17 US states (1,800,000 km^2^). LAGOS includes two modules: LAGOS_GEO_, with geospatial data on every lake with surface area larger than 4 ha in the study extent (~50,000 lakes), including climate, atmospheric deposition, land use/cover, hydrology, geology, and topography measured across a range of spatial and temporal extents; and LAGOS_LIMNO_, with lake water quality data compiled from ~100 individual datasets for a subset of lakes in the study extent (~10,000 lakes). Procedures for the integration of datasets included: creating a flexible database design; authoring and integrating metadata; documenting data provenance; quantifying spatial measures of geographic data; quality-controlling integrated and derived data; and extensively documenting the database. Our procedures make a large, complex, and integrated database reproducible and extensible, allowing users to ask new research questions with the existing database or through the addition of new data. The largest challenge of this task was the heterogeneity of the data, formats, and metadata. Many steps of data integration need manual input from experts in diverse fields, requiring close collaboration.

**Electronic supplementary material:**

The online version of this article (doi:10.1186/s13742-015-0067-4) contains supplementary material, which is available to authorized users.

## Introduction

Addressing many of the most pressing global environmental problems requires data and knowledge at spatial scales that have been historically understudied (e.g., regional, continental, and global). For example, freshwaters are influenced by eutrophication, climate and land use changes, and the spread of invasive species, all of which have regional to continental controls. The contribution of freshwaters to global carbon cycles is still largely unknown [1–8]. Studying these kinds of ‘macrosystems ecology’ questions (*sensu* [9]) that can inform environmental problems and developing continental or global ecological assessments, requires both data and understanding at broad spatial and temporal scales. In part, our perception generally deepens or changes when variation across both fine and broad scales is taken into account [10]. Many current technological and computing advances are allowing this process to become a reality.

The ‘big data’ era is rapidly transforming the research landscape in the environmental sciences [11–14]. Fast, inexpensive computing has enabled processing of vast amounts of data, which often originates both from modern observational technologies, such as automated sensors, and from national- and global-scaled observatory networks that are generating massive data streams of high spatial and temporal resolution. However, large databases of unprecedented spatial and temporal extent can also be generated by integrating many smaller, site-level environmental datasets, collected *in-situ* across continents to create highly curated integrated data products [12, 15]. Although site-level environmental datasets are labor-intensive and expensive to collect, they are fairly common in many parts of the world and have been collected for many more decades than automated sensors have been in operation. Further, because site-level datasets often focus on relatively few sampled variables, these datasets will be far more useful for answering broad scale research questions when combined with complementary geographic information system (GIS) datasets, available at national scales for features such as land use/cover, climate, topography and atmospheric deposition, to name a few.

To date, much of the discussion of data integration in ecology has focused on the importance and possible use of ‘dark’ data in the ‘long tail’ of science, i.e., the large number of small datasets that make up the majority of science, that are not well indexed or stored and typically are not publicly accessible [16]. Such datasets are essentially invisible to scientists and other potential users and therefore are more likely to remain underused and eventually lost [16]. For environmental data, many such potentially underused datasets are collected by governmental natural resource agencies (e.g., state/provincial, tribal, national), researchers, industry or consulting firms, or citizen science programs. These datasets are often moderately well curated, involve relatively large sample sizes, and have been used primarily for assessment and reporting rather than for research. When attempting to place monetary value on environmental datasets, higher values are often associated with final data products that are properly curated, as compared to poorly curated products, with values exceeding the cost of curation by many times (five to 200 fold [7]). However, the value gained from combining disparate datasets to address broad-scaled research questions can only be fully realized through data harmonization, i.e., adjusting for differences in units, formatting, naming, and other conventions, so that datasets collected by different data providers can be integrated. Although the technology and data exist, there are few existing standards or examples that provide the detailed methods and strategies needed for integrating disparate datasets and data types. In addition to this, environmental science needs a change in perspective. Synthetic and integrated research questions can only be answered in an open-science environment in which both collectors of site-based datasets and creators of integrated databases (each requiring extensive cost and labor) are willing to share their data products and their methods of collection, processing, and integrating, and where they receive proper attribution of their important contributions.

The idea of combining many smaller, site-level environmental datasets into a single database for policy or management purposes has existed for several decades (e.g., for water quality: STORET [17] and NWIS [18]). However, broader use of these datasets is limited as they typically include only a single type of data (e.g., water quality) or lack supporting geographic data. In addition, data integration efforts to answer synthetic research questions have been conducted in the last few decades by empirical ecologists performing secondary or meta-analyses of ecological processes (e.g., [19–23]), and by researchers in working groups at national synthesis centers in the US and other countries producing new knowledge through synthesis [4, 24–27]. These two types of effort have often integrated a moderate number of data types or variables, frequently from published studies. The project that we describe in this paper goes even further to obtain large sample sizes across a broad geographic extent, to integrate heterogeneous types of data (e.g., climate, hydrology, land use, in addition to the site-level data), and to document the full geographic description of all ecosystems within a study area. Creating databases of all ecosystems is important to be able to quantify potential biases inherent in site selection of site-based datasets [28]. Our methods are similar to ongoing work by scientists who are part of networked observatories (e.g., FluxNet, AmeriFlux, NutNet, GLEON) and are responsible for documenting and maintaining large, integrated databases.

For cases in which a relatively manageable number of site-level datasets are integrated, merging can often be done manually and well informed quality control and assurance can be completed using expert knowledge of individual datasets. However, creating large curated data products, such as those commonly used in genomics (e.g., [29, 30]), or through networked observatories, requires methods that are done ‘at scale’, in other words not manually, and that are automated and extensively documented. Further, making such databases extensible, i.e., building the database for future use, requires explicit strategies [23]. A critical step in creating an extensible database is to document all methods associated with integrating disparate datasets, including data provenance, processing, modeling, and formatting. Such documentation ensures that future users of the data can fully understand the construction and limitations of the integrated data product, which is required for effective use and extension.

In this database methods paper, we describe data integration of multi-thematic and disparate datasets. Just as data papers benefit from peer review, so too will database methods papers, facilitating future use and extensibility of the database [30]. Although we describe the methods for our specific database, LAGOS (see below), this paper serves a different purpose from our forthcoming ‘data paper’ that will make LAGOS fully accessible in an online repository and will include data providing co-authors who are recognized and receive credit for their data (e.g., [31]). The purpose of this database methods paper is to document the detailed methods of data integration and database development that our research team of ecologists, ecoinformatics specialists, GIS specialists, and computer scientists used, so that others have an example to build upon.

**Fig. 1:**
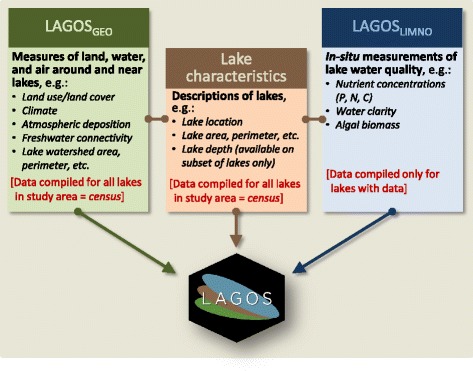
A description of the major components and data themes that are integrated to create LAGOS. P is phosphorus, N is nitrogen, C is carbon. Further detail is provided in Figures 5 and 6

**Fig. 2:**
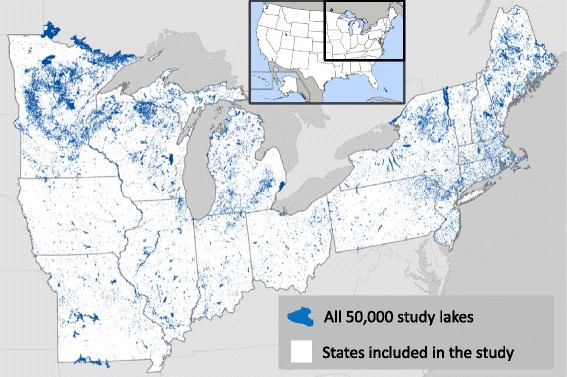
The study extent of LAGOS, showing location of all lakes ≥ 4 ha (blue polygons). The study extent included 17 states in the upper Midwest and Northeastern parts of the US. Note that there are many lakes that straddle the state boundaries but are still included in the database because the source data for the lakes are based on natural watershed boundaries rather that state boundaries

We describe the major steps, challenges, and considerations for building an integrated database of lake ecosystems, called LAGOS (LAke multi-scaled GeOSpatial and temporal database; Fig. 1). LAGOS includes two modules. The first is a geospatial characterization of all lakes within the study extent from ~1980 to 2011, which we refer to as the census lakes (LAGOS_GEO_). The second module is a compilation of water quality data (including lake nutrients, water clarity measures, and pelagic chlorophyll concentrations) from the same time period on a subset of the lakes (LAGOS_LIMNO_). The version of LAGOS described here (version 1.040.0) is at the sub-continental scale of 17 US states spanning 1,800,000 km^2^ (Fig. 2) and includes 40 lake water quality datasets for ~10,000 lakes (with an additional 60 datasets remaining to be imported in the immediate future), and geospatial data from ~21 national geospatial datasets in the public domain.

Although our focus is on lake ecosystems, the steps we outline are broadly applicable to the integration of disparate, multi-thematic, heterogeneous databases in any geospatial scientific discipline. In particular, our approach for integrating broad spatial coverage data with time series data for individual locations will be particularly relevant to a broad range of environmental scientists.

## Review

### Interdisciplinary approach for building integrated databases

**Fig. 3:**
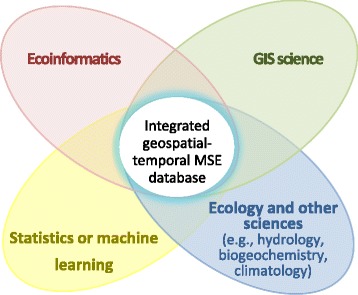
Contributions and collaborations of disciplines for developing an integrated geospatial-temporal database for macrosystems ecology (MSE). Ecoinformatics includes database systems, metadata, and other informatics tools needed for documenting and integrating datasets. Although statistics and machine learning are not used to create the integrated database, the constraints and requirements for future statistical and machine learning modeling should be incorporated into the process from the beginning

The first step when building an integrated geospatial-temporal macrosystems ecology database is to assemble an interdisciplinary research team (Fig. 3). There should be expertise from a combination of disciplines including the main domains related to the research questions (e.g., ecology, hydrology, biogeochemistry, climatology), ecoinformatics, statistics or machine-learning, and geographic information systems (GIS) science. Domain experts formulate the questions that motivate the construction of the database, but often lack the technical expertise required to conduct macrosystems research. Hence, ecoinformatics professionals provide essential specialized knowledge and skills to design and build the database and GIS science professionals provide the skills and tools to create the geospatial component of the database that is so critical for macrosystems ecology research. Statistics and machine-learning professionals play a critical role in the analysis of the finished database, and must also be involved at the early stages to identify database constraints for the anticipated later statistical or machine-learning analysis software, as well as optimal data formats. We found it helpful to have more than one person per discipline, such that no one discipline or disciplinary perspective is either dominant or marginalized [32], and to have team members who serve as ‘disciplinary brokers’; that is, who possess the ability to bridge knowledge or approaches across disciplinary boundaries, thus facilitating the translation of ideas and language across disciplines [33].

We recommend several fundamental principles to help guide the building, maintaining, and sharing of integrated databases for macrosystems ecology research with an open-science perspective (Table 1). First, it is beneficial to create both a census database as well as a ‘sampled’ database to facilitate extrapolation, a common objective of macrosystems research. Second, the database, the metadata of source data, technical documentation of the database integration procedures, and code should be shared for future users in online repositories with permanent identifiers; either immediately, at the end of the project period, or following a suitable embargo period. Third, the provenance of the original data should be preserved to the greatest degree possible, and existing community standards be used to facilitate integration with other efforts. In the case of macrosystems ecology, community standards are still evolving, which makes thorough and clear data documentation at all steps especially important. We also recommend that the database be fully documented via a peer-reviewed data methods paper with a permanent identifier to allow future use and understanding of the database, and to give credit to the database integrators. Similarly, we suggest that a data paper be written with co-authors who are data providers to recognize their data provision. Finally, it is assumed that once the database is shared, there is a set of community policies by which other scientists use and credit the data [34].

**Table 1:** Assumptions and fundamental principles in building, maintaining, and sharing integrated macrosystems ecology databases

• The database should include both a ‘census’ population in which all possible ‘ecosystems’ or ‘sites’ are geographically represented in addition to the sites with *in-situ* data.
• The database will be fully documented, including descriptions of: the original data providers or sources, database design, all data processing steps and code for all data, possible errors or limitations of the data for the integrated dataset and individual datasets, and methods and code for geospatial data processing.
• To the greatest degree possible, existing community data standards are used to facilitate integration with other efforts.
• To the greatest degree possible, the provenance of the original data will be preserved through to the final data product.
• The database will include a versioning system to track different versions of the database for future users and to facilitate reproducibility.
• The database will be made publicly accessible in an online data repository with a permanent identifier using non-proprietary data formats at the end of the project or after a suitable embargo if necessary.
• A data paper will be written with the original data providers as co-authors to ensure recognition of data providers.
• A data-methods paper is written with the data-integration team as co-authors to ensure recognition of data integrators.
• Once the database is made available in a data repository and is open-access, whether it is static (no further data is added to the database) or ongoing (data continues to be added to it), there are a set of community policies by which other scientists use and cite the database, the original data providers, and the database-integrators.

**Fig. 4:**
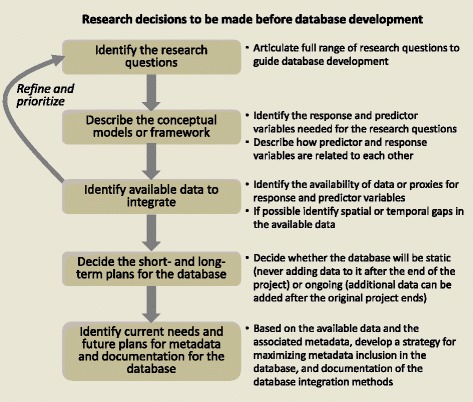
Flow chart of the sequence of research decisions relevant to the database design and integration efforts that are required prior to entering the database development phase

There are five important decisions to be made before developing the database (Fig. 4): (1) identify the overarching and specific research questions; (2) describe the conceptual model to guide the research and identify and prioritize relevant predictor and response variables; (3) identify available data sources and document spatial and temporal gaps; (4) decide the short- and long-term plans for the database as either a static product or an ongoing, extensible, supported product; and (5) based on the short- and long-term plans for the database, develop a strategy for documenting the database integration efforts and for incorporating metadata into the database to make it usable to current and future users. These decisions, and the team discussions leading to them, will strongly influence database design due to the complexity of building integrated spatial-temporal macrosystems ecology databases. In fact, this process is iterative; refinements to the research questions or conceptual models are likely as the database plans or the availability of data change through time. In the next section, we describe the procedures we used to build LAGOS, including the research decisions that guided our efforts.

### Steps in building LAGOS, a multi-scaled geospatial temporal ecology database

**Fig. 5:**
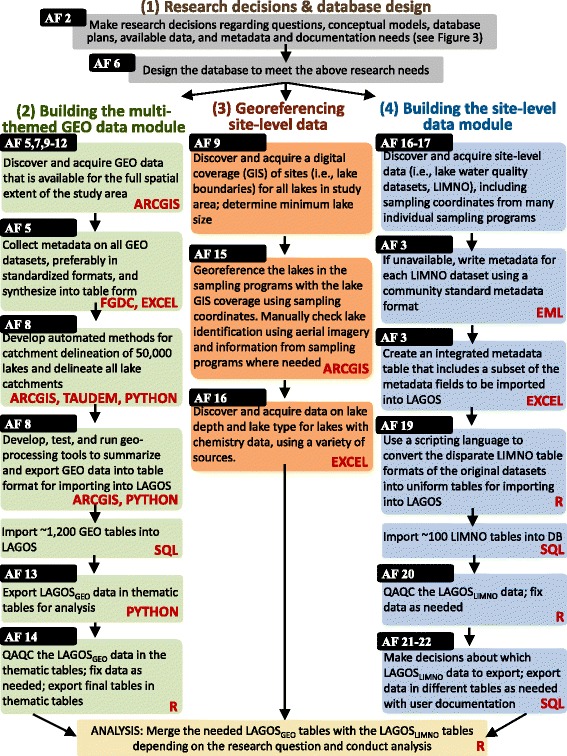
The workflow used to create LAGOS, including the research decisions needed to design the database. Once the research decisions have been made (grey boxes), the workflow is divided into three modules: building the multi-themed GEO data module (green boxes); georeferencing the site-level data (orange boxes); and building the site-level data module (blue boxes). The black boxes with white text identify the Additional files (AF) that describe each element in further detail and the red text provides the programming language or software used for each step. ARCGIS is ArcGIS, Ver 10.1 (ESRI); FGDC is the Federal Geographic Data Committee metadata standard; EXCEL is Microsoft Excel; TAUDEM is the TauDEM Version 5 suite of models to analyze topographical data; PYTHON is the Python programming language; SQL is structured query language used in the PostgreSQL database system; R is the R statistical language [36]; and EML is ecological metadata language

Next we briefly describe the steps to create LAGOS in the text and figures, and include more detailed methods in the additional files, including a glossary of terms that is provided in Additional file 1. Creating a multi-scaled geospatial temporal ecology database required four major efforts described in detail in the following sections (Fig. 5). First, as described above, central research decisions were made to guide database design and development (grey boxes in Fig. 5; and described in detail in Additional file 2. As there were more datasets to integrate into LAGOS than there were funds or time available (a common problem in science), prioritization of data was critical to ensure that our research goals were met. Second, we quantified the diverse geospatial characteristics of the ecosystems under study (green boxes in Fig. 5) at a range of spatial and temporal extents, which involved incorporating information from a range of datasets such as land use/cover, topography, climate, and hydrology. This step required skilled analyses and the development of novel GIS methods specific to our research questions. Because the geospatial data required such different database protocols from our site-level data, these data were put into a separate database module, LAGOS_GEO_. Third, site-level data were georeferenced to enable linkages between the two database modules, a step that was far more complicated and labor-intensive than was anticipated. Fourth, we combined the site-level datasets into one module, LAGOS_LIMNO_.

#### (1) Research decisions and database design

##### Research questions

LAGOS was built to provide answers to our overarching question about cross-scale interactions (CSIs) and their drivers (see [10] for a detailed description of CSIs). Specifically, we asked: (1) At which spatial scales do CSI drivers explain spatial heterogeneity in lake water quality? (2) At which temporal scales do CSI drivers explain temporal dynamics in lake water quality among regions? (3) What are the relative contributions of spatial and temporal drivers to the CSIs that explain spatial and temporal variation in lake water quality? These questions motivated the following decisions in our design of LAGOS. First, LAGOS covers a broad spatial extent (or study area) to enable analysis of lakes along broad gradients of driver variables, such as land use, climate, hydrology, and geology. Second, LAGOS_LIMNO_ covers a broad temporal extent by including as much current and historical data of sampled lakes as possible. Third, to support multi-scaled spatial analysis and to measure and study CSI drivers, LAGOS_GEO_ includes measures of driver variables at spatial extents that range from fine (near an individual lake) to coarse (regions that the lakes are nested within) scales. Finally, LAGOS_LIMNO_ includes a variety of ecosystem-level variables (i.e., measures of water quality in lakes) derived from lake sampling programs. We included all available data from lake sampling programs that varied widely in the timing and frequency of monitoring. LAGOS can then be filtered to select observations at any desired and available timing, frequency, or spatial extent. A critical decision in building LAGOS_LIMNO_ was to import only data that characterized water quality and lake depth rather than other in-lake measures (e.g., acid–base chemistry, temperature, or conductivity). As each lake variable required manual interpretation and harmonizing across datasets, and thus a significant investment of time and financial resources, we prioritized the variables that were necessary to answer our initial research questions.

##### Conceptual framework

We built LAGOS to answer the following fundamental question in macrosystem ecology: what are the CSIs that regulate spatial heterogeneity and temporal dynamics of ecosystems at sub-continental scales? Despite the high probability that CSIs influence lakes, these ecosystems have not been studied in the spatially explicit manner required to quantify CSIs. This is in part because of a lack of a suitable comprehensive multi-scaled spatial framework. The landscape limnology conceptual model [35], which is based on principles of landscape and freshwater ecology, provides a unique lens for understanding how a diverse set of drivers (e.g., land use, climate, hydrology) from different scales interact to create CSIs that affect freshwater ecosystems. Therefore, LAGOS was designed to include measures of landscape, hydrology, atmospheric, and climate driver variables that are thought to control lake ecosystems individually and through interactions with each other within and across scales.

##### Identify available data to integrate

In the US, state (and some tribal) natural resource agencies are mandated by the US Environmental Protection Agency (EPA) to monitor their water bodies for changes in water quality. The EPA requires agencies to document and report the data at regular intervals, resulting in high quality data that have been collected using relatively similar standardized methods. A second data-rich category of lake water quality data we targeted was information from citizen monitoring programs and university researchers (Table 2). Twelve of the ecologists on our team were given the responsibility of identifying and contacting data sources in one to three states, depending on the number of likely sources per state. We first identified and secured state agency data sources. Then the team identified gaps in spatial and temporal coverage and targeted additional citizen and university data sources to fill those gaps. The minimum requirements for including lake datasets in LAGOS_LIMNO_ were: sufficient metadata to describe sampling and sample processing methods (in any metadata form, which was typically a Microsoft Word or.pdf document), information on the water depth at which the water sample was taken, information on the lake location, and data that were not aggregated into means or medians (i.e., only individual sample events).

**Table 2:** The description of the sources of site-level datasets that were identified to integrate into LAGOS_LIMNO_

Program type providing dataset	Number of datasets	Type of sampling	Spatial resolution	Temporal range of data	Temporal resolution
Federal agency	7	Survey	US	1991 - 2007	Single summer sample
	7	Long-term	Single lake - Regional	1984 - 2011	Weekly - Yearly
LTER program	5	Long-term	Single lake - Regional	1967 - 2013	Weekly - Monthly (up to all year)
State agency	14	Survey	State	1937 - 2011	Single summer sample - Monthly
	14	Long-term	Watershed - Regional	1984 - 2011	Weekly - Yearly
Citizen monitoring program	6	Survey	Regional - State	1989 - 2011	Single summer sample - Monthly
	4	Long-term	Regional - State	1974 - 2012	Monthly - Multi-years
Non-profit agency	3	Long-term	Regional	1990 - 2011	Monthly - Multi-years
Tribal agency	5	Long-term	Regional	1998 - 2011	
University research program	16	Long-term	Single lake - Regional	1925 - 2011	Single summer sample - Weekly (some fall and winter samples)

Note that at the time of writing, we have not incorporated all of these datasets into the database. The table describes the types of programs providing data, the type of sampling conducted, the spatial resolution, and the temporal range and resolution of the data

##### Identify short- and long-term plans for the database

Our short-term plan for LAGOS was to answer the above research questions regarding the influence of CSIs on lake water quality, based on the landscape limnology conceptual model. This plan guided which datasets we collected for predictor and response variables. We also had two important long-term plans for the database. First, we intended to make the database available at the end of the project period in an online open access data repository minus any dataset in which the provider has requested the data not be further shared. Second, we wanted the database to be extensible, in other words, we wanted future users to be able to incorporate different geospatial or lake data to the LAGOS infrastructure, in order to conduct new research on lake ecosystems across broad spatial and temporal extents. For example, LAGOS could be used to study how lake water temperature responds to climate change, or how pH responds to changes in atmospheric deposition, and how both vary through space and time. To meet these two goals, we ensured that LAGOS could accommodate the addition of data (such as temperature or pH variables) in the future through a flexible database design, and through careful documentation of the entire data integration process. This latter action was done to ensure proper use and provenance of the underlying data and to provide a road map for adding new data to LAGOS in the future. We will have reached the short-term goals of this research project if we successfully build such a database and answer the set of research questions that were identified *a priori*. We will have reached the long-term goals of our research project if we enable other researchers to build upon and use the database (through both open-access at the end of the project and detailed documentation described here) to answer a diverse range of future research questions.

##### Identify the metadata and documentation needs for the database and establish a metadata plan

We took a multi-pronged approach to metadata for LAGOS because no single approach would meet all of our needs. The metadata for LAGOS_LIMNO_ were created as follows, which are described in more detail in Additional file 3. First, we created a control vocabulary to provide a standardized way to describe the data, variable names, and units. Our control vocabulary for LAGOS_LIMNO_ is provided in Additional file 4. Second, we documented the individual site-level metadata for each water quality dataset using ecological metadata language (EML), which is the community standard for ecological datasets. We wrote the documentation in this standard format ourselves because few datasets had existing standard metadata files. Third, to facilitate reuse of the data, we added important components of metadata, related to the data source and laboratory methods, directly into LAGOS_LIMNO_ at both the level of the dataset ‘source’ and the data ‘value’ (Fig. 5). Fourth, for all data manipulations conducted prior to loading into LAGOS, we used scripting languages for documentation (see below). For the LAGOS_GEO_ module, we compiled existing metadata that was mostly in FGDC (Federal Geographic Data Committee) format, which is the standard for GIS datasets. Parts of the metadata were compiled into tables in order to document, among other things, the program that produced the data layer, the data type, the source metadata file URL, and the temporal and spatial resolution of the data, all of which is provided in table form in Additional file 5. For both modules, we carefully recorded all methods for data integration as described in this paper and the Additional files. In addition, we created a user documentation file for each data export version that describes changes to the database or data.

##### Database design

The key principles underlying the design of traditional relational databases are based on the theory of database normalization, which dictates how the schemas in a database should be organized to minimize duplicate information across multiple tables, to reduce wasted storage of null values, and to ensure that the dependencies among data items are correctly manifested in the database. These databases also provide means for increased quality control by employing strong data typing (e.g., dates go in date fields, numbers in number fields), and by including lookup tables that eliminate spelling errors and constrain users to controlled vocabularies. However, applying these principles alone for the design of LAGOS was insufficient. We needed a design that would resolve a range of data integration challenges while remaining flexible enough to accommodate future database extensibility, requiring increased complexity in the design and implementation of LAGOS. A detailed description of the database design is provided in Additional file 6.

##### LAGOS is a combination of two modules

**Fig. 6:**
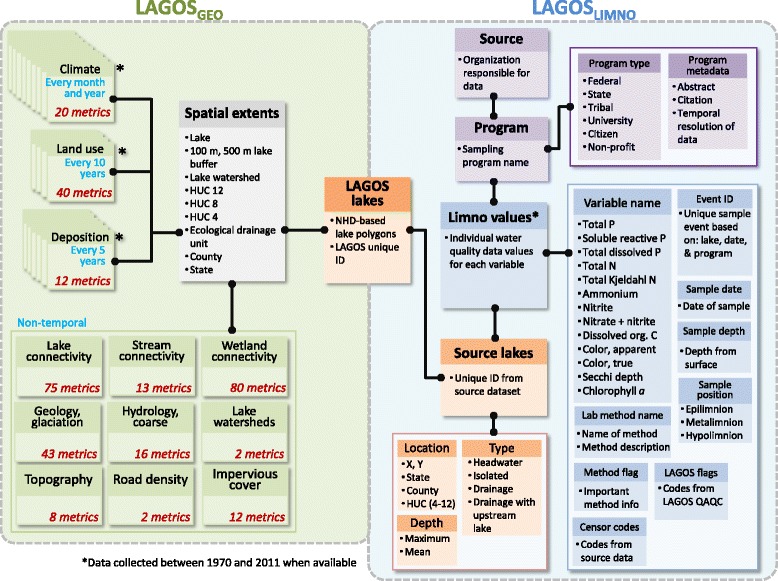
Database schema for LAGOS including the two main modules: LAGOS_GEO_ (green box) and LAGOS_LIMNO_ (blue box). The component that links the two models is the ‘aggregated lakes’ table (LAGOS lakes) that has the unique identifier and spatial location for all 50,000 lakes. LAGOS_GEO_ data are stored in horizontal tables that are all linked back to the spatial extents for which they are calculated and ultimately linked to each of the 50,000 individual lakes. The LAGOS_GEO_ data includes information for each lake, calculated at a range of different spatial extents that the lake is located within (such as its watershed, its HUC 12, or its state). Each green box identifies a theme of data, the number of metrics that are calculated for that theme, and the number of years over which the data are sampled. LAGOS_LIMNO_ data are stored in vertical tables that are also all linked back to the aggregated lakes table. The ‘limno values’ table and associated tables (in blue) include the values from the ecosystem-level datasets for water quality; each value also has other tables linked to it that describe features of that data value such as the water depth at which it was taken, the flags associated with it, and other metadata at the data value level. The ‘program-level’ tables (in purple) include information about the program responsible for collecting the data. Finally, the ‘source lakes’ table and associated tables include information about each lake where available. Note that a single source can have multiple programs that represent different datasets provided to LAGOS

LAGOS_LIMNO_ and LAGOS_GEO_ (Fig. 6). LAGOS_LIMNO_ required integration of nearly 100 limnological datasets from disparate sources. To ensure that the LAGOS_LIMNO_ database module would be extensible, a vertically oriented (i.e., long) database design was developed (Fig. 6). We provide a detailed description of our database design in Additional file 6. This design enables new variables to be appended to the database as new datasets are loaded, without altering the underlying database schema. For the database design, we chose to extend the CUAHSI (Consortium of Universities for the Advancement of Hydrologic Science) Community Observations Data Model [36] that implements these characteristics and is well accepted by a large user community for storing hydrologic measurements.

 The LAGOS_GEO_ module includes a wide range of data derived from publicly available information from multiple sources, including variables on climate, land use and land cover, atmospheric deposition, hydrology, and freshwater connectivity. LAGOS_GEO_ primarily consists of data values calculated at a series of spatial extents such as lake, county, state, watershed, or region that are described in detail in Additional file 7. LAGOS_GEO_ is almost exclusively horizontal in orientation because there are no metadata columns related to the data value columns. Thus, we gain no flexibility or thoroughness of documentation of the underlying data values by storing them vertically (unlike with LAGOS_LIMNO_). Despite the horizontal orientation of this module, it is still fairly extensible through the addition of new tables.

#### (2) Building the multi-themed geospatial/temporal data module: LAGOS_GEO_

We built LAGOS_GEO_ using a number of geospatial datasets that are available online from US federal agencies and other research groups. Most of the available data had to be processed before being integrated in LAGOS_GEO_. Hence we created a GIS toolbox, the LAGOS-GIS toolbox, containing multiple tools to calculate a series of metrics from these layers, in order to define, classify, and characterize the population of surface water environments found in the study extent, based on their hydrologic and landscape context. Additional file 8 provides the full documentation for the LAGOS-GIS toolbox that is provided online in a repository.

The entire population of lakes (>50,000) across the study extent (i.e., the census data) is simply too large and complex to characterize manually. Instead, the LAGOS-GIS Toolbox allows a semi-automated geoprocessing workflow leading to: 1) watershed delineations for each lake, 2) robust addition of attributes to lakes and the zones (or spatial extents) in which they reside, 3) determination of ‘connectivity’ metrics for census lakes, and 4) tools that summarize continuous data in a consistent way for a variety of spatial extents. This toolbox was crucial for building LAGOS_GEO_ and provides a mechanism for easily repeating analyses as new data become available, or when these variables need to be calculated for other regions or with different sources of data. Additional file 5 describes the metrics of climate, atmosphere, geology, topography, and land use and land cover features that have been generated for LAGOS_GEO_ using the toolbox. In addition, Additional files 9, 10, 11 and 12 describe the underlying data and the connectivity metrics that we calculated in order to define and classify lakes, streams, and wetlands based on their position in the hydrologic flowpath and according to their connection(s) with other surface water features.

The above metrics have been calculated in several different ways to carve up the landscape (i.e., spatial extents): (1) political boundaries, (2) hydrological units [37], (3) lake watersheds based on topography, and (4) buffers consisting of boundaries a specified distance from the lake shoreline. These metrics allow the users to choose those that best match the scientific questions addressed (e.g., understanding how nearby land use affects lake nutrient concentrations would take advantage of land use/cover calculated for the 100 m lake buffer). Calculating all of these different geographical metrics, however, results in nearly unmanageable numbers of columns (e.g., calculating average catchment slope ten different ways results in ten different variables and hence ten columns in the database). To circumvent this problem, we generated ‘ZoneIDs’ that are directly linked to each spatial extent in LAGOS_GEO_ and can be associated with any lake in LAGOS_LIMNO_. We then exported, separately, smaller tables that included a number of variables sharing a main theme and common data sources (e.g., land use/ cover) for each spatial extent. Based on analytical needs, one can then reassemble the relevant elements using the ZoneIDs and work with a more manageable database. Additional file 13 describes the strategy for exporting the data for use for statistical modeling.

The last step in building LAGOS_GEO_ was the quality assurance/quality control (QAQC) procedures. Our QAQC procedures for LAGOS_GEO_, which are fully described in Additional file 14, was not able to rule out errors in the base layers themselves. Nor was our verification intended to identify statistical outliers. Rather, we flagged errors and egregious values that 1) do not make ecological sense, 2) are well beyond what has been detected in previous studies, 3) are not technically feasible (e.g., lake mean depth > maximum depth), or 4) are indicated as ‘not available’ when data exist. Once these basic verifications were performed, the data were made available for use by researchers with the recognition that QAQC is an ongoing process that benefits from continuous feedback from the database users, and that different uses of the database may require further QAQC procedures.

#### (3) Georeferencing site-level data

A census lake in LAGOS is a perennial body of relatively still water ≥ 4 ha in surface area, including natural lakes and reservoirs, but excluding entirely artificial water bodies such as sewage treatment or aquaculture ponds (identified as such by our lake data source, the National Hydrography Dataset (NHD). A threshold of 4 ha for lakes was the best trade-off between having as many lakes as possible included in the census dataset balanced against minimizing errors for extrapolation purposes as we describe in Additional file 9.

We describe how we georeferenced the lake sampling location from monitoring and research programs to a lake polygon in the NHD in Additional file 15. This step was challenging because of differences in unique lake identifiers among programs (data sources), and inconsistencies and sometimes errors in the locational information provided for lakes. We concluded that using a lake’s latitude/longitude (which was almost always provided by the water quality data providers) was the best way to link a lake’s sampling data to its location in the NHD dataset in an automated way. However, this approach was ‘semi-automated,’ requiring manual checking and additional manipulations because the provided coordinates sometimes fell outside the NHD lake polygon (e.g., the coordinates indicated the shoreline or the lake access point).

#### (4) Building the site-level data module: LAGOS_LIMNO_

A multi-step process was developed to create LAGOS_LIMNO_, the site-level data module containing water quality information; steps included identifying and contacting data providers, acquiring the data, creating metadata, manipulating and importing data into LAGOS_LIMNO_, developing QAQC procedures, and exporting the data for statistical modeling and analysis. The strategy that we used for identifying potential data providers is described in Additional file 16. We prioritized datasets that were already in the public domain, such as those from state agencies and citizen monitoring programs, because these datasets often had the most data, and facilitated future data sharing. Additional file 17 describes all of the datasets that we identified and obtained data from. When we contacted data providers, we described the general goals of the research project and the data needs, in order for the potential data provider to assess their willingness and ability to contribute to LAGOS_LIMNO_ as we describe in Additional file 18.

Although lakes included in this module do not necessarily have simultaneous measurements of all variables, all lakes have at least one measurement of one of the 17 variables. In addition, lake depth, a variable very important for interpretation of water quality data, is also included in LAGOS_LIMNO_. However, it was not always available in the water quality databases that we obtained. Therefore, we conducted web searches to identify additional sources of lake depth data from lake associations, fishing maps and resources, and other state databases. LAGOS_LIMNO_ contains 17 water quality variables.

The structural and semantic heterogeneity of the data sources (including their diverse file formats, schemas, naming conventions, sampling approaches, measurement units, and detection limits) presented significant challenges to the data integration task. In many cases, a single source provided us with multiple data tables with different information that were not easily related to each other, or that contained a considerable amount of unrelated information. In some cases, no locational information was provided and the lake locations had to be determined manually based on lake names or other auxiliary information. The lack of a controlled vocabulary, common schema, and metadata standards presented enormous challenges in developing automated techniques for processing and importing data into LAGOS_LIMNO_. Instead, we used a semi-automated approach, which was labor-intensive and required customized scripts to be written for processing and loading each data source separately.

Individual datasets were processed using scripts developed in the R statistical [37], SQL, and Python languages to transpose the data from the schema in which the data were provided to the schema employed by LAGOS_LIMNO_ which is described in detail in Additional file 19. Individual scripts were retained to ensure data provenance documentation and reproducibility of procedures. Although we have written scripts for all of the ~100 datasets that we have received, as of the writing of this paper, we have imported about half of those datasets due to the labor-intensive nature of dataset harmonization.

After sufficient datasets were imported to create an integrated LAGOS_LIMNO_ database, the water quality data were exported for detailed QAQC analysis of the integrated database, which we describe in detail in Additional file 20. The goals and procedures for QAQC of LAGOS_LIMNO_ were different than for LAGOS_GEO_ due to the different data types, processing, and potential errors. The overarching purpose of the QAQC analysis for LAGOS_LIMNO_ was to identify potential problems in the data import process such as incorrect unit conversion and to locate egregious values that were either not feasible (e.g., dissolved fraction of a specific nutrient having a greater concentration than total dissolved + particulate form) or had a high likelihood of exceeding the maximum possible value in a lake. For example, of the 1,227,922 observations of all water quality variables in LAGOS_LIMNO_ Ver 1.040.0, only 21 values were deleted due to exceeding the ‘egregious value’ threshold. These thresholds were set at extremely high levels to ensure that no extreme but real values would be unnecessarily dropped. After that step, there were several other procedures to identify values that were questionable that were then flagged in the database with a LAGOS flag. In order to remove observer bias and ensure repeatability of the QAQC procedures, we generated scripts in R that automatically identified and flagged egregious and questionable values based on the set of criteria explained. In total, approximately 0.5 % of the data values were flagged as egregious or questionable (i.e., 6,498 out of 1,227,922 observations).

The final step in building the LAGOS_LIMNO_ data module involved creating scripts to export the data into a readily accessible format for statistical analysis and ecological synthesis as described in Additional file 21. This process involved transposing a multi-table, vertical-structure database into horizontal flat files that were optimized for most statistical applications. Finally, with each export, a corresponding user documentation file, which we provide in Additional file 22, was generated, highlighting any important changes that occurred with the corresponding export, the data tables exported, the fields associated with those tables, and a description of the contents of each field exported. As described, we have implemented a versioning system that allows users to use the database before all datasets have been loaded and actually recognizes the advantage to be able to always add data to the database into the future. For each LAGOS_LIMNO_ version, we implement all steps described in this section to create a functional database that can be used for research.

### Lessons learned from building an integrated database

Harmonizing measurements from many heterogeneous datasets is a challenging task, regardless of environmental discipline or ecosystem type. Throughout the process of harmonizing ecological measurements from diverse lake datasets, we were confronted with unanticipated challenges. For example, we found many different sampling schemes and methods for recording sampling events. Sampling approaches appeared to have been driven by a combination of specific hypotheses and research goals; convenience and logistical feasibility; and historic precedent, all of which became incorporated into formal protocols. Even when lake sampling was intended for long-term monitoring, analytical methods were not always coordinated among different lakes, lake districts, counties, or states. We also found that detection limits of analytical methods were lacking for many lake datasets, or that detection limits changed through time or were different across methods that were employed through time. Many of the challenges we encountered required manual integration, interpretation, or fixing, which is labor-intensive and thus expensive.

We developed a set of best practices for data integration to overcome these (and other) obstacles, resulting in a highly functional, integrated, and well documented data product that can be maintained and extended into the future and used to answer questions that have not yet been conceived. In particular, we suggest consideration of three important design features of integrated databases: 1) a flexible database design that does not cater to a particular type of data analysis or programming language; 2) a controlled vocabulary with explicit definition of terms and mappings of disparate terminology across datasets; and 3) strategies to preserve data provenance and detailed data provenance documentation. Below, we elaborate on the three design features critical to producing an integrated database.

#### 1. The data model

Although most statistical analyses require a horizontal data array, the more flexible data model for storage and manipulation is the long, or vertical, data matrix format. The vertical format can easily accommodate variables that link to other tables, describing additional data such as sampling location and methods, data originator, data provenance, and other metadata that may be needed for specific analyses.

#### 2. Controlled vocabulary

An important part of data harmonization is the agreement on a standardized vocabulary for variables. This process not only involves basic agreement on the variable definition, but it also requires extensive domain knowledge for interpreting terminology used by each data provider, particularly if information that would help with interpretation is missing. A mapping between variables used by the data source and the controlled vocabulary of the integrated database may involve the need to apply major transformations of the data. Once these decisions are made, they need to be implemented consistently across datasets.

#### 3. Preserving and documenting data provenance

Preserving data provenance ensures that a majority of the original information in a given dataset is retained during the data integration process. Similarly, data provenance documentation refers to a record of all changes made to a dataset during the integration process (e.g., R script, text file, extensible markup language (XML) file). Ensuring and documenting data provenance are crucial for creating a valuable integrated database for a variety of reasons. First, the original data provider needs to be acknowledged and linked to the original and unaltered raw data and metadata. Ideally, the original datasets are archived and published in a formal repository and the citation is used in the provenance documentation of the integrated data product. However, because few data providers have published raw data, the link to the originator information needs to be maintained in the data product. Next, it is important to document all data conversions and QAQC measures that were applied to the original data, as well as to maintain as much information from the source dataset as possible. Finally, the data product should be meticulously documented, formally archived in a data repository, and preferably published in the form of a data paper (including all scripts and related data provenance documentation).

The success of these three best practices was essential to the formation of LAGOS and relied upon the close collaboration between domain and informatics experts on the team. For example, it was not enough to assign data manipulation tasks to informatics staff without frequent and deep interactions with domain experts. These best practices, implemented in a highly collaborative environment, are themselves labor-intensive and fairly expensive. However, the investment is easily justified when one takes the long view: many future research questions can be answered with such databases, resulting in a wide range of high-impact research outcomes (e.g., future publications, education applications, public outreach materials, and decision-making applications). When these future database uses are factored in, the cost of curation becomes quite low indeed.

## Conclusions

Large, synthetic, reproducible databases, compiled from disparate, minimally accessible, datasets and well integrated with heterogeneous data sources, are required to address some of the most important large-scale environmental problems facing society. In the current big data and open science research era, these integrated databases require thorough harmonization and documentation to be useable by other researchers and policy-makers and extended into the future. Despite computational and technological advances and an increasing emphasis on interdisciplinary research, several challenges remain to creating such databases for synthetic ecological research. Although traditional training in ecology has emphasized quantitative analysis, such training has not adequately equipped most ecologists with the ‘data-intensive science’ skills needed to design, construct, document, and manipulate the databases that are now available or buildable. Based on our experience building LAGOS, two of the largest challenges are the extreme heterogeneity of data sources and the lack of standards for ecological data, both of which create problems for automation of data harmonization and integration. A major conclusion of our effort is that even at the larger temporal and spatial scales associated with macrosystems ecology research, numerous data integration steps require manual processing from domain experts in conjunction with site experts or data providers, and close interactions between domain and informatics experts. Although there are difficult challenges associated with building these integrated datasets, these same challenges provide substantial opportunities, especially for early-career ecologists, for interdisciplinary training in ecoinformatics and database management, and classical ecology; thus pushing the ecological boundary to answer important macrosystems ecology questions.

## Additional files

Additional file 1: **Glossary of some of the terms used in creating LAGOS.** This file contains definitions of many of the commonly used terms that were used to create LAGOS to aid future users of this database. Additional file 2: **Research decisions that guided the creation of LAGOS.** A description of the major decisions and rationale in the creation of LAGOS that were related to the research questions that LAGOS was designed to answer, the conceptual framework for the project, and the longterm plans for the database. Additional file 3: **Creating integrated metadata for LAGOS**_**LIMNO.**_ The approaches that were used to document multiple forms of metadata for LAGOS_LIMNO_. Additional file 4: **Controlled vocabulary for LAGOS**_**LIMNO.**_ A list of standardized vocabulary used to translate each of the disparate individual datasets into a common vocabulary for the data itself (i.e., the variable names) as well as for the metadata. Additional file 5: **Compilation of metadata and description of metrics that we calculated for LAGOS**_**GEO.**_ This file contains three tables, the first contains the metadata for the GIS baselayers, the second contains a description of the metrics that we created for LAGOS_GEO_, and the third contains the lookup table for the classes of surficial geology that we created for all LAGOS metrics. Additional file 6: **LAGOS database design.** A description of the LAGOS database design, the origin and justification of the design, and the extensibility of the database. Additional file 7: **The spatial extents for LAGOS**_**GEO.**_ The spatial extents for which we calculated all GEO features that were delineated by either political, topographical, or hydrologic boundaries. Additional file 8: **LAGOS GIS Toolbox documentation.** This file is the documentation for the LAGOS GIS Toolbox that is currently available in an online repository. Additional file 9: **Data sources, definition, and classification of lakes.** Detailed description of the sources, definitions and classifications that were used to describe lakes in LAGOS. We also include a description and justification for the minimum lake size used in LAGOS based on an error analysis of the data source. Additional file 10: **Data sources, definition, and classification of rivers and streams.** Detailed description of the sources, definitions, and classifications that were used to describe rivers and streams in LAGOS. Additional file 11: **Data sources, definition, and classification of wetlands.** Detailed description of the sources, definitions, and classifications that were used to describe wetlands in LAGOS. Additional file 12: **Freshwater connectivity and composition metrics.** Detailed description and justification of the freshwater connectivity and composition metrics that we developed for LAGOS. The metrics were designed to quantify the spatial heterogeneity in lake, stream, and wetland abundance and surface hydrologic connectivity at a range of spatial extents. Additional file 13: **Database export formats for LAGOS**_**GEO.**_ This file contains a description of the strategies for exporting the heterogeneous and large volume of data from LAGOS_GEO_. Additional file 14: **QAQC protocol for LAGOS**_**GEO.**_ The protocols that we used to QAQC the data after all of it had been loaded into and then exported from LAGOS. Additional file 15: **Georeferencing the lake sampling locations to lake polygons using GIS.** A description of the challenges and solutions to georeference each lake in our study area and connect that location to lake sample events. Additional file 16: **Strategy for discovering and acquiring lake water quality datasets.** The approach that we used for discovering, requesting and acquiring lake water quality datasets from a variety of data providers, focusing on datasets already in the public domain. Additional file 17: **LAGOS**_**LIMNO**_ **data sources and providers.** A detailed description of the programs and sources of limnological water quality data used in LAGOS_LIMNO_ Additional file 18: **Example memo sent to a potential data provider requesting a water quality dataset.** This file contains the memo that we sent to potential data providers requesting data that described the purpose of the study as well as the short- and long-term plans for the data. Additional file 19: **Procedure for converting individual water quality datasets into the LAGOS**_**LIMNO**_ **schema.** The steps that used to convert the heterogeneous water quality datasets into a common format to eventually import into LAGOS. We include an example script and log file created for each dataset prior to loading into LAGOS_LIMNO_. Additional file 20: **QAQC protocol for LAGOS**_**LIMNO.**_ The protocols that we used to QAQC the data after all of it had been loaded into and then exported from LAGOS_LIMNO_. Additional file 21: **Database export formats for LAGOS**_**LIMNO.**_ This file contains a description of the strategies for exporting the large volume of data from LAGOS_LIMNO_, and the challenges in dealing with sample depth or position in the lake. Additional file 22: **Example user documentation for LAGOS**_**LIMNO.**_ This file contains the user documentation that is provided to project participants for each new version of LAGOS_LIMNO_.

## Abbreviations

ARCGIS: ArcGIS software version 10.1
CSIs: Cross-scale interactions
CUAHSI: Consortium of Universities for the Advancement of Hydrologic Science
EML: Ecological metadata language
EPA: Environmental Protection Agency
EXCEL: Microsoft Excel
FGDC: Federal Geographic Data Committee
GEO: Individual geospatial dataset used to populate LAGOS_GEO_
GIS: Geographic information system
HUC: Hydrologic unit code IQR, interquartile range
LAGOS: Lake multi-scaled geospatial and temporal database
LAGOS_GEO_: Multi-themed geospatial data in LAGOS
LAGOS_LIMNO_: Site-level limnological data in LAGOS
LIMNO: Individual limnological datasets used to populate LAGOS_LIMNO_
LTER: Long-Term Ecological Research program
MSE: Macrosystems ecology
NHD: National Hydrography Dataset
Python: Python programming language
QAQC: Quality assurance/quality control
R: R statistical language
SQL: Structured query language used in the PostgreSQL database system
TAUDEM: TauDEM version 5
XML: Extensible markup language
