# Supplementary material for: Building a multi-scaled geospatial temporal ecology database from disparate data sources: fostering open science and data reuse
Source: Gigascience. 2015 Jul 1;4:28. doi: 10.1186/s13742-015-0067-4 (PMC4488039; doi:10.1186/s13742-015-0067-4)
Supplement: Additional file 1: — Glossary of some of the terms used in creating LAGOS. This file contains definitions of many of the commonly used terms that were used to create LAGOS to aid future users of this database. [file 13742_2015_67_MOESM1_ESM.docx]

Additional file 1

**Glossary of some of the terms used in creating LAGOS**

Biplot Plot showing the relationship between two variables used for detection of outliers during QAQC.

Censorcode (LAGOS flag) Values that are created by the data-import step of LAGOS based on the flags from the original source data (one of three options: 'less than', 'greater than', or 'not censored'). These are defined as data values where the value of the measurement is only partially known, i.e., a measured concentration above or below a published detection limit or a Secchi measurement that hit the bottom of a lake. 'Not censored' indicates that either the data are within the analytical range of the method, or no information about detection limits was provided.

CHAG The category of geospatial features that are exported together in a single table that includes: climate, hydrology, atmospheric chemistry, and geology.

Chla Chlorophyll *a*

Citizen monitoring program Citizen or volunteer program (e.g., New York Citizens Statewide Lake Assessment Program) that samples lakes using citizens or volunteers, but is often run by a state agency or a not-for-profit type organization.

CLIM_ANN The category of geospatial features that are exported together in a single table of annual climate data for 42 years; includes temperature (mean, minimum, and maximum) and total precipitation.

CLIM_MON The category of geospatial features that are exported together in a single table of monthly climate data for 42 years; includes temperature (mean, minimum, and maximum), total precipitation, ENSO, and NAO.

Colora Apparent water color, in which water color is measured from unfiltered water in Pt-Co units.

Colort True water color, in which water color is measured from filtered water in Pt-Co units.

Column Refers to specific variables included in LAGOS (water chemistry, geospatial metric, unique identifiers, or metadata information).

CONN The category of geospatial features that are exported together in a single table that includes: stream density, lake and wetland connectivity metrics and glaciation.

Connectivity A lake having a surface hydrological connection with a lake, a river or stream or a wetland.

Controlled vocabulary A controlled vocabulary is an established list of standardized terminology for use in indexing and retrieval of information. An example of a controlled vocabulary is subject headings used to describe library resources (definition obtained from <http://stats.oecd.org>)

CSI Cross-scale interaction: a CSI occurs when a driver at one scale, such as local land use, interacts with a driver at another scale, such as regional climate. These CSIs can lead to nonlinear and often unexpected relationships between drivers and responses [1].

Cumulative watershed The entire area of land that drains directly into a lake via upstream connected permanent streams and lakes

CWA The US Clean Water Act regulates the discharge of pollutants to waters of the US The USEPA is mandated to implement water quality standards, monitoring programs, and standards for contaminants in surface water within this Act

Data flags (generic) Attribute associated with a specific variable and sampling event where the QAQC process identified a potential problem with a reported value. This attribute is given a flag characterizing the issue being generated and associated with the reported value.

Data model Specific design of the database with tables and links between them that implements the best practices for database design as outlined in the text.

Data provenance A record that details how a dataset was produced, all changes that were made to a dataset, and any other details required to analyze a dataset.

Data source Refers to the governmental, private organization, or individual who collected the data or maintains the database.

Data themes Groups of variables related either by the type, source, or spatial resolution of data that are combined in an individual table that is exported from LAGOS. Because there are so many variables in LAGOS, all of them cannot be exported into a single table and be useable by standard software packages. The themes for LAGOS_GEO_ data include: CHAG, LULC, CONN, CLIM_ANN, and CLIM_MON.

Dataset schema The structure of a dataset, i.e. characteristic arrangement of columns and rows that comprise a dataset.

DKN Dissolved Kjeldahl nitrogen

DOC Dissolved organic carbon

Drainage lake Lakes connected to surface water through inflowing streams, with no upstream lakes.

Drainage lake UPKL Lakes connected to surface water through inflowing streams, with at least one upstream lake ≥10 ha.

Ecoinformatics Ecological informatics, or ecoinformatics, is the development and application of computer technologies relevant to the management of ecological data and information. It is an emerging discipline focusing on research and development of structures and algorithms that improve communication, understanding, and management of ecological information. Ecoinformatics focuses on the development of software tools to manipulate, store, distribute, and display ecological information. The objective of ecoinformatics is to integrate data, knowledge, and methods necessary to provide ecological data to a scientific or policy-making process [2].

EDU Ecological drainage unit [3]

EML Ecological Metadata Language: standardized format for creating and storing database metadata described in [4].

ENSO El Niño Southern Oscillation; long-term oscillation in sea surface temperatures that influences temperature and precipitation in many parts of the globe.

Epilimnion Well-mixed, upper lake zone

Epilimnetic sample in LAGOS A sample taken from any of the following: the surface, just below the surface, an integrated zone of the entire epilimnion, or any single depth from within the epilimnion.

Federal Agency Federal level agency (e.g., US National Park Service (USNPS), US Geological Survey (USGS), US Environmental Protection Agency (USEPA)).

Forested wetland Wetlands dominated by woody vegetation 6 m or taller.

Freshwater features Surface water bodies including lakes, streams, and wetlands (groundwater can also be included in an expanded definition).

GEO Geospatial: spatially-explicit features characterizing climate, terrestrial, hydrologic, and anthropogenic elements of the landscape.

GIS "Geographic Information System: computer system designed to capture, store, manipulate, analyze, manage, and present all types of spatial or geographical data." [5]

GIS tools A GIS tool accepts spatial data as an input and performs one or more calculations, conversions, joins, comparisons, selections, summaries, or spatially aware analyses with those data to create new output data.

GitHUB Web-based repository hosting service providing version control.

Grab sample Water sample collected at the lake surface or at a discrete depth by lowering a sampler or sample bottle.

Graph-based patch metrics Spatial heterogeneity quantified on the basis of a hybrid of patch-mosaic and spatial graphs that explicitly incorporates topology to characterize landscape patches (used to compile connectivity metrics).

Headwater lake Lakes at the headwater of a stream network, with at least one outflowing stream.

Headwaters Subset of 'streams': stream line segments with Strahler number 1-3.

Horizontal data tables Data tables structured with a single row representing individual entities (such as lake and sample date). Named columns represent data values for descriptor variables. This type of data table is often called 'wide format'.

HUC Hydrological Unit Code; refers to several scales of geographical extents included within a common watershed [6]

Hypolimnion Thermally isolated bottom lake waters.

Integrated tube sampler Method for sampling an entire section of the lake water column using a tube lowered vertically.

Isolated lake Lakes with no inflow or outflow permanent streams.

IWS Interlake watershed: the area of land that drains directly into a lake. This area includes all upstream connected permanent streams to that lake, but does not include upstream lake watersheds for lakes >10 ha that are connected via permanent streams (see Additional file 12).

LAGOS: The name for the integrated geospatial-temporal database described in this study (**LA**ke multi-scaled **G**e**OS**patial and temporal database). It has two main components, LAGOS_GEO_ and LAGOS_LIMNO_

LAGOS_GEO_: The geographical component of LAGOS. It contains census data on climate, land use, landscape geomorphological features, etc. for all lakes in the study extent.

LAGOS_LIMNO_: The limnological component of LAGOS. It contains data for measured lake physical, chemical, and biological variables for a subset of the lakes found in the study extent.

LAGOS flag (all_lagosflag) A data field in LAGOS created by the database administrator using the data flags generated through the QAQC process.

LAGOS GIS toolbox A set of python script tools developed using ArcGIS 10.1 to create tools that automate GIS-related processes.

Lake: A ‘lake' in LAGOS is a perennial body of relatively still water. We include lakes and reservoirs that range from being completely natural to highly modified: a lake can be entirely natural, modified natural (i.e., a water control structure on a natural lake), or fully impounded stream or river (i.e., a reservoir). We explicitly exclude the following water bodies: sewage treatment ponds, aquaculture ponds or other such detention ponds that are known to contain basins that are entirely artificial and were built for one of these high-intensity human uses. The smallest size of lakes for which we have both limnology and geospatial representation (i.e., we know the location of the lake, and its general outline) is 4 ha; therefore, the operational minimum lake size in our database is 4 ha. For all lakes between 1 and 4 ha, there are very large errors in the NHD (see below for details). Therefore, we recommend caution in analyses, interpretation, and inference for lakes < 4 ha in size that depend on NHD's spatial representation of water bodies. In fact, even though we have some limnological data for lakes < 4 ha, we do not calculate any geographic data for those lakes.

Lake buffer Zone generated around a lake shoreline at an equidistant distance from the shoreline.

Lake hydrologic type Lake classification based on connectivity with other surface water ecosystems in which four classes were defined based on connectivity to streams and to other lakes: isolated, headwater, drainage, and drainage-upstream lake.

LakeID A unique identifier for each lake based on NHD and water body identifiers.

Lake order The order of a lake calculated and defined based on the order of the stream that flows out of the lake.

Landscape limnology The spatially explicit study of lakes, streams, and wetlands as they interact with freshwater, terrestrial, and human landscapes to determine the effects of pattern on ecosystem processes across temporal and spatial scales [7].

LTER Long Term Ecological Research Site (e.g., North Temperate Lakes LTER)

LTREB Long Term Research in Environmental Biology

LULC The category of geospatial features that are exported together in a single table that includes: land use and land cover, impervious surface, canopy cover, slope, terrain ruggedness index, road density and dam density.

Macrosystems ecology A sub-field of ecology that studies diverse ecological phenomena at the scale of regions to continents and their interactions with other types of phenomena (geophysical or social) and phenomena at other scales [8]

Metadata "Documentation describing all aspects of the data (e.g., who, why, what, when and where) that would allow one ot understand the physical format, content and context of the data, as well as possibly how to acquire, use and cite the data" [9].

Metalimnion Thermal transition zone between the lake epilimnion and hypolimnion.

Mid-reaches Subset of 'streams': stream line segments with Strahler number 4-6.

NA Designation used to indicate missing values in a database.

NAO North Atlantic Oscillation: Fluctuations in atmospheric pressure that control westerly winds and storm tracks in the north Atlantic.

NCAR The National Center for Atmospheric Research

NED National Elevation Database (used to derive slope)

NHD National Hydrography Dataset

NHDFlowline Geographical information system (GIS) layer within the NHD containing the information for identifying streams and rivers

NH_4_ Ammonium nitrogen

NLA National Lake Assessment

NO_2_ Nitrite nitrogen

NO_2_NO_3_ Nitrite + Nitrate nitrogen

Non-profit agency An organization that exists for charitable purposes, and from which its shareholders or trustees do not benefit financially

NSF US National Science Foundation

NWI National Wetland Inventory: Vector-based wetland delineation for the US with wetlands classified using high altitude imagery and on-the-ground verification.

Observational data model A relational database design produced by the CUAHSI community: “The observations data model is designed to store hydrologic observations and sufficient ancillary information (metadata) about the data values to provide traceable heritage from raw measurements to usable information allowing them to be unambiguously interpreted and used. A relational database format is used to provide querying capability to allow data retrieval supporting diverse analyses.” Definition obtained from http://his.cuahsi.org/odmdatabases.html.

Ontology "A formal representation or classification of concepts and their relationships within a domain of interest." [9]

Open water wetland Wetlands with open water habitat.

Palustrine wetland Geographical extent containing wetlands, which are defined to not be a lake, river, or stream, nor is tidal or contains saltwater. Palustrine wetlands are obtained from the National Wetlands Inventory database and are included in LAGOS_GEO_ but other types of wetland are not.

Patch-based metrics Spatial heterogeneity quantified as discrete, relatively homogeneous patches.

Point-based geostatistics Spatial heterogeneity quantified as continuous gradients across space.

PERL A programming language used to export geographic data from LAGOS.

PPT Precipitation

PRISM Long-term climate databases maintained by the US Department of Agriculture (USDA).

Program The organization or person that collected or provided data for LAGOS; there can be multiple sources of data from a single program (for example, a state agency such as the Wisconsin Department of Natural Resources has multiple data sources).

Provenance "In science, data provenance refers to the ability to track data from creation through all transformations, analyses and interpretations, enabling full understanding of the processes used to create derived scientific products." [9]

Python A programming language used to create code for implementing tools in ArcGIS.

QAPP Quality Assurance Project Plan required by the USEPA to document state water quality monitoring programs.

QAQC Quality assurance/ Quality control: "Refers to the mechanisms for preventing errors from entering a dataset that are used *a priori* to ensure high data quality before collection and to monitor and maintain data quality during and after the data collection process." [9]. Note, in our case, we also developed and implemented additional QAQC procedures once all data were fully harmonized in the integrated database.

Raster Representation of spatial data as attributes of equally-sized pixels that represent the landscape.

River Subset of 'streams', stream line segments with Strahler number > 6.

River order See Stream order

RivEX Proprietary river network tool that calculates Strahler stream order for each feature of the NHD Flowline shapefile within ArcGIS.

Sampling event Represents the measurement of a variable on a known date, in a known lake, and at a unique depth.

Scrub-shrub wetland Wetlands dominated by woody vegetation < 6 m tall.

Secchi A Secchi disk is a black and white disk attached to the end of a rope. It is used to measure water transparency by lowering into the water and the user records the depth at which it disappears.

Secondary data Derived data or data that have been modified, aggregated and/or quality controlled post-sampling.

Spatial extent Spatial boundaries of each of a set of nested distances or areas used to describe the landscape (i.e., lake buffer, lake catchment, HUC).

Spatial graphs Spatial heterogeneity quantified using mathematical constructs of space whereby nodes (points) and links (lines) model typology and connectivity.

SRP Soluble reactive phosphorus

State agency US state-level natural resource agency (e.g., Wisconsin Department of Natural Resources) responsible for managing and monitoring water bodies of the state (in addition to other responsibilities).

Strahler stream order A stream-reach classification scheme (also called river order) based on methods developed by Strahler [10]. In general, stream order increases as one moves down a stream network starting from the headwater stream to the terminal point.

STORET The STORET (short for STOrage and RETrieval) Data Warehouse is a repository for water quality, biological, and physical data and is used by state environmental agencies, EPA and other federal agencies, universities, private citizens, and many others (www.epa.gov/storet/).

Stream All NHD Flowline features except 1) coastlines and 2) artificial paths, and that are not stream centerlines. These flowlines were classified according to Strahler stream order.

Study extent Spatial boundary of the LAGOS database: 17 North Eastern US states comprising much of the glaciated, lake-rich area of the US.

TDN Total dissolved nitrogen

TDP Total dissolved phosphorus

TKN Total Kjeldahl nitrogen

TMAX Maximum temperature.

TMEAN Average temperature.

TMIN Minimum temperature.

TN Total nitrogen

TOC Total organic carbon

TON Total organic nitrogen

Tribal agency A federally recognized tribe is an American Indian or Alaska Native tribal entity that is recognized as having a government-to-government relationship with the United States, with the responsibilities, powers, limitations, and obligations attached to that designation, and is eligible for funding and services from the Bureau of Indian Affairs [11]. Many such tribes have natural resource agencies that sample water quality in water bodies on their lands or that they are able to access.

TP Total phosphorus

TRI Terrain ruggedness index

University researcher Data originates from individual research program within a University (e.g., Michigan State University).

Variable An individual limnological variable (such as total phosphorus, Secchi depth, etc.).

Vector Spatial data represented as geographic features such as points, lines, or polygons.

Vertical data tables Data tables structured with multiple rows for each entity (such as lake and sample date). One named column contain the descriptor variable name and another column contains the corresponding data value. This type of data table is often called 'long format'.

Water body feature class Defined within the NHD, including lakes, rivers, and wetlands.

Wetland classification Wetlands classified by spatial connectivity, vegetation composition, and water regime (see Additional file 7); a subset of the classes were includes in LAGOS_GEO_.

Wetland water regime Prevailing pattern of water flow (see Additional file 7).

XML Extensible Markup Language: encoding language used to create documents included in metadata within EML.

Zone We divided the study extent into zones, or polygons, that were delineated for different spatial extents.

ZoneID Identifier of the zone or spatial extent used to measure GEO data; used for the purpose of linking data tables of similar spatial extents.

**References**

1. Soranno PA, Cheruvelil KS, Bissell EG, Bremigan MT, Downing JA, Fergus CE, Filstrup CT, Henry EN, Lottig NR, Stanley EH, Stow CA, Tan P-N, Wagner T, Webster KE: **Cross-scale interactions: quantifying multi-scaled cause–effect relationships in macrosystems**. *Front Ecol Environ* 2014, **12**:65–73.

2. National Center for Ecological Analysis and Synthesis: **What is ecoinformatics?**. https://www.nceas.ucsb.edu/nceas-web/projects/2059/ecoinformatics.html.

3. Higgins JV, Bryer MT, Khoury ML, Fitzhugh TW: **A Freshwater Classification Approach for Biodiversity Conservation Planning**. *Conserv Biol* 2005, **19**:432–445.

4. Michener WK, Brunt JW, Helly JJ, Kirchner TB, Stafford SG: **Nongeospatial metadata for the ecological sciences**. *Ecol Appl* 1997, **7**:330–342.

5. **Geographic information system**. *Wikipedia, the free encyclopedia* 2014.

6. Seaber P, Kapinos F, Knapp G: **Hydrologic unit maps: U.S. Geological Survey water-supply paper 2294**. 1987.

7. Soranno PA, Cheruvelil KS, Webster KE, Bremigan MT, Wagner T, Stow CA: **Using Landscape Limnology to Classify Freshwater Ecosystems for Multi-ecosystem Management and Conservation**. *BioScience* 2010, **60**:440–454.

8. Heffernan JB, Soranno PA, Angilletta MJ, Buckley LB, Gruner DS, Keitt TH, Kellner JR, Kominoski JS, Rocha AV, Xiao J, Harms TK, Goring SJ, Koenig LE, McDowell WH, Powell H, Richardson AD, Stow CA, Vargas R, Weathers KC: **Macrosystems ecology: understanding ecological patterns and processes at continental scales**. *Front Ecol Environ* 2014, **12**:5–14.

9. Michener WK, Jones MB: **Ecoinformatics: supporting ecology as a data-intensive science**. *Trends Ecol Evol* 2012, **27**:85–93.

10. Strahler AN: **Hyposometric (area-altitude) analysis of erosional topography**. *Geol Soc Am Bull* 1952, **63**:1117.

11. U.S Department of the Interior Indian Affairs: **Frequently Asked Questions**. http://www.bia.gov/FAQs/.
