# Supplementary material for: Building a multi-scaled geospatial temporal ecology database from disparate data sources: fostering open science and data reuse
Source: Gigascience. 2015 Jul 1;4:28. doi: 10.1186/s13742-015-0067-4 (PMC4488039; doi:10.1186/s13742-015-0067-4)
Supplement: Additional file 2: — Research decisions that guided the creation of LAGOS. A description of the major decisions and rationale in the creation of LAGOS that were related to the research questions that LAGOS was designed to answer, the conceptual framework for the project, and the longterm plans for the database. [file 13742_2015_67_MOESM2_ESM.docx]

Additional file 2

**Research decisions that guided the creation of LAGOS**

Patricia Soranno, Kendra Spence Cheruvelil, Emily H. Stanley, Noah R. Lottig, John A. Downing, Pang-Ning Tan, Ty Wagner, Katherine E. Webster, and Mary Tate Bremigan

**Overview**

The primary scientific rationale for constructing LAGOS, the **LA**ke multi-scaled **G**e**OS**patial and temporal database, was to create an integrated database designed to answer basic research questions in macrosystems ecology related to cross-scale interactions (CSIs) influencing lake water quality. The design and structure of LAGOS reflects a landscape limnology conceptual framework that considers freshwater locations as elements within a patch-mosaic model that are defined by their patch context (i.e., geomorphology and human landscapes), and patch connectivity (e.g., hydrologic connections among lakes, streams, and wetlands). This framework considers specific freshwater responses and states as influenced by drivers acting at multiple and hierarchically structured spatial scales. CSIs exist when drivers at one scale interact with drivers at another scale to modify relationships with freshwater response variables, and are of general interest because they often lead to nonlinear and unexpected relationships between drivers and response variables. The design of LAGOS thus had to be flexible enough to integrate very large lake chemistry datasets derived from multiple and idiosyncratic sources; ensure that records for individual lakes were georeferenced accurately and metadata were carefully documented; and provide linkages between lakes and potential drivers in a multi-thematic, multi-scaled landscape database appropriate for answering our core CSI-related research questions.

Here we describe the research questions and conceptual framework that underpinned the development of LAGOS which combines lake quality (nutrients, water chemistry, water clarity) datasets from a diverse range of sources, with geospatial data generated in a consistent way for all lakes larger than 4 ha within a 17-state, lake-rich region of the north-eastern US. This unprecedented data effort has produced a database that, while initially designed to meet the goals of this specific NSF-funded macrosystems project, also provides a valuable ecological legacy for the future both as a framework for including additional lake datasets thereby expanding the study extent, and as a publically available resource of tremendous value to managers, researchers, and public users.

**Description of our funded project**

We initiated the project to build LAGOS in order to answer ecological questions about lake water quality at the sub-continental scale in the US. This effort began as an NSF-funded project initiated to build the database from scratch and consequently, we began with our research questions. As the project progressed, the clear articulation of these research questions guided the subsequent database development, in part because every decision about what data to include in the database, particularly data that required manual editing/searching, depended on the importance of the variable in addressing our research questions.

*The effects of cross-scale interactions on freshwater ecosystem state across space and time. PIs: PA Soranno, KS Cheruvelil, JA Downing, NR Lottig, EH Stanley, PN Tan. NSF Emerging Frontiers Division, Macrosystems Biology Program. 2011-2016.*

Understanding CSIs is a critical research frontier in the field of macrosystems ecology that extends across all of the environmental sciences. A CSI exists where a driver at one scale, such as local land use, interacts with a driver at another scale, such as regional climate. These CSIs can lead to nonlinear and often unexpected relationships between drivers and responses. Unfortunately, general properties of CSIs, such as the conditions in which they occur, are largely unknown, partly because they have been quantified in only a few instances. We use lakes and their major nutrients (phosphorus, nitrogen, and organic carbon) as a model system because lakes are affected by many of the main classes of drivers postulated to be a part of CSIs (e.g., connectivity, land use, and climate), and because there is a wealth of existing data and knowledge from small-scale studies regarding possible mechanisms through which drivers could interact across scales. We use this model system to ask our primary research question: ***What are the cross-scale interactions that regulate spatial heterogeneity and temporal dynamics of lake nutrients at sub-continental scales?***

Despite the high probability that CSIs influence lake nutrients, lakes have not been studied in the fully spatially explicit fashion that is required to quantify CSIs, in part because of a lack of a suitable, comprehensive, multi-scaled spatial framework. Our landscape limnology conceptual model fulfils this requirement, however. It is based on principles of landscape and freshwater ecology [1] and organizes drivers into multi-scaled spatial and temporal classes. Landscape limnology provides a unique lens for understanding how drivers from different scales and classes interact to create CSIs that affect freshwater ecosystems.

Our overall research strategy is to: (1) use the landscape limnology conceptual framework to guide research; (2) assemble a lake nutrient and multi-scaled landscape database that has unprecedented spatial and temporal coverage (~5,000 lake ecosystems in 11 states spanning 25 years); and (3) use robust statistical approaches to quantify relationships across scales and integrate the spatial and temporal domains. One of the analytical approaches we will use is multi-level (i.e., hierarchical) modelling. This approach quantifies CSIs, allows for explicit testing of alternative potential drivers responsible for CSIs, and facilitates the effective communication of the complex dynamics driving the ecosystem state.

**Research questions**

Derived from our primary research question stated above, we focused on three research questions that we wanted to answer with the LAGOS databases:

1. At which scales do CSI drivers explain spatial heterogeneity in lake water quality?
2. At which scales do CSI drivers explain the temporal dynamics in lake water quality across regions?
3. What are the relative contributions of spatial and temporal drivers to CSIs that explain spatial and temporal variation in lake water quality?

These three questions emphasized the important components that we needed to include in our database:

- Broad spatial extent of the study area, to include lakes along gradients of driver variables (such as land use, climate, and geology).
- Broad temporal extent of the sampled lakes to find as much current and historical data as possible.
- Multi-scaled spatial analysis of driver variables that include measures of these variables at very fine scales (near the lakes), and measures that defined the regions that the lakes are nested within.
- In-lake variables for water quality (nutrients, water chemistry, and water clarity).

These components determined what variables we needed to prioritize in collecting and integrating databases from a wide range of available data sources. For example, when we acquired data from data providers, they often sent us additional sampled variables, particularly data such as water temperature and dissolved oxygen data sampled at many depths. Processing such data takes much more time than processing chemical data, which is often sampled at a single depth. Because water temperature and dissolved oxygen were not directly related to our immediate research questions, we decided to not devote project resources to manipulating such data. This decision allowed us to focus resources on other, more critical, aspects of the database development.

**Conceptual model underlying LAGOS:**

***The landscape limnology conceptual framework***

We based our research questions and database development on a landscape limnology conceptual framework [1], modified to include a more strongly temporal component as shown in Figure S1 [2, 3]. This framework is based on core landscape ecology principles whereby freshwaters are viewed via a patch-mosaic model and defined by their patch context (i.e., geomorphology and human landscapes), and patch connectivity (e.g., hydrologic connections among lakes, streams, and wetlands) across a range of spatial scales. We incorporate a temporal component in this framework by including climate and atmospheric deposition.


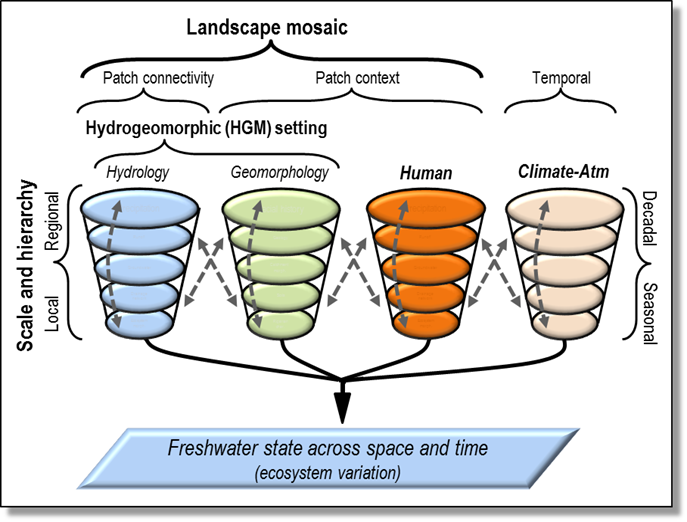


**Figure S1**. **Description of the landscape limnology conceptual framework used to guide LAGOS database development.** Modified from [1]. Freshwater state refers to the response variables of interest, such as lake nutrients. Ovals represent examples of hierarchically organized drivers of freshwater ecosystem variation within each driver class. The gray dashed arrows represent CSIs between or within CSI driver classes. Note that interactions can occur within scales and among all combinations of driver classes but these are not all represented graphically. Climate-Atm, climate and atmospheric deposition.

Based on this framework, we sought to develop a comprehensive lake and landscape database that would incorporate potential driver variables (from geospatial data sources in GIS format) from each of the four main categories of landscape feature. Where available, temporal data were included (for example annual climate variables, annual nitrogen deposition, and land use/cover from different time periods), and the database was built to represent as many of the variables as possible at the full range of potential spatial scales. Because not all of the desired data were available, and in some cases the datasets were not suited to this research project, we made choices regarding the data with reference to the research questions.

LAGOS consists of two modules: LAGOS_LIMNO_, which is the portion of the database that includes the in-lake sampling data along with lake-specific information (such as depth, lake name, and location); and LAGOS_GEO_, which includes the data extracted from the geospatial data sources. Because ultimately we needed to merge the LAGOS_GEO_ database with LAGOS_LIMNO_, we needed to calculate geospatial metrics that quantified the landscape features and linked them to specific lakes for statistical analysis.

**Availability of data for building LAGOS**

Lake ecosystems, and particularly lake nutrients and other measures of water quality, provide a unique and rich source of data to conduct macrosystems ecology research. In the US, state agencies are mandated by the USEPA to report the nutrient status of their water bodies under the Clean Water Act, and so must sample and monitor them using standard protocols to meet quality assurance requirements. These data are publicly available, although there have been few efforts to integrate such data across states, or to combine the data with a multi-scaled landscape database. Therefore, at the outset of this project, we anticipated that there would be available data, particularly dating from the late 1970s or 1980s when lake sampling programs were being initiated with USEPA funds. In addition, we knew of many available sources of geospatial data on a national scale which could be used to build LAGOS_GEO_ including data on land use/cover, geology, topography, climate, nitrogen deposition, and freshwater location and connectivity. See Additional file 19 for a description of the datasets compiled for LAGOS_LIMNO_, and Additional files 7, 9-13 for a description of the datasets compiled for LAGOS_GEO_.

Because our main goal was to study long-term (inter-annual) rather than short-term (intra-annual) patterns and trends we were interested in collecting data from the summer period corresponding to the period of maximum biological activity, and from the surface waters of the lake (the epilimnion), because that is where most of the biological activity occurs.

**Short-term and long-term plans for LAGOS**

Our short-term goal in building LAGOS was to develop an integrated geospatial temporal macrosystems ecology database to answer basic research questions about macrosystems ecology in relation to CSIs (see above). The choice of predictor and response variables were guided by these research questions, together with a conceptual model that explains how spatial and temporal variations in lake water quality are influenced by geospatial drivers. However, we also recognized early on that this database could form the foundation to ask alternative research questions on lake ecosystems through the addition of more data (e.g., data from in-lake sampling programs for lake nutrients and water quality), or new response or predictor variables. Our long-term goal was to design the database so that it could accommodate new lake variables and new geospatial data that might be needed in the future and we thus required the database design to be able to accommodate both the short- and long-term goals. Finally, we also intended to make the database publicly accessible at the end of the project and so we generated documentation throughout the process to ensure the correct use and provenance of the underlying data.

**Identifying the metadata and documentation needs for LAGOS**

Any data use effort should ideally compile and document metadata on the individual datasets. For databases in which different data are being combined into a single database, the decisions regarding how to document and integrate the metadata are less clear as there are few relevant standards for such a process integration. For example, we wanted to include in LAGOS the metadata that is frequently required in the analysis of lake chemistry. It is not possible however to include all of the information provided in a standard metadata file for ecological data such as EML (ecological metadata language). In addition, our long-term plans for LAGOS meant that we had to carefully document all steps in the data integration process so that other researchers could both conduct robust research on the existing dataset, and also be able to add new data to it.

**References**

1. Soranno PA, Cheruvelil KS, Webster KE, Bremigan MT, Wagner T, Stow CA. Using landscape limnology to classify freshwater ecosystems for multi-ecosystem management and conservation. BioScience. 2010;60:440-54.

2. Lottig NR, Carpenter SR. Interpolating and forecasting lake characteristics using long-term monitoring data. Limnol Oceanogr. 2012;57:1113-25.

3. Robertson GP, Collins SL, Foster DR, Brokaw N, Ducklow HW, Gragson TL, *et al*. Long-term ecological research in a human-dominated world. BioScience. 2012;62:342-53.
