# Supplementary material for: Building a multi-scaled geospatial temporal ecology database from disparate data sources: fostering open science and data reuse
Source: Gigascience. 2015 Jul 1;4:28. doi: 10.1186/s13742-015-0067-4 (PMC4488039; doi:10.1186/s13742-015-0067-4)
Supplement: Additional file 3: — Creating integrated metadata for LAGOSLIMNO. The approaches that were used to document multiple forms of metadata for LAGOSLIMNO. [file 13742_2015_67_MOESM3_ESM.docx]

Additional file 3

**Creating integrated metadata for LAGOS_LIMNO_**

Emi Fergus, Ed Bissell

**Overview**

We created an overarching metadata document, referred to as LAGOS_LIMNO_ integrated metadata, that documents in a standardized format all of the individual lake sampling datasets populating the LAGOS_LIMNO_ database. The objective was to assemble and harmonize individual metadata into a fully comparable format that was ultimately included in the final database. Our metadata strategy for LAGOS_LIMNO_ serves multiple purposes: 1) to record information on the content, context, quality, structure, and accessibility of the individual datasets; 2) to standardize limnological variable names and measurement units across the datasets; and 3) to facilitate data importation into the LAGOS_LIMNO_ database by organizing data content and structure. Comprehensive metadata documentation should promote the appropriate utility and longevity of the LAGOS database for current and future users.

**Background**

LAGOS_LIMNO_ compiles limnological data from multiple, independent lake sampling programs and data providers into an integrated database that spans broad spatial and temporal domains. The original datasets come from federal, state, and tribal agencies; university researchers; citizen monitoring programs; and non-profit organizations. The datasets contain thematically similar limnological data on water quality measurements, but the context and purpose of the sampling program, geographic and temporal range, and field and laboratory methods are different from program to program. Thus, comprehensive metadata documentation of the individual datasets is necessary to appropriately integrate the datasets into the database, to promote proper use of the data, and to understand the underlying complexity and heterogeneity of LAGOS_LIMNO_.

We created an overarching metadata document, LAGOS integrated metadata, to accompany the LAGOS database that details information on the individual lake datasets such as the source of the data, purpose behind the data collection, geographic and temporal ranges represented by the data, limnological variables and units of measurement, sampling and analytical methods, data anomalies, and other pertinent information necessary for appropriate use and integration of the datasets into LAGOS_LIMNO_.

**LAGOS metadata documentation process**

The creation of LAGOS_LIMNO_ metadata documentation followed several steps to compile relevant information from original lake datasets into one standardized, structured metadata workbook. We modeled our metadata collection after Ecological Metadata Language (EML) and made modifications to fit the data characteristics and objectives of LAGOS_LIMNO_. Below we outline the steps that we used to create the metadata documentation.

**Collecting metadata from original data source**

At the stage of data collection from individual data sources, we requested detailed metadata to accompany the dataset. This information was necessary to evaluate the suitability of the data to be included in LAGOS_LIMNO_, and to provide adequate information to fully document the individual dataset so as to ensure proper use and interpretation. The metadata provided by the source came in many forms such as annual reports, program notes, field and laboratory handbooks, and EML files. We contacted data providers if additional information was needed.

**Creating a LAGOS_LIMNO_-specific metadata document for individual datasets**

We filtered specific information from the original metadata documents following categories similar to EML structure. EML is a metadata standard developed by ecologists [1]. For each dataset we recorded information on the data source agency, organization, or individual (e.g., contact person, website links, funding source); on the specific data sampling program (e.g., purpose for collecting the data, how to cite the data, geographic and temporal range, status of the program, and accessibility of the data); and on the limnological variables recorded (e.g., variable names, units of measurement, detection limits, field and laboratory methods, and data quality).

**Creating EML-formatted metadata for individual datasets**

In addition to creating our LAGOS_LIMNO_ specific metadata individual documents, we also created EML formatted metadata files for each data program using Morpho software to promote data documentation standards. In some instances, EML files were provided by the data source, but for the majority of programs we created the EML files and saved them separately.

Morpho is a free software program available through The Knowledge Network for Biocomplexity that facilitates standard EML metadata creation [2]. The EML formatted metadata files are stored as a series of XML documents that describe modular parts of the metadata.

**Creating LAGOS integrated metadata**

We combined metadata from individual datasets into the LAGOS_LIMNO_ integrated metadata, which is stored as an Excel workbook. The LAGOS_LIMNO_ integrated metadata organizes key information about each of the individual datasets into four different categories: Data Source, Program Description, Metadata, and Variables. This information was used for data importation and integration into the LAGOS database. Table S33 provides detailed descriptions of the structure and components of the LAGOS_LIMNO_ integrated metadata document.

We standardized attributes of the data such as limnological variable names across the different programs and prioritized variables to integrate into the database. LAGOS controlled vocabulary words and phrases that were used to standardize the datasets are stored in Additional file 4. Standardizing the data attributes was an important step to be able to integrate information from disparate programs into one coherent document.

Creating the LAGOS_LIMNO_ integrated metadata required many manual hours of work to process each individual lake dataset. We could not automate this step because lake datasets varied in data content and structure. We logged the time spent to process each lake dataset to gauge the time required to complete this step. The amount of time to process a lake dataset varied by program type and was related to a number of factors such as the number of variables in the dataset and the data table structure and organization. On average it took 3.7 hours to process an individual lake dataset. Federal, State, and Tribal lake sampling programs took the most time to process (Federal = 5.4 hours, State = 4.4 hours, and Tribal = 5 hours) and University took the least amount of time (2.2 hours).

**LAGOS_limno_ integrated metadata Descriptors**

Below is a table describing the metadata format and descriptors to organize the *LAGOS integrated metadata*.

Table S33. LAGOS_LIMNO_ integrated metadata descriptors

| **Metadata**  **Worksheet** | **LAGOS Metadata descriptor** | **Description** |
| --- | --- | --- |
| **Data source** |  |  |
|  | SourceID | Unique ID for the data collection organization |
|  | SourceName | Name of organization that collected or is otherwise responsible for the data. Follows a specific format: State initials_Organization initials Example: IL_EPA |
|  | SourceDescription | Description of data collection organization |
|  | Comments | Information about the data collection organization |
| **Data program** |  |  |
|  | ProgramId | Unique ID for the program |
|  | SourceName | Name of organization that collected or is otherwise responsible for the data (this is duplicate information from the above worksheet) |
|  | ProgramName | Name of sampling program that the data were collected under. Follows a specific format: State initials _ program initials_years (if the year differentiates between programs) |
|  | Composite | Specifies whether the data program is a composite of data from multiple sampling programs. 1 = Yes, 0 = No |
|  | ProgramType | Specifies the type of organization or agency conducting and running the program. Example: Federal Agency, State Agency |
|  | FundingSource | Specifies the type of organization or agency funding the program. Example: NSF, NSF-LTER |
|  | DataSharingPolicy | Specifies data-sharing policies as specified by the individual sharing the data |
|  | DataSharingPolicyDetails | Details on data sharing policy |
|  | ProgramDescription | Name of sampling program that the data was collected under. Follows a specific format: Source name (initials): program name_years |
|  | LabType | Indication of whether the variable is analyzed at a federal, state, university or private laboratory |
|  | ProgramLink | A link to the program website |
|  | ProgramStatus | Whether the program is still collecting data or is complete |
|  | DatabaseComments | Any additional information about the program that should be noted in LAGOS database |
|  | Comments | Any additional information (this may be extraneous to LAGOS) |
| **Metadata** |  |  |
|  | MetadataId | Unique ID for the program |
|  | ProgramName & EMLFileName | Name of sampling program that the data were collected under (duplicate from above worksheet) |
|  | Title | Unique identifying title for metadata record |
|  | Abstract | Describes the particular data that are being documented and can include the objectives, design or methods of the data collection/study |
|  | Citation | How the data collecting organization prefers to be cited, if stated |
|  | MetadataLink | Link to eml file that was authored for Source/Program, just specify eml filename |
|  | TemporalScale | Years of the program |
|  | Comments | Any additional information about the program that should be noted in LAGOS database |
|  | ExtraneousComments | Any additional information (this may be extraneous to LAGOS) |
| **Variables** |  |  |
|  | Status | Prioritization of variable to be included in LAGOS database (D = Drop (never to be included in LAGOS), P = Priority (the first group of variables identified to be loaded into LAGOS), N = NonPriority (variables that may be loaded in the future, but not in the first several versions of LAGOS), M = Morphometry (variables measuring lake morphometry and given a high priority) |
|  | ProgramName | Name of sampling program that the data was collected under (duplicate from above) |
|  | SourceVariableName | Variable name as recorded in the data source (not standardized) |
|  | LAGOS-VariableName | Standardized variable name. This field was populated using a detailed list of water quality variable names using the controlled vocabulary. The detailed list of variable names is in Additional file 4. |
|  | StandardizedLAGOS-VariableName | Standardized and condensed variable name used to populate LAGOS database. Water quality variables that measure similar components were condensed together into general variable names as deemed appropriate by expert opinion. A list of controlled vocabulary terms is in Additional file 4. |
|  | LAGOS-VariableUniqueID | Unique code for Standardized LAGOS Variable Name |
|  | MethodInfo | Provides information about the variable methods in particular if there are methods that need to be flagged for consideration (standardized) |
|  | SamplePosition | Provides information about the location in the water column the sample was taken (standardized). Epi = Epilimnion, META = metalimnion, HYPO = hypolimnion, SPECIFIED = specified depth (also includes Secchi and profile samples), and UNKNOWN = not specified where the sample was collected. |
|  | VariableDescription | Detailed description of the variable (not standardized). |
|  | LabMethodName | Standardized record of the name of laboratory processing procedure, possibly from a standards body. If there are multiple laboratory methods listed we state 'MULTIPLE'. Example: EPA_312.5; APHA_5310B |
|  | LabMethodInfo | A field for more descriptive explanation of lab analysis |
|  | SourceVariableUnits | Original measurement units that sample was collected in as reported by the data source |
|  | LAGOS-UnitsName | ODM standardized units for the original units in which the sample was measured |
|  | PreferredLAGOS-Units | Preferred units for LAGOS database using standardized ODM controlled vocabulary |
|  | LAGO-UnitsUniqueID | Unique ID for preferred units for LAGOS |
|  | FORMULA | Conversion formula to convert from original units to LAGOS preferred units. NULL = no conversion necessary |
|  | SampleType | Indicates the sample type. GRAB = grab sample taken from a single depth, INTEGRATED = sample taken from multiple depths using a tube sampler that integrates the water column to a determined depth or Secchi depth, PROBE =sample taken from probe, UNKNOWN = sample type is unknown, MULTIPLE = more than one method used, SPECIFIED = sample type is specified in the original data, and NULL = non-water quality variables e.g., lake morphometric variables |
|  | DetectionLimit | Reports the measurement detection limits. Programs that changed laboratory methods over time report detection limits and the year they applied |
|  | Comments | Any additional comments with regards to the variable |

**References**

1. Michener WK, Brunt JW, Helly JJ, Kirchner TB, Stafford SG. Nongeospatial metadata for the ecological sciences. Ecol Appl. 1997;7:330-42.

2. KNB Repository - The Knowledge Network for Biocomplexity. https://knb.ecoinformatics.org/index.jsp. Accessed 19 May 2015.
