# Supplementary material for: Building a multi-scaled geospatial temporal ecology database from disparate data sources: fostering open science and data reuse
Source: Gigascience. 2015 Jul 1;4:28. doi: 10.1186/s13742-015-0067-4 (PMC4488039; doi:10.1186/s13742-015-0067-4)
Supplement: Additional file 4: — Controlled vocabulary for LAGOSLIMNO. A list of standardized vocabulary used to translate each of the disparate individual datasets into a common vocabulary for the data itself (i.e., the variable names) as well as for the metadata. [file 13742_2015_67_MOESM4_ESM.docx]

Additional file 4

**Controlled vocabulary for LAGOS_LIMNO_**

Emi Fergus, Ed Bissell

**OVERVIEW**

Descriptive metadata are essential to facilitate data sharing with end users and to preserve the integrity of datasets over time. This is especially true where individual datasets are integrated into large databases. Because individual datasets can use agency, or program-specific vocabularies, it is necessary to standardize the descriptive information they contain into a common controlled vocabulary when compiling disparate datasets into a database. The purpose of this document is to define the vocabulary used to translate individual datasets into the single vocabulary used in the LAGOS_LIMNO_ database. This document also describes how we standardize and document metadata from each source. We created a controlled vocabulary for LAGOS_LIMNO_ by downloading the CUAHSI ODM controlled vocabulary [1] and modifying it to our requirements. We made use of the tables called *units, VariableNameCV* and *SpeciationCV*. In addition, we documented each of the individual datasets by populating information into worksheets for the program, the metadata, and the variables. The program worksheet contains information on the program type (e.g., federal, state, tribal, university), the funding source (e.g., federal, state, private), data sharing policies associated with the dataset (i.e., whether or not the data are in the public domain), a brief description of the program, laboratory type (e.g., federal, state, private), and program status (i.e., ongoing or completed). The metadata worksheet contains information on the program organization names, a brief description of the program, and the number of years funded. The variables worksheet contains information associated with sample collection and analytical techniques used, including but not limited to the standardized variable name, analytical method name, the vertical position of the sample in the water column (e.g., epilimnion or hypolimnion), and the sample type (e.g., grab, integrated, probe).

**PROGRAM WORKSHEET**

***ProgramType***

**Table S2. Lake sampling program type controlled vocabulary phrases**

| **Term** | **Definition** |
| --- | --- |
| Federal Agency | Federal Agency (e.g., US National Park) |
| National Survey Program | National Survey Program (e.g., EPA National Lake Survey) |
| State Agency | State Agency (e.g., Wisconsin Department of Natural Resources) |
| Tribal Agency | Tribal Agency (e.g., Grand Portage Band of Lake Superior Chippewa Water Quality Program) |
| University | University (e.g., Michigan State University) |
| LTER | Long Term Ecological Research Site (e.g., North Temperate Lakes LTER) |
| Citizen Monitoring Program | Citizen or Volunteer Sampling Program (e.g., New York Citizens Statewide Lake Assessment Program) |
| Non-Profit Agency | Non-Profit Agency (e.g., Michigan Leelanau Conservancy Lakes Program) |
| State Agency/Citizen Monitoring Program | Combined State Agency and Citizen Monitoring Program (e.g., Maine Department of Environmental Protection Lake Monitoring and Assessment) |
| State Agency/University/Citizen Monitoring Program | Combined State Agency, University, and Citizen Monitoring Program (e.g., Michigan Cooperative Lakes Monitoring Program) |
| Federal Agency/University | Combined Federal Agency and University (e.g., Paul Lake Cascade Project) |

***FundingSource***

**Table S3. Funding source controlled vocabulary phrases**

| **Term** | **Definition** |
| --- | --- |
| Federal Agency | Federal Agency |
| State Agency | State Agency |
| NSF | National Science Foundation |
| NSF-LTREB | National Science Foundation – Long Term Research in Environmental Biology |
| NSF-LTER | National Science Foundation – Long Term Ecological Research |
| EPA | Environmental Protection Agency |
| EPA-Long-term monitoring | Environmental Protection Agency Long term monitoring |
| EPA-National Lake Survey | Environmental Protection Agency National Lake Survey |
| Tribal Agency | Tribal Agency |
| Non-Profit Agency | Non-Governmental Non-Profit Agency |
| Unknown | Funding source not known |
| Private | Consultant company, other |
| Federal/State Agency | Federal/State Agency partnership |
| State Agency/University | State Agency/University partnership |
| Varied | Multiple/various funding sources |
| EPA/University | EPA/University funding |

***DataSharingPolicy***

**Table S4. Data sharing policy controlled vocabulary phrases**

| **Term** | **Definition** |
| --- | --- |
| Public |  |
| Synthesis Only | Data to be used only in synthesis, not independently |
| Public-request | Data are public but there are requests associated with sharing – see comments for specific requests |
| Public-restrictions | Data are public but there are some restrictions – see comments for specific restrictions |

***ProgramDescription***

General format for *ProgramDescription*: Organization name (state abbreviation): description of program (if applicable), years

***LabType***

**Table S5. Laboratory type controlled vocabulary phrases**

| **Term** | **Definition** |
| --- | --- |
| Federal | Laboratory samples are processed at a Federally owned laboratory |
| State | Laboratory samples are processed at a State owned laboratory |
| University | Laboratory samples are processed at a University or Faculty laboratory |
| Private | Laboratory samples are processed at a privately owned laboratory (e.g., consulting firm) |
| Not Applicable | Sample is not processed in a laboratory (e.g., Secchi) |
| Unknown | Location of laboratory sample processing is not known |
| Varied | Laboratory samples are processed at multiple laboratory types |

***ProgramStatus***

**Table S6. Program status controlled vocabulary phrases**

| **Term** | **Definition** |
| --- | --- |
| Unknown | Not known if sample program is completed or ongoing |
| Ongoing Program | Sample program is ongoing |
| Program Completed | Sample program is completed |

**METADATA WORKSHEET**

***Title***

General format for *Title* follows *ProgramDescription*: Organization name (state abbreviation): description of program (if applicable), years

**VARIABLES WORKSHEET**

***Status***

All limnological variables were assigned a priority status based on the objectives of LAGOS: D = Drop, P = Priority, N = NonPriority, M = Morphometry.

***LAGOS-VariableName***

Water chemistry variables were given standardized names from a list of controlled vocabulary words listed in the Controlled Vocabulary LAGOS-VariableName column below.

***StandardizedLAGOS-VariableName***

Water chemistry variables were aggregated to one variable name where it was deemed appropriate by limnologists and biogeochemists. These aggregated variables are listed in the LAGOS-Standardized-VariableName column below. DROP indicates that the variable was not included in the final database.

***LAGOSVariableUniqueID***

Each aggregated variable name was assigned a unique variable ID.

**Table S7**. **Controlled vocabulary for limnological variables, aggregated variable names, unique ID, and priority status**

| **Controlled Vocabulary**  **LAGOS-VariableName** | **StandardizedLAGOS- VariableName** | **VariableID** | **Status** |
| --- | --- | --- | --- |
| Acid neutralizing capacity | Alkalinity | 1 | N |
| Alkalinity |  |  |  |
| Alkalinity, total |  |  |  |
| Alkalinity, carbonate |  |  |  |
| Alkalinity, bicarbonate | Alkalinity, bicarbonate | 2 | N |
| Anion | DROP |  | D |
| Anions | DROP |  | D |
| Calcium | Calcium | 3 | N |
| Carbon, dissolved inorganic | Carbon, dissolved inorganic | 4 | N |
| Carbon, total inorganic | Carbon, total inorganic | 5 | N |
| Carbon, dissolved organic | Carbon, dissolved organic | 6 | P |
| Carbon, total organic | Carbon, total organic | 7 | P |
| Cation | DROP |  | D |
| Cations | DROP |  | D |
| Cations-Anions | DROP |  | D |
| Chloride | Chloride | 8 | N |
| Chlorophyll (a+b+c) | Chlorophyll a | 9 | P |
| Chlorophyll a |  |  |  |
| Chlorophyll a corrected for pheophytin |  |  |  |
| Chlorophyll a, corrected for pheophytin |  |  |  |
| Chlorophyll a, corrected for pheophytin |  |  |  |
| Chlorophyll a, uncorrected for pheophytin | Chlorophyll a, uncorrected for pheophytin | 10 | P |
| Chlorophyll, b | DROP |  | D |
| Chlorophyll, pheophytin | DROP |  | D |
| Color, apparent | Color, apparent | 11 | P |
| Color, true | Color, true | 12 | P |
| Color, true spec |  |  |  |
| Conductance, specific | Conductivity | 13 | N |
| Conductivity |  |  |  |
| Magnesium | Magnesium | 14 | N |
| Nitrogen, dissolved Kjeldahl | Nitrogen, dissolved Kjeldahl | 15 | P |
| Nitrogen, total Kjeldahl | Nitrogen, total Kjeldahl | 16 | P |
| Nitrogen, nitrite (NO2)* | Nitrogen, nitrite (NO2) | 17 | P |
| Nitrogen, nitrate (NO3) | Nitrogen, nitrite (NO2) + nitrate (NO3) | 18 | P |
| Nitrogen, nitrite (NO2) + nitrate (NO3) |  |  |  |
| Nitrogen, dissolved nitrate (NO3) |  |  |  |
| Nitrogen, dissolved nitrite (NO2) + nitrate (NO3) |  |  |  |
| Nitrogen, NH3 | Nitrogen, NH4 | 19 | P |
| Nitrogen, NH3 total |  |  |  |
| Nitrogen, NH4 |  |  |  |
| Nitrogen, total organic | Nitrogen, total organic | 20 | P |
| Nitrogen, total | Nitrogen, total | 21 | P |
| Nitrogen, total dissolved | Nitrogen, total dissolved | 22 | P |
| Oxygen, dissolved | Oxygen, dissolved | 23 | N |
| pH | pH | 24 | N |
| pH, closed | pH, closed | 25 | N |
| pH, equilibrated | DROP |  | D |
| Phosphorus, particulate | DROP |  | D |
| Phosphorus, orthophosphate | Phosphorus, soluble reactive orthophosphate | 26 | P |
| Phosphorus, soluble reactive |  |  |  |
| Phosphorus, total | Phosphorus, total | 27 | P |
| Phosphorus, total dissolved | Phosphorus, total dissolved | 28 | P |
| Potassium | Potassium | 29 | N |
| Secchi | Secchi | 30 | P |
| Secchi, no view |  |  |  |
| Secchi, unknown |  |  |  |
| Secchi, view |  |  |  |
| Silica | Silica | 31 | N |
| Sodium | Sodium | 32 | N |
| Solids, total suspended | Solids, total suspended | 33 | N |
| Sulfate | Sulfate | 34 | N |
| Temperature | Temperature | 35 | N |
| Turbidity | Turbidity | 36 | N |

***MethodInfo***

Variables with flagged methods were noted here with the following standardized notation.

**Table S8. Flagged method controlled vocabulary phrases**

| [**V**](javascript:__doPostBack('dgCV_11$ctl01$ctl00',''))**ariable** | [**Description**](javascript:__doPostBack('dgCV_11$ctl01$ctl01','')) | [**Flagged Notation**](javascript:__doPostBack('dgCV_11$ctl01$ctl01','')) |
| --- | --- | --- |
| Alkalinity | Alkalinity measurements by gran titration were noted | ALK_GRAN_TITRATION |
| Secchi | Secchi depth measurements with a view scope were noted | SECCHI_VIEW |
| Secchi | Secchi depth measurements where it was not known if used a view scope | SECCHI_VIEW_UNKNOWN |

***SamplePosition***

The position in the water column where the sample was collected.

**Table S9. Sample position controlled vocabulary phrases**

| [**Term**](javascript:__doPostBack('dgCV_11$ctl01$ctl00','')) | [**Definition**](javascript:__doPostBack('dgCV_11$ctl01$ctl01','')) |
| --- | --- |
| EPI | Epilimnion (this also includes surface samples, euphotic zone, upper 2 m of surface water) |
| META | Metalimnion (also includes samples collected from 'mid-depth') |
| HYPO | Hypolimnion (also includes samples collected from 'bottom') |
| SPECIFIED | Specified depth (also includes Secchi and profile samples) |
| UNKNOWN | Not specified where sample was collected |

***LabMethodName***

General format to record laboratory method names: All caps, no spaces, no dashes, and underscore between organization abbreviation and method number. Ex) 'EPA_531.2'.

For variables with multiple methods: 'MULTIPLE'

***LAGOS-UnitsName***

Measurement units and unique ID based on CUAHSI Observations Data Model (ODM) format.

**Table S10. ODM standardized measurement unit names and abbreviations**

| [**UnitsID**](javascript:__doPostBack('dgCV_11$ctl01$ctl00','')) | [**UnitsName**](javascript:__doPostBack('dgCV_11$ctl01$ctl01','')) | [**UnitsType**](javascript:__doPostBack('dgCV_11$ctl01$ctl02','')) | **[UnitsAbbreviation](javascript:__doPostBack('dgCV_11$ctl01$ctl03',''))** |
| --- | --- | --- | --- |
| 7 | hectare | Area | ha |
| 8 | square meter | Area | m^2 |
| 9 | platinum cobalt units | Color | PCU |
| 10 | milligrams per liter | Concentration | mg/L |
| 11 | micrograms per liter | Concentration | ug/L |
| 12 | milligrams per cubic meter | Concentration | mg/m^3 |
| 13 | microequivalents per liter | Concentration | ueq/L |
| 14 | percent | Dimensionless | % |
| 15 | pH Unit | Dimensionless | pH |
| 16 | micromho | Electrical Conductivity | Umho |
| 17 | micromho per centimeter | Electrical Conductivity | Umho/cm |
| 18 | microsiemens per centimeter | Electrical Conductivity | uS/cm |
| 19 | feet | Length | ft |
| 20 | meter | Length | m |
| 21 | gram | Mass | g |
| 22 | kilogram | Mass | kg |
| 23 | milligram | Mass | mg |
| 24 | microgram | Mass | ug |
| 25 | degree Celsius | Temperature | degC |
| 26 | year month day | Time | yymmdd |
| 27 | nephelometric turbidity units | Turbidity | NTU |
| 28 | absorbance units per cm | Color | AU/cm |
| 29 | Micromoles per liter | Concentration | umol/L |
| 30 | Parts per million | Concentration | ppm |
| 31 | Parts per billion | Concentration | ppb |

***SampleType***

The method with which the measurements were taken or the water was sampled.

**Table S11. Sample type controlled vocabulary phrases**

| **Term** | **Definition** |
| --- | --- |
| GRAB | Sample taken from a single depth |
| INTEGRATED | Sample taken from multiple depths using a tube sampler that integrates the water column to a determined depth; or Secchi depth |
| PROBE | Samples taken from probe |
| UNKNOWN | The sample type is unknown |
| MULTIPLE | More than one method used |
| SPECIFIED | The sample type is specified in the data table |
| NULL | For lake variables that are not measured by field sampling, e.g., lake morphometric characteristics such as mean depth, max depth, elevation, and surface area |

**References**

1. Consortium of Universities for the Advancement of Hydrologic Science, Inc. CUAHSI ODM 2015. https://www.cuahsi.org/ODMControlledVocabulary. Accessed 2 December 2011.
