# Supplementary material for: Building a multi-scaled geospatial temporal ecology database from disparate data sources: fostering open science and data reuse
Source: Gigascience. 2015 Jul 1;4:28. doi: 10.1186/s13742-015-0067-4 (PMC4488039; doi:10.1186/s13742-015-0067-4)
Supplement: Additional file 6: — LAGOS database design. A description of the LAGOS database design, the origin and justification of the design, and the extensibility of the database. [file 13742_2015_67_MOESM6_ESM.docx]

Additional file 6

**LAGOS database design**

Ed Bissell, Corinna Gries, Pang-Ning Tan, Patricia Soranno, Sam Christel

**Overview**

Our research goal was to produce an integrated geospatial temporal database (LAGOS) that incorporated heterogeneous data of both lake chemistry and geospatial information on lakes at a sub-continental scale. Compiling LAGOS was challenging because the source datasets were so heterogeneous. As a result, our data did not meet the assumed levels of data standardization that are needed for many current approaches to automate data integration. Therefore, we used a more labor-intensive semi-automated approach for our database integration. For our database design, we chose the Consortium of Universities for the Advancement of Hydrologic Science, Inc. (CUAHSI) Operational Data Model (ODM) because it provides a flexible data model, a controlled vocabulary, and extensive metadata built directly into the database. In this document, we provide the rationale for selecting the CUAHSI ODM for LAGOS, most of which relates to addressing the challenges of integrating highly heterogeneous data into an integrated database and to meeting the broader research goals for our research project, as described in Additional file 2.

**Introduction**

Relational databases were traditionally designed for storing relatively homogeneous data. The key principles underlying the design of these relational databases are based on the theory of database normalization [1], which dictates how the schemas in a database should be organized to minimize duplicate information across multiple tables, to reduce wasted storage of null values, and to ensure that the dependencies among the data items are correctly manifested in the database. However, in addition to the fundamental practice of database normalization, LAGOS, like nearly all databases, also requires optimization. In addition to maintaining storage efficiency, the design must resolve a myriad of data integration challenges while remaining flexible enough to accommodate future database expansion (e.g., updates of old datasets with additional sampling years or addition of completely new datasets). These requirements have led to greater complexity in the design and implementation of LAGOS than is associated with homogenous databases of similar scope.

LAGOS required integration of almost 100 lake chemistry and 21 geospatial datasets that were collected from diverse sources, all of which used different formats for storing data. LAGOS is a relational database consisting of two modules, lake chemistry data (LAGOS_LIMNO_) and geospatial data (LAGOS_GEO_), which are linked to each other by a common identifier_._ Furthermore, each module is structured as a relational database in that the corresponding data tables are linked by common variables. We compiled both database modules because both classes of data are required to answer the questions posed by our macrosystems ecology research program. LAGOS does not support many simultaneous users; rather, it is a repository database from which custom exports that suit a particular researcher’s unique data requirements can be generated. Next, we describe the process of designing the LAGOS database. In particular, we describe the rationale, the challenges we encountered, and we also suggest best practices for research projects that wish to produce similar multi-scaled, multi-themed integrated databases. We begin by summarizing the variety of data formats included in LAGOS.

**A description of the data formats of the datasets that were integrated into LAGOS_LIMNO_**

The chemical limnology datasets that we acquired had a variety of different file formats, including text files (.txt, .csv), spreadsheets (.xls, .xlsx), documents (.doc, .docx, .pdf), and databases (.mdb, .accdb). The composition of the source datasets varied considerably. Some datasets were a single flat file containing all of the relevant information (e.g., nutrient samples, metadata, lake information), whereas other datasets had the same information in multiple source files of various formats. In many cases, these source files were not easily related to each (i.e., there were no linked variables) or the source datasets contained a considerable amount of unrelated information. In some cases no locational information was provided and instead we determined the lake locations manually based on lake names, communication with the data provider, or other information. Furthermore, some datasets included documentation in the form of descriptive columns in the source dataset or external metadata, whereas other datasets had included no documentation. In the latter case, we often had to communicate with the data provider again or do post-hoc research to determine what methods were employed in producing a given dataset.

A major challenge during our data integration efforts was that the fundamental approach to ‘data modeling’ (i.e., how data are conceptually described) varied considerably across datasets. For example, consider a standard approach to limnological investigations, which requires a lake to be sampled at the deepest location. Not all of the programs were organized this way. In fact, there was tremendous variability in sampling regimes. For example, some datasets contained data sampled from multiple locations on a lake, and included sample information about the basin (specific sample locations within the lake) that may or not be unique (and may or not have been in the lake’s deepest location). In contrast, most datasets simply reported samples from an arbitrary location on the lake, often in the ‘middle’ because of the common assumption that the middle is the deepest location. In addition, some datasets uniquely identified different lakes based on specific criteria (e.g., natural resource agency identification codes) while some did not. In summary, there was a large variation in how different programs designed their research programs, databases, and in how they documented the databases and the lake location in particular. The lack of individual database standards created considerable challenges for the development of strategies to automate data processing (i.e., manipulating the source data to match the format of LAGOS) and for the importing of the processed data into LAGOS. Thus, our approaches were only semi-automated and, therefore, they were very labor-intensive.

**A description of the data formats of the datasets that were integrated into LAGOS_GEO_**

For LAGOS_GEO_, we created our own datasets from each of several geospatial data layers. These datasets were individual tables that contained a wide range of geospatial metrics that we had calculated (i.e., derived data products). We used 21 geospatial datasets that are publicly accessible online from federal agencies and other research groups, including associated metadata. We compiled the most important metadata from each data source, including information such as the program that produced the data layer, the data type (e.g., raster, vector), and the temporal and spatial resolution of the data (see Table S1 in Additional file 5). Furthermore, we developed a GIS-toolbox to calculate a series of metrics from these data in order to define, classify, and characterize the landscape context of all of the lakes in our study area (see Additional file 8). Finally, because of the multi-scaled and hierarchal nature of the research questions for our project, we chose to summarize the geospatial datasets at a range of spatial extents (see Additional file 7).

**Database design for LAGOS_LIMNO_ and LAGOS_GEO_**

The overall goal in designing the LAGOS database was to maximize information content and flexibility at the expense of other database design considerations, such as storage efficiency and query performance. We chose to design LAGOS to maximize information content and flexibility because of the questions that drive our research program, the heterogeneous nature of the disparate source datasets that comprise LAGOS, and especially because of our long-term goals for the database (see Additional file 2). Importantly, LAGOS integrates much supporting information (i.e., metadata) directly within the database, although we also created formal machine readable metadata (EML) for each individual LAGOS_LIMNO_ source dataset. Storing metadata in a relational database is often best accomplished by leveraging a vertically oriented (long) database design. Long tables are most amenable to storing metadata because new descriptive information can be added to the database as new datasets are loaded without altering the underlying database schema. In general, horizontal database designs do not lend themselves to storing metadata because a separate column is required for each new variable being measured or observed (Figure S2A) and, therefore, variable-specific metadata also require a separate column.

A database design does not have to be exclusively horizontal or vertical. Therefore, depending on the different data types, the different levels of metadata to be integrated into the database, and other important criteria, an integrated database can include tables that are either horizontal or vertical. In fact, LAGOS includes both vertical and horizontal tables, which we describe below.

**
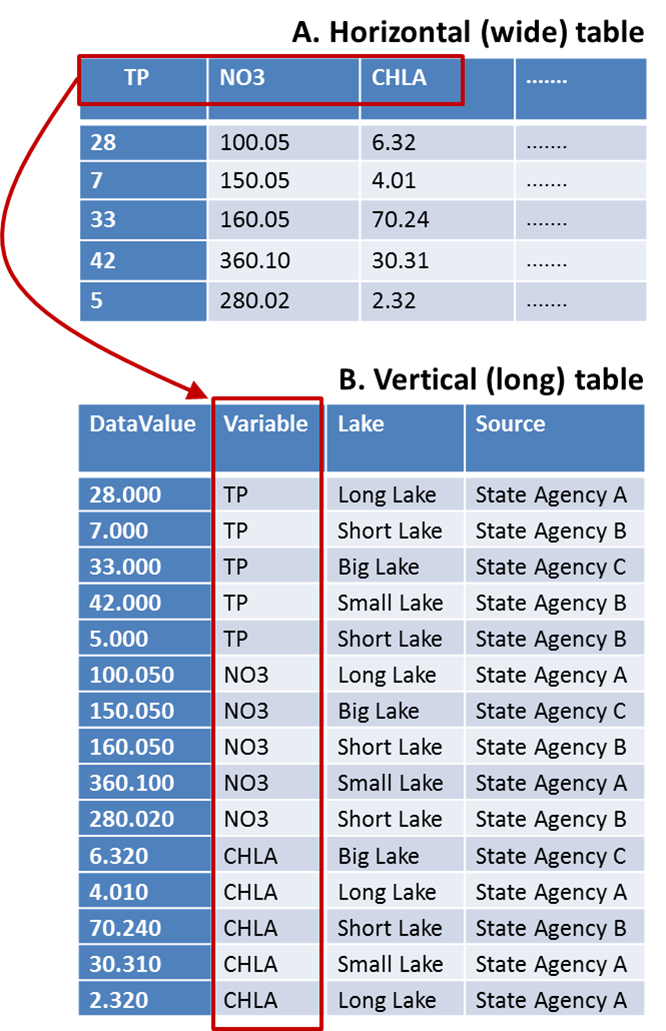
**

**Figure S2. An example schema of a horizontal database model (A), also called wide; and, an example schema of a vertical database model (B), also called long.** The variables contained in the columns of the wide database model are collapsed into a single column in the long database model.

**LAGOS_GEO_**

The major design difference between LAGOS_GEO_ and LAGOS_LIMNO_ is that LAGOS_GEO_ is almost exclusively horizontal in orientation, and LAGOS_LIMNO_ is almost exclusively vertical. LAGOS_GEO_ primarily consists of *datavalues* that are calculated at a series of spatial extents, such as: lake, county, state, IWS, EDU, HUC4, HUC8, and HUC12. These datavalues do not have accompanying metadata columns and, consequently, there would have been no gain in flexibility or data provenance for the geospatial datavalues being stored vertically. Additionally, LAGOS_GEO_, will predominately be used by researchers in its native horizontal orientation.

**LAGOS_LIMNO_**

We created LAGOS using PostgreSQL, which is an open source relational database management system. We selected an existing database design for LAGOS_LIMNO_ based on the Consortium of Universities for the Advancement of Hydrologic Science, Inc. (CUAHSI) Community Observations Data Model (ODM) because it is a flexible data model (i.e., allows the incorporation of both LAGOS_LIMNO_ and LAGOS_GEO_) that allows for the incorporation of controlled vocabulary and, importantly, allows for extensive documentation through a relational database structure of linked tables containing metadata [2]. The datavalues table in ODM, which stores the data observations, consists of a limited number of columns in which one column contains the actual data values, one column provides the variable name (e.g., TP or NO3) for each data value, and subsequent columns store information about the variable and its datavalue (Figure S2B). The vertical structure of the ODM datavalues table has greater data management flexibility than horizontally structured tables because of the limited number of columns and also because metadata can be directly incorporated into the table. Because a separate row is required for each variable being measured, the table becomes long (i.e., it has many rows) but remains narrow (i.e., it has few columns). As noted above, LAGOS_GEO_ contains wide tables (Figure S2A) and, consequently, LAGOS is a quasi-vertical database because only the tables in LAGOS_LIMNO_ follow the CUAHSI ODM.

An important feature of the database is that the column names and data stored in the table are assigned observations based on a controlled vocabulary [2]. A controlled vocabulary is a set of standardized key words used for column names and values of categorical variables in a database. Using a controlled vocabulary helps in the data integration process because it standardizes which elements are extracted from the source dataset and incorporated in the integrated dataset. Using a controlled vocabulary also simplifies statistical analyses of categorical data because the data categories are limited to a standardized list. It is important to note that consistency with the use of controlled vocabulary is essential to good data management of databases; therefore, in compiling LAGOS, we ensured absolute consistency with the use of controlled vocabulary tables (see Additional file 4). Please refer to the section “Exploration of existing database designs – CUAHSI-ODM” below for further information on the CUAHSI ODM.

As an alternative to the vertically structured datavalues table of LAGOS_LIMNO_, we considered the short and wide relational database model (Figure S2B), which is a ubiquitous database model for many ecological studies and was the format that we used for LAGOS_GEO_. This database model is characterized by tables designed such that each variable occupies a column and also that contain a fewer number of columns with metadata (Figure S2B). The short and wide database model is less flexible to manage because there are a large number of columns and new information stored in the database will require the addition of new columns. As discussed above, the vertically structured database model is much more flexible for managing highly heterogeneous data and for incorporating new data in the future, which was an important goal of our research effort.

*Data provenance:* Ensuring that there is a plan for data provenance is critical to designing a database for synthetic research projects that aim to produce integrated databases. Data provenance is a record that details: how a dataset was produced, all of the changes that were made to a dataset, and any other details required to analyze a dataset. In many of the datasets that we received, there was either no documentation or it was scattered in independent word processor files. Compiling and meticulously managing metadata for a dataset is central to ensuring adequate data provenance. We also discovered that many programs switched dataset formats over time, they also shifted the methods of documentation (e.g., data flags). This situation is an example of inadequate data provenance and the data manipulation (See Additional file 19) that is required to produce an integrated database is consequently much more extensive. It is possible and likely that datasets produced from long-term research projects will require alterations to the database design or metadata because of changes in the research questions and technology. If changes to the database design are required, then data provenance can be achieved by ensuring that all of the changes are adequately addressed in either the documentation or the metadata.

*Challenges with sample position:* LAGOS_LIMNO_ maximizes information content by often storing multiple descriptive columns that encompass how individual data sources approached data modeling of lake chemistry data. One of the biggest challenges in documenting samples collected in lakes is to properly assign the depth that the sample was taken, particularly because there is no standard way to document this important piece of information. Therefore, LAGOS_LIMNO_ contains two important variables associated with each datavalue: *sampleposition* (a descriptive location of where a sample was taken within the water column, such as the epilimnion or hypolimnion) and *sampledepth* (numeric depth in meters below the water surface from which a sample was taken) (see Additional file 4 for more information on these two variables). One approach would have been to rely solely on *sampleposition* as the column to define where the samples were measured within the water column. However, this approach is problematic because some source datasets included information regarding *sampledepth* but not *sampleposition* while in other (considerably rarer) cases both attributes were included. Standardizing solely on *sampleposition* would have considerably decreased the information content in LAGOS_LIMNO_ by arbitrarily aggregating *sampledepths* into *samplepositions* or excluding records entirely from LAGOS_LIMNO_ for which a reliable *sampleposition* could not be accurately determined.

**Exploration of existing database designs – CUAHSI-ODM**

The LAGOS database design was based largely on the Observations Data Model (ODM) from the Consortium of Universities for the Advancement of Hydrologic Science, Inc. (CUAHSI). ODM is a relational database model designed to serve as a “*standard format to aid in the effective sharing of information between investigators and to allow analysis of information from disparate sources both within a single study area or hydrologic observatory and across hydrologic observatories and regions*” [2]. The ODM design facilitates storing descriptive information (metadata) directly in the database which allows users to trace the lineage of data values back to their source datasets. This is critical when working with composite datasets produced from integrating multiple source datasets.

Another advantage of the ODM is that although it was designed for a slightly different subject matter (hydrology), many of the fundamental aspects of how data are modeled for hydrology and chemical limnology are identical or similar. Hence, many of the controlled vocabulary tables from the CUAHSI ODM could be used in LAGOS_LIMNO_ with only minor modifications. In addition, the basic ODM table structures and relationships were largely retained in our design of LAGOS_LIMNO,_ although we also made some significant changes, mostly in the form of simplifying the design because the information content in the source datasets was not thorough enough to populate many of the ancillary tables that are in the CUAHSI ODM. As mentioned above, the principle design requirements of LAGOS_LIMNO_ included flexibility in data storage, standardization of methods that allow for integration of multiple disparate datasets, and also a format that maximizes information content. Thus, we based the LAGOS_LIMNO_ schema on CUAHSI ODM because it accommodates these design requirements.

**Long term vision: updating and adding to LAGOS**

The design of LAGOS_LIMNO_ facilitates the addition of new data, updates of old datasets with data for additional sampling years, or completely new datasets with minimal changes to the underlying database schema. Many of the research and monitoring programs from which we obtained data continue to collect lake chemistry data. The design of LAGOS (e.g., standardized format) and ample data provenance (e.g., SQL and R data manipulation scripts) produced in this data integration effort greatly facilitate updating the database and using it to ask new questions related to lakes across broad geographic extents. Currently, LAGOS is an unprecedented database in terms of sample size, documentation, and spatial extent. Future additions to LAGOS will add more value to the database by facilitating additional research questions that were not part of the original research goals, and in so doing they will leverage the cost and investment to create it in the first place. LAGOS_LIMNO_ also stores considerably more data than is exported in a single database export. For example, to date, our research questions have focused only on surface samples for lake chemistry; however, the database includes chemistry data from more depths, which can be analyzed in future efforts.

**References**

1. Codd EF: **A relational model of data for large shared data banks**. *Communications of the ACM* 1970, **13**: 377-387.

2. Tarboton, D.G., Horsburgh, J.S., and D.R. Maidment (2008): **CUAHSI community observations data model (ODM) design specifications document: Version 1.1**. http://his.cuahsi.org/odmdatabases.html
