# Supplementary material for: Building a multi-scaled geospatial temporal ecology database from disparate data sources: fostering open science and data reuse
Source: Gigascience. 2015 Jul 1;4:28. doi: 10.1186/s13742-015-0067-4 (PMC4488039; doi:10.1186/s13742-015-0067-4)
Supplement: Additional file 7: — The spatial extents for LAGOSGEO. The spatial extents for which we calculated all GEO features that were delineated by either political, topographical, or hydrologic boundaries. [file 13742_2015_67_MOESM7_ESM.docx]

# Additional file 7

# **The spatial extents for LAGOS_GEO_**

Patricia Soranno, Nicole Smith*,* Scott Stopyak, Ed Bissell

**Overview**

In developing LAGOS_GEO_, we identified a set of spatial extents, some of which are nested, in which we characterized the geospatial features around all lakes ≥ 4 ha that are in the 17-state study area. For many features, we calculated estimates of all spatial extents; however, for other GEO features, it made sense to only calculate values for a subset of spatial extents (see Additional file 13). Here, we first provide a description of the spatial extents in LAGOS_GEO_ and document the modifications we made to the source data prior to geoprocessing. We then explain the decisions made regarding selection of the appropriate spatial extent(s) used to calculate an individual feature with respect to the horizontal spatial resolution of the feature’s original dataset, the spatial extent of the natural variation of the feature, and the ecological justification for the calculation of the feature at that scale. In future research efforts, decisions about how to handle boundary definitions of different spatial extents should be considered early in the process to avoid problems.

**Description of spatial extents**

There are three main categories of spatial extents for which we calculate the GEO features of LAGOS. Brief descriptions of the spatial extents and data sources are explained below.

Political boundaries

- **State** - boundaries of the 17 US states (Figure S3)

*Source:* The Tiger (US Census Bureau) states database that includes high-resolution boundaries of the 17 US states, released in 2013. The data were downloaded from the Tiger database [1].


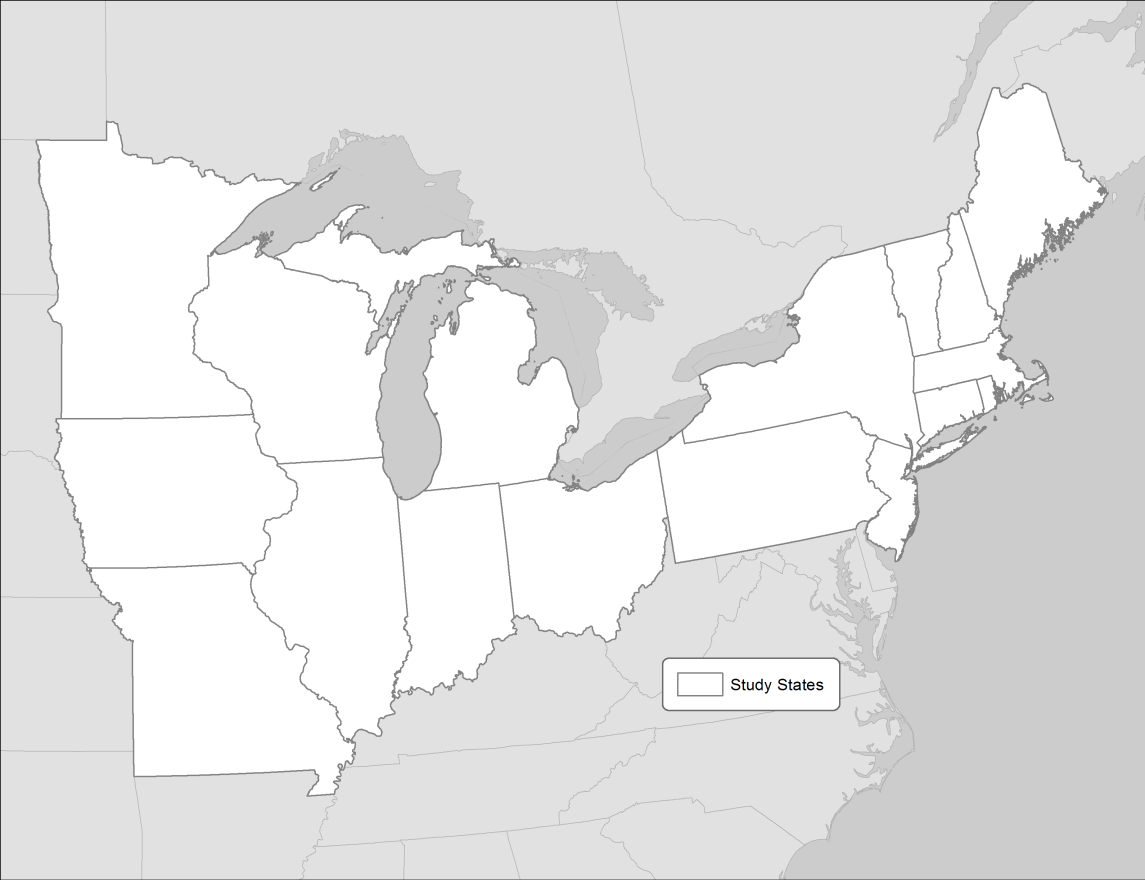


**Figure S3. Geographical distribution of the 17 states included in LAGOS.**

- **County** - boundaries of counties in the 17 states (Figure S4)

*Source:* The 1:100,000-scale Counties of the US, USGS, released July 2012. We were not able to use the above Tiger data source for county boundaries because county lines extended into the center of the Great Lakes. This USGS dataset uses the Great Lakes shoreline as the county line [2].


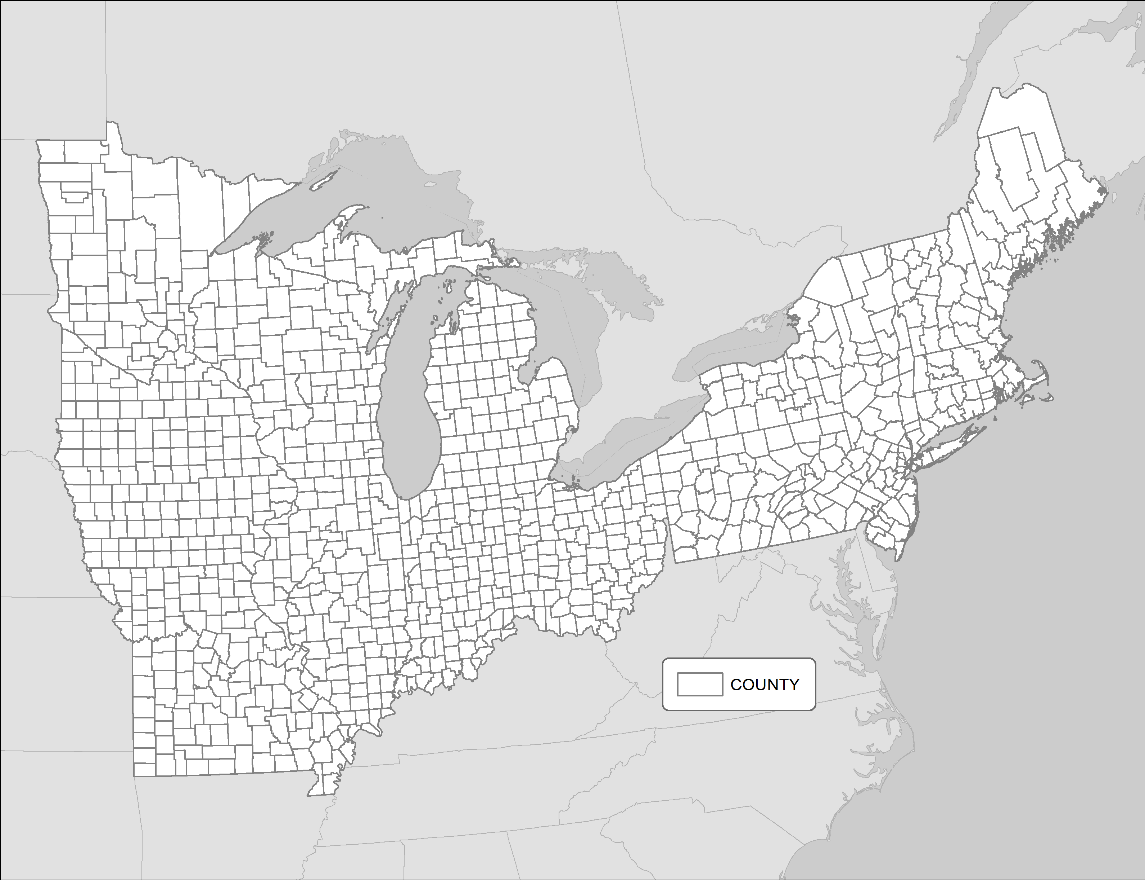


**Figure S4.** **Geographical distribution of the counties distributed across the 17 states included in LAGOS.**

Hydrologic unit spatial extents

*Source:* The Watershed Boundary Dataset (WBD, USDA Natural Resources Conservation Service), downloaded June 2013. The WBD defines boundaries based on surface water drainage and creates nested Hydrologic Units (sometimes abbreviated and referred to as HUCs, which are hydrologic unit codes) that are georeferenced to the USGS 1:24,000 topographic base map. See Appendix 1 for additional information on the WBD that is relevant to the LAGOS database. The data were downloaded from the WBD index of the USDA Natural Resources Conservation Service website [3].

- **HUC4** - The second level of classification in the WBD dataset, called a subregion (Figure S5). A subregion includes the area drained by a river system, a reach of a river and its tributaries in that reach, a closed basin(s), or a group of streams forming a coastal drainage area.

**
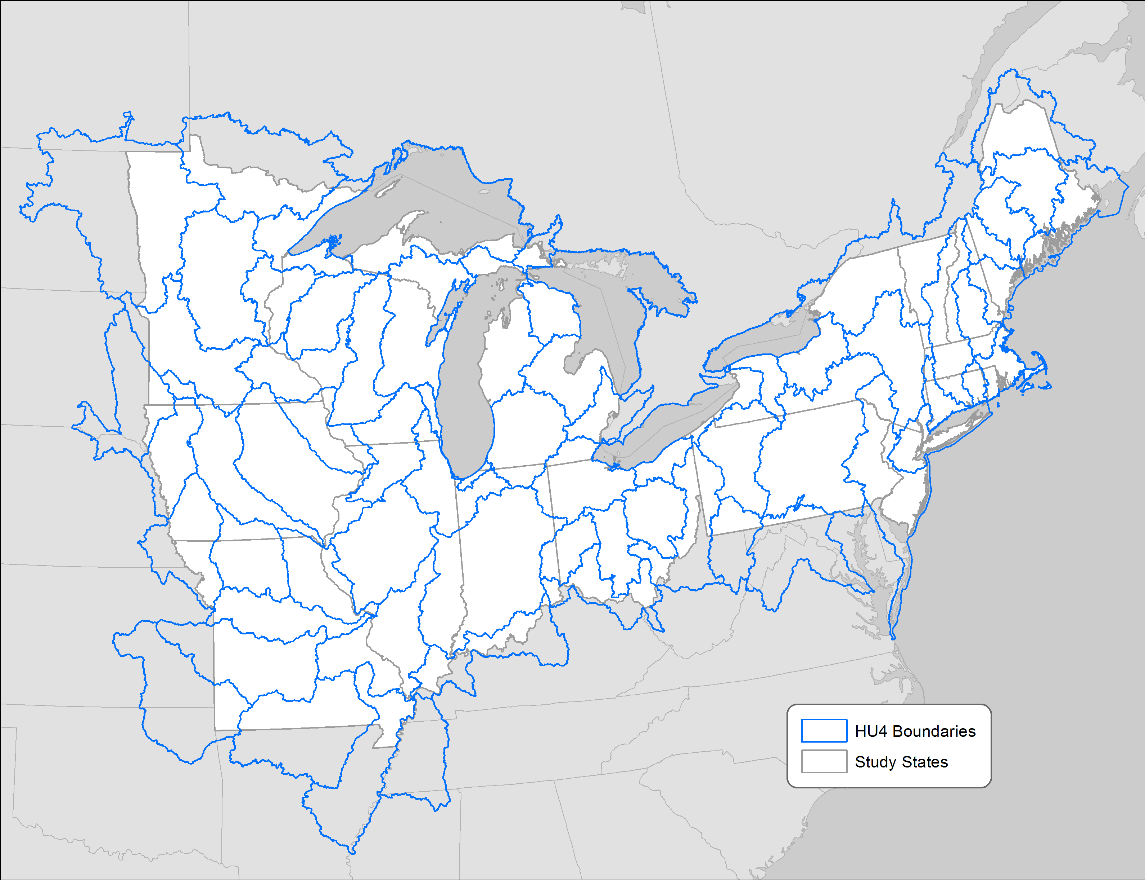
**

**Figure S5. Geographical distribution of the HUC4 boundaries (referred to as HU4 in the figure.**

- **HUC6 -** The third level of classification subdivides many of the subregions into accounting units which are nested within, or can be equivalent to, the subregions. We do not recommend the use of HUC6s because sometimes they are equivalent in size to HUC4s, and sometimes not, hence they seem less consistent than HUC4s.
- **HUC8** - The fourth level of classification is the cataloging unit, which subdivides the subregions and accounting units into smaller areas, and is sometimes called a 'watershed' (Figure S6). A HUC8 is a geographic area representing part or all of a surface drainage basin, a combination of drainage basins, or a distinct hydrologic feature.


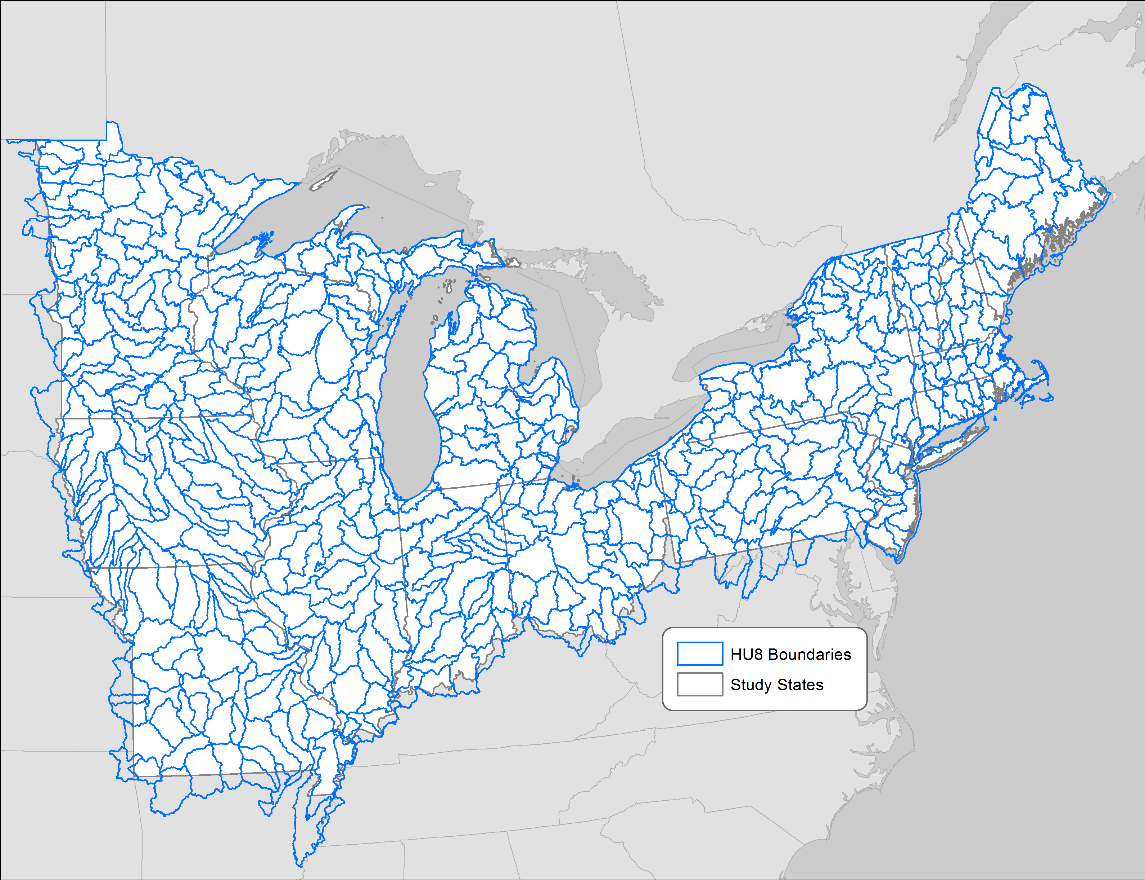


**Figure S6**. **Geographical distribution of the HUC8 boundaries (referred to as HU8 in the figure).**

- **HUC12** - The sixth level of classification is the HUC12, which subdivides the HUC8s into smaller areas (Figure S7; note that HUC10s are also calculated in the WBD, but not used in LAGOS). HUC10s and HUC12s are defined along natural hydrologic breaks based on land surface and surface-water flow and they have a single flow outlet except in frontal, lake, braided-stream, or closed-basin hydrologic units.

**
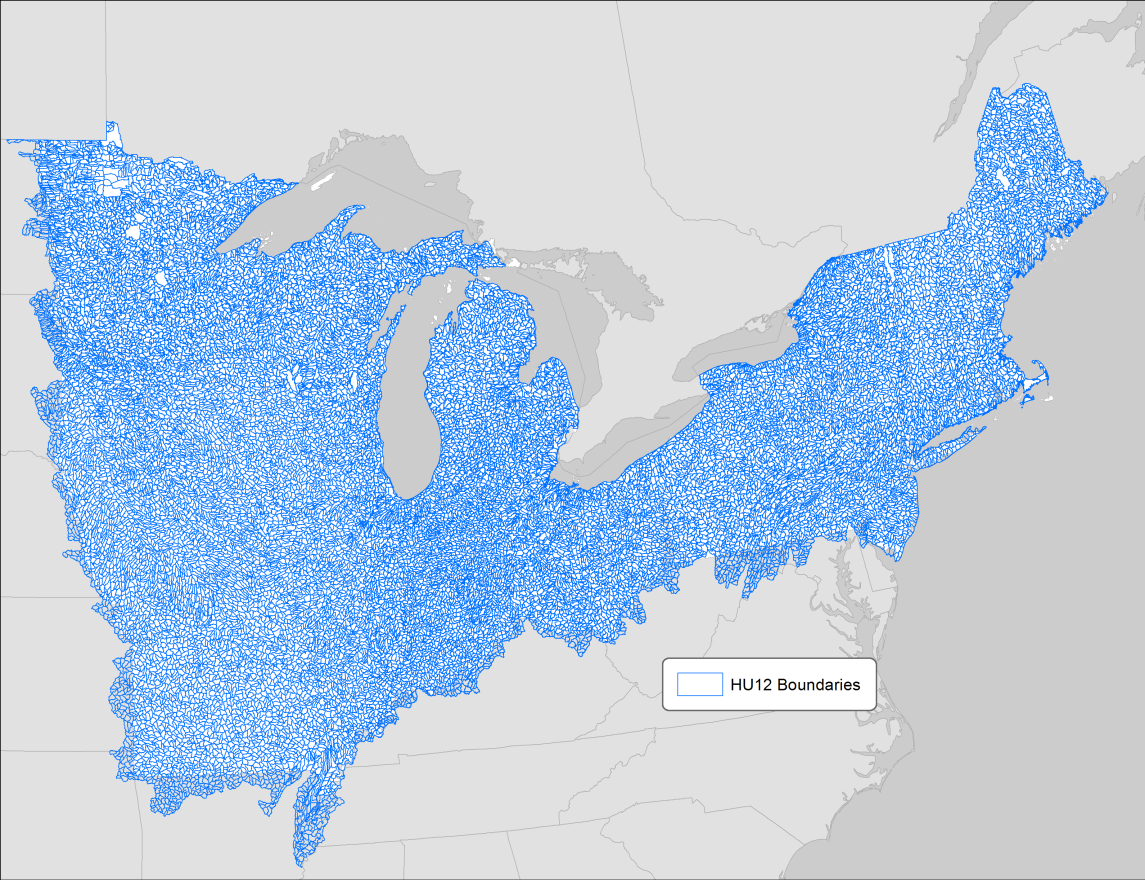
**

**Figure S7. Geographical distribution of the HUC12 boundaries (referred to as HU12 in the figure)**

- **EDU** - Ecological Drainage Units ([4], Figure S8) follow the WBD boundaries, and are of roughly similar size to HUC6s. EDUs were delineated by grouping the HUC8 watersheds based on common zoogeographic history, and physiographic and climatic characteristics. For our 17-state study area, some areas did not have complete coverage, and so required manual interpretation by our team. We obtained the data from a National Fish Habitat Action Plan dataset. We created a coverage of EDUs for our study area by combining 24K NHD HUC8 geometry with EDUs that were originally derived from NHD 250K datasets. The conflation was achieved by rasterizing the EDUs and transferring the values to the higher-resolution HUC8s via zonal statistics operations that assigned an EDU to an HUC8. Linkage was based on the majority of EDU raster cells that fell inside each of the HUC8 polygons. [*Note, we recommend for analyses with LAGOS to use HUC4 as the 'regionalization' rather than EDU, because the EDU coverage is not easily available at the national level and because HUC4s have more standard and commonly used delineations.]*


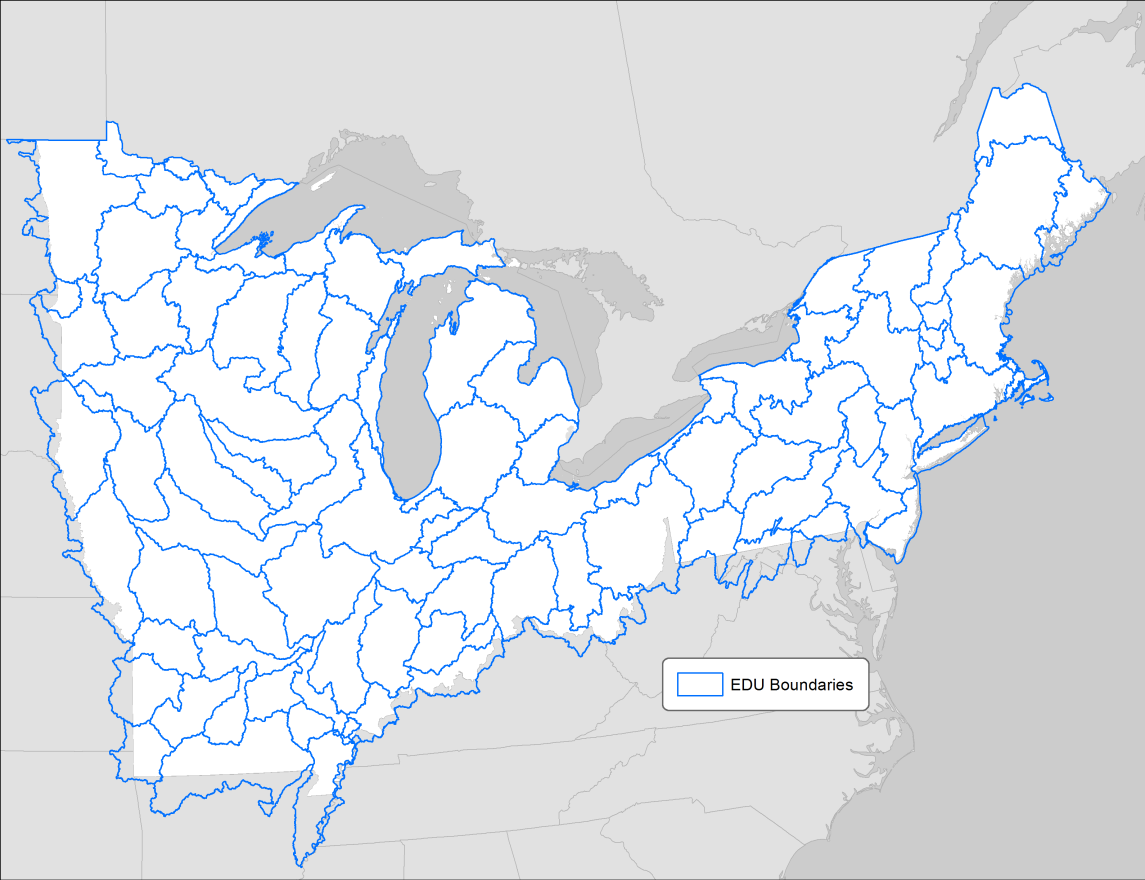


**Figure S8. Geographical distribution of the Ecological Drainage Units boundaries.**

Zones created around lakes (LAGOS generated)

- **IWS (Interlake watershed)** - We delineated lake watersheds for all lakes ≥ 4 ha in LAGOS. We defined lake watersheds in LAGOS as the following: The area of land that drains directly into a lake, and into all upstream-connected, permanent streams to that lake exclusive of any upstream lake watersheds for lakes ≥ 10 ha that are connected via permanent streams. See the LAGOS watershed delineation documentation in the LAGSO GIS Toolbox (Additional file 8) for further details.
- **500 m lake buffer** - for all lakes ≥ 4 ha, we generated a polygon feature that is a 500 m equidistant buffer of the land that is 500 m from the lake shoreline using the ArcGIS Buffer tool. We created this buffer primarily to calculate land use/cover around a lake because this buffer distance has been used by previous studies to quantify land use/cover around lakes.
- **100 m lake buffer** - for all lakes ≥ 4 ha, we generated a polygon feature that is a 100 m equidistant buffer of the land that is 100 m from the lake shoreline using the ArcGIS Buffer tool. We created this buffer primarily to calculate land use/cover in the riparian zone around a lake, which is sometimes defined as land within 100 m of the shoreline.

**Description of source data manipulations**

Watershed Boundary Dataset manipulations for LAGOS

We downloaded the version of the NRCS Water Boundary Dataset issued on June 2013. We first used the HUC8 layer to create a master study boundary, and then we clipped the HUC12, HUC6, and HUC4 feature classes to this master study boundary (all the HUC feature classes share the same extent). We selected only the HUC8s we needed using this chain of steps:

1. Clip the features to the United States boundaries. HUC8s that were entirely in Canada were removed entirely, while those that were at least partly in the US were kept, but their geometry was modified to be clipped at the border. This clip operation was performed using the US_Study_Bounds polygon in CSI_Master_Geodatabase_2014_*.gdb.
2. Select HUC8s with ≥ 10% of their area within the 17 study states to retain in the feature class. This was accomplished by first using Tabulate Intersection to identify the percentage of a HUC8 falling within the state boundaries above. HUCs that were clipped at the border in step 1 were considered 100% within the US for this step and so none of these HUCs were removed in this step-the order of the steps is important if this process is repeated.
3. Drop the HUC8s that correspond to the Long Island Sound, the Delaware Bay, and the five Great Lakes.

The final HUC8 extent/boundary follows the US/Canada border and the Great Lakes shoreline in the north, the Atlantic coastline to the east, and slightly exceeds the extent of the 17-state region on the south and the western edges (Figure S9).


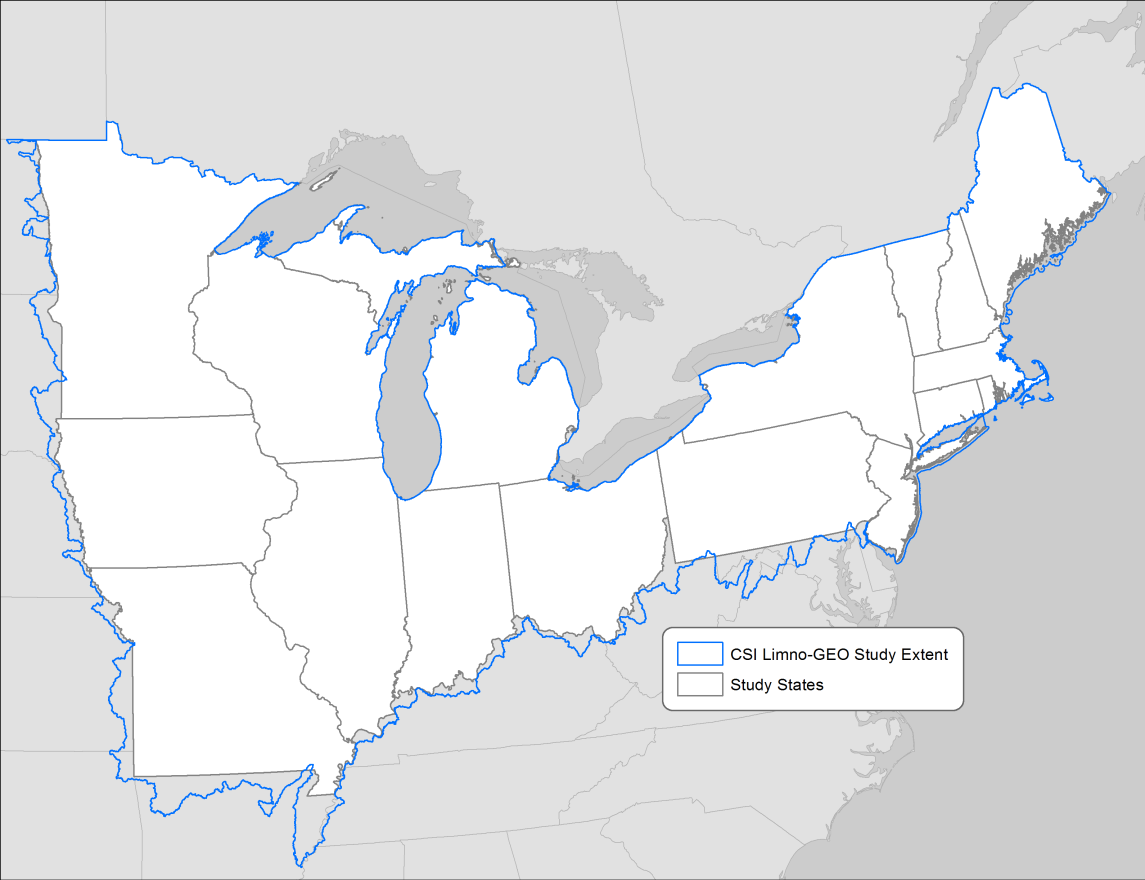


**Figure S9. Overall extent of the LAGOS_GEO_ database.**

The HUC8 boundary was then used to clip the HUC4, HUC6, and HUC12 layers. For the HUC4 and HUC6 layers, the clipping resulted in reducing the size of some HUC features (Figure S10).


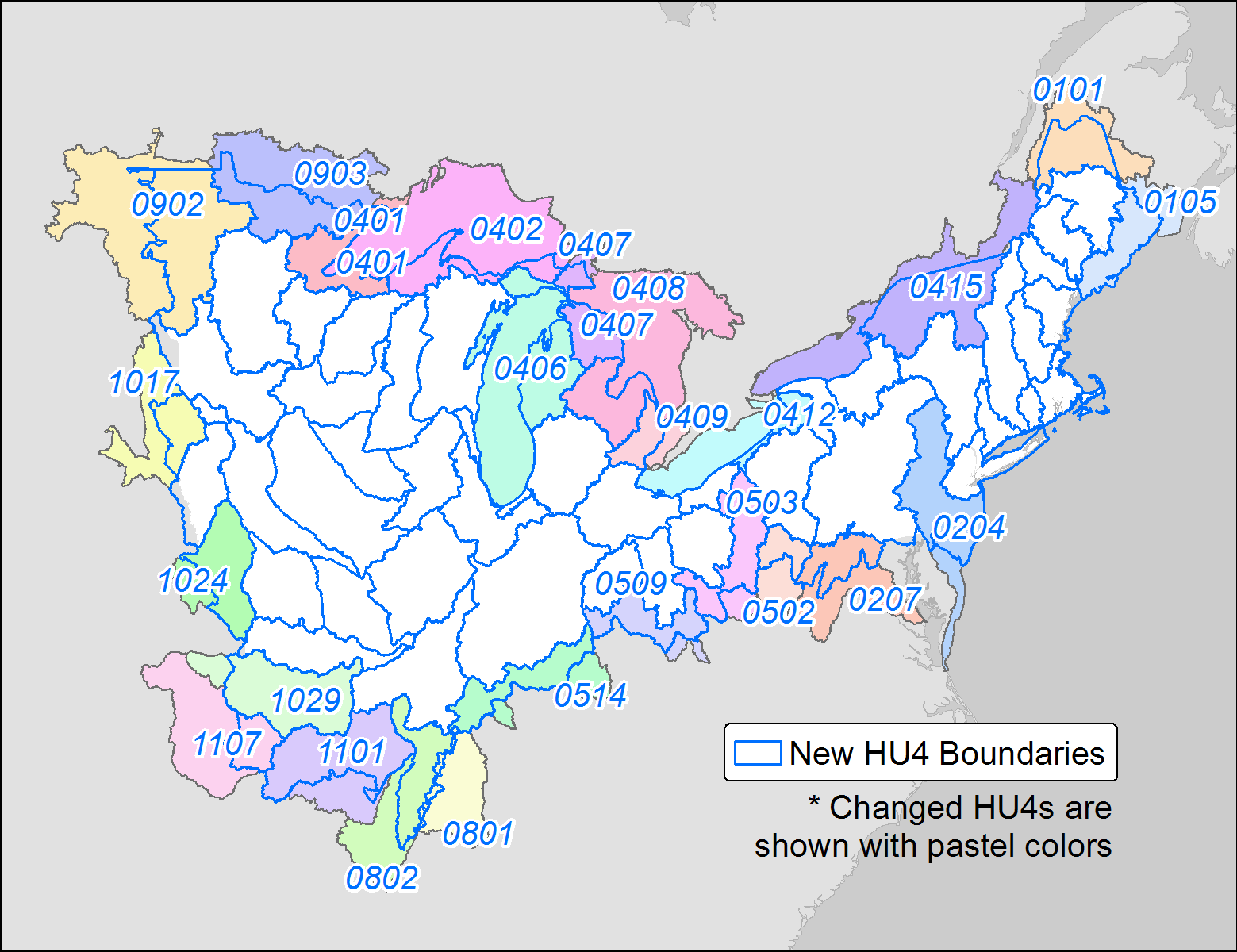


**Figure S10. Illustration of how some HUC4 extents (referred to as HU4 in the figure) were clipped to fit LAGOS_GEO_ extent shown in Figure S9.**

Some HUC12s dominated by ocean, the Great Lakes, or coastal island features were also removed using best professional judgment because the spatial extent of most of the data sources used to characterize these regions does not extend to these areas (Figure S11).


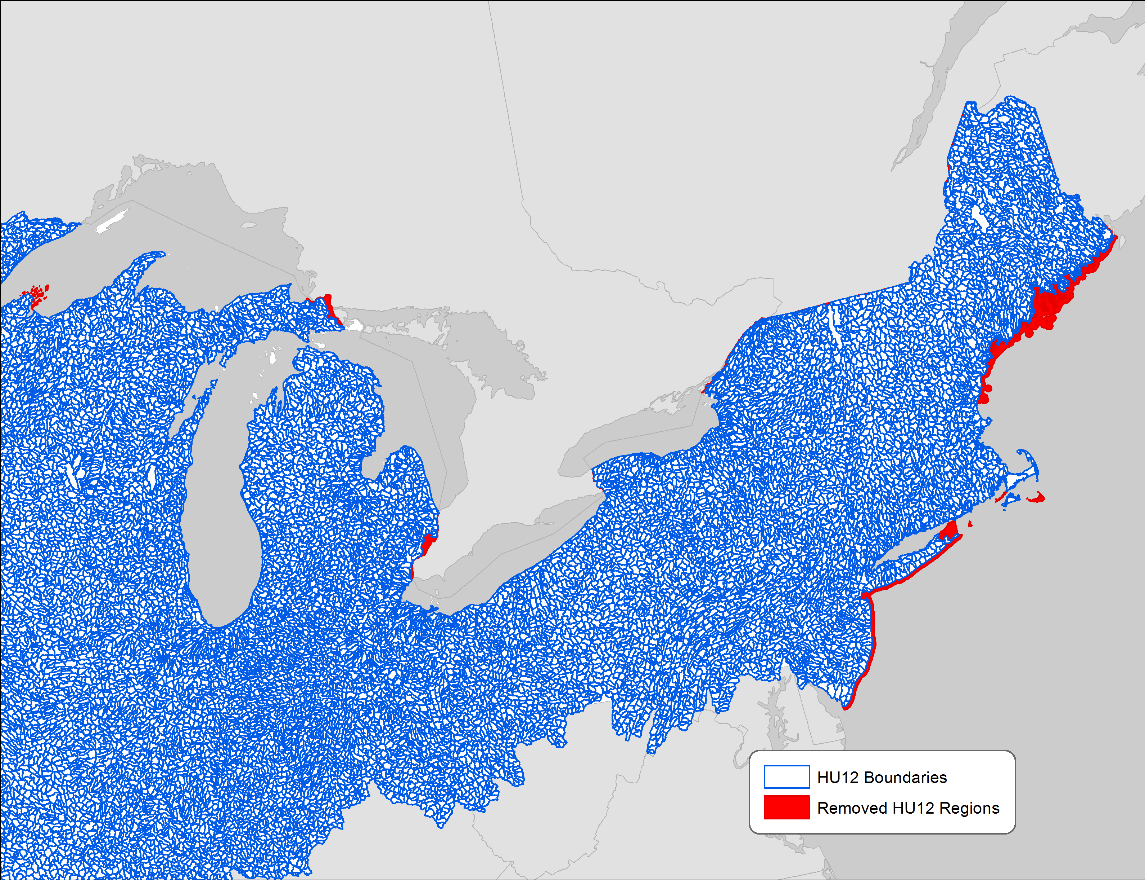


**Figure S11. Illustration of how some HUC12s (referred to as HU12 in the figure) were removed because their boundaries extended into the Great Lakes or into the ocean.**

For each of the HU feature classes, we also verified that none of the polygons overlapped each other, and that each had a unique HU code. We added a 'Zone_ID' field to uniquely identify each zone.

For a description of the numbers of HUCs at each level within the entire US and within the LAGOS study extent, see Table S15.

**Table S15. Number of HUCs at each level within the entire US and within the 17-state LAGOS study extent.**

| **Name** | **Level** | **Digit** | **Number of HUCs** | **Number of HUCs in 17-state LAGOS** |
| --- | --- | --- | --- | --- |
| Region | 1 | 2 | 21 | -- |
| **Subregion** | **2** | **4** | **222** | **65** |
| **Basin** | **3** | **6** | **352** | **--** |
| **Subbasin** | **4** | **8** | **2,149** | **511** |
| Watershed | 5 | 10 | 22,000 | -- |
| **Subwatershed** | **6** | **12** | **160,000** | **20,257** |

Bold indicates that the spatial extent is included in LAGOS.

**References**

1. Tiger. US Census Bureau, Washington, DC. 2013. ftp://ftp2.census.gov/geo/tiger/TIGER2013/STATE/.

2. 1:100,000-scale Counties of the United States. United States Geological Survey, Reston, Virginia. 2012. http://water.usgs.gov/GIS/metadata/usgswrd/XML/county100.xml.

3. The Watershed Boundary Dataset (WBD). USDA Natural Resources Conservation Service, Washington, DC. 2013. ftp://ftp.ftw.nrcs.usda.gov/wbd/WBD_Annual_NRCS_OfficalSnapshot_ForTheCurrentFiscalYear/.

4. Higgins JV, Bryer MT, Khoury ML, Fitzhugh TW: **A freshwater classification approach for biodiversity conservation planning**. *Conserv Biol* 2005, **19**:432–445.

**Appendix 1**

# **Watershed boundary dataset (wbd)**

***The information below was taken directly from the website and is relevant for using the WBD in LAGOS:*** <http://www.nrcs.usda.gov/wps/portal/nrcs/detail/national/water/watersheds/dataset/?cid=nrcs143_021617>

What is WBD?

Watershed boundaries define the aerial extent of surface-water drainage to a point. The intent of defining hydrologic units (HU) for the Watershed Boundary Dataset is to establish a baseline drainage boundary framework, accounting for all land and surface areas. The selection and delineation of hydrologic boundaries are determined solely upon science-based hydrologic principles, not favoring any administrative or special projects nor particular program or agency. At a minimum, they are being delineated and georeferenced to the USGS 1:24,000-scale topographic base map meeting National Map Accuracy Standards (NMAS). A hydrologic unit has a single flow outlet except in coastal or lakefront areas. As stated by the Federal Standard for Delineation of Hydrologic Unit Boundaries,

*"A hydrologic unit is a drainage area delineated to nest in a multi-level, hierarchical drainage system. Its boundaries are defined by hydrographic and topographic criteria that delineate an area of land upstream from a specific point on a river, stream or similar surface waters. A hydrologic unit can accept surface water directly from upstream drainage areas, and indirectly from associated surface areas such as remnant, non-contributing, and diversions to form a drainage area with single or multiple outlet points. Hydrologic units are only synonymous with classic watersheds when their boundaries include all the source area contributing surface water to a single defined outlet point."*

The Watershed Boundary Dataset is being developed under the leadership of the [Subcommittee on Spatial Water Data](http://acwi.gov/spatial/), which is part of the [Advisory Committee on Water Information](http://water.usgs.gov/wicp/) (ACWI) and the [Federal Geographic Data Committee](http://www.fgdc.gov/) (FGDC). The USDA Natural Resources Conservation Service (NRCS), along with many other federal agencies and national associations, have representatives on the Subcommittee on Spatial Water Data.

***The information below was taken directly from the website:*** <http://www.nrcs.usda.gov/wps/portal/nrcs/detail/national/water/watersheds/dataset/?cid=nrcs143_021616>

History of Hydrologic Units

Hydrologic unit boundaries define the aerial extent of surface-water drainage to a point. Hydrologic units through four levels were created in the 1970s and have been used extensively throughout the United States. During that time, the US Geological Survey (USGS) developed a hierarchical hydrologic unit code (HUC) for the United States. This system divides the country into 21 Regions, 222 Subregions, 352 Accounting Units, and 2,149 Cataloging Units based on surface hydrologic features. The smallest USGS unit (8-digit HUC) is approximately 448,000 acres. During the late 1970s the Natural Resources Conservation Service (NRCS), formerly the Soil Conservation Service, initiated a national program to further subdivide HUCs into smaller watersheds for water resources planning. A 3-digit extension was added to the 8-digit identification. By the early 1980s this 11-digit HUC mapping was completed for most of the US

During the 1980s several NRCS state offices starting mapping watersheds into subwatersheds by adding 2 or 3 digits to the 11-digit HUC. By the late 1980s and early 1990s the advent of GIS made the mapping of digital HUC boundaries feasible, and in the early 1990s the Natural Resources Conservation Service started to delineate hydrologic units to the fifth and sixth level by using GIS to meet 1:24,000 National Map Accuracy Standards. Subsequently, the NRCS decided to delineate and map the entire US to the 11- and 14-digit level. With increased interest from other federal, state, and local entities, this initiative became an interagency effort.

The goal of this initiative is to provide a hydrologically correct, seamless, and consistent national GIS database at a scale of 1:24,000, which has been extensively reviewed and matches to a minimum the USGS topographical 7.5-minute quads. The new levels are called watershed (fifth level, 10 digit) and subwatershed (sixth level, 12 digit). The watershed level is typically 40,000 to 250,000 acres, and the subwatershed level is typically 10,000 to 40,000 acres with some as small as 3,000 acres. An estimated 22,000 watersheds and 160,000 subwatersheds will be mapped to the fifth and sixth level. The GIS coverages are publicly available via the Internet. The database will assist in planning and describing water use and related land use activities.

The mapping is done by the use of GIS, incorporating DEMs, DRGs, and a variety of geospatial data and techniques. A national standard, first called NI-170-304, which is now superseded by the National Interagency Guidelines, established procedures and specifications for delineating and mapping hydrologic units. These guidelines help ensure that HUC boundaries are accurate and consistent nationwide and that the digital database is and will be usable with other natural resource, digital data layers in a GIS. The first national standard, NI-170-304, was issued in 1992 and was updated until it was superseded by the interagency guideline, described below.

Over the last ten years, many federal and state agencies have realized current 8-digit hydrologic unit maps are unsatisfactory for many purposes because of inadequate bases or scales. Thus, the NRCS worked with other federal and state agencies and with the Subcommittee on Spatial Water Data [Federal Geographic Data Committee](http://www.fgdc.gov/) (FGDC) to establish a federal interagency standard covering mapping and delineation of hydrologic units that would be suitable for all agencies. In cooperation with the FGDC and the [Advisory Committee on Water Information](http://water.usgs.gov/wicp/acwi/spatial/) (ACWI), a new interagency guideline was written. During December of 2002, this document was presented to the FGDC for review. This document has superseded NI-170-304 as the official standard for delineation of fifth- and sixth-level hydrologic units.

With the interagency standard, some changes have been made to the criteria for delineation and attribution of the fifth and the sixth level. These changes include coding the fifth level as 10 digit (formerly 11 digit in NI-170-304) and the sixth level as 12 digit (was 14 digit in NI-170-304). Another change is that the third level will officially be called 'basins' (formerly known as 'cataloging units') and the fourth level will be called 'subbasins' (formerly known as 'accounting units'). Additional attribute fields have also been added to the dataset. Over the last several years, a series of workshops have been held to promote this interagency effort and to resolve delineation and attribution issues.

This effort to delineate and digitize the HUCs is coordinated by federal, state, and local agencies, as well as universities and others interested in the effort. The NCGC provides coordination, verification, and certification of state datasets and integrates the state coverages into a national Watershed Boundary Dataset.
