# Supplementary material for: Building a multi-scaled geospatial temporal ecology database from disparate data sources: fostering open science and data reuse
Source: Gigascience. 2015 Jul 1;4:28. doi: 10.1186/s13742-015-0067-4 (PMC4488039; doi:10.1186/s13742-015-0067-4)
Supplement: Additional file 8: — LAGOS GIS Toolbox documentation. This file is the documentation for the LAGOS GIS Toolbox that is currently available in an online repository. [file 13742_2015_67_MOESM8_ESM.docx]

Additional file 8

**LAGOS GIS Toolbox documentation**

*Nicole J. Smith, Patricia A. Soranno, Scott Stopyak*


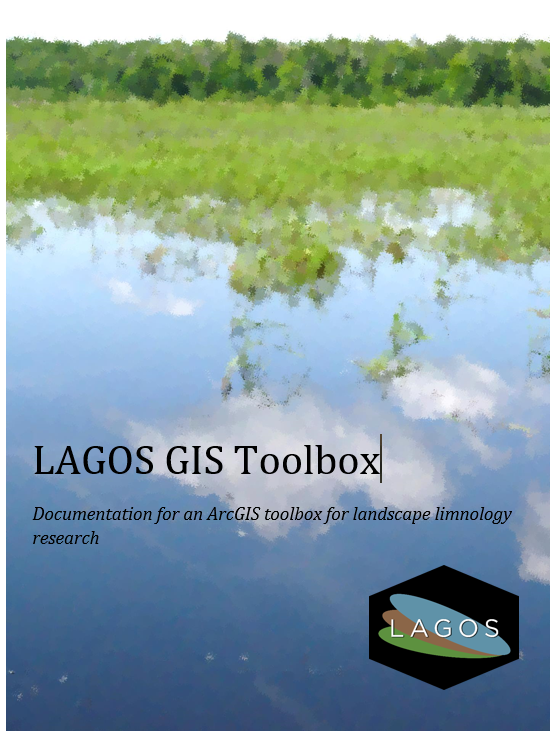


Prepared by:

Nicole J. Smith, Patricia A. Soranno, Scott Stopyak

December 31, 2014

Funding provided by:

**National Science Foundation**, Emerging Frontiers Division

Macrosystems Biology Program Grants (EF-1065786, EF-1065649, EF-1065818)

*The Effect of Cross-Scale Interactions on Freshwater Ecosystem State*

*Across Space and Time (CSI-Limnology)*

**PIs:** PA Soranno, KS Cheruvelil, P-N Tan, EH Stanley, NR Lottig, and JA Downing

Project period 2011-2016

http://csi-limnology.org


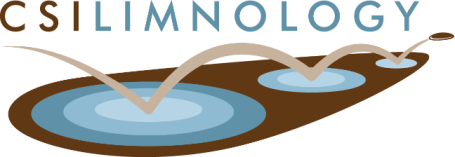


**Citing this document and code:**

All of the **code** for these methods is available on Github and should be cited as:

Smith NJ, PA Soranno, and S Stopyak. 2014. LAGOS GIS Toolbox. GitHub repository, https://soranno.github.io/LAGOS_GIS_Toolbox/

Please note that this document is provided for reference only as an example of a real document that is on GitHub, and as such the numbering of the figures and tables do not follow those used in the other additional files and links are included in the text rather than in the reference list.

**This document**, which describes the above code and methods, should be cited as a document within the following published article:

Patricia A. Soranno, Edward G. Bissell, Kendra S. Cheruvelil, Samuel T. Christel, Sarah M. Collins, C. Emi Fergus, Christopher T. Filstrup, Jean-Francois Lapierre, Noah R. Lottig, Samantha K. Oliver, Caren E. Scott, Nicole J. Smith, Scott Stopyak, Shuai Yuan, Mary Tate Bremigan, John A. Downing, Corinna Gries, Emily N. Henry, Nick K. Skaff, Emily H. Stanley, Craig A. Stow, Pang-Ning Tan, Tyler Wagner, Katherine E. Webster. *In Press*. Building a multi-scaled geospatial temporal ecology database from disparate data sources: Fostering open science and data reuse. GigaScience xx-xx.

**Table of Contents**

[Overview 5](#_Toc421200308)

[Testing the tools 5](#_Toc421200309)

[Hardware and Software 6](#_Toc421200310)

[Using the HPCC (for MSU Users) 6](#_Toc421200311)

[Batch processing with the LAGOS GIS Toolbox 6](#_Toc421200312)

[Batch processing using the graphical user interface 6](#_Toc421200313)

[Batch processing using Python scripting 7](#_Toc421200314)

[Data sources used throughout the toolbox 7](#_Toc421200315)

[Sub-toolboxes 8](#_Toc421200316)

[Lake Analysis 8](#_Toc421200317)

[Lake Connectivity Classification 8](#_Toc421200318)

[Lake Order Classification 9](#_Toc421200319)

[Upstream Lakes 9](#_Toc421200320)

[Lake-Wetland Connections 10](#_Toc421200321)

[Wetland Analysis 12](#_Toc421200322)

[Preprocess NWI 12](#_Toc421200323)

[Wetland Order 13](#_Toc421200324)

[Summarize Data by Zones 15](#_Toc421200325)

[Null values in Summarize Data by Zones tools 16](#_Toc421200326)

[Point Density 16](#_Toc421200327)

[Line Density 18](#_Toc421200328)

[Polygons in Zones 19](#_Toc421200329)

[Lakes in Zones 21](#_Toc421200330)

[Wetlands in Zones 23](#_Toc421200331)

[Streams in Zones 24](#_Toc421200332)

[Zonal Attribution of Raster Data 26](#_Toc421200333)

[Subset Overlapping Zones 29](#_Toc421200334)

[Zonal Attribution of Raster Data for Overlapping Zones 30](#_Toc421200335)

[Watershed Delineation 31](#_Toc421200336)

[Overview 31](#_Toc421200337)

[Scope 32](#_Toc421200338)

[Data sources 32](#_Toc421200339)

[Suggested Folder Structure 33](#_Toc421200340)

[Tool Order 34](#_Toc421200341)

[Stage and Mosaic Data for the Subregion 34](#_Toc421200342)

[Burn Flowlines into DEM 36](#_Toc421200343)

[Clip DEM to HU8 Boundaries 38](#_Toc421200344)

[File transfer to high-performance computing cluster (1) 40](#_Toc421200345)

[Pit Remove (DEM Conditioning) – TauDEM/HPCC 40](#_Toc421200346)

[Create HU8 Walls 43](#_Toc421200347)

[Flow Direction 44](#_Toc421200348)

[TauDEM to ArcGIS Flow Direction 46](#_Toc421200349)

[Create Pour Points 48](#_Toc421200350)

[Create Lake Watersheds 50](#_Toc421200351)

[Merge Lake Watersheds by Subregion 54](#_Toc421200352)

[Aggregate Watersheds: Interlake Mode 54](#_Toc421200353)

[Aggregate Watersheds: Cumulative Mode 60](#_Toc421200354)

[Utilities 61](#_Toc421200355)

[Export to CSV 61](#_Toc421200356)

[Merge NHD Features 61](#_Toc421200357)

[References 62](#_Toc421200358)

[Michigan State University HPCC Users Appendix 63](#_Toc421200359)

# Overview

This toolbox provides a set of tools that simplify the complex geoprocessing and modeling of hydrologic data over vast regions. The tools are designed to minimize user parameter inputs, making them easy to use. The tools in this extension to ArcGIS 10.1 are designed to automate tasks in the following general categories:

1. The automation of geoprocessing workflows that lead to quality watershed delineations.
2. Tools to help add attributes to lakes and the regions in which they reside from a variety of data sources.
3. Tools to create custom attributes for lakes or wetlands to help identify their situation and connectivity to the landscape.
4. Tools to summarize continuous data (such as climate data) in a consistent way for a variety of spatial extents.

The purpose of creating this toolbox was twofold. The first objective was to provide attribution for LAGOS_GEO_. The second was to provide a methodical legacy that could be used to easily recreate the same geospatial and tabular data products in the future as input data sources evolve.

The tools in this toolbox are developmental and targeted to our research environment. Several were intended to be used only once and very little time was spent producing code for elegant error handling or to enable processing of a wide variety of data formats. That being said, the tools generally require freely available geodata, in standard form, such as the National Hydrography Dataset (NHD), the National Elevation Dataset (NED), and the National Wetlands Inventory (NWI). The toolbox was designed for use in the US and relies heavily on US federal geodata products. However, there are some general tools in the box that are useful in any number of workflows, making the toolbox at least marginally useful outside the context of US based limnological research. Some tools require that the Spatial Analyst extension be installed. Only one tool, the Lake Order Tool, requires an input feature class with a field (Strahler) that must be produced outside of ArcGIS or this toolbox.

Extensions that complement the toolbox and/or are required for it to have full functionality include:

Spatial Analyst - http://www.esri.com/software/arcgis/extensions/spatialanalyst

TauDEM- http://hydrology.usu.edu/taudem/taudem5/index.html

RivEX- http://www.rivex.co.uk/

The toolbox was written in Python, it is open-source, and it can be freely distributed and modified. It comes with no guarantees or technical support of any kind, but questions, feedback and contributions are always welcome.

# Testing the tools

To test tools, except for Summarize Data by Zones, an ArcGIS file geodatabase of sample data called TestData_0411.gdb is packaged with the toolbox to assist with the testing and to demonstrate the tools in the Summarize Data by Zones sub-toolbox. The sample data covers the HU8 region with HU 04110003. The HU12 feature class is the best feature class to use as a zone for demonstrating how the tools behave and to quickly test any changes.

**Developers only:** Each module has a function called test() where the file paths to useful, illustrative test inputs can be saved. The script test_tools.py can then be altered to import the module that you wish to test and will run the test() function, allowing the outputs to be overwritten and the running time of the tool to be printed.

# Hardware and Software

**The estimated running times of tools are based on performance in the following environments.**

**Dell Precision T1600**

**Processor:** Intel Xeon CPU E3-1280 @ 3.50 GHz (Quad-core)

**Memory:** 16.0 GB RAM

**Operating System:** 64-bit Windows 7

**MSU HPCC (High Performance Computing Cluster)—example cluster:**

**Typical Processor:** 2.3 GHz Quad-core Intel Xeon E5345 (2 per node)

**Memory:** 8 GB per node

**Operating System:** Red Hat Enterprise Linux 6.3 using Torque for resource management

**We requested 8-9 nodes using 1 processor per node for each job.**

We recommend a workstation running 64-bit Windows (7 or higher) with at least 8 GB of RAM and ArcGIS 10.1 or higher installed with a Spatial Analyst license. ArcGIS uses single-threaded processing for almost all tasks. CPU speed is the main bottleneck in most tools. Another limiting factor in processing speed is disk read/write speed.

ArcGIS 10.1 or higher is required because this toolbox uses the Data Access module that was introduced with this release. We strongly recommend using this toolbox with 64-bit Background Geoprocessing installed and turned on (see the 'Background Geoprocessing (64-bit)' in the ArcGIS Help for more details). Without 64-bit Background Processing, only 2 GB of RAM is available to ArcGIS, however, many of our tools are designed to work effectively with datasets larger than that.

We used the RivEX 10.6 proprietary software to calculate the Strahler number for each segment in our line-based flow network. This step must be completed outside of the LAGOS GIS Toolbox.

Finally, we used the TauDEM 5.2 (Terrain Analysis Using Digital Elevation Models) command line tools in the HPCC environment. Our workflow used two of the TauDEM suite's proven and stable algorithms: Pit Remove and D8 Flow Direction. Although these analyses can be performed using ArcGIS, TauDEM tools use multiprocessing via MPICH2 to implement message passing between multiple processes, which makes it more feasible to use when working with many large datasets. To use the TauDEM tools, GEOTiffs cannot exceed 4 GB.

Finally, ArcGIS was not installed in the HPCC environment, so we used the GDAL (Geospatial Data Abstraction Library) command line utilities for data translation and processing tasks, as needed.

## Using the HPCC (for MSU Users)

See the Michigan State University HPCC Users Appendix.

# Batch processing with the LAGOS GIS Toolbox

There are two possible ways to run these tools in batch runs, which are explained below.

## Batch processing using the graphical user interface

In the 10.1 ArcCatalog application, it is easiest to set up many runs of these tools by using the 'Results' window to alter the first run with new parameters. For instance, to run the Line Density tool for streams data and different zones (HU12, HU8, HU6, HU4):

1. Open the tool that you want to run repeatedly and then fill out all of the information using the first zones feature class in your list, say, HU4. Click 'OK' to start running the tool.
2. Ensure that the Results window pane is displayed. If it is not, open the 'Geoprocessing' menu option in the main menu and click on 'Results'.
3. In the Results window pane, click '+' to the left of Current Session to show a list of previous tool runs. Double-click the most recent item with the name of the tool that you just started, and then alter the zones feature class and the output table name by either typing over the old text (change 'HU4' to 'HU6', etc.) or using the menus to navigate to your next zones feature class and output.

This method is faster, easier, and it permits fewer mistakes than running the tool with blank parameters over and over again. In addition, with a little patience many executions can be staged to run sequentially in a few minutes. If a task in the queue fails, ArcGIS will indicate a failure and move on to run the remaining tasks in the queue.

The built-in batch method in 10.1 (accessed by right-clicking on the tool/script name and selecting Batch) is not very robust to failures: when one item fails, none of the items after it will be run. Our toolbox has limited error handling capabilities and easily corrected failures frequently arise while the analyst is becoming familiar with their data and these tools.

## Batch processing using Python scripting

The following description provides a detailed walk-through of how to set up a batch processing script in Python:

An example script called batch_raster_attribution.py is included in the toolbox directory and can be edited directly to create a new batch run of Summarize By Zone tools, or it can be used as a suggestion/template for new code. Any number of rasters can be included in the list variable called ALL_RASTERS and any number of zone feature classes (without overlapping features and sharing a common ID field) can be included in the list variable called ALL_EXTENTS. Entering items in the list by hand, or collecting them using Python or ArcGIS directory listing methods requires some of the analyst's time. The batch function checks the existence of all of the paths before attempting any calculations and it will identify which paths have failed the existence check.

Once all of the paths are valid, this script can execute Zonal Attribution for Raster Data repeatedly for a very long time. It prints in both the interactive Python window and the optional text log which table it is currently creating, how long the table took to create, and whether the calculation of a table has failed. If an output cannot be created (e.g., a tool failure of some kind), then the batch script will print the traceback and move on to the next parameter combination. It can also be stopped and re-started at any time with minimal time lost because it will check for tables that were already calculated. You may have to sift through a lot of output text in the log to find information about a particular table.

# Data sources used throughout the toolbox

For information on the data sources used in these tools, please see Additional file 5,7,9-11.

# Sub-toolboxes

The LAGOS GIS Toolbox is functionally organized into five sub-toolboxes, which are described in detail below:

Lake Analysis

Wetland Analysis

Summarize Data by Zones

Watershed Delineation

Utilities

Each of these toolboxes has a group of related scripts within it. In the Watershed Delineation and Wetland Analysis toolboxes, the scripts may be numbered in the order that they should be used.

## Lake Analysis

This toolbox contains the following tools that use lakes as the unit of analysis:

- Lake Connectivity Classification
- Lake Order Classification
- Lake-Wetland Connections
- Upstream Lakes

### Lake Connectivity Classification

#### Description

This tool classifies lakes based on hydrologic connectivity and adds the classification in a new field called 'Connection' in the output feature class. The Lake Connectivity Classifications are:

**Isolated:** Has no stream connectivity.

**Headwater:** Has one outlet and no upstream connectivity.

**DR_ Stream (Drainage Stream):** Has upstream connectivity to streams and/or lakes < 10ha in area.

**DR_LakeStream (Drainage Lake Stream):** Has upstream connectivity to streams and lakes > 10ha in area.

#### Inputs

- **High resolution (24k) NHD file geodatabase for a NHD subregion:** High resolution (24k) NHD subregion file geodatabase.
- **Output folder:** Folder location where the output will be written.

#### Output(s)

The output is a feature class containing all of the input lakes. The 'Connection' field will be added and populated with the calculated values.

#### QA/QC

We performed an error analysis by examining three river watersheds in Michigan. A random selection was used to select approximately 100 lakes to validate for each one. The calculated connectivity value was compared to the connectivity evaluated by mapping the NHD data and performing a visual evaluation.

| **Watershed** | **# Lakes Evaluated** | **# errors** |
| --- | --- | --- |
| Huron Watershed (04090005) | 106 | 1 |
| Pere Marquette (04020202) | 102 | 1 |
| Tahquamenon (04060101) | 100 | 0 |
|  |  |  |

The overall error rate was 0.65%. Both of the lakes in error were identified as headwater lakes instead of lakes with upstream connectivity and the errors were found to be due to the inclusion of problematic artificial paths in the NHDFlowline feature class within the boundaries of the lake.

#### Source code location

LAGOS_GIS_Toolbox/LakeConnectivity.py

### Lake Order Classification

#### Description

This tool classifies lakes according to both connectivity to the hydrologic network and the Strahler stream order at the lake's pour point. A Strahler stream order field calculated for the input stream feature class is required to run the tool. This is fairly easy to produce using the RivEX extension to ArcGIS, which is available at: http://www.rivex.co.uk/

#### Inputs

- **RivEX stream network polyline with Strahler:** This is a stream line feature class that has a Strahler field with numbers representing the Strahler stream orders. For this project, we used RivEX software to add this field to High Resolution NHD Flowlines. Although RivEX is not free, it is reasonably priced.
- **Output from Lake Connectivity Classification tool:** As a prerequisite to running this tool, you must run the Lake Connectivity Classification tool. The output is used for this parameter.
- **NWI that covers the whole NHD subregion: This is the** NWI dataset that covers the NHD subregion (HU4). It is likely that you will have to merge NWI state datasets because the NWI is distributed by state and subregions that do not conform to state lines.
- **Output folder:** This is the folder where the output will be written.

#### Source code location

LAGOS_GIS_Toolbox/LakeOrder.py

### Upstream Lakes

#### Description

This tool counts the number and area of each upstream lake in two classes: lakes greater than 4 ha and lakes greater than 10 ha. This tool uses network analysis to search upstream. 'Upstream' stops at the subregion boundaries because this tool is run on individual subregions.

#### Inputs

- **The NHD geodatabase for the subregion to be processed:** **This is the NHD geodatabase for the subregion (HU4) you want to process. Although we recommend using a copy rather than the original, this tool will not alter the original.**
- **Output table:** The location of the output table.

#### Output(s)

The output is a table with a record for each lake in the subregion as defined using the definition established for the LAGOS database (i.e., greater than 4 ha and with an appropriate FCode). This will be less than the number of records in the NHDWaterbody. The columns in the table are:

Permanent_Identifier

Upstream_lakes_4ha_Count

Upstream_Lakes_10ha_Count

Upstream_Lakes_4ha_Area_ha

Upstream_Lakes_10ha_Area_ha

#### Warning

Upstream networks stop at the HU4 subregion boundaries.

#### Tool steps summary

1. Identify the necessary items in the NHD geodatabase and set the environments.
2. Create the layer of lakes greater than 4ha and the layer of lakes greater than 10ha.
3. Add fields to the output table for count and total area.
4. Using a search cursor, for each lake:
   1. Make a layer containing only this lake.
   2. Select junctions (points at each hydrological connection) within 1 m of this lake.
   3. If the number of junctions is 0, then there is no upstream network at all. Enter 0s in all the output fields.
   4. If the number of junctions is at least 1:
      1. Trace the geometric network upstream of the junction(s) for this lake.
      2. Select lakes that intersect the flowlines in the trace (these are the upstream lakes).
      3. If there are no lakes in the upstream network, then enter 0s in the output fields.
      4. If there are lakes in the upstream network:
         1. Create a layer of the 4ha lakes, count them, and using a search cursor, sum their area. Update the output fields with these values.
         2. Create a layer of the 10ha lakes, count them, and using a search cursor, sum their area. Update the output fields with these values.

#### Time

Working on all of the subregions for our 17-state region takes about 12 hours. For each subregion, the minimum time is a few minutes and the maximum time is about an hour.

#### Source code location

LAGOS_GIS_Toolbox/upstream_lakes.py

### Lake-Wetland Connections

#### Description

This tool identifies the wetland connections for each lake. Wetland connections are considered for all wetlands and for each of the following four classes derived from a selection with the VegType field: all wetlands, forested wetlands, scrub-shrub wetlands, open water wetlands, and other wetlands (see Additional file 11 for further details). Wetland connections are identified using a 30 m buffer around the lake to allow for digitization errors and they are described by the count of intersecting wetlands, the total area (in ha) of all the wetlands that intersect/touch each lake, and the length of the lake shoreline (in kilometers) that intersects the wetlands. We defined wetlands and lakes to be mutually exclusive (i.e., in our case all of the wetland regions within the NHD-derived lake boundaries were removed prior to running this tool).

#### Inputs

- **Lakes feature class:** This is a feature class containing the lakes that you wish to describe wetland connections for. There should be no extra lakes.
- **Lake ID field:** This field uniquely identifies each lake, which is usually 'Permanent_Identifier' when using the NHD.
- **Wetlands feature class:** This is a polygon feature class with wetlands data processed with the Wetland Order tool (and the Preprocess NWI tool). The VegType field should be present and no wetlands besides those that you want to be considered as connections should be present. The wetlands feature class should have a geographic extent greater than or equal to that of the lakes feature class.
- **Output table:** This is the location of the output table.

#### Output(s)

The output is a table with the following columns and one output record for every input lake.

AllWetlands_Count

Contributing_AllWetlands_AREA_ha

AllWetlands_Shoreline_Km

ForestedWetlands_Count

Contributing_ForestedWetlands_AREA_ha

ForestedWetlands_Shoreline_Km

ScrubShrubWetlands_Count

Contributing_ScrubShrubWetlands_AREA_ha

ScrubShrubWetlands_Shoreline_Km

OpenWaterWetlands_Count

Contributing_OpenWaterWetlands_AREA_ha

OpenWaterWetlands_Shoreline_Km

OtherWetlands_Count

Contributing_OtherWetlands_AREA_ha

OtherWetlands_Shoreline_Km

#### Warning(s)

Although the 30 m buffer is a standard throughout the toolbox, it may not always be sufficient to capture digitization errors. Lakes and wetlands may have been digitized at totally different times/seasons and connections might be missed, even with the 30 m buffer.

Shoreline lengths can add up to slightly more than the NHD lake shorelines because they are calculated from the buffered lakes.

#### Tool steps summary

1. Create a layer called 'lakes_30m' that is the lake polygons buffered by 30 m. Use this throughout the analysis instead of the lakes themselves.
2. Create a line-based lakes representation (from the buffered lakes) called 'shorelines'.
3. For each VegType selection (i.e., all, forested, scrub-shrub, open water, other):
   1. Use the polygons_in_zones function from the LAGOS GIS Toolbox with the lake as the zone, the wetlands as the polygon feature class of interest, and the current selection query (All, forested, etc.). This summarizes the selected wetlands by their INTERSECT relationship to the lake.
   2. Rename the output fields to be specific to this selection (e.g., 'Poly_Count' becomes 'ForestedWetlands_Count').
   3. Use the current selection query to make a 'selected_wetlands' layer.
   4. Intersect the 'shorelines' layer with the 'selected_wetlands layer.
   5. Sum the length of all of the shoreline-wetlands intersection for each lake: this is the shoreline connection length.
   6. Ensure that each input lake receives an output record, even if it has no wetland connections and the values are 0.
   7. Join the temporary output from step 3a to the shorelines output to make the final table for this selection query.
4. Once all of the selections have been calculated, join all of the tables together and ensure that the fields are populated with 0s, even if, for instance, there were no 'other' wetlands.

#### Source code location

LAGOS_GIS_Toolbox/ConnectedWetlands.py

## Wetland Analysis

This toolbox contains the following tools for processing NWI wetland data and using wetlands as the unit of analysis:

- Preprocess NWI
- Wetland Order

### Preprocess NWI

#### Description

This tool addresses the following three differences between the data that the NWI delivers and the wetlands data that are needed for our project:

1. The NWI data are delivered by state, with a fairly generous overlap between states.
2. We define wetlands as palustrine wetlands that are not freshwater ponds (see Additional file 11).
3. NHD-based lakes overlap with NWI wetlands. We choose to prefer the NHD representation of the landscape and erase any wetland regions that fall within lakes (any bordering wetland region in the same polygon will be retained).

This tool selects only the wetlands with their centroid within each state of the correct type and it performs the erase analysis. It also adds an ID field to use for each wetland called 'WET_ID' and a field with the wetland area in ha. The resulting feature classes are able to be merged together seamlessly (for instance, to have one feature class that covers our 17-state region) using the ArcGIS Merge or Append tools.

#### Inputs

- **State polygon:** This is a polygon of the US state for which the NWI dataset will be processed. It should be a single polygon that expresses the state's extent/borders. Features outside of this polygon will be dropped from the output feature class.
- **NWI State Feature class:** This is the NWI CONUS_wet_poly feature class in the CONUS_wetlands feature dataset for the state geodatabase that you want to process
- **Lakes feature class:** This is a polygon feature class containing lakes according to the official definition (i.e. non-lake waterbodies should already have been filtered out if they were present because this tool does not subset the lakes in any way). This feature class should have a geographic extent that exceeds the wetlands. We recommend creating this layer for your study region and re-using it for each state's wetlands, as well as for other analyses.
- **Output feature class:** This is the location of the output feature class. Please use a geodatabase to retain the full length of field names, which are used again later.

#### Output(s)

The output will be a feature class with only the wetlands in this state. Although the wetlands may extend beyond the state border, their centroid will fall within the state. This ensures that they cannot be present in more than one state's output. There will be only palustrine wetlands and no freshwater ponds. Wetland polygons will be altered where they used to overlap with lakes because the overlap will be erased (anything within the lake polygon will be cut out).

#### Warning

Be careful to use an input lakes feature class that meets your project's definition of a lake.

#### Tool steps summary

1. Set up environments: outputs will be saved in the USGS Albers projection.
2. Select wetlands that 'HAVE_THEIR_CENTER_IN' the state polygon.
3. From the selection in step 2, do a subset selection of wetlands with this query: 'ATTRIBUTE' LIKE 'P%' AND 'WETLAND_TYPE' <> 'Freshwater Pond'.
4. Use the Erase analysis to erase the regions of wetlands that fall within the lakes feature class.
5. Add the field 'WET_ID' and copy the OBJECTID field to create a permanent ID for each wetland.
6. Add the field 'AreaHa' and calculate the area of the wetland polygon in ha.

#### Source code location

LAGOS_GIS_Toolbox/PreprocessNWI.py

### Wetland Order

#### Description

This tool assigns a class to wetlands based on their connectivity to the landscape. In addition, this tool reclassifies the wetlands into four vegetation types (VegType) based on their attribute code (these classes are discussed in detail in Additional file 11). It extracts the regime from the attribute code and assigns it to the new field Regime. More information about the attribute codes in the NWI data can be found at: http://www.fws.gov/wetlands/data/wetland-codes.html.

The Wetland Order tool assesses the spatial connectivity of wetlands and streams, adding new fields to the NWI attribute table, which include:

**WetlandHa –** This field is the ArcGIS calculated area of the NWI wetland polygon in ha once the data has been projected to EPSG 102039- USGS Albers Equal Area.

**Wet_ID** – This identifier is simply an OBJECTID that gets transferred to a new field. This is done because using the OBJECTID as an identifier is inadvisable because there are geoprocessing tasks that can alter the OBJECTID.

**StrOrdMax** – As a prerequisite to running this tool, Rivex software is used to attribute the NHD 24k Flowlines with a Strahler Order field. StrOrdMax is the maximum Strahler Stream Order value of streams that intersect or come within 30 meters of intersecting the wetland perimeter. This is useful because it provides an idea of the magnitude of the wetland's most significant hydrologic connection.

**StrOrdSum** – This is similar to the StrOrdMax but is the sum rather than the maximum value of Strahler Stream Order values for intersecting streams (within 30 m). This metric is useful because it helps to conceptualize the total connectivity of the wetland patch to the hydrologic network. Research indicates that Strahler order provides a surprisingly useful approximation of the ranges of catchment size, distance to source, modeled mean annual discharge, and field-based low-flow and bank-full channel dimensions for most streams within a given Strahler order [2]. So, in theory, this is a better measure of connectivity than a simple count of intersecting streams because streams of greater Strahler Order should, in very general terms, be larger in size and have greater discharge than lower order streams within the same area of the hydrologic network. This metric could be useful for characterizing the wetland's potential positive influence on water quality as it relates to the amount of water flowing through it.

**StreamKm –** This is the sum of the line lengths of intersecting stream *segments*. Segments are often from confluence to confluence. This metric, if divided by stream count, would yield an average distance between confluences for the intersecting segments.

**VegType -** The NWI code field 'ATTRIBUTE' is reclassified and simplified to four palustrine wetland vegetation types:

PEMorPAB = Palustrine emergent or palustrine aquatic bed

PSS = Palustrine shrubs and scrub

PFO = Palustrine forested

Other = Palustrine, but otherwise different from those above.

**WetOrder -** Wetland Order, this tool's namesake, is a simple assessment of wetland connectivity to streams in the hydrologic network. Wetlands are classified as being:

Isolated – the wetland is not intersected (within 30 m) by streams.

Single – the wetland has a single 1st order stream intersecting its perimeter (within 30 m)

Connected – the wetland has a > 1st order stream or multiple streams intersecting its perimeter (within 30 m)

#### Inputs

- **Rivex feature class:** Stream lines attributed with a field named 'Strahler' that contains the Strahler stream order of each stream feature. This can be produced with RivEX software and the NHDFlowline feature classes. The geographic extent of this feature class should exceed that of the wetlands feature class.
- **Stream polygons feature class:** A polygon feature class with only the StreamRiver polygons from the NHDArea. This can be the output of the Merge NHD Features tool. The geographic extent of this feature should exceed that of the wetlands feature class.
- **Wetlands feature class:** A polygon feature class with wetlands. This should be the output of Preprocess NWI (or the result of merging the outputs).
- **Output feature class:** This is the location of the output feature class

#### Output(s)

The output will be a wetlands feature class that looks similar to the input but with the addition of the fields (note: the wetland polygon shapes have not changed).

StrOrdMax

StrOrdSum

WetOrder

VegType

Regime

StreamKm

StreamCnt

#### Tool steps summary

1. Only process the extent of the wetlands feature class.
2. Create a layer called 'wetland_buffers' by buffering wetlands 30 m.
3. Update the extent environment to be equal to the extent of buffered wetlands feature class.
4. Select rivex features (stream centerlines) that are completely within the stream polygons and copy to a new feature class called 'rivex_for_splitting'.
5. Switch selection to stream centerlines that are NOT completely within the stream polygons and copy these to 'rivex_not_areas'.
6. Split stream polygons up by Strahler order (previous to this step, they do not stop and start at confluences, nor do they share IDs with their stream centerlines):
   1. Calculate a Euclidean allocation raster: for each cell, calculate the nearest stream.
   2. Convert the allocation raster to polygons.
   3. Join the 'Stahler', 'LengthKm', and ID fields from the streams to the allocation polygons from step 6b.
   4. Use an Identity analysis to split the stream polygons using the allocation polygons (this will cut them up very close to but not exactly at the confluences).
7. Convert the split stream polygons from step 6 (which now have centerline-based IDs, Strahler orders, and reach length assigned) to lines.
8. Merge the polygon-derived lines from step 7 with the stream centerlines for segments that were not also represented by polygons from step 5: This line feature class now has a centerline for most streams and a double-banked LINE-based representation for large streams/rivers.
9. Establish the following field mappings and spatially join 'wetland_buffers' to the output of step 8:
   1. StrOrdSum is the sum of the Strahler numbers for streams intersecting 'wetland_buffers'.
   2. StrOrdMax is the maximum of the Strahler numbers for streams intersecting 'wetland_buffers'.
   3. StreamKm is the sum of the LengthKm field for streams intersecting 'wetland_buffers'. It is important to note that this is not the length of stream that actually intersects the wetland buffers.
   4. StreamCnt is the count of the stream features that intersect 'wetland_buffers'.
10. Join the new fields to the original wetlands feature class ensure every input wetland is in the output.
11. Classify the VegType field from the ATTRIBUTE code.
12. Classify the Regime field from the ATTRIBUTE code.
13. Classify the WetOrder field:
    1. If the StrOrdSum value is 0, the wetland is classified 'Isolated'.
    2. If the StrOrdSum value is 1, the wetland is classified 'Single'.
    3. If the StrOrdSum value is >1, the wetland is classified 'Connected'.

#### Source code location

LAGOS_GIS_Toolbox/WetlandOrder.py

## Summarize Data by Zones

This toolbox contains the following tools for summarizing variables at a chosen level of analysis (zone feature class):

- Line Density
- Point Density
- Polygons in Zones
- Lakes in Zones Full Report
- Wetlands in Zones Full Report
- Zonal Attribution of Raster Data
- Subset Overlapping Zones
- Zonal Attribution of Raster Data for Overlapping Zones
- Wetlands in Zones
- Lakes in Zones
- Streams in Zones

These tools are used to summarize data within 'zones' bounding area polygons, such as counties, watersheds, and hydrologic units (HUs). Each tool produces a table in which there is a record for each input zone and a column/field for each statistic calculated by the tool, as well as a column/field for the zone identifier.

For our project, we used a variety of GIS data types: point data, line data, polygon data, and raster data. The analysis for each of these is different, so the users must start by selecting the right tool for the type of data that they want to summarize.

| **Type of data you wish to summarize** | **Tool** |
| --- | --- |
| Point (e.g., dams: count, density) | Point Density |
| Line (e.g., streams, roads: total length, density) | Line Density, Streams in Zones if appropriate |
| Polygon (e.g., lakes, wetlands: count, total area) | Polygons in Zones, Lakes in Zones or Wetlands in Zones if appropriate |
| Raster (e.g., land cover, precipitation: mean, standard deviation, minimum, maximum, area by class) | Zonal Attribution of Raster Data  or, if the zone polygons overlap each other:  1) Subset Overlapping Zones  2) Zonal Attribution of Raster Data for Overlapping Zones |

The Line Density, Point Density, and Polygons in Zones tools have an option to execute a SQL selection expression on the input data to analyze only the subset of the point, line, or polygon input data by attribute (geographic selections must be performed by the analyst prior to using these tools). For instance, if you are using a lakes feature class that has been categorized by connectivity with the Lake Connectivity Classification tool, then you can use a selection to calculate the number and total area of *only* the 'Isolated' lakes with the Polygons in Zones tool.

Three tools in the toolbox simplify that calculation of the statistics for all of the selections/groupings that we were interested in for streams, lakes, and wetlands, which are: Streams in Zones, Lakes in Zones, and Wetlands in Zones.

A special two step workflow is needed to summarize the raster data when the zones feature class contains overlaps between neighboring zone polygons. For instance, using our definition of watersheds for LAGOS lakes (the interlake watersheds), their polygons can overlap each other in some instances, as do the lake buffer polygons. The analyst must first use the Subset Overlapping Polygons tool, and then open the Zonal Attribution of Raster Data for Overlapping Polygons tool and select *all* of the outputs from the Subset Overlapping Polygons for the first input. Overlapping polygons are not a problem with the point, line, or polygon data types and a special workflow is not needed.

### Null values in Summarize Data by Zones tools

We recommend using a file geodatabase to store table outputs because dBASE tables will not output fields with names longer than 10 characters. The Export to CSV tool in our Utilities sub-toolbox can be used to convert the table to a CSV file that can be used and edited in other software, such as Excel and R.

ArcGIS uses <Null> to represent values in a table with no data. All of our tools for vector data types assume that the data being summarized equals or exceeds the zone polygons in extent. One of the final steps in each tool is to change <Null> values in the output table to 0 values to indicate that there really were no points, lines, or polygons in these zones. Raster data present another problem in that sometimes the zones are too small compared to the resolution of the raster and they do not have any statistics calculated for them. For these records, these tools record <Null> values in the output. However, our Export to CSV tool converts the <Null> values to 'NA' (string) values in the CSV file, which are automatically read in as missing data when imported directly into R.

### Point Density

#### Description

This tool calculates the following three values:

1) Number of points inside a zone ('PointCount');

2) Density of points inside a zone in 'points per hectare' units ('PointsPerHa'); and,

3) Density of points inside a zone in 'points per square kilometer' units ('PointsPerSqKm').

It was originally designed to summarize the number of dams within zones, such as the HU12 zones.


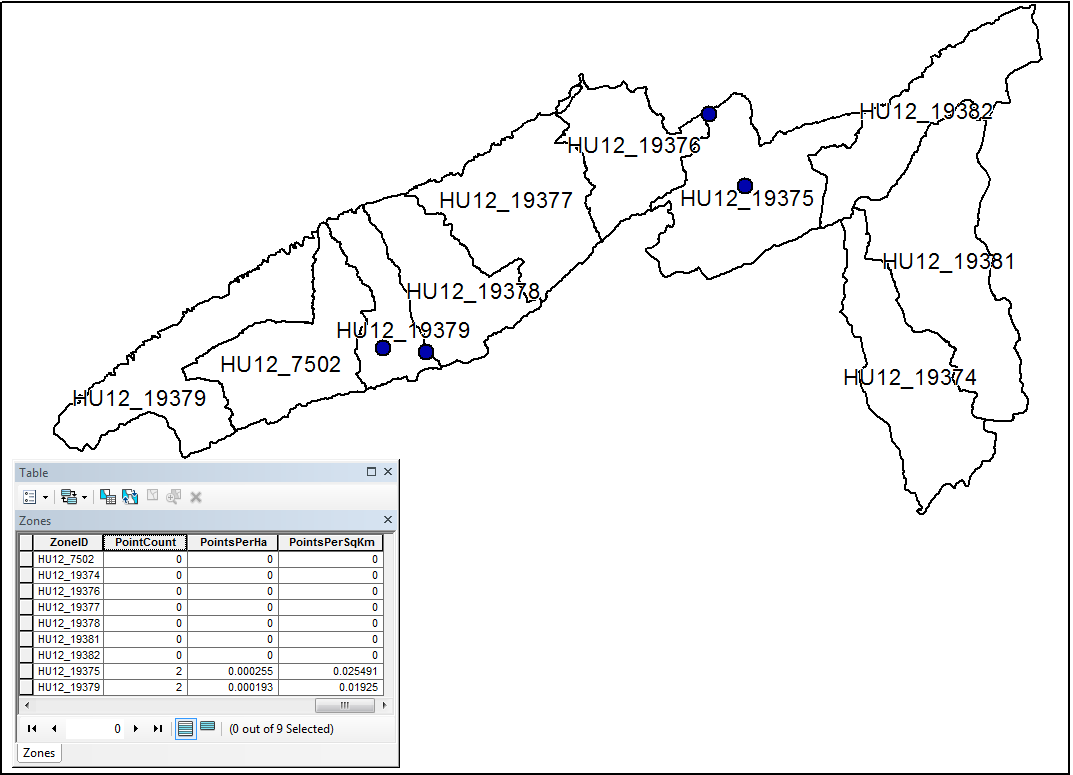


**Figure 1. Dam density using the point density tool.**

#### Inputs

- **Zone feature class:** This is the polygon feature class containing the zones that you want to summarize data for.
- **Zone field:** This is the field that uniquely identifies each zone.
- **Points feature class:** This is the point feature class containing the point data that you want to summarize by zone (e.g., dams). It should contain all of the points that you want to describe and it should have no extra points (however, you can filter points by attribute using the 'Points selection expression' parameter).
- **Points selection expression:** This is an SQL expression limiting which point features will be included in the analysis.
- **Output table:** This is the location of the output table.

#### Output(s)

The output is a table with one record per input zone and three calculated fields, as described above. 0 is the smallest possible value and null values are not produced by this tool.

#### Warning(s)

The input data should be best described by points (i.e. features that are point-based data not polygons). For LAGOS, the only data we have of this type are dams. If you try to use this tool on lake polygons converted to points, for instance, you will get a different number of lakes per zone and a less meaningful density value than if you used polygon lake data as an input to the Polygons in Zones tool.

This tool assumes that the point data has an extent greater than or equal to the zones data. If this is not true, then the user should create a workflow to calculate the fraction of the zones with point data available, and then use that fraction to adjust the values resulting from this tool.

#### Tool steps summary

1. Spatially join (INTERSECT) points to the zones, one record per zone in output, keep a record for each zone regardless of whether it has points intersecting it.
2. Rename Join_Count to PointCount in the output from step 1.
3. Calculate the two density metrics by dividing number of points by the zone's area derived from its geometry.

#### Time

This tool usually takes under 2 minutes to run with our dam data.

#### Related ArcGIS help topics

Spatial Join

#### Source code location

LAGOS_GIS_Toolbox/PointDensityInPolygons.py

### Line Density

#### Description:

This tool calculates the following two values:

1) Total length of lines (e.g., streams, roads) in the zone (meters) ('SUM_LengthM').

2) Density of lines in the zone (meters per hectare) ('Density_MperHA').

This is useful for determining the prevalence of features like streams or roads in a given area. For convenience, we have created the Streams in Zones tools to streamline the use of this tool for multiple groupings of lakes and wetlands data. To calculate values such as total length of streams by stream order, first create feature classes separating the stream orders by selecting specific classes using the Select function in ArcGIS and then use these feature classes as inputs to this tool.

#### Inputs

- **Zone feature class:** This is the polygon feature class containing the zones you want to summarize data for.
- **Zone field:** This is the field that uniquely identifies each zone.
- **Lines feature class:** This is the line feature class containing the line data you want to summarize by zone (e.g., streams, roads). It should contain all of the lines that you want to describe and it should include no extra lines.
- **Lines selection expression (optional):** This is an SQL expression limiting which line features will be included in the analysis.
- **Output table:** This is the location of the output table.

#### Output(s)

The output is a table with one record per input zone and two calculated fields, as described above. 0 is the smallest possible value and null values are not included in the output but are internally converted to 0 by this tool.

#### Warning(s)

This tool assumes that the line data has an extent greater than or equal to the zones data. If this is not true, then the user should create a workflow to calculate the fraction of the zones with line data, and then use that fraction to adjust the values resulting from this tool.

#### Tool steps summary

1. Use ArcGIS Identity (analysis) tool to crack lines at polygon boundaries and append zone information to line segment ('lines_identity').
2. Add 'LengthM' field and calculate line segment length for 'lines_identity'.
3. Summarize 'lines_identity' by zone: 'SUM_LengthM'.
4. Copy the zone area field from the input to the output.
5. Add the 'Density_MperHA' field and calculate by dividing 'SUM_LengthM' by zone area in ha.
6. Ensure zones with no line data inside are included in the table with a value of 0.

#### Related ArcGIS help topics

Identity, Statistics

#### Source code location

LAGOS_GIS_Toolbox/LineDensity.py

### Polygons in Zones

#### Description

This tool calculates the following four values:

1) Number of polygons (lakes or wetlands) intersecting zone (Poly_Count).

2) Total overlapping area of the polygons intersecting the zone (Poly_Overlapping_Area_ha).

3) Percent of zone covered by polygons (Poly_Overlapping_Area_pct).

4) The sum of the total area of polygons intersecting the zone (Poly_Contributing_Area_ha), which can be divided by the count to get average polygon (wetland, lake, etc.) size.

Although this tool was originally designed to be used to summarize lake data and wetland data by zones, it is also used internally in the Lake-Wetland Connections tool. For convenience, we have created the Wetlands in Zones and Lakes in Zones tools to streamline the use of this tool for multiple groupings of lakes and wetlands data. To calculate values such as 'number of lakes that are isolated' outside of these additional tools, use the 'Polygons of interest' selection expression to select features by attribute(e.g., 'Connection' = 'Isolated').

#### Inputs

- **Zone feature class:** This is the polygon feature class containing the zones that you want to summarize data for.
- **Zone field:** This is the field that uniquely identifies each zone.
- **Polygons feature class:** This is a feature class that contains all of the polygon features that you want to summarize by zone (e.g., lakes, wetlands). It should contain all of the polygons that you want to describe and it should contain no extra polygons (although you can use the selection expression below to filter by attribute).
- **Polygons selection expression (optional):** This is an SQL expression limiting which lake/wetland/other features will be included in the analysis. For instance, if 'Zone feature class' is 'all lakes >1ha in our area' and you use a selection like ' 'Area_HA' > 4', then only lakes greater than 4 ha will be summarized during this run of the tool.
- **Output table:** This is the location of the output table.

#### Output(s)

The output is a table with one record per input zone and four calculated fields, as described above. 0 is the smallest possible value and null values are not included in the output but are internally converted to 0 by this tool. Poly_AREA_ha values cannot exceed the area of the input zone. Poly_AREA_pct values cannot exceed 100%.


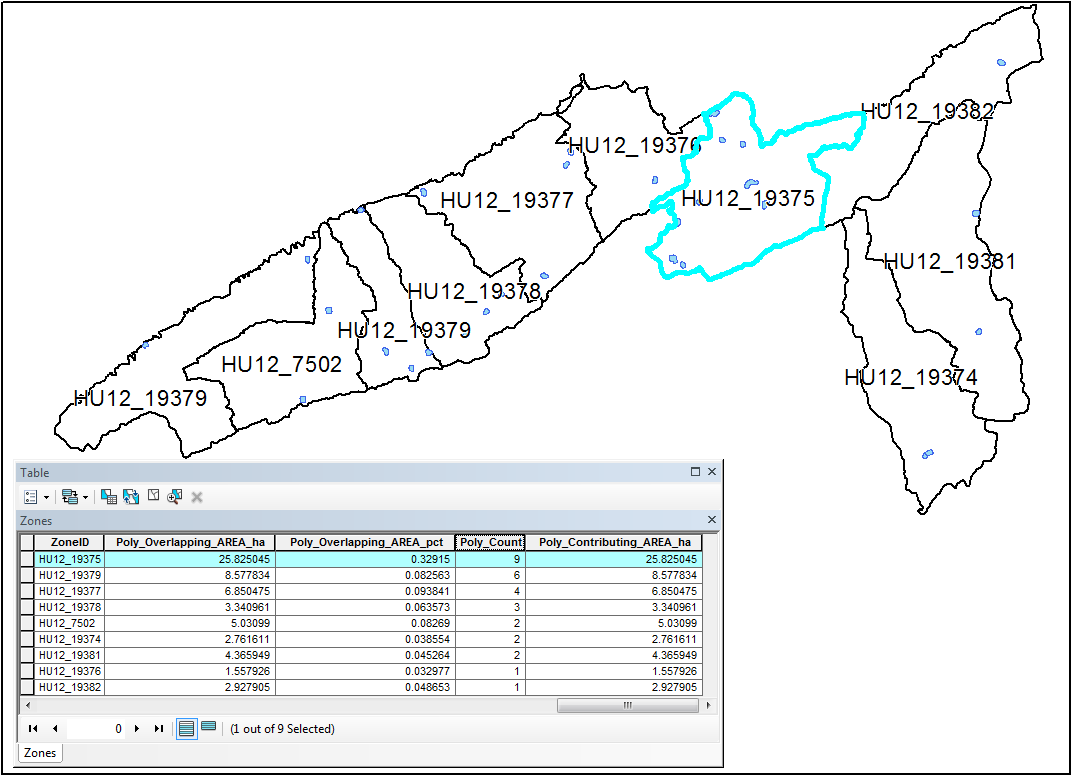


**Figure 2. Demonstration of polygons in zones tool using lakes as the polygon data to summarize.**

#### Warning(s)

The users should spend some time with maps of the polygons of interest (wetlands, lakes, etc.) and zones to understand how to interpret these values In particular, the user should look at how often wetlands and lakes cross boundaries, and what size they have relative to zones.

#### Tool steps summary

1. Make a feature layer from the polygons of interest feature class, including only features selected by the selection expression if one is provided.
2. Use Tabulate Intersection analysis to calculate the area of intersection and the percent of each zone that intersects wetlands.
3. Use a spatial join to count how many polygons intersect the zone.
4. Ensure that each input zone is represented by a record in the output and if there are no polygons in the zone (assume complete data coverage) the value is 0.


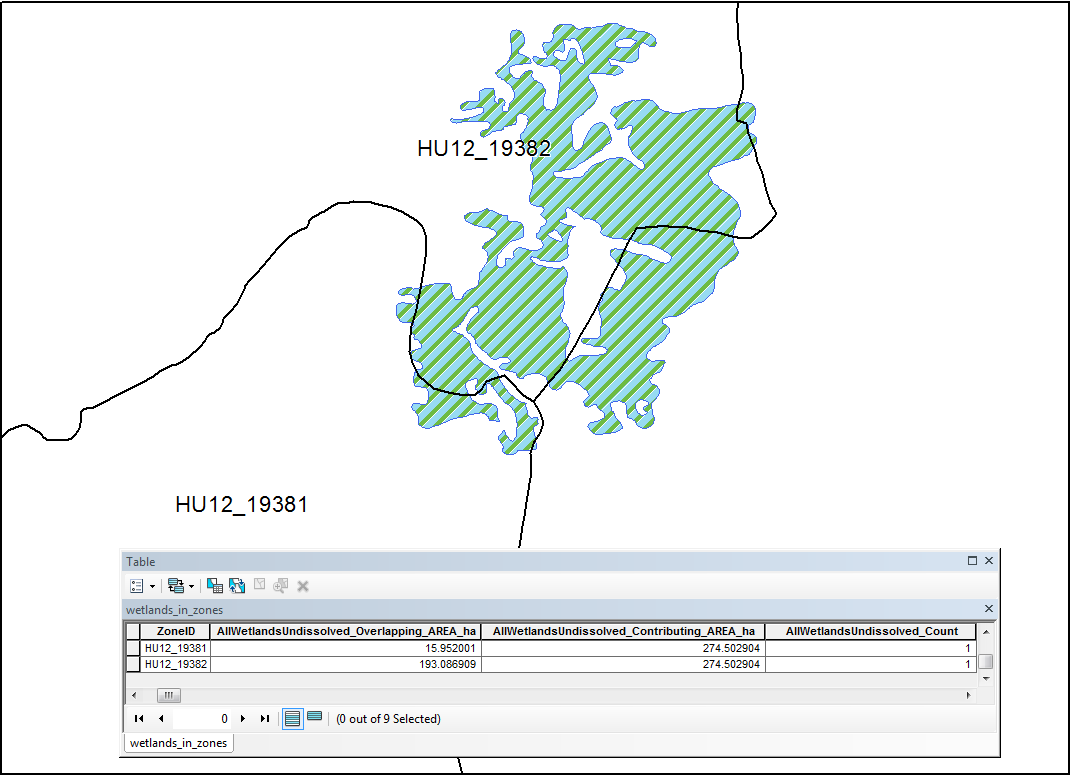


**Figure 3. This output is from the Wetlands in Zones tool but is shown here so that the users can see how polygon data that crosses zones is treated.** This is one wetland polygon. Its CONTRIBUTING area is counted the same in each zone. Overlapping values are the portion in each zone. Count is 1 for each zone.

#### Related ArcGIS help topics

Tabulate Intersection, Spatial Join

#### Source code location

LAGOS_GIS_Toolbox/polygons_in_zones.py

### Lakes in Zones

#### Description

This tool is a wrapper around the Polygons in Zones tool that summarizes lake data using all the subsets/groupings that we wanted to explore for developing LAGOS. For each grouping, this tool calculates the following four values that are the same as in the Polygons in Zones tool:

1) Number of lakes intersecting zone ([label]_Count).

2) Total overlapping area of lakes intersecting zone ([label]_Overlapping_Area_ha).

3) Percent of zone covered by lakes ([label]_Overlapping_Area_pct).

4) The sum of the total area of lakes intersecting the zone ([label]_Contributing_Area_ha), which can be divided by the count to get average lake size.

This tool will summarize the following subsets/groupings and label the variables accordingly:

| All Lakes ≥ 4 ha | Lakes4ha |
| --- | --- |
| All Isolated Lakes ≥ 4 ha | Lakes4ha_Isolated |
| All Headwater Lakes ≥ 4 ha | Lakes4ha_Headwater |
| All 'DRStream' Lakes ≥ 4 ha | Lakes4ha_DRStream |
| All 'DRLakeStream' Lakes ≥ 4 ha | Lakes4ha_DRLakeStream |
| All Lakes ≥ 4 ha and <10 ha | Lakes4to10ha |
| All Isolated Lakes ≥ 4 ha and <10 ha | Lakes4to10ha_Isolated |
| All Headwater Lakes ≥ 4 ha and <10 ha | Lakes4to10ha_Headwater |
| All 'DRStream' Lakes ≥ 4 ha and <10 ha | Lakes4to10ha_DRStream |
| All 'DRLakeStream Lakes ≥ 4 ha and <10 ha | Lakes4to10ha_DRLakeStream |
| All Lakes ≥ 10 ha | Lakes10ha |
| All Isolated Lakes ≥ 10 ha | Lakes10ha_Isolated |
| All Headwater Lakes ≥ 10 ha | Lakes10ha_Headwater |
| All 'DRStream' Lakes ≥ 10 ha | Lakes10ha_DRStream |
| All 'DRLakeStream' Lakes ≥ 10 ha | Lakes10ha_DRLakeStream |

#### Inputs

- **Zone feature class:** This is the polygon feature class containing the zones that you want to summarize data for.
- **Zone field:** This is the field that uniquely identifies each zone.
- **Lake feature class:** This is a feature class containing all the lake features you want to summarize by zone. It should contain all the lakes you want to describe, and no extra lakes.
- **Output table:** This is the location of the output table.

#### Output(s)

The output is a table with one record per input zone and 60 calculated fields, as described above. 0 is the smallest possible value and null values are not included in the output but are internally converted to 0 by this tool. *_AREA_ha values cannot exceed the area of the input zone. *_AREA_pct values cannot exceed 100%.

#### Warning(s)

If you try to sum all of the lake counts from this tool, you will get more than the original number of lakes for that region due to the lakes being counted as 'belonging' to more than one zone. Consequently, we recommend that you use other methods for aggregating the count of lakes, although you may sum the area values.

#### Tool steps summary

1. For each selection query listed in the left column of the table above, this tool runs the Polygons in Zones tool and renames the outputs to contain the naming convention in the right column of the table above

#### Time

This tool takes 4-8 hours to run for each zone/scale that you are interested in summarizing data for.

#### Related ArcGIS help topics

Tabulate Intersection, Spatial Join

#### Source code location

LAGOS_GIS_Toolbox/lakes_in_zones2.py

### Wetlands in Zones

#### Description

This tool is a wrapper around the Polygons in Zones tool that summarizes wetland data using all of the subsets/groupings that we were interested in exploring for LAGOS. For each grouping, this tool calculates the following four values that are the same as in the Polygons in Zones tool:

1) Number of wetlands intersecting zone ([label]_Count).

2) Total overlapping area of wetlands intersecting zone ([label]_Overlapping_Area_ha).

3) Percent of zone covered by wetlands ([label]_Overlapping_Area_pct).

4) The sum of the total area of wetlands intersecting the zone ([label]_Contributing_Area_ha), which can be divided by the count to get average wetland size.

This tool will summarize the following subsets/groupings and label the variables accordingly:

| All wetlands, no dissolve (as they came from NWI) | AllWetlandsUndissolved |
| --- | --- |
| Isolated wetlands* (order) | IsolatedWetlandsUndissolved |
| Wetland with a 'single' connection* (order) | SingleWetlandsUndissolved |
| 'Connected' wetlands* (order) | ConnectedWetlandsUndissolved |
| Forested wetlands* (vegetation type) | ForestedWetlandsUndissolved |
| Scrub-shrub wetlands* (vegetation type) | ScrubShrubWetlandsUndissolved |
| Open water wetlands* (vegetation type) | OpenWaterWetlandsUndissolved |
| Wetlands with regime code* 'A' | RegimeAWetlandsUndissolved |
| Wetlands with regime code* 'C' | RegimeCWetlandsUndissolved |
| Wetlands with regime code* 'F' | RegimeFWetlandsUndissolved |
| Wetlands with regime code* 'G' | RegimeGWetlandsUndissolved |
| Wetlands with regime code* 'H' | RegimeHWetlandsUndissolved |
| All wetlands, dissolved, i.e. bordering wetlands dissolved into single wetland | AllWetlandsDissolved |

*None of the subset statistics are made with dissolved wetland polygons

#### Inputs

- **Zone feature class:** This is the polygon feature class containing the zones that you want to summarize data for.
- **Zone field:** This is the field that uniquely identifies each zone.
- **Wetlands feature class:** This is a feature class containing all of the wetland features that you want to summarize by zone. It should contain all of the wetlands you want to describe and it should contain no extra wetlands.
- **Output table:** This is the location of the output table.
- **Summarize dissolved wetlands (checkbox):** When checked, the 'all wetlands' statistics will be performed with both undissolved and dissolved wetlands

#### Output(s)

The output is a table with one record per input zone and 52 calculated fields, as described above. 0 is the smallest possible value and null values are not included in the output but are internally converted to 0 by this tool. *_AREA_ha values cannot exceed the area of the input zone. *_AREA_pct values cannot exceed 100%.

#### Warning(s)

If you try to sum all of the wetland counts from this tool, you will get more than the original number of wetlands for that region due to the wetlands being counted as 'belonging' to more than one zone. We recommend that you use other methods for aggregating the count of lakes, although you may sum the area values.

#### Tool steps summary

1. For each selection query listed in the left-hand column of the table above, this tool runs the Polygons in Zones tool and renames the outputs to contain the naming convention in the right-hand column of the table above.

#### Time

This tool takes 15-20 hours to run for each zone/scale that you are interested in summarizing data for.

#### Related ArcGIS help topics

Tabulate Intersection, Spatial Join

#### Source code location

LAGOS_GIS_Toolbox/wetlands_in_zones2.py

### Streams in Zones

#### Description

This tool is a wrapper around Line Density that summarizes the stream data for a variety of subsets/groupings that we were interested in exploring for LAGOS. For each grouping, this tool calculates the following two values that are the same as in the Line Density tool:

1) Total length of streams in the zone (meters) ('[label]_SUM_LengthM').

2) Density of streams in the zone (meters per hectare) ('[label]_Density_MperHA').

This tool will summarize the following subsets/groupings and label the variables accordingly:

| All streams | Streams |
| --- | --- |
| Headwater streams (Strahler # 1-3) | Headwaters |
| Midreach streams (Strahler # 4-6) | Midreaches |
| Rivers (Strahler # 7-9) | Rivers |


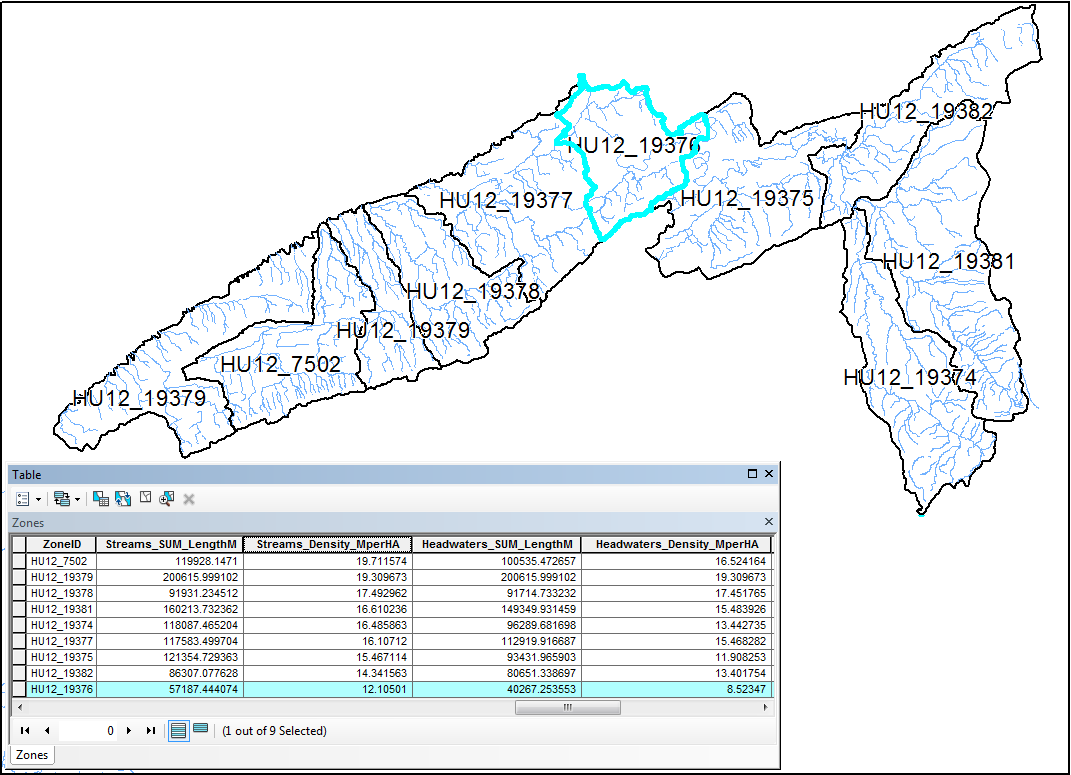


**Figure 4. Zone 'HU12_19376' has the lowest overall stream density.**

#### Inputs

- **Zone feature class:** This is the polygon feature class containing the zones that you want to summarize data for.
- **Zone field:** This is the field that uniquely identifies each zone.
- **Streams feature class:** This is the line feature class containing the streams data that you want to summarize by zone. It should contain all of the streams you want to describe and it should contain no extra streams.
- **Output table:** This is the location of the output table

#### Output(s)

The output is a table with one record per input zone and eight calculated fields, as described above. 0 is the smallest possible value and null values are not included in the output but are internally converted to 0 by this tool.

#### Warning(s)

This tool assumes that the line data has an extent greater than or equal to the zones data. If this is not true, then the user should create a workflow to calculate the fraction of the zones with line data and then use that fraction to adjust the values resulting from this tool.

#### Tool steps summary

1. For each selection query listed in the left-hand column of the table above, this tool runs the LineDensity tool and renames the outputs to contain the naming convention in the right-hand column of the table above.

#### Related ArcGIS help topics

Identity, Statistics

#### Source code location

LAGOS_GIS_Toolbox/streams_in_zones.py

### Zonal Attribution of Raster Data

#### Description

This tool calculates summary raster statistics for each zone.

When used with thematic raster data (categorical data such as land cover class), the user will check the box asking "Thematic dataset?" This tool will then calculate a series of values prefixed with Ha_ or Pct_ followed by the class name/value for each class represented in the thematic dataset that represent the area and percentage of the zone covered by that class in the zone.

When used with numeric (continuous or interval/ratio) raster data, the user will uncheck the box asking "Thematic dataset?" and the tool will then calculate Min, Max, Mean, and Std for each zone.


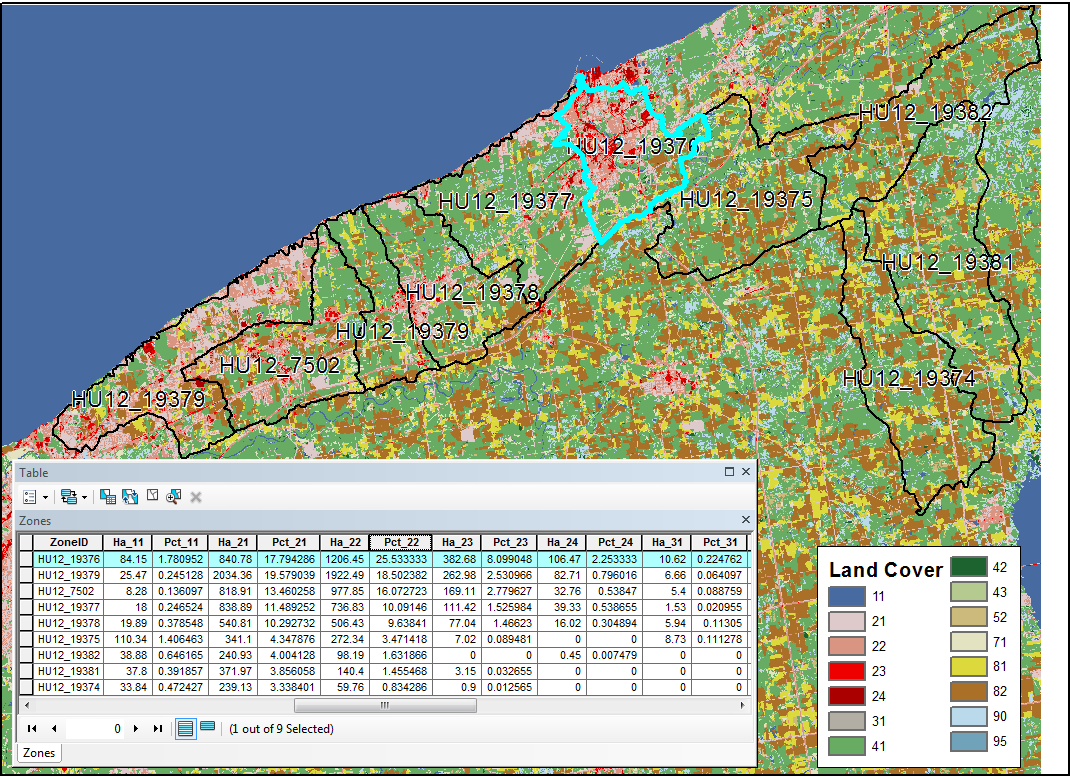


**Figure 5. Land Cover (NLCD 2006) summarized by zones.** Notice that the land cover classes for Developed (21,22,23,24) have very high values in HU12_19376, also represented on the map.

#### Inputs

- **Zone feature class:** This is the polygon feature class containing the zones that you want to summarize data for.
- **Zone field:** This is the field that uniquely identifies each zone.
- **Input value raster:** This is the raster dataset containing the raster data you want to summarize by zone (e.g., land cover, mean precipitation, total nitrogen deposition, topographic index, etc.).
- **Categorical raster dataset?:** Check this box if the raster data are categorical/classified data. Leave it unchecked for numeric data (continuous or interval/ratio).
- **Output table:** This is the output table that will contain the summary of the values in each zone.

#### Output(s)

The output is a table with one record per input zone and the calculated fields as described above. Occasionally, when the zones are small and the raster is coarse, some records will have missing data represented by Null values. The field descriptions are as follows (adapted from ArcGIS 10.1 help):

Variety: Calculates the number of unique values for all cells in the value raster that belong to this zone (For Thematic Data Only).

Majority: Determines the value that occurs most often of all cells in the value raster that belong to this zone (For Thematic Data Only).

Minority: Determines the value that occurs least often of all cells in the value raster that belong to this zone (For Thematic Data Only).

Median: Determines the median value of all cells in the value raster that belong to this zone (For Thematic Data Only).

Min: Determines the smallest value of all cells in the value raster that belong to this zone (For Continuous/Interval/Ratio Data Only).

Max: Determines the largest value of all cells in the value raster that belong to this zone (For Continuous/Interval/Ratio Data Only).

Mean: Calculates the average of all cells in the value raster that belong to this zone (For Continuous/Interval/Ratio Data Only).
Std: Calculates the standard deviation of all cells in the value raster that belong to this zone (For Continuous/Interval/Ratio Data Only).

Ha_*: Calculates the total area of all cell of class * in the value raster that belong to this zone.

Pct_*: Ha_* divided by total area of all cells in the value raster that belong to this zone.

#### Warning(s)

This tool assumes that the zones are completely covered by raster data. If there are missing data in the raster dataset within the zone polygons, then you may need to do additional work to describe how this affects the output. Missing data might be indicated if a very large number of features are listed in the warning message about missing features.

This tool makes a substantially different output based on whether or not 'Thematic raster dataset?' is checked. Be sure that the value of the check box is appropriate for your raster data type.

The values in the Ha_* fields are calculated from the raster-based area covered by that class. When all of the class areas are summed, they may equal slightly more or less than the vector-based area described in the zones feature class (typically +- 3% of the total area). If this is a problem for your application, then multiply the Pct_* fields by the vector-based area to get class areas that will add up to the vector-based area.

Rarely, if ArcGIS does not recognize the NoData value used by a continuous raster, then these values may erroneously be used in the calculation of the zonal statistics. This can be corrected by updating the NoData property of the raster in ArcCatalog (see the ArcGIS 10.1 Help article 'NoData in raster datasets').

Interpretation of field names must be done with care for some variables that themselves describe something like a minimum or maximum or mean. For instance, the minimum value within each zone of the PRISM 30-year normal mean temperature raster might be called something like 'HU12_prism_30yr_normal_tmean_min', and is the lowest 'mean temperature' for each HU12. The mean value within each zone of the PRISM 30-year normal minimum temperature raster might be called something like 'HU12_prism_30yr_normal_tmin_mean' and is the average of the 'minimum temperature' cell values for each HU12. The output data documentation should take care to describe these fields precisely.

Caution is recommended when using this zonal attribution tool for raster data with very small zones and very coarse raster data because it is possible that some zones will not be attributed with any raster data owing to the scale of the analysis. Hence, it is preferable to calculate percent raster data after export from the toolbox, especially for smaller zones.

The Ha_* and Pct_* fields will usually use the raster values (typically integers) to describe each class. The users will need to refer to the raster legend or metadata to figure out what the names of these classes are. It is best to write up this information in a codebook or in the database documentation.

#### Tool steps summary

1. Run ArcGIS Zonal Statistics as As Table tool. As a part of this function, the zones are converted to a raster with the same grid alignment and cell size as the input data.
2. If the 'Thematic raster dataset?' box was checked, then run Tabulate Area with the same cell size as the input raster.
3. Calculate the percent of the total area occupied by each class by dividing the class area by the total area of the zone reported by Tabulate Area (this is the raster-based area of the zone).
4. If there are zones with null data, then it is because the zone was close to the same size as one cell in the raster data, or smaller, and was eliminated in the internal vector-to-raster conversion of the Zonal Statistics As Table. The output will be created and an ArcGIS warning will be issued to alert the analyst that some zones were not attributed.

#### Time

The time that this tool takes to run depends mainly on the resolution of the raster (a higher resolution takes longer) as well as on the zones input.

#### Related ArcGIS help topics

Zonal Statistics As Table, Tabulate Area

#### Source code location

LAGOS_GIS_Toolbox/zonal_tabarea.py

### Subset Overlapping Zones

#### Description

This tool uses a graph-coloring algorithm to efficiently split a layer with overlapping polygons into multiple layers, each of which has no overlap. The output of this tool can then be used in the Zonal Attribution of Raster Data for Overlapping Zones tool, which will calculate each layer separately and merge the results so that each zone is correctly attributed in the end.

Overlapping zones, such as inter-lake watersheds, cannot be used as an input to the Zonal Attribution of Raster Data tool because the zones are internally converted to a raster, which cannot have more than one value for each cell. The outputs of this tool can be re-used with all of the raster data that you wish to summarize.

#### Inputs

- **Zones feature class with overlaps:** This is a polygon feature class with overlapping features, such as Interlake Watersheds (IWS) or lake (polygon) buffers.
- **Zone field:** This is the field that uniquely identifies each zone.
- **Output geodatabase:** This is the geodatabase that will store the multiple feature classes output from this tool. It is suggested that the user should create a new geodatabase.

#### Output(s)

The outputs include multiple polygon feature classes in a geodatabase with the name of the input feature class plus '_NoOverlap' and a unique number at the end (starting with no number, then 0, 1, 2, 3….). The number of polygon feature class layers will be at least as many as the largest number of polygons overlapping in one spot in the input feature class, but may be more. The total number of features among the multiple layers should be equal to the number of features in the input feature class.

#### Warnings

This tool will not necessarily make the same subsets each time that it is run. However, together, they will always add up to the original dataset, but a 'FeatureClass_NoOverlap1' of one run cannot be swapped out for 'FeatureClass_NoOverlap1' of another run.

#### Tool steps summary

The majority of this script was written by the ESRI Spatial Analyst team for 1.3 release of 'Spatial Analyst Supplemental Tools' (http://blogs.esri.com/esri/arcgis/2013/11/26/new-spatial-analyst-supplemental-tools-v1-3/). It has been modified for the LAGOS GIS Toolbox to save the non-overlapping layers to disk.

1. Dissolve features on the zone field.
2. Identify polygon overlaps.
3. 'Color' polygons (nodes): each polygon gets a 'color' not among the colors of its neighbors.
4. Classify polygons (nodes) by 'color'.
5. Save each color class to a new feature class in the output geodatabase, ensuring that each feature class has a unique name.

#### Time

On a feature class (of interlake watersheds) with 51,111 features split into 16 output layers, this tool took 20 minutes to run. It is a little faster for less complicated feature classes with fewer features.

#### Related ArcGIS help topics

Polygon Neighbors

#### Source code location

LAGOS_GIS_Toolbox/color_polygons.py

### Zonal Attribution of Raster Data for Overlapping Zones

#### Description

This tool is used with the multiple outputs of Subset Overlapping Zones to create a single table with summary raster statistics for all zones. See the description of Zonal Attribution of Raster Data for more information as the only substantial difference between these tools is the nature of the zones input.

#### Inputs

- **ALL 'NoOverlap' subset feature classes:** All of the layers output from Subset Overlapping Zones should be input here using the '+' button. The order does not matter.
- **Zone field:** This is the field that uniquely identifies each zone. There is no dropdown menu so the user will have to note the zone field name common to the 'NoOverlap' subsets and type it here.
- **Input value raster:** This is the raster dataset containing the raster data that you want to summarize by zone (e.g., land cover, mean precipitation, total nitrogen deposition, topographic index, etc.).
- **Categorical raster dataset?:** Check this box if the raster data are categorical/classified data. Leave it unchecked for numeric data (continuous or interval/ratio).
- **Output table:** The output table will contain the summary of the values in each zone

#### Output(s)

The output is a table with one record per input zone and calculated fields, as described in the documentation for Zonal Attribution for Raster Data. Occasionally, when the zones are small and the raster is coarse, some records will have missing data represented by Null values.

#### Warning(s)

See the warnings for the Zonal Attribution of Raster Data.

#### Tool steps summary

1. For each of the NoOverlap subsets, run the Zonal Attribution for Raster Data tool and save the result to a temporary table.
2. Merge the temporary tables into a single table.

#### Time

This tool takes a very long time to run.

#### Related ArcGIS help subjects

Zonal Statistics as Table, Tabulate Area

#### Source code location

LAGOS_GIS_Toolbox/overlap_stats.py

## Watershed Delineation

### Overview

We created the watershed using publicly accessible data for the 17 states in our study's extent. We used a custom toolbox to mosaic data to subregions, burn in streams, remove pits, bound watersheds within subregions, calculate flow direction, generate seeds, and calculate watersheds, which were then cleaned and aggregated. The final output after running all of the tools in this toolbox will be a single feature class of aggregated watersheds within the entire study area. Because of the design of the aggregation, this feature class will have overlapping polygons, which is an important detail to remember when using this feature class as the input zones to any future processing. Several of the intermediate coverages are also useful afterwards, such as the subregion mosaic. To process such a large extent in a reasonable amount of time, our process used a high-performance computing cluster (whose details will also be described in this document).

The major steps in the watershed delineation process are as follows:

1. Obtain and mosaic NED DEMs, NHD data. Introduce 'walls' that prevent flow from crossing HU8 boundaries. Data is projected here to USGS Albers coordinate system, and later as necessary.
2. DEM conditioning: Burn NHD flowlines (streams, and more*) into DEM using AGREE-like conditioning. This step ensures that flow accumulates into cells that correspond with the NHD flowlines.
3. DEM conditioning: Fill pits in the DEM. This step prevents flow from accumulating in isolated pits (Jenson and Domingue 1988).
4. Force the flow to not cross HU8 boundaries by adding 'walls' along the boundary.
5. Calculate D8 flow direction from the DEM.
6. Select pour points: All NHD flowlines and lakes meeting the size threshold and being of certain FCodes (non-intermittent lakes and reservoirs) are included. Lakes take precedence where lakes and flowlines (in particular, artificial paths through lakes) overlap.
7. Calculate watersheds from the flow direction rasters using the selected pour points.
8. Remove watersheds smaller than 1ha and those that do not intersect the NHD flowlines or selected lakes. Use a 'nibble' algorithm to partition the areas where watersheds were removed into the retained watersheds.
9. Ensure that watershed polygons do not cross HU8 boundaries and that the lakes are perfectly nested within their own watersheds.
10. Aggregate watersheds. An aggregated watershed is created for each eligible lake in the NHD. The lake itself is erased from the watershed polygon and any remaining holes smaller than 3.9 ha are filled in.
    1. 'Interlake watersheds': All of the watersheds corresponding to network elements (lakes/streams) upstream of any eligible lake are merged until a 10-hectare lake is reached in the network, and no watersheds including and beyond the 10-hectare lake are included.
    2. 'Cumulative watersheds': All watersheds corresponding to network elements (lakes/streams) upstream of any eligible lake are merged until the HU8 boundary prevents any additional watersheds from being included. The lake itself is erased from the watershed polygon and any remaining holes smaller than 3.9 ha are filled in.

*The NHD flowline feature class is a complete line-based hydrological network that includes the following seven major categories: stream/river, canal/ditch, connector ("a known, but non-specific connection between two non-adjacent network segments that have flow"), artificial path ("represents flow through a 2-dimensional feature, such as a lake or double-banked stream"), coastline, pipeline, and underground conduit.

### Scope

The geographic extent of the analysis performed was the watershed subregions comprising a 17 state region (i.e., Connecticut, Illinois, Indiana, Iowa, Maine, Massachusetts, Michigan, Minnesota, Missouri, New Hampshire, New Jersey, New York, Ohio, Pennsylvania, Rhode Island, Vermont, and Wisconsin).

### Data sources

The following are the data sources used in the watershed delineation tools.

#### National Hydrography Dataset

For a complete description of the NHD data used in the LAGOS database, see the NHD documentation.

Use of the NHD in this toolbox:

The toolbox uses the WBD_HU4 (polygon), WBD_HU8 (polygon), NHDWaterbody (polygon), and NHDFlowline (polyline) feature classes in the NHD geodatabase. It also uses the HYDRO_NET hydrological network, which also makes use of the HYDRO_NET_Junctions point feature class.

Our base areal unit for analysis is the area defined by NHD HU4 Subregions. It is desirable to use these as the base areal unit for the following reasons:

1. The 24k NHD datasets are distributed by Subregions.
2. Most forms of computational analysis can be practical at this extent.
3. There should be significant situational correlations between water features that share a subregion.
4. Subregions can be easily aggregated to regions or disaggregated to smaller units such as HU8, 10, 12, if desired.

#### Watershed Boundary Dataset

The analyst should obtain a version of the seamless 4-digit Watershed Boundary Dataset (WBD) available at ftp://ftp.ftw.nrcs.usda.gov/wbd/ that matches the version of the NHD that they are using. A subset of the HU4 subregions to be included in processing can be created by performing a spatial query in which the output is the intersection of the WBD and the study area boundary. This output will be used to determine which NED tiles need to be obtained in the next step.

#### National Elevation Dataset

The USGS maintains the NED in the public domain. The NED is a digital raster dataset with the best-available elevation data for US (1/3 arc-second in most areas, but with resolution as fine as 1/9 arc-second in limited regions and as coarse as 2 arc-second in Alaska). The data are delivered with a geographic coordinate system using the North American Datum of 1983 (NAD83) and can be downloaded as 'tiles' of size one degree latitude, one degree longitude. The USGS periodically releases updates to the NED.

The user does not need to unzip the NED data, the first tool in the toolbox can do this.

**National Map Viewer**

[**http://www.nationalmap.gov**](http://www.nationalmap.gov)

**Bulk download** <http://cumulus.cr.usgs.gov/webappcontent/neddownloadtool/NEDDownloadToolDMS.html>

If you are seeking just a few specific tiles or your region is small enough to be covered by only a few tiles, then you can use the 'Download Data' tool of the National Map Viewer to select your region of interest and order the data. However, bulk downloads are not available this way (a warning will be issued if your region is too large and you will not be able to select 'US Topo' data), so for a study of this size we recommend determining the bounding coordinates (in decimal degrees) of your study area and obtaining a single order using the bulk download method. You can also download tiles by State, but for a multi-State region you will download many duplicates this way. After inputting the bounding coordinates on this page, look for the 'National Elevation Dataset (1/3 arc-second)' results part-way down the resulting page, select 'ArcGrid' from the drop-down menu of format choices, and download all of the links (a download manager for your browser, such as DownThemAll, may be helpful here).

To determine which tiles to download or confirm that all of the necessary tiles have been downloaded, you will need a boundary file for your study region (for ours, the 17 states), the 4-digit WBD. Select the HU4 features that intersect your study region (using Select By Location in ArcGIS) and then select the grid cells that overlap the selected HU4 features. The values of the FILE_ID column will be a list of the files that you will need.

### Suggested Folder Structure

**INPUTS**: This is a folder with all of the NHDs (unzipped) and a folder with all the NEDs (all unzipped), a footprints file, and a WBD file

**OUTPUTS:**

NHD0101

----NHDH0101.gdb

----NED13_0101.tif

----Burnt_0101.tif

----clipped_0101

--------NED01010101.tif

--------(and so on)

----filled_0101

--------NED01010101fel.tif

--------(and so on)

----walled_0101

--------NED01010101fel.tif

--------(and so on)

----flowdir_0101

At the point where the watersheds are being created, it is easier to switch to one geodatabase for all the outputs, but you can make a geodatabase in each subregion's folder if you prefer.

### Tool Order

The tools should be used in the following order:

1. Download files
2. Stage and Mosaic
3. Generate Seeds (at any point after step 2 and before step 14)
4. Burn Streams
5. Clip to HU8 boundaries
6. (Upload to HPCC)
7. Fill pits
8. (Download to workstation)
9. Establish walls at HU8 boundaries
10. (Upload to HPCC)
11. Flow direction
12. (Download to workstation)
13. Convert to ESRI Raster
14. Watershed tool
15. Create Watersheds
16. Interlake Watersheds or Cumulative Watersheds
17. Merge Watersheds

### Stage and Mosaic Data for the Subregion

#### Description

The zipped NED tiles and NHD geodatabases that are arranged in single-theme directories are combined and processed so that each NHD subregion (HU4) has a directory with its NHD geodatabase and DEM mosaic inside. Any missing tiles will be discovered and a warning will be issued to the user when the tool exits. Once this tool is successfully run all the way through with no warnings or errors, the analyst can run the next tool, 'Burn streams'. All of the outputs from the following tools will be arranged in the subregion-based directory created in this tool.

#### Inputs

- **NHD geodatabase for the subregion to process:** This is the NHD geodatabase for the subregion (HU4) that you want to process. This file will have a name like NHDH0101.gdb. See Data Sources for more details.
- **NED Directory:** This is a directory that contains all of the NED tiles that could be needed for your study area. This tool will select the files needed for this subregion and copy them. If there are missing files, the tool will fail with a message indicating the tile that the user should acquire before running the tool again.
- **NED Footprints:** This is the NED footprints feature class (a vector file with a rectangular grid indicating the extent of each NED tile). See Data Sources, NED, for more details.
- **Output directory for this subregion:** This is an umbrella directory for the outputs from this tool. A directory will be created for this subregion, with a name like NHD0101, inside the umbrella directory.
- **Zipped?:** This tool can quickly unzip NED data as needed to allow the user to store the original data unzipped to save space. If you have already unzipped your NED data, uncheck this box.
- **Available RAM (in GB):** This is optional. This tool will run much faster for most subregions if your workstation has a lot of RAM. Type an integer here indicating how much RAM is available to ArcGIS (it should be a little less than the amount of physical RAM on your machine). For instance, if your workstation has 16 GB of RAM, try typing 14 here.

#### ***Output(s)***

The output of this tool is a folder with the name NHDxxxx inside the umbrella directory indicated by the user. Inside the NHDxxxx folder will be the NHDHxxxx.gdb and a mosaic raster in the USGS Alber projection with 10 m resolution, named 'NED13_xxxx.tif' (along with its accompanying files) that is clipped to the boundary of a 5 km buffer around the subregion. Further use of the NHD geodatabase should point to the copy created in this tool. The mosaic will be used at multiple points during the delineation process and can also be used for other purposes; for example, to generate terrain derivatives like slope or terrain ruggedness index.


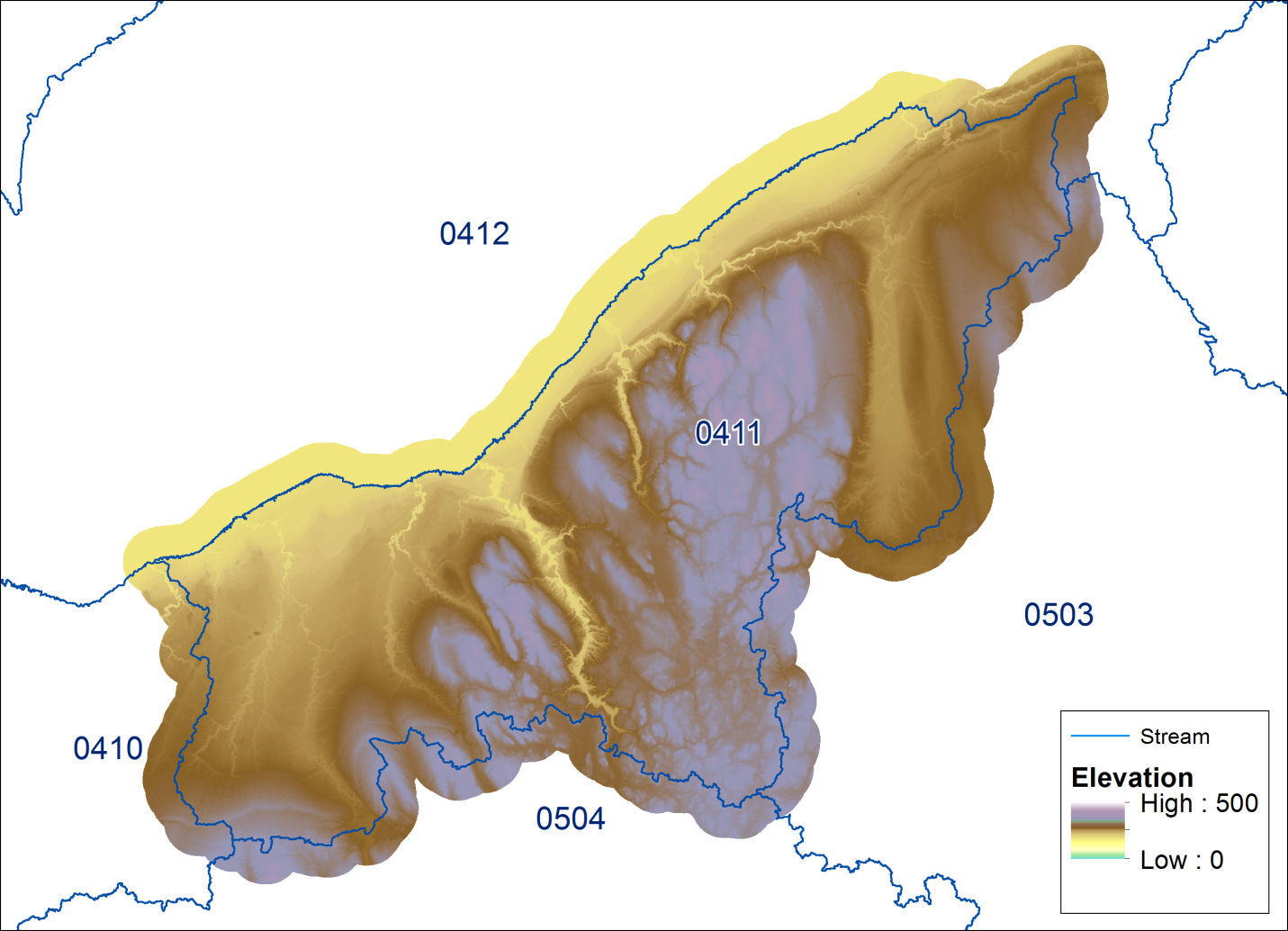


**Figure 6. Output of stage and mosaic data for subregion.**

#### Warning(s)

#### Tool steps summary

##### Stage files

1. Determine the subregion HU4 code from the input file names.
2. Create the output directory for this subregion.
3. Copy the NHD geodatabase to the output directory for this subregion that was just made.
4. Select this subregion from the WBD_HU4 dataset in the NHD geodatabase and make it a feature layer.
5. Create a layer that is the subregion with a 5000 m buffer.
6. Select the NED footprints that intersect the buffered subregion.
7. For each value of the 'FILE_ID' field of the clipped footprints layer (the list of NED tiles we need), find the corresponding NED tile folder/file in the NED directory. Issue a warning if it does not exist.
8. If the NED is zipped, unzip it to the output directory. If not, simply copy the folder.

##### Mosaic

1. Set up initial processing environment: outputCoordinateSystem = our projection (USGS Albers).
2. Determine the subregion HU4 code from the input file names.
3. Select this subregion from the WBD HU4 dataset in the NHD geodatabase and make it a feature layer.
4. Create a layer that is the subregion with a 5000 m buffer.
5. Find all the NED tile rasters that were staged.
6. Mosaic the NED Tiles using their native projection.
7. Project the NED mosaic using bilinear interpolation and an output cell size of 10 m exactly.
8. Clip the projected mosaic to the shape of the subregion buffer.

#### QA/QC needed

-If the tool exits with the warning that an NED is missing, then the user should download the NED tile into the NED directory and try the tool again, unless there is a valid reason for the NED tile to be missing.

-The output mosaic should be visually checked to confirm that it covers the entirety of the subregion as long as NED data are available (see Data Sources, NED for a description of how we handled border regions). Occasional problems with unzipping NED tiles or other bad data can occur. Subsequent tools will usually complete and create output regardless of whether the mosaic is complete, so skipping this very important check now can result in undetected problems or considerable time spent re-processing when the error finally becomes obvious.

#### Error analysis

This tool does not introduce any new information. Any error present in the output was already present in the inputs (DEM error).

#### ***Disk space required***

In our 17 state region, the two largest outputs were the 0415 directory (18 GB) and the 0902 directory (10 GB). The smallest output was the 0411 directory (<1 GB).

#### Related documentation subjects

Mosaic To New Raster, Project Raster

#### Source code location

LAGOS_GIS_Toolbox/NED2Subregion.py (functions: stage_files, mosaic, unzip_ned, delete_neds)

### Burn Flowlines into DEM

#### Description

The flow network of the original DEM and the flow network of the NHD do not necessarily agree due to horizontal error, resolution, or digitizing differences. In this step, the DEM is forced to agree with the hydrologic network identified in the NHD by lowering the elevation values of cells coinciding with the vector flowlines in the NHD by 10 m. Flow is forced into the lowered cells. In addition to 'burning in' the flowlines for the cells that they coincide with, we lowered elevations within 500 m of streams using a beveled surface that lowers the cells closer to the flowline and tapers off as the distance from the flowline increases. The output of this tool is ready to be clipped to HU8 boundaries before using the pit remove tool.

#### Inputs

- **Subregion DEM:** This is a DEM covering the subregion that you want to process (the same as in NHD Geodatabase). Most likely, this will be **the mosaicked NED raster created in Stage and Mosaic files.**
- **NHD geodatabase for the subregion to process: This is the NHD geodatabase for the subregion (HU4) that you want to process. We recommend using the copy created in Stage and Mosaic files rather than the original.**
- **Output raster: This is the location of the output raster. We recommend saving this in the directory created in Step 1 of the watersheds toolbox.**

#### Output(s)

The output is a raster with the projection, extent, and cell size of the input DEM, but with the elevation values lowered in the neighborhood of the NHDFlowline lines.


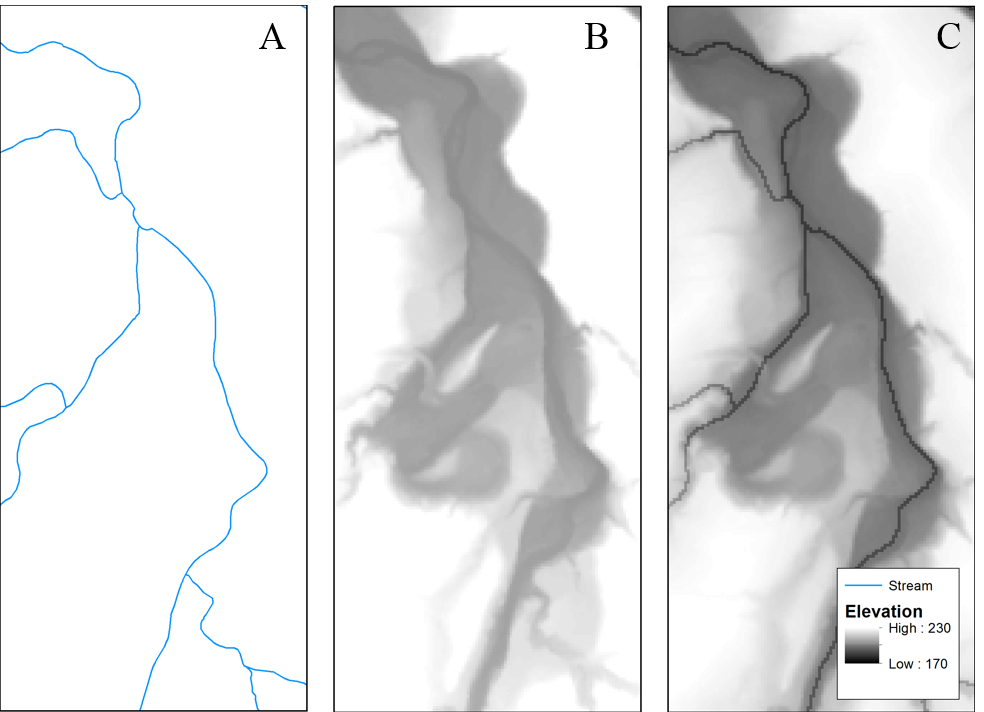


**Figure 7. a) Streams for burning, b) elevation raster before burning, and c) elevation raster after burning.**

#### Warning(s)

#### Tool steps summary

1. Rasterize NHDFlowline using the same cell size and 'snap' (alignment) as the DEM.
2. Reclassify the flowline raster so that stream cells are 1 and non-stream cells are 0.
3. Burn in the streams 10 m below the surrounding surface, beveling in from 500 m:


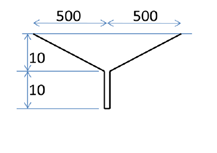
burnt = Raster(subregion_ned) - (10 * streams) - (0.02 * (500 - distance) * (distance < 500))

Figure 8. Visual of the burning equation.

#### QA/QC needed

When zoomed out, the output raster will look superficially the same as the input raster; however, when zoomed in to the point where you can almost see individual pixels, the lower values in the neighborhood of streams and other flowlines should be apparent and should be exactly 10 m directly underneath the line followed by a feature in NHDFlowline. As long as the input raster covers the complete area needed and NHDFlowline has not been corrupted somehow, the output of this tool is almost always okay (as long as it completes).

#### Error analysis

This may not resolve all of the conflict between the DEM and the flowlines in the NHD.

Can still suffer from problems with parallel streams.

#### Disk space required

The output raster will be the same size as the input raster (space needed will roughly double).

#### Related documentation subjects

Feature to Raster, Spatial Analyst: Euclidean Distance, Reclassify, Raster Calculator

#### Source code location

LAGOS_GIS_Toolbox/burn_streams.py (function(s): burn)

### Clip DEM to HU8 Boundaries

#### Description

After burning in the flowlines, the next step is to remove pits from the DEM. However, the TauDEM tools can only accept uncompressed GeoTIFFs that are smaller than 4 GB as inputs. To stay within this size limit, we clip the DEMs to HU8 boundaries because we ultimately coerce the flow to not cross the HU8 boundary and also because the resulting rasters are a good size for processing. A buffer of 5 km is used because we will add a wall at the HU8 boundary later and also because the buffer prevents loss of flow information along the edges of the raster. The output of this tool is ready for file transfer, if necessary, and then for the Pit Remove tool.

#### Inputs

- **Subregion DEM with burned streams:** This is a burned DEM covering the subregion that you want to process (the same as in the NHD Geodatabase). Use the output from Burn Flowlines into DEM.
- **NHD geodatabase for this subregion: This is the NHD geodatabase for the subregion (HU4) that you want to process. We recommend using the copy created in Stage and Mosaic files rather than the original.**
- **Output directory for this subregion: This is the NHDxxxx folder for the subregion that you want to process. A folder named 'huc8clipsXXXX' will be created and populated with the clipped outputs.**

#### ***Output(s)***

The output is a folder named 'huc8clipsXXXX', which will be created in the output directory and populated with multiple uncompressed TIFF rasters, each one clipped to a different HU8 within the subregion, named such as NEDXXXXXXXX.tif (where the X's are the 8-digit HU code). This raster is compatible with the TauDEM 5.2 tools.


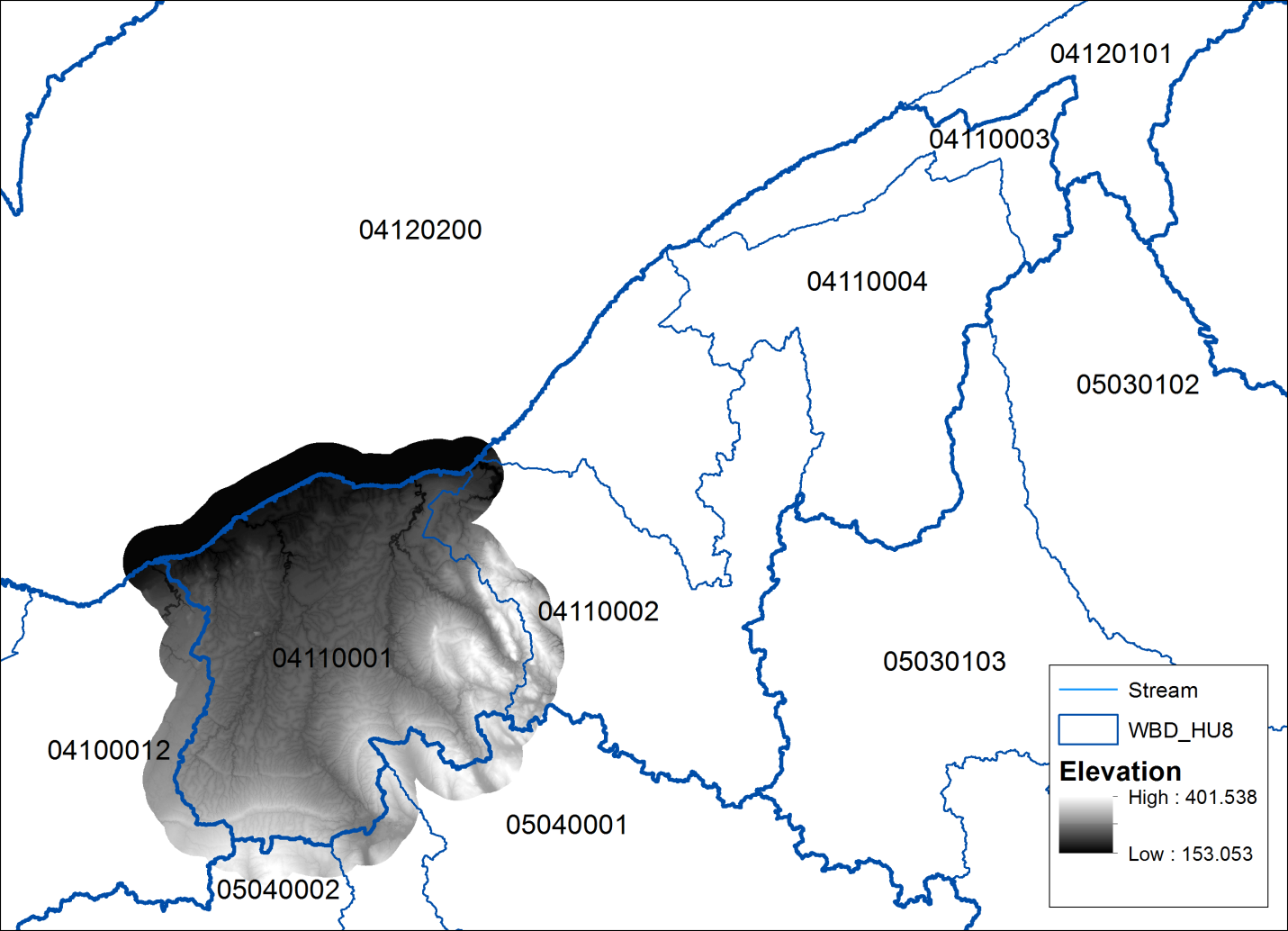


**Figure 9. Result of clipping to HU8 04110001.**

#### Tool steps summary

1. Create a feature layer from the 'WBD_HU8' layer in the geodatabase.
2. Find the HU4 in the geodatabase name.
3. Create a directory called 'huc8clipsxxxx' (xxxx is the HU4) in the output folder.
4. For each HU8 that is in this HU4, clip the input raster to the boundary of the HU8 feature (this raster will be written out to hard disk with no compression because TauDEM tools cannot use compressed TIFFs).

#### QA/QC needed

Confirm that a file is present for each HU8 and visually inspect the file to be sure that the entire HU8 is covered with terrain data. If all of the HU8 clips are loaded into a map, then they should cover the entire HU4/subregion.

#### ***Error analysis***

There is no error analysis to perform.

#### Disk space required

The amount of disk space will increase by at least the size of the input burned raster but more space will be required because the clipped TIFs are uncompressed.

#### Related documentation subjects

Clip (Raster)

#### Source code location

LAGOS_GIS_Toolbox/clip_to_hu8.py

### File transfer to high-performance computing cluster (1)

#### Description

The next tool, Pit Remove, is computationally intensive. If the users are moving to a computing cluster or other high-performance environment, then they must transfer data now.

We used the MSU HPCC resources to run the tool. Before we could do so, we had to transfer the HU8-clipped rasters to the file storage space on the HPCC. We used the Globus for Researchers file transfer tool to quickly and safely move our files between the local workstation and the HPCC file storage space (find out more about this option at: http://www.globus.org).

### Pit Remove (DEM Conditioning) – TauDEM/HPCC

#### Description

We use the TauDEM 'Pit Remove' tool out-of-the-box to remove pit/sinks from the DEMs. Pits are assumed to be artifacts of the DEM creation. 'Real' pits are also removed in this step because we chose not to identify and preserve them. This step is necessary to prevent flow from entering the pits/sinks during the calculation and then becoming trapped there instead of continuing in a flow network.


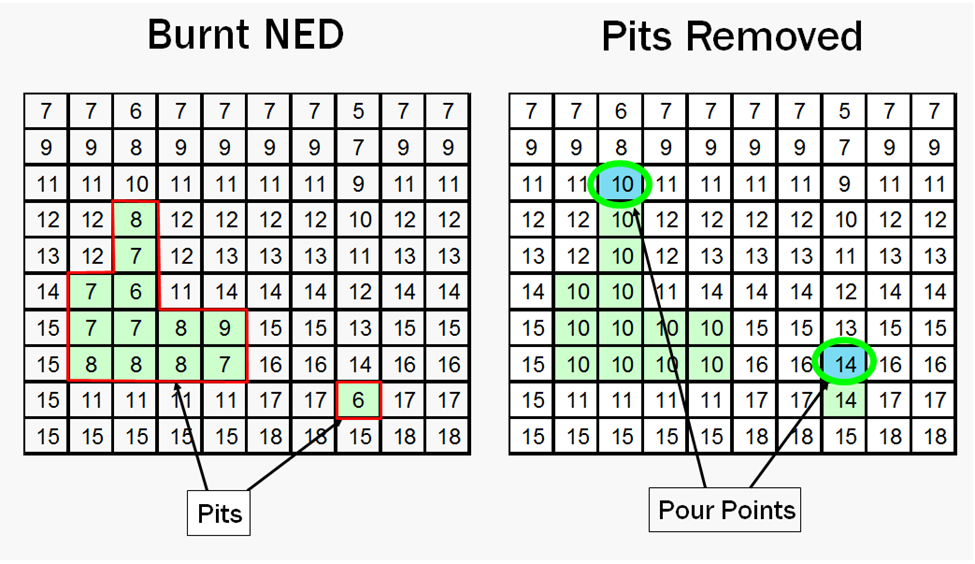


**Figure 10. How the pit fill algorithm behaves, borrowed from TauDEM (available at: http://hydrology.usu.edu/taudem/taudem5/index.html).**

#### Inputs

Each execution of the Pit Remove tool requires one of the burned and clipped DEMs that is the output of Burn Flowlines, followed by Clip DEM to HU8 Boundaries.

#### Output(s)

The output of this tool will automatically be named NED01010001fel.tif and written in the same directory as the original file. TauDEM tools use a naming convention to identify files and later tools will recognize the *fel.tif filename to mean a filled DEM. It is simplest to leave the filename alone or change it but leave the ending the same.


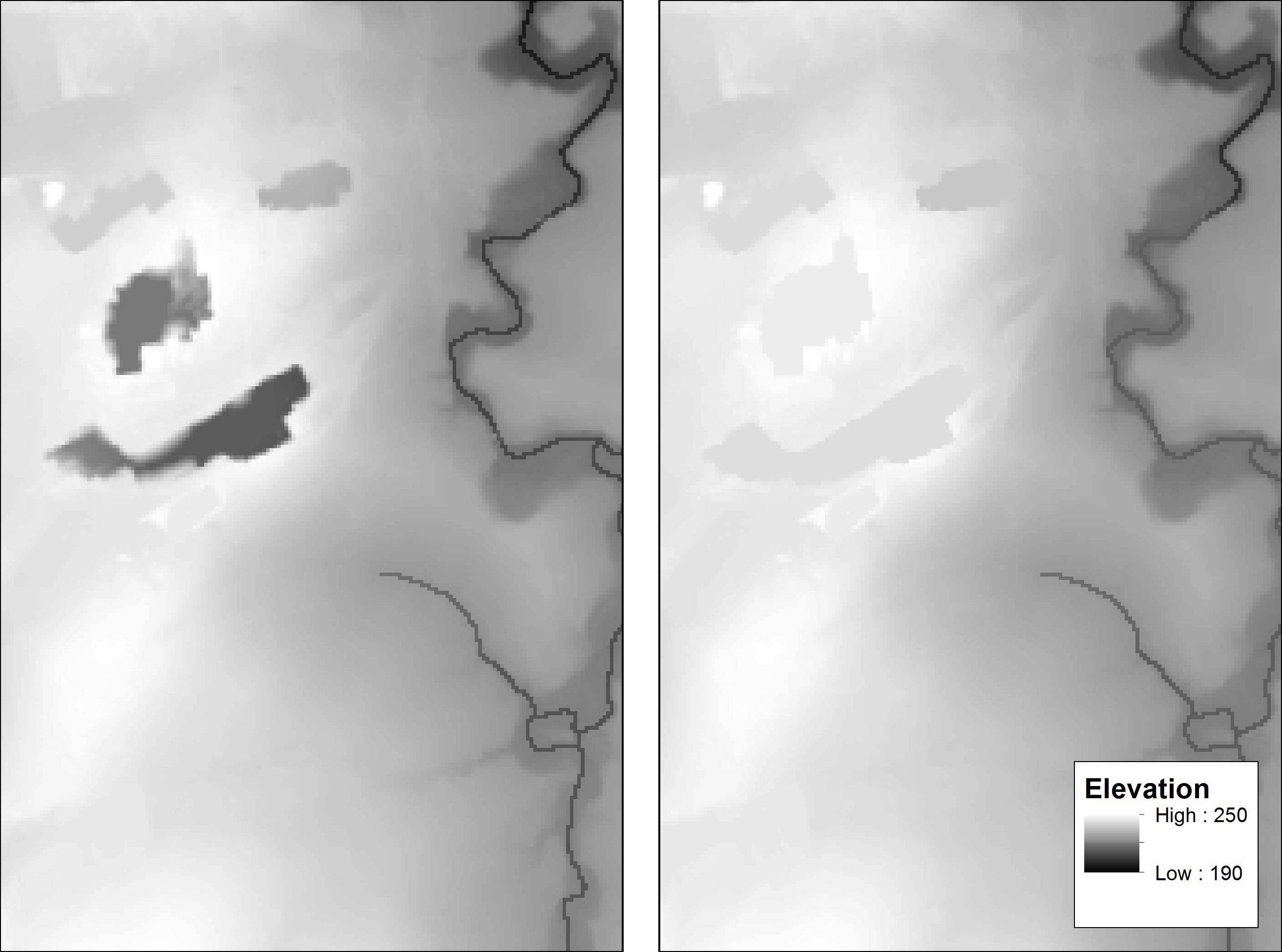


**Figure 11. Left panel: Pits before filling. Right panel: Result of filling these pits.**

#### Tool steps summary

We have included a job submission file (pit_remove.qsub) to demonstrate how we simultaneously performed multiple executions of this tool in the MSU HPCC. The file includes directions for other HPCC users telling them how to update the '#PBS', 'directory' and 'walltime' variables to re-use the file.

Running the Pit Remove tool by itself is simple. If you want to run the tool on NED01010001.tif, type 'mpiexec -n 8 pitremove NED01010001.tif' at the command line.

#### QA/QC needed

When zoomed out, the output should look substantially similar to the input. Examine at least one output compared to its input while zoomed in to understand what regions were considered pits and how they changed. Otherwise, simply verify that a *fel.tif file was created for every input *tif.

#### Error analysis

We have not evaluated what portion of the pits in the NHD are 'real' versus 'artifact', nor have we compared how using or skipping this tool affects watersheds. The error introduced is not quantified.

#### Disk space required

This tool will create an output occupying the same amount of disk space as the input.

#### Time

For most files, Pit Remove executed in under 5-10 minutes using the HPCC. Some files took up to 2 hours and, very rarely, a file can take far longer than 2 hours. Smaller rasters are typically faster on average.

#### Related documentation subjects

http://hydrology.usu.edu/taudem/taudem5/help/PitRemove.html

#### Source code location

LAGOS_GIS_Toolbox/pit_remove.qsub

#### File transfer to local workstation with ArcGIS

The files must be transferred back to the workstation with ArcGIS in order to run the Create HU8 walls tool. See the description for 'File transfer to high powered computing cluster (1)' for description and details.

### Create HU8 Walls

#### Description

The flow is coerced to avoid crossing HU8 boundaries by converting the boundary lines to a 'walls' raster with height 250 m only along the lines, and then adding the walls raster to the filled DEM (with an extent slightly beyond the HU8 boundary line because of the buffer used in Clip DEM to HU8 Boundaries). Using walls instead of simply clipping the DEMs to the HU8 boundaries is necessary because the flow direction output has a strip of NoData along the edge where the input was clipped because it could not calculate a flow direction. Using walls ensures that the flow direction is calculated for the cells all the way up to the edge of the HU8 boundary. The output of this tool is ready for transfer to the high-performance computing environment, if necessary, for the flow direction calculations.

#### Inputs

- **NHD geodatabase for the subregion to process: This is the NHD geodatabase for the subregion (HU4) that you want to process. We recommend using the copy created in Stage and Mosaic files rather than the original.**
- **Filled HU8 DEMs:** These are one or more filled, HU8-clipped DEMs for the subregion that you want to add walls to. Although we recommend doing all of the rasters for a single subregion at the same time, you can do one a time if necessary.
- **Output directory for this subregion: This is the NHDxxxx folder for the subregion that you want to process. A folder named 'walledXXXX' will be added to this folder and populated with the walled outputs.**

#### Output(s)

A folder named 'walledXXXX' will be created in the output directory and populated with multiple uncompressed TIFF rasters, one for each HU8, named such as NEDXXXXXXXXwfel.tif(where the X's are the 8-digit HU code). Each raster should have a wall along the perimeter (and a small buffer outside the wall). This raster is compatible with the TauDEM 5.2 tools.

#### ****Tool steps summary****

1. Create the walledXXXX output folder with the correct HU4.
2. Convert the HU8 polygons to polylines.
3. Add a 'height' field to the polylines.
4. Convert the polylines to a raster using a 500 (meter) 'height' as the cell value wherever there is a polyline.
5. For each filled, HU8-clipped input raster, add the walls raster (in other words, add 500 to the elevation value wherever there was a wall).

#### QA/QC needed

Examine at least one output to see that the tool is working and that the wall was added correctly at the HU8 boundary (you will need to zoom in, but it will be obvious). Otherwise, as long as an output is created for each input raster, everything should have gone okay, although you may desire to visually confirm that terrain data covers the entirety of each HU8 and that together they cover the entire subregion.

#### Error analysis

A trivial amount of error is added by forcing the flow inward at the HU8 boundary. Watersheds are forced to nest within HU8 (sub-basin) boundaries.

#### Disk space required

This tool will create an output occupying the same amount of disk space as the input for each input.

#### Related documentation subjects

Polygon to Line, Feature to Raster (conversion), Spatial Analyst: Con, Spatial Analyst: IsNull

#### Source code location

LAGOS_GIS_Toolbox/WallsHU8.py

#### File transfer to high performance computing cluster (2)

Once again, the files must be transferred to the high performance computing cluster before calculating the flow direction. See the description for 'File transfer to high powered computing cluster (1)' for description and details.

### Flow Direction

#### Description

A flow direction algorithm is used to model the movement of water through the DEM. D8 Flow Direction calculates the flow direction from each grid cell to one of its adjacent or diagonal neighbors, calculated using the direction of steepest ascent. This is the most common approach to flow direction modeling. The TauDEM direction coding is: 1 - East, 2 - Northeast, 3 - North, 4 - Northwest , 5 - West, 6 - Southwest, 7 - South, 8 - Southeast. In flat areas, the flow direction is routed according to the method described by Garbrecht and Martz (1997) [1].


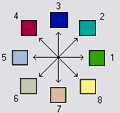


**Figure 12. Flow direction coding according to TauDEM.** Figure borrowed from TauDEM (available at: http://hydrology.usu.edu/taudem/taudem5/index.html).

#### Inputs

Use the output from Create HU8 Walls (i.e. the filled, clipped, and walled DEMs) as the input to D8 Flow Directions.

#### Tool steps summary

We have included a job submission file (flow_direction.qsub) demonstrating how we simultaneously performed multiple executions of this tool in the MSU HPCC. The file includes directions for other HPCC users telling them how to update the '#PBS', 'directory' and 'walltime' variables to re-use the file.

Running D8 Flow Direction by itself is simple: If you want to run the tool on NED01010001fel.tif, then type 'mpiexec -n 8 d8flowdir NED01010001.tif' at the command line. If you want to use optional flags to overrule the naming conventions, try 'mpiexec –n 8 d8flowdir –fel NED01010001fel.tif –p NED01010001p.tif –sd8 NED01010001sd8.tif', replacing the three filenames with names that match your files.

#### Output(s)

For each input raster, the following two files will be created and written in the same directory as the original file:

NEDXXXXXXXXp.tif: This is the flow direction raster.

NEDXXXXXXXXsd8.tif: This is a raster of the slope, as evaluated in the direction of steepest descent, and reported as drop/distance. We do not require this raster for anything else in the toolbox.

TauDEM tools use a naming convention to identify files and later tools will recognize the *p.tif and *sd8.tif filenames to be the flow direction raster and the slope raster, respectively. It is simplest to leave the filenames alone or change them but leave the ending the same if you want to use any further TauDEM tools with these files.

#### QA/QC needed

Visually examine a couple of the flow direction rasters compared to the input DEM using a high-contrast display option for the DEM. Flow direction values should make sense with the terrain that you see and reach all the way to the wall without any NoData values in between. Otherwise, as long as there is a *p.tif file created for every input, the outputs should be okay.

#### Disk space required

The flow direction rasters take up a fraction of the disk space (i.e., less than 1/10) used by the input raster. The slope rasters take up about as much space as the input raster, but can be deleted if you are not going to use them in further analysis.

#### Error analysis

The limitations of the D8 Flow Direction method include: flow direction only occurs in intervals of 45 degrees and rarely agrees with aspect; and, flow lines tend to be parallel in areas of similar aspect.

#### Source code location

LAGOS_GIS_Toolbox/flow_direction.qsub

#### Related documentation subjects

http://hydrology.usu.edu/taudem/taudem5/TauDEM51CommandLineGuide.pdf

http://hydrology.usu.edu/taudem/taudem5/help/D8FlowDirections.html

The flow direction routing across flat areas is performed according to the method described by Garbrecht and Martz (1997) [1].

### TauDEM to ArcGIS Flow Direction

#### Description

ArcGIS uses a different direction coding for D8 Flow Direction than does TauDEM. We use ArcGIS for the remaining watershed delineation steps. This tool reclassifies the TauDEM flow direction rasters into the coding used by ArcGIS and saves the output. The direction coding used by ArcGIS is: 1 - East, 128 - Northeast, 64 - North, 32 - Northwest, 16 - West, 8 - Southwest, 4 - South, 2 – Southeast.


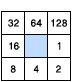


**Figure 13. Flow direction coding according to ArcGIS** (source: ArcGIS 10.1)

Although the toolbox will not use the TauDEM flow direction rasters again, the users may desire to have access to these for further analysis in TauDEM on their own.

#### Inputs

- **TauDEM Flow Direction Raster(s):** One or more flow direction rasters are created using the TauDEM D8 Flow Direction tool.
- **Output folder**: This is the folder where the outputs will be saved. We suggest creating a directory called 'arcgis_flowdirXXXX' in the output directory for each subregion; where XXXX is the 4-digit HU code, but you can use any system that you prefer.

#### Output(s)

The output is one or more flow direction rasters using the ArcGIS direction coding convention.


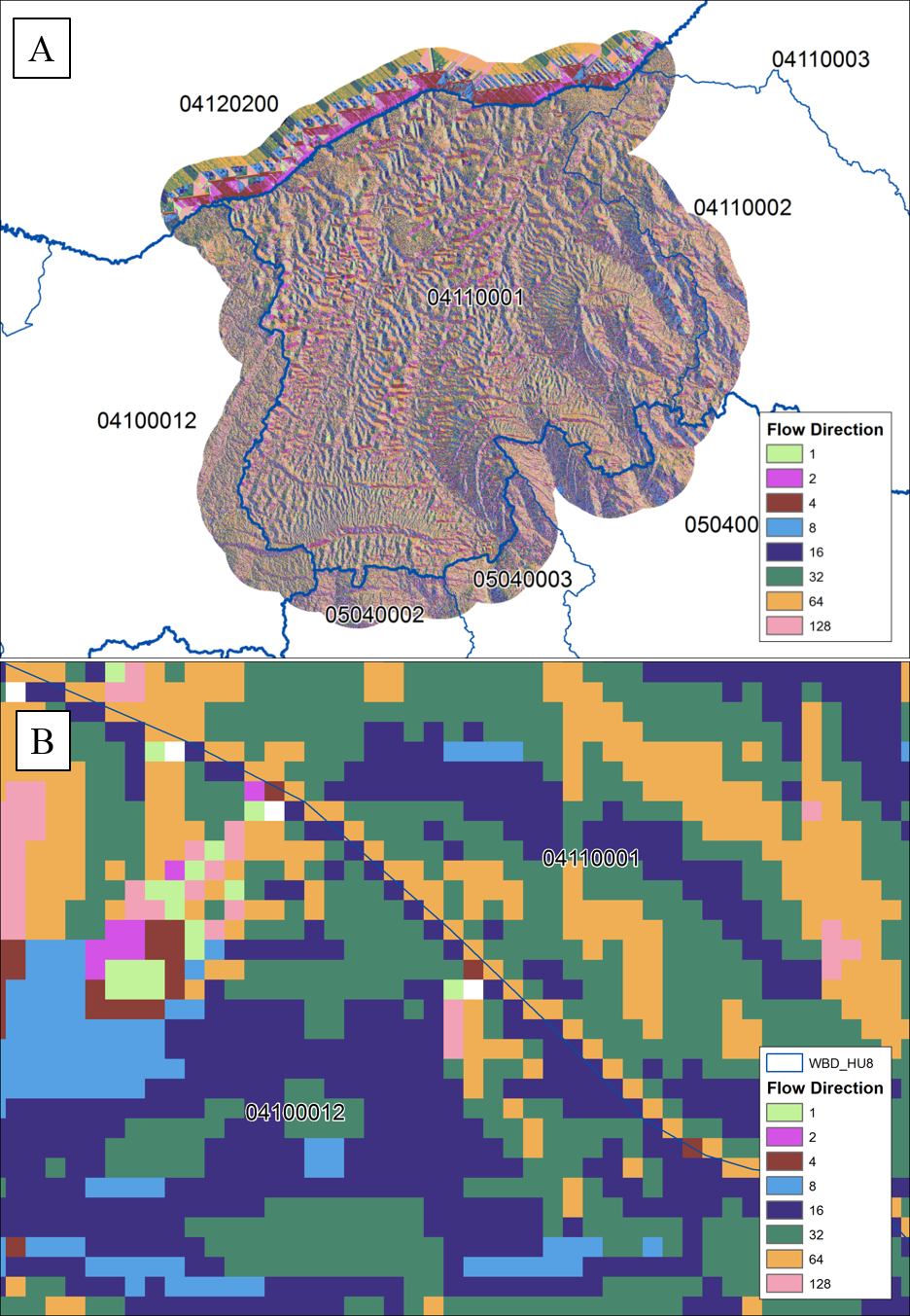


**Figure 14. a) Flow direction for whole HU8. b) Flow direction zoomed in.** The effect of the walls can be seen as a change in direction on either side of the blue line (actual wall).

#### QA/QC needed

Verify that the coding has changed for at least one raster and the rest should be okay.

#### Error analysis

No error has been introduced.

#### Disk space required

The output raster takes up about the same amount of disk space as the input raster.

#### Time

This tool is very fast.

#### Related documentation subjects

ArcGIS Spatial Analyst: Reclassify

#### Source code location

LAGOS_GIS_Toolbox/TauDEMToESRIFlowDirection.py

### Create Pour Points

#### Description

A watershed will be delineated for each outlet, or pour point, identified in this tool. We wish to identify delineate watersheds for each of the lakes and reservoirs larger than four ha (as described in Additional file 9). The NHD waterbodies corresponding to these lakes and reservoirs are identified as pour points, along with each flowline segment in the NHDFlowline layer. The pour points are represented by a raster.

#### Inputs

- **NHD geodatabase for the subregion to process: This is the NHD geodatabase for the subregion (HU4) that you want to process. We recommend using the copy created in Stage and Mosaic files rather than the original.**
- **Subregion DEM:** This is a DEM covering the subregion you want to process (the same as in NHD Geodatabase). Most likely, this will be **the mosaicked NED raster created in Stage and Mosaic files.**
- **Output directory for this subregion: This is the NHDxxxx folder for the subregion that you want to process. A folder named 'pourpointsXXXX' will be added to this folder and populated with the outputs.**

#### Output(s)

**The output is a folder named 'pourpointsXXXX' containing a raster named pour_points.tif and a geodatabase named pourpoints.gdb. The geodatabase contains two feature classes: one called 'eligible_flowlines', which is simply a copy of the NHD flowline layer, and one called 'eligible_lakes', which is the subset of NHDWaterbody that meets the LAGOS definition of a lake (i.e., greater than four ha and having one of the eligible FCodes). These layers can be merged together to make master geodata layers for your entire study region, if desired.** The pour points raster should be mostly blank space filled by NoData and each stream segment or waterbody should have a different integer cell value. Artificial lines inside water bodies will not be represented in the raster because the water bodies were laid on top of the streams in the mosaic process.


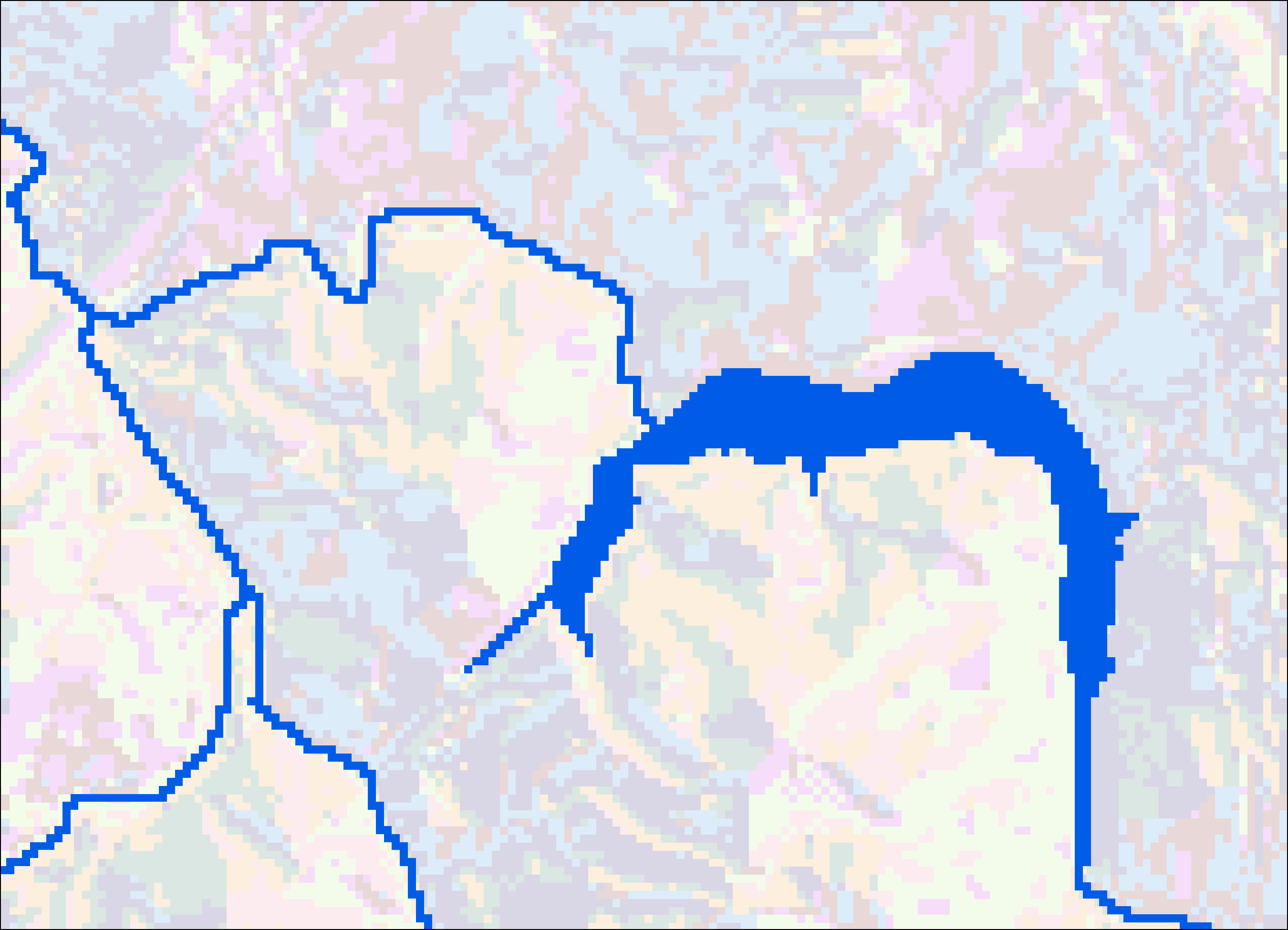


**Figure 15. Output of select pour points.** Rasterized version of lakes and streams with same grid (snap) as flow direction grid.

#### Tool steps summary:

1. Ensure that the pour points have the same cell size and 'snap' (alignment) as the DEM.
2. Make a folder and geodatabase for the pour points.
3. Make a layer from NHDWaterbody with the lakes that fit the LAGOS definition of a lake.
4. Calculate ID values that will be shared by lakes and streams so that all the raster cell values in the final product can be tied to their originating lake or stream, for example:
   1. Lakes 1-50.
   2. Streams 51-1000.
5. Convert lakes and flowlines to rasters.
6. Add the lake and flowline rasters together, with the lakes 'on top'.

#### QA/QC needed

Determine that the selections used on the features are the ones that agree with your project design, if it differs from the design developed for LAGOS.

Ensure that a geodatabase and a raster are created for each subregion.

Open at least one raster to see that the streams and waterbodies are apparent and surrounded by mostly NoData values. Water bodies will usually have much lower cell values than streams because there are many fewer water bodies than streams, and so you may need to change the symbology to be able to see them.

#### Disk space required

Usually, less than 100 MB is required for a subregion, which is often less than the size of the ESRI flow direction raster for the subregion.

#### Time

1-10 minutes each is required for most subregions.

#### Related documentation subjects

Select (analysis), Polyline to Raster, Polygon to Raster, Mosaic to New Raster

#### Source code location

LAGOS_GIS_Toolbox/GenerateSeeds.py

### Create Lake Watersheds

#### Description

This tool delineates watersheds for all of the pour points in the input using the flow direction raster to identify the divides between the watersheds.


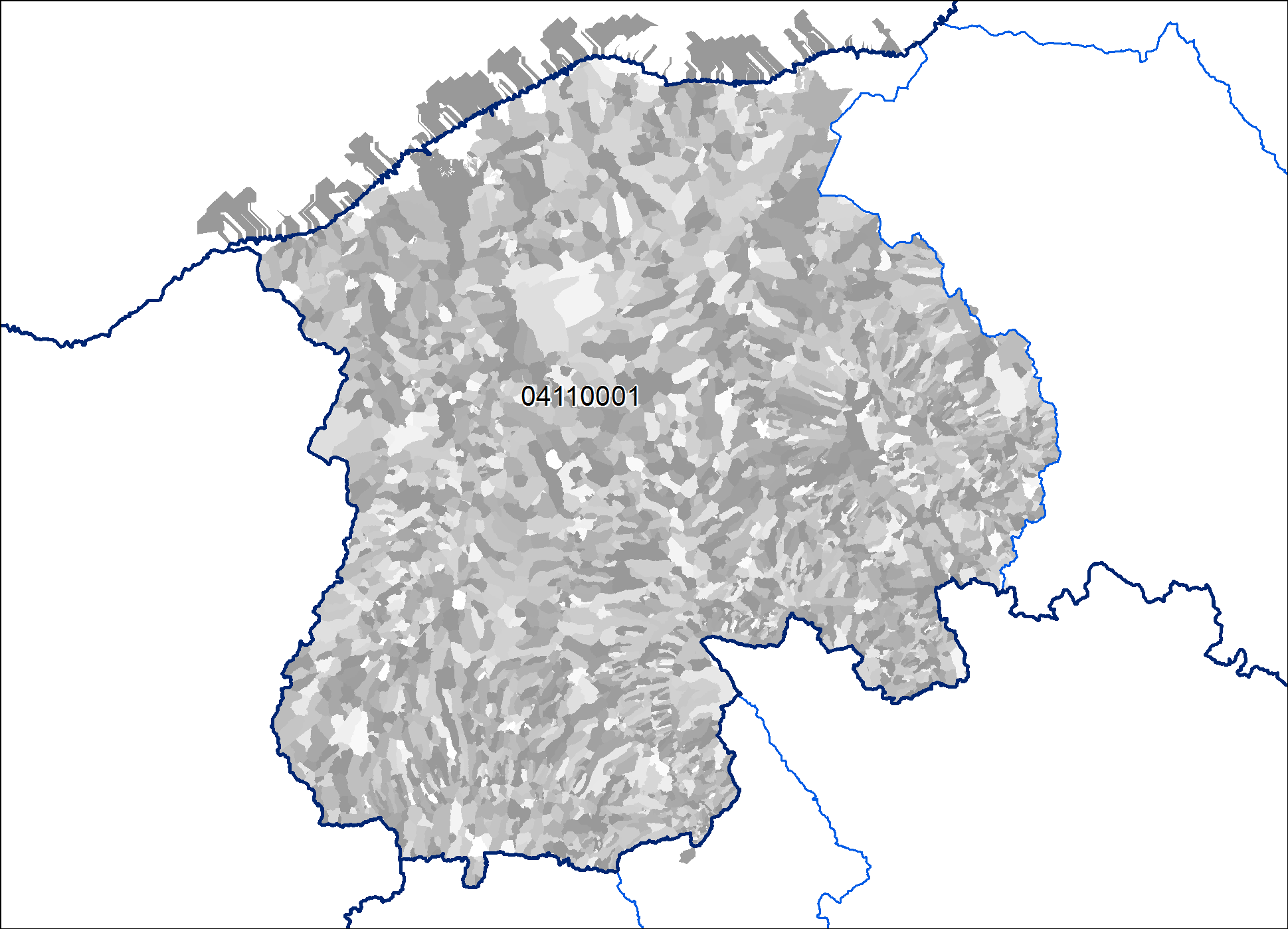


**Figure 16. Output from ArcGIS Watershed tool.** These are the watersheds that were cleaned up by our custom process.


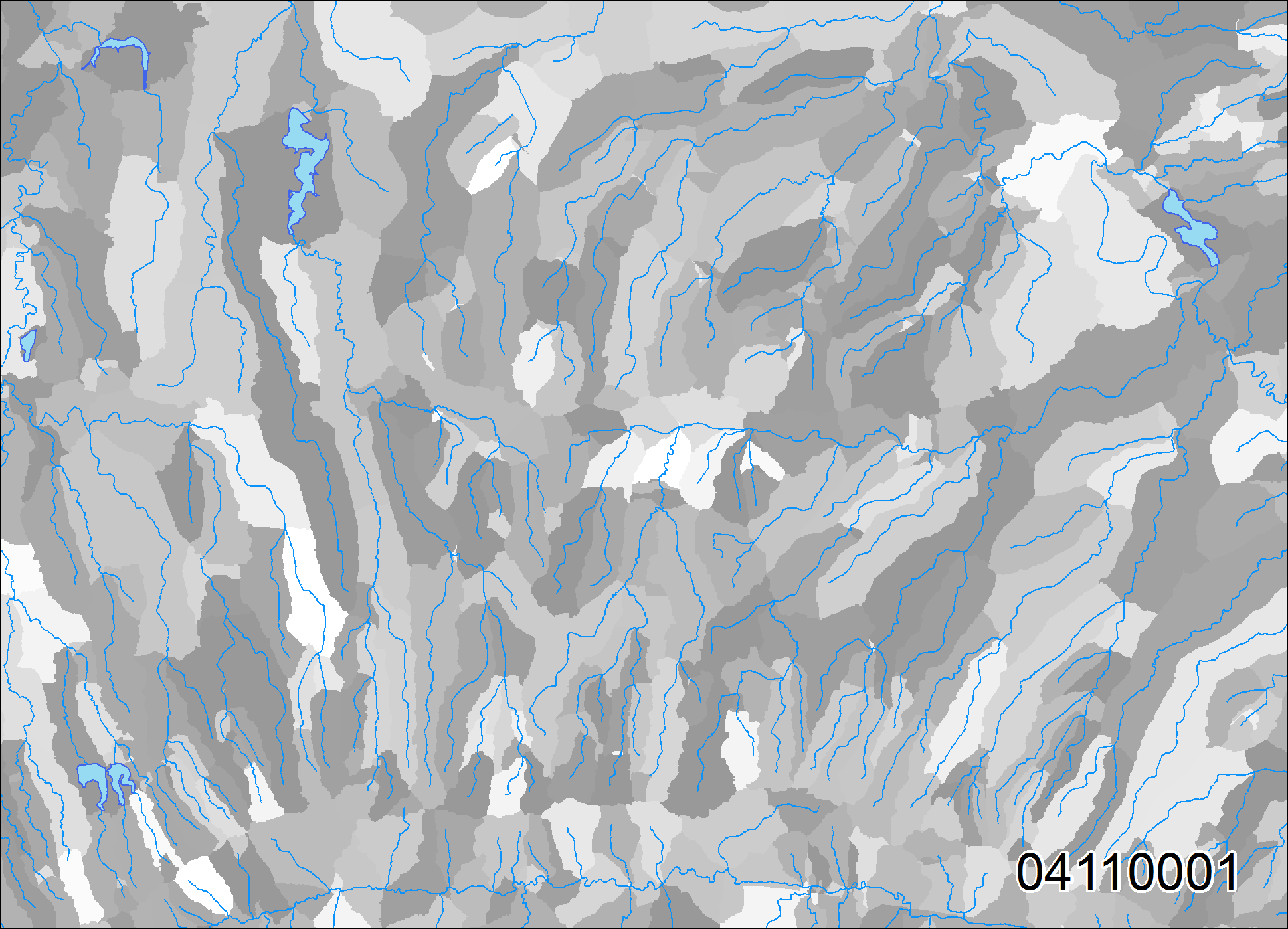


**Figure 17. Close-up view of watersheds output that shows how the landscape is divided up into watersheds for each lake and for each flowline segment (confluence to confluence).**

The watersheds are then re-shaped to eliminate small sliver watersheds and watersheds that do not 'belong' to any pour points, and divide the vacant areas among the remaining watersheds using a nibble function. The watersheds are converted to polygons in the output, which are clipped to the HU8 boundary. The Permanent_Identifier of each lake is joined to its watershed using common identifiers. Lakesheds are re-shaped slightly to guarantee that the associated lake is perfectly nested within its own watershed region. This tool results in a set of lakesheds that contain the lake completely and are perfectly nested within HU8s (the lakesheds cannot cross HU8 boundaries, even if the lake does).


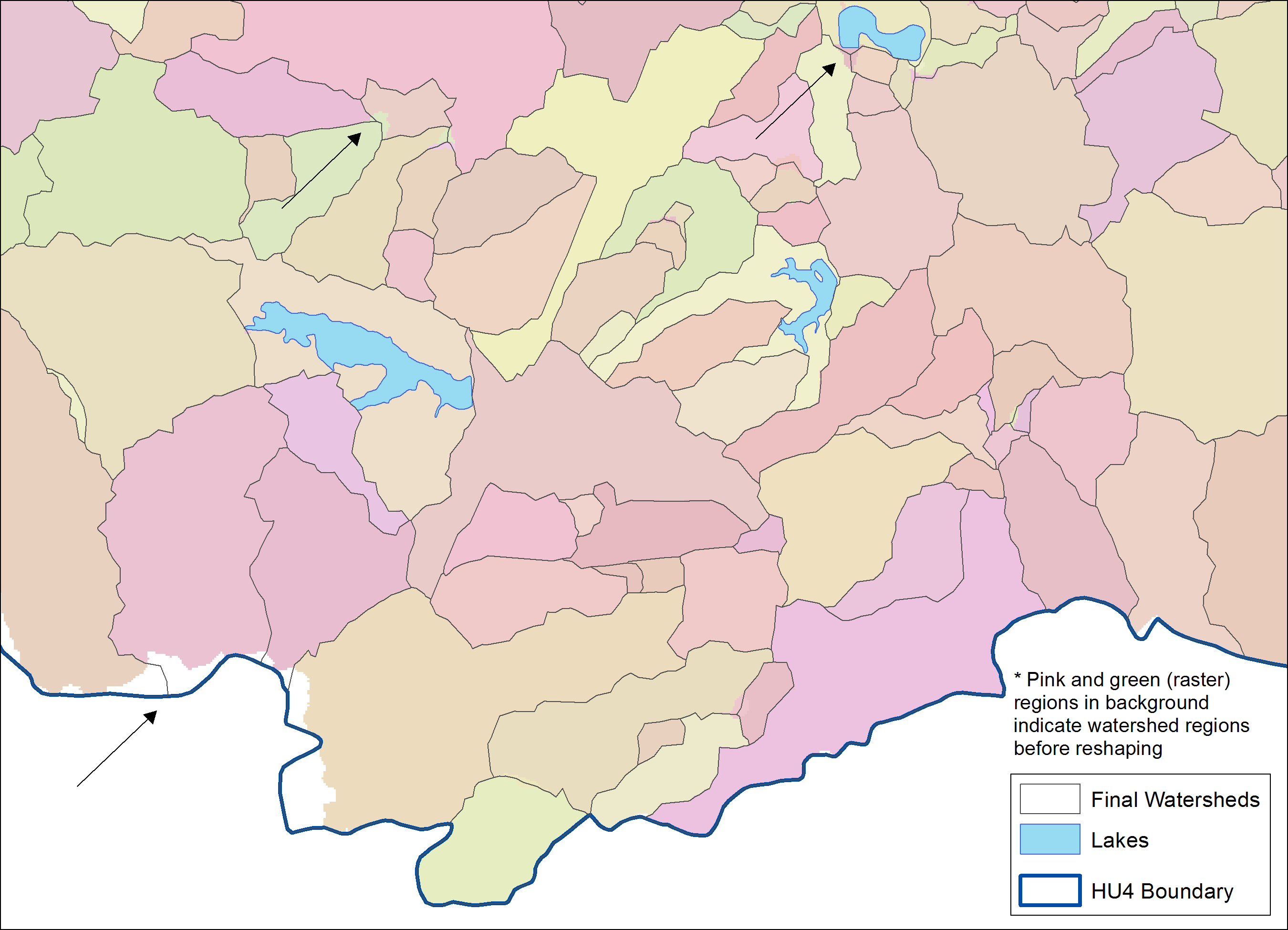


**Figure 18. Some of the changes made by our custom cleaning procedure.** The arrows point to sliver watersheds that are eliminated and voids that are filled in by the nearest watershed during the 'nibble' analysis within the tool.

#### Inputs

- **Flow direction raster:** This is the flow direction raster using the ArcGIS direction coding (the output from Convert TauDEM Flow Direction Raster to ArcGIS format).
  **Pour points directory for this HU8's subregion:** This is the pourpointsXXXX directory output from the Create Pour Points tool for the subregion (HU4), including the HU8 that you want to process.
- **NHD geodatabase for this HU8's subregion: This is the NHD geodatabase for the subregion (HU4), including the HU8 that you want to process. We recommend using the copy created in Stage and Mosaic files rather than the original.**
- **Output geodatabase: This is an output geodatabase that will contain the watersheds 'precursors' raster and the final watersheds polygon feature class. You can either create a new geodatabase in the folder you have been using for this subregion or one geodatabase to hold all of the outputs.**

#### Output(s)

This tool produces the following two outputs in the output geodatabase:

1. The original watersheds raster created by the watersheds tool, called hucXXXXXXXX_watersheds_precursors.
2. A polygon feature class with the final watersheds belonging to lakes sharing their respective 'Permanent_Identifier' values. The lakes are not erased from their watersheds. Stream/flowline watersheds are included but they are not identified because they will not need to be associated with their origin flowline at a later point.

#### Warning(s)

Our custom 'cleaning' procedures suit our purposes but they slightly modify the boundaries of the watersheds created by the ArcGIS watershed tool and are not exactly the same. Mostly, they resolve small watersheds caused by any remaining mismatch between pour point locations and flow accumulation locations, even after our DEM conditioning, and they fix the null areas created by our walls along the HU8 boundaries. However, this process may obscure legitimate problems with our DEM conditioning; it may also cause other changes.

Watersheds may not cross HU8 boundaries even if the lake is on a HU8 boundary. 265 out of 51107 lakes in our 17-state region are on the boundaries of HU8s. A few lakes, like Lake Champlain, span multiple HU8s.

Because there are duplicated lakes between subregions in the NHD, we recommend caution when merging these watersheds for multiple subregions. A good approach to deal with this is to merge them all and then join the result to a master lakes layer, and then let any unmatched watersheds drop out.

#### Tool steps summary

1. Use the ArcGIS Watershed tool to calculate watersheds using pour points and flow direction raster.
2. Convert watershed raster to polygons.
3. Clip polygons to HU8 boundary.
4. Create feature class of watershed polygons ≥1 ha that intersect pour point lakes or stream segments (as polygons/lines).
5. Convert only these 'valid' watersheds back to a raster and clip to the HU8.
6. Manipulate raster cell values to create the NoData mask used in the upcoming nibble function.
7. Nibble at cells that are NoData in the mask, replace the values with the nearest 'eligible' value from the value raster.
8. Clip the nibble output to a 100 m buffer of the HU8 and leave the buffer there so that once the raster is converted to polygon, the polygons will come right up to the edge of the HU8 when clipped off.
9. Convert the raster to a polygon.
10. Clip the polygons to the HU8 boundary.
11. The polygons have a field 'grid_code' that corresponds to the lake 'POUR_ID' values. Use this relationship to join the watersheds and lakes so that the lakesheds have the appropriate 'Permanent_Identifier'.
12. Ensure that the lakesheds contain their own lakes. Bump out lakeshed boundaries slightly where they are within their own lakes using the ArcGIS Update (analysis) and Dissolve (data management) tools.

#### QA/QC needed

Visually inspect the watersheds to see how they look.

Ensure that there is a value for the ID field for every lake. Null values will be present for watersheds that do not belong to lakes.

Ensure that the watersheds do not cross any boundaries that they should not cross.

#### Error analysis

We have not performed an error analysis for the un-aggregated lakesheds. See the Aggregate Watersheds: Interlake error analysis section for a discussion of how the watersheds delineated by this process compared to watersheds delineated 'by hand'.

#### Disk space required

Output (1) usually takes less than 20 MB and Output (2) is usually less than 5 MB.

#### Time

Larger HU8s take longer to run. The fastest HU8s take about 3 minutes to run. Most HU8s take under 30 minutes but the very largest may take several hours.

#### Related documentation subjects

ArcGIS tools: Watershed, Raster to Polygon, Polygon to Raster, Select Layer By Location, Select Layer By Attribute, Spatial Analyst: Con, Is Null, Nibble, Spatial Join, Update, Dissolve

#### Source code location

LAGOS_GIS_Toolbox/create_lake_watersheds.py

### Merge Lake Watersheds by Subregion

#### Description

This is a simple tool that merges the lakesheds into one feature class per subregion. It also checks whether the number of input feature classes is less than, equal to, or more than the number of HU8s in the original HU4 (as counted in the NHD geodatabase) and issues an error, warning, or okay message accordingly.

#### Inputs

- **Watersheds feature classes: Select all of the watershed feature classes for the subregion that you want to process. Do not select watershed feature classes from outside this subregion!**
- **NHD geodatabase for this subregion: This is the NHD geodatabase for the subregion (HU4) that you want to process. We recommend using the copy created in Stage and Mosaic files rather than the original.**
- **Output feature class: This is the location of the output feature class**

#### Output(s)

The output is one feature class per subregion with all the watersheds. Watersheds may be duplicated (one on top of another), or there may be two non-overlapping watersheds for the same lake if that lake is on an HU8 boundary. The Aggregate Watersheds: Interlake tool will handle these watersheds by dissolving them.

### Aggregate Watersheds: Interlake Mode

#### Description

For each lake in the subregion or sub-basin, an aggregated watershed is created by tracing the network upstream of the lake. This watershed is the area on the landscape draining to the focal lake, which includes the land that drains into inflowing streams but does not include the area of land that drains into upstream lakes with an area ≥10 ha. Each lake is erased from its own watershed. Lakes without any upstream connections are included in the output with the watersheds that they had from the last tool.

***
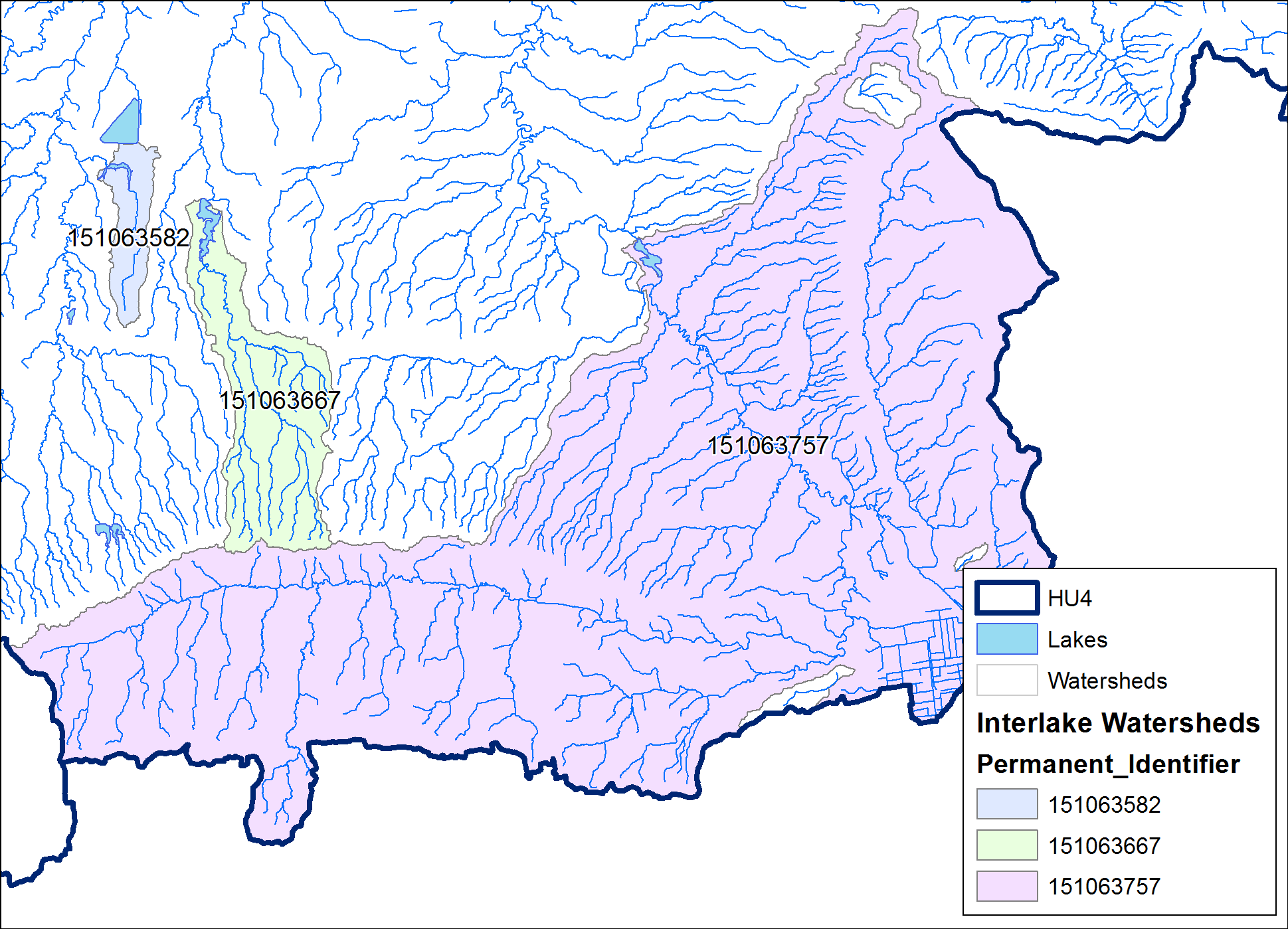
***

**Figure 19. Interlake watersheds extend to where the upstream network ends.** They do not cross subregion boundaries.


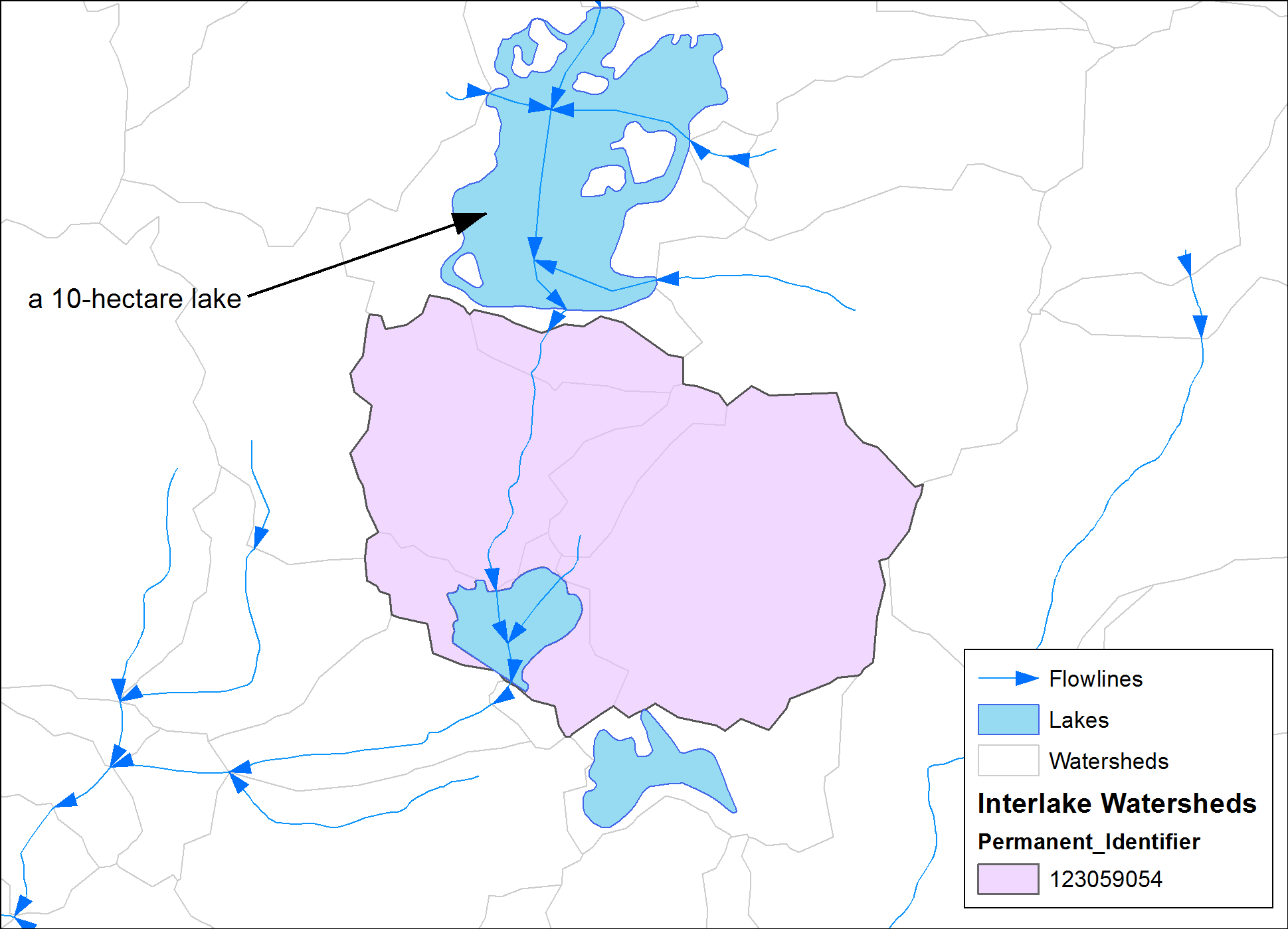


**Figure 20. Shows how the interlake watershed does not include any of the upstream network beyond and including a lake ≥10ha.**

#### Inputs

- **Input watersheds feature class: This is the watersheds feature class (an output from Create Lake Watersheds).**
- **NHD geodatabase for this subregion: This is the NHD geodatabase for the subregion (HU4) that you want to process. We recommend using the copy created in Stage and Mosaic files rather than the original.**
- **Pour points directory for this subregion:** This is the pourpointsXXXX directory output from the Create Pour Points tool for the subregion (HU4) that you want to process.
- **Output feature class: This is the output feature class with the interlake watersheds.**

#### Output(s)

The output is a polygon feature class with an interlake watershed for each lake. Lakes with no upstream connections will still be included with their own watersheds. Lakes are erased from their own interlake watershed.

Interlake watersheds cannot cross subregion boundaries (they are nested within subregions).

Interlake watershed polygons can and frequently do overlap.

An interlake watershed is the same extent or smaller than the cumulative watershed for the same lake.

#### Warning(s)

Watershed polygons can and frequently do overlap. Display may be difficult. Try using the 'Identify' tool in ArcGIS to identify which polygons are overlapping at a point and flash a particular one. Consider whether overlapping polygons are a problem in any analysis you perform using interlake watersheds. Tools from the LAGOS GIS Toolbox are designed to handle these overlapping polygons.

#### Tool steps summary

1. Use a layer of lakes ≥ 4 ha as the population of lakes.
2. Create a layer of lakes ≥10 ha and select the junctions from the HYDRO_NET (hydrology network) in the NHD geodatabase that intersect them (use a 1 m search radius, i.e. a 1 m tolerance).
3. For each lake in (1):
   1. Make a feature layer with only this lake ('this lake').
   2. Select junctions that intersect this lake (use a 1 m tolerance).
   3. If the count of the junctions is zero:
      1. Select the watershed containing this lake and go to step d., iv.
   4. If the count of the junctions is at least 1:
      1. Trace Geometric Network using HYDRO_NET geometric network in the NHD geodatabase, selecting all junctions and flowlines upstream of the junctions that intersect this lake
      2. Select watersheds that contain or are crossed by the outline of (i.e. intersect, but using the intersect keyword causes extra watersheds that nearly intersect to be included, for unknown reasons) the flowlines in the upstream network. Flowlines go through lakes too, so in other words, select lake/stream watersheds upstream of this lake.
      3. Remove any watersheds containing lakes ≥10ha from the selection.
      4. Dissolve the selected watersheds into one feature.
      5. Erase this lake from its own interlake watershed.
      6. Append this interlake watershed to the output feature class.

#### QA/QC needed

Identify a few lakes representing different positions in their respective local stream networks and see that the watershed encompasses all that it should, and stops where it should (i.e., the beginning of its upstream network, the subregion boundary, or a ≥ 10 ha lake, whichever comes first).

#### Error analysis

A formal error analysis of the interlake watersheds resulting from the LAGOS GIS Toolbox has yet to be performed. A preliminary qualitative comparison is presented in the following figures, which show the automated interlake watersheds created by this tool overlaid with interlake watersheds created by MSU RS&GIS using the traditional method for some lakes in Michigan that were all greater than 20 ha. The RS&GIS watershed requirements specified that the watershed sometimes did not cross roads which the automated watersheds could, but otherwise the same requirements were specified.


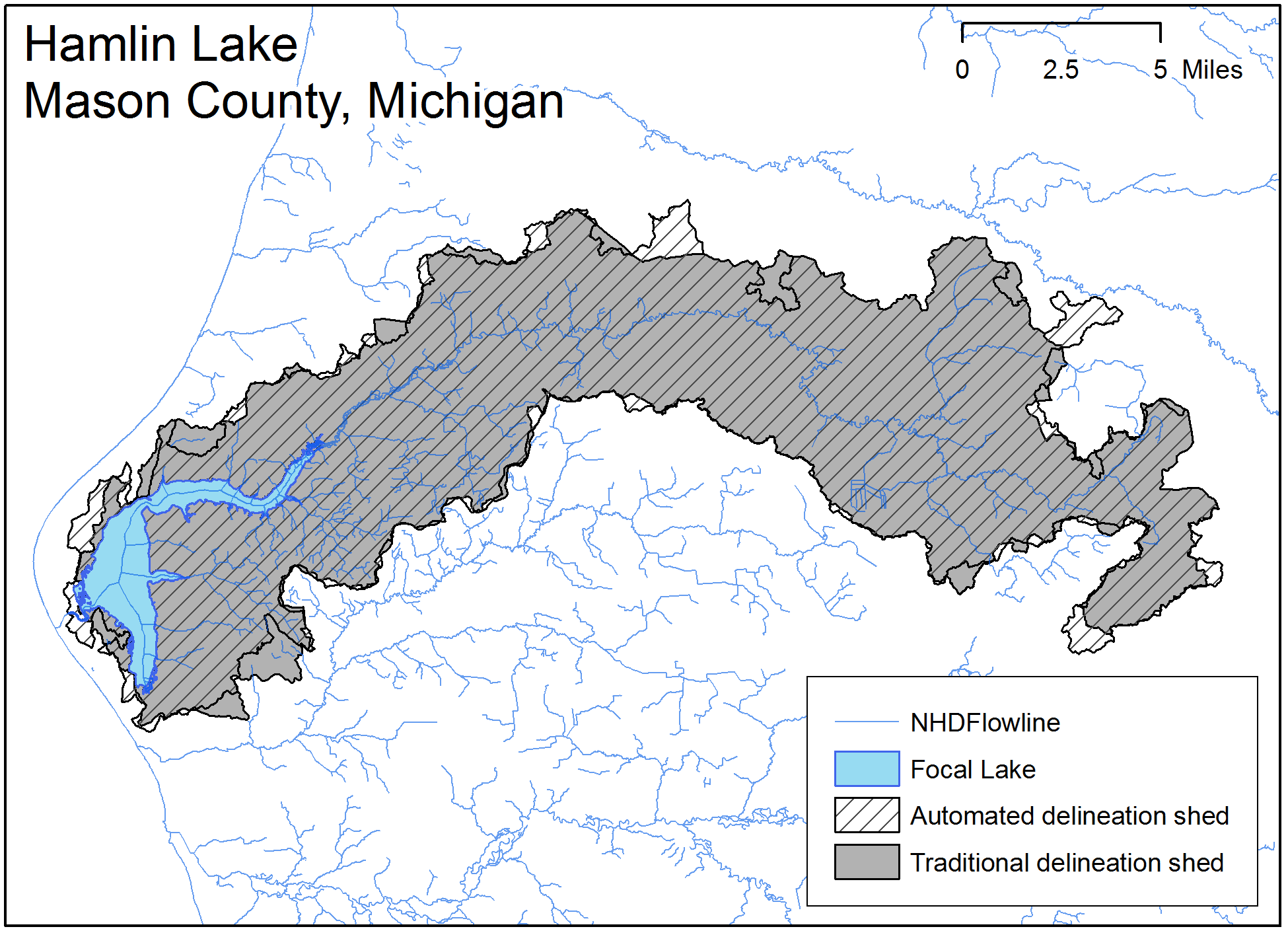


**Figure 21: The two watersheds are substantially the same in shape and extent, with some disagreement along the outer boundaries.** In the central area of the northern border, you can see a straight line in the traditionally delineated shed where the boundary was drawn along a major road.


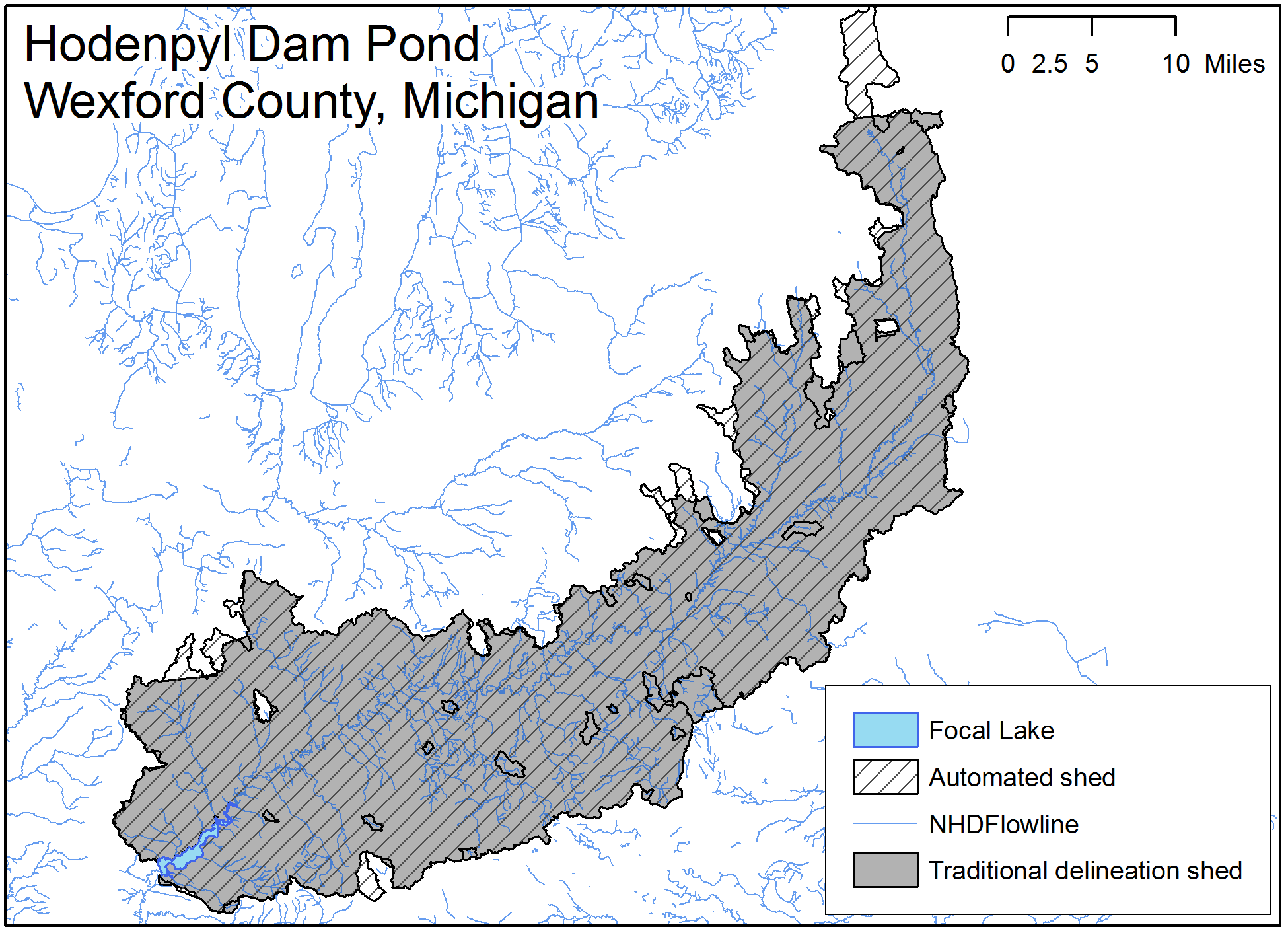


**Figure 22: The two watersheds are substantially the same in shape and extent.** However, there are more holes (i.e., regions of flow isolated from the watershed) in the watershed created with this toolbox. The automated shed also has a major protrusion 'upstream' beyond the boundaries of the traditionally -delineated watershed.


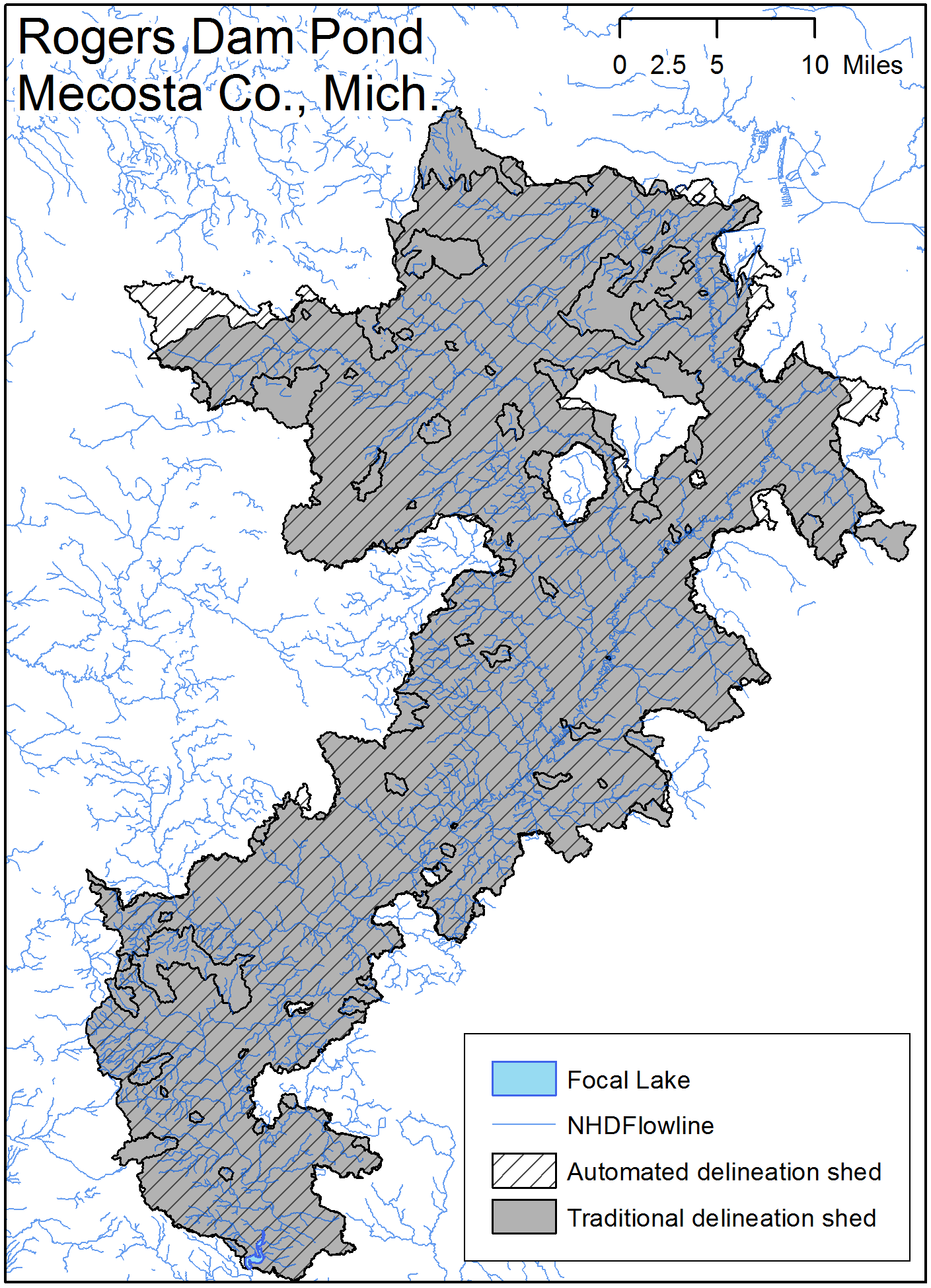


**Figure 23. These two watersheds are substantially similar in shape and extent.** However, the watershed delineated by this toolbox has considerably more holes (i.e., regions of isolated flow) than the traditionally delineated watershed, although the two agree on several large isolated regions.

#### Disk space required

Under 50 MB for most subregions.

#### Time

This tool usually takes under an hour to run for a subregion.

#### Related documentation subjects

Select (analysis), Select Layer By Location, Trace Geometric Network, Dissolve, Erase

#### Source code location

LAGOS_GIS_Toolbox/interlake2.py

### Aggregate Watersheds: Cumulative Mode

#### Description

For each lake in the subregion or sub-basin, an aggregated watershed is created by tracing the network upstream of the lake. This watershed is the area on the landscape draining to the focal lake, which includes the land that drains into inflowing streams. Aggregation will continue until the network ends or the HU8 boundary is encountered. Each lake is erased from its own watershed. Lakes without any upstream connections are included in the output with the watersheds that they had from the last tool.

#### Inputs

- **Input watersheds feature class: This is the watersheds feature class (an output from Create Lake Watersheds).**
- **NHD geodatabase for this subregion: This is the NHD geodatabase for the subregion (HU4) that includes the HU8 that you want to process. We recommend using the copy created in Stage and Mosaic files rather than the original.**
- **Pour points directory for this subregion:** This is the pourpointsXXXX directory output from the Create Pour Points tool for the subregion (HU4) that includes the HU8 you want to process.
- **Output feature class: This is the output feature class with the cumulative watersheds.**

#### Output(s)

The output is a polygon feature class with an interlake watershed for each lake. Lakes with no upstream connections will still be included with their own watersheds. Lakes are erased from their own interlake watershed.

Interlake watersheds cannot cross HU8 boundaries (they are nested within HU8s).

Watershed polygons can and frequently do overlap.

An interlake watershed is the same size or smaller than the cumulative watershed for the same lake.

#### Warning(s)

Watershed polygons can and frequently do overlap. Display may be difficult. Try using the 'Identify' tool in ArcGIS to identify which polygons are overlapping at a point and flash a particular one. Consider whether overlapping polygons are a problem in any analysis you perform using interlake watersheds. Tools from the LAGOS GIS Toolbox are designed to handle these overlapping polygons.

Coastlines are included in the upstream trace. If a lake intersects a coastline, then it may have a strange interlake watershed.

#### Tool steps summary

1. From the lakes in the pour point layer, select those that intersect the HU8 (lakes on the boundary will be included.)
2. Create a layer of lakes ≥10 ha and select the junctions from the HYDRO_NET (hydrology network) in the NHD geodatabase that intersect them (use a 1 m search radius, i.e. a 1 m tolerance).
3. For each lake
   1. Make a feature layer with only this lake ('this lake').
   2. Select junctions that intersect this lake (use a 1 m tolerance).
   3. If the count of the junctions is zero:
      1. Just copy the watershed containing this lake
   4. If the count of the junctions is at least 1:
      1. Trace Geometric Network using HYDRO_NET geometric network in the NHD geodatabase, selecting all of the junctions and flowlines upstream of the junctions that intersect this lake.
      2. Select watersheds that contain or are crossed by the outline of (i.e. intersect, but using the intersect keyword causes extra watersheds that nearly intersect to be included, for unknown reasons) the flowlines in the upstream network. Flowlines also go through lakes. In other words, select lake/stream watersheds upstream of this lake.
      3. Remove any watersheds containing lakes ≥10ha from the selection.
      4. Dissolve the selected watersheds into one feature.
      5. Erase this lake from its own interlake watershed.
      6. Append this interlake watershed to the output feature class.

#### QA/QC needed

Identify a few lakes representing different positions in their respective local stream networks and see that the watershed encompasses all that it should, and stops where it should (i.e., the beginning of its upstream network, the HU8 boundary, or a >10 ha lake, whichever comes first).

#### Error analysis

We have not yet performed an error analysis of cumulative watersheds.

#### Disk space required

Under 5 MB for most HU8s.

#### Time

This tool usually takes just a couple of minutes to run and almost always completes in less than 10 minutes.

#### Related documentation subjects

Select (analysis), Select Layer By Location, Trace Geometric Network, Dissolve, Erase

#### Source code location

LAGOS_GIS_Toolbox/interlake2.py

## Utilities

Only selected utilities are described here.

### Export to CSV

All of the outputs created by the LAGOS GIS Toolbox are saved to file geodatabases. When you want to use tabular data outside of ArcGIS, this tool exports the tables (or feature class attribute tables) to CSV files. It converts null values to 'NA' strings, drops shape geometry fields, and suppresses scientific notation in the CSV files.

### Merge NHD Features

This tool is used to merge NHD features 'seamlessly, 'avoiding duplicates along the boundaries between subregions. It also allows the user to subset a selection of the data.

NHD features may be previously subset or have additional fields (such as Strahler number) as long as they still have one feature per Permanent_Identifier ID.

Recommended uses:

-Merge all flowlines in your study area (and select only certain types of flowlines, if desired).

-Merge all waterbodies and select only certain lakes in your study area to make a master lakes layer to -use in many of the LAGOS GIS Toolbox tools.

-Merge all NHDArea polygons and subset the StreamRiver type ('FType' = 460) before using the Wetland Order tool.

#### Inputs

- **NHD geodatabases:** Choose all of the geodatabases from which you wish to merge features.
- **NHD feature class (Choose from any geodatabase):** This is the feature class that you will merge from each geodatabase, for instance: NHDArea, NHDFlowline, or NHDWaterbody. Choose a feature class from any of the geodatabases and the other feature classes of the same name in the other geodatabases will be found automatically by the script.
- **Selection query (optional):** This is an SQL expression to subset certain features from the input feature classes. WARNING: The 'Get Unique Values' button will turn up all unique values from the feature class that you chose in the parameter above but it will not necessarily represent all the possible values in all of the feature classes that you are merging. You can (and should) still type these values in the query yourself.
- **Output feature class:** This is the location of the output feature class

#### Output

The output is one feature class with features from all the geodatabases merged together. If you need a GEOGRAPHIC subset of these features (clip or select along the borders of your project area), then you must do this yourself to the output of this tool.

#### Tool steps summary

1. Set up environments (all outputs will be saved in the USGS Albers projection).
2. Make a list of all the feature classes using the exemplar feature class entered by the user and merge them (do this by copying the first feature class, lengthening the Permanent_Identifier field to avoid schema mismatch problems that always come up with that field, and append the remaining feature classes).
3. If there is a selection query expression entered, do the selection.
4. Remove all but the newest feature for every set of duplicated ID values using the assumptions.remove_nhd_duplicates function from the LAGOS GIS Toolbox.
5. If the feature class has the 'Polygon' shape type (for instance, lakes and stream polygons), then remove geographic duplicates or substantial overlaps (more than 10%) by using the assumptions.remove_geographic_doubles function from the LAGOS GIS Toolbox.

# References

1. Garbrecht J, Martz LW: **The assignment of drainage direction over flat surfaces in raster digital elevation models**. *Journal of Hydrology* 1998, **193**: 204-213.

2. Hughes RM, Kaufmann PR, Weber MH: **National and regional comparisons between Strahler order and stream size**. *Journal of the North American Benthological Society* 2011, **30**: 103-121.

3. TauDEM, Version 5. http://hydrology.usu.edu/taudem/taudem5/index.html

# Michigan State University HPCC Users Appendix

The documentation and user manual for the MSU HPCC can be found at https://wiki.hpcc.msu.edu/display/hpccdocs/Documentation+and+User+Manual

A few tips:

Use Globus to transfer large amounts of data. As a courtesy to other users, the HPCC asks that you try to plan very large transfers to take place outside of normal work hours (e.g., after 6 pm on weekdays).

If you are on the MSU network, you can map an HPCC drive to your campus computer following these directions: https://wiki.hpcc.msu.edu/display/hpccdocs/Mapping+HPC+drives+to+a+campus+computer

Use HPCC/[your NetID] as the username and your MSU NetID password.

When you first connect to the HPCC, issue the command 'ssh dev-intel07' or ssh followed by whichever node has low usage or meets your needs. The gateway is intended for basic file manipulation and job submission only.

As far as we can tell, it is okay to run very short jobs like GDAL translate, merge, etc. that are under a few minutes outside the job queue.
