# Supplementary material for: Building a multi-scaled geospatial temporal ecology database from disparate data sources: fostering open science and data reuse
Source: Gigascience. 2015 Jul 1;4:28. doi: 10.1186/s13742-015-0067-4 (PMC4488039; doi:10.1186/s13742-015-0067-4)
Supplement: Additional file 9: — Data sources, definition, and classification of lakes. Detailed description of the sources, definitions and classifications that were used to describe lakes in LAGOS. We also include a description and justification for the minimum lake size used in LAGOS based on an error analysis of the data source. [file 13742_2015_67_MOESM9_ESM.docx]

# Additional file 9

# Data sources, definition, and classification of lakes

Patricia Soranno, Kendra Spence Cheruvelil, Emi Fergus, Katherine Webster, Nick Skaff, Craig Stow, Scott Stopyak, Ty Wagner, Mary Bremigan, Ed Bissell

# OVERVIEW

LAGOS_GEO_ uses the National Hydrography Dataset (NHD) GIS coverage [1] as the basis for locating and identifying all lakes comprising the LAGOS census population within the 17 state study area. The purpose of this document is 3-fold: 1) to define the term 'lakes' as used in this project, 2) to describe our efforts to quantify the error associated with the NHD's characterization of the presence/absence of lake polygons and how these errors differ regionally in our study area, and 3) to describe how lakes were classified according to hydrological connectivity.

LAGOS comprises LAGOS_GEO_ (a database of all lakes in the 17 state study area for which we have all of the geographic data) and LAGOS_LIMNO_ (a database of only lakes for which we have at least one lake chemistry data point). LAGOS_LIMNO_ is thus nested within LAGOS_GEO_.

Before we can define the term 'lake', we must first examine the error within NHD's classification of the presence/absence of a lake polygon. We focus on error rates in lakes at the small end of lake size distribution because more errors occur in digitization of smaller lakes. Large lakes are consistently well-represented in the NHD but small lakes (those less than 4 ha) are not.

There are several features of LAGOS that require a detailed description of how lakes are identified and included in LAGOS. An important theme of our research is to consider and extrapolate results to the full population of lakes in our 17 state study area. However, if we extrapolate to all lakes larger than 1 ha, although the NHD only detects about 50% of the lakes between 1 and 4 ha, then our extrapolations will be inaccurate for small lakes. Additionally, if regional differences in the inaccuracies occurring in the NHD exist, then the extrapolation in our study area will suffer further bias. In addition, the delineation of lake watersheds for all lakes in our study area is done using an automated procedure. Because the NHD only detects about 50% of lakes between 1 and 4 ha, only that percentage of the small lakes will receive a watershed using this automated procedure and the remaining small lakes and their watersheds will incorrectly be incorporated within other watersheds.

Another important characteristic of LAGOS is the surface connectivity among lakes through stream connections. To identify lakes within a 'lake network' and to determine the correct number and degree of connectivity of lakes within that network, we need to be sure that lakes are consistently being detected across our study region. We classified each lake ≥ 1 ha based on its surface connections to streams and other lakes. This classification scheme is based on many past limnological classifications that divide lakes into such types as 'drainage vs seepage'. However, we have added two additional categories that we consider important in describing the surface hydrology of lakes. The intent of this classification is to identify lakes that have similar connections to the surrounding landscape. This modification to the connectivity classification is based on recent research on lake nutrients in a landscape context that have examined the importance of upstream lakes in influencing downstream nutrients [2], the relationship between landscape position and lake nutrients [3,4], and the differing relationships between land use/cover and lakes of different hydrologic type.

# Data source: National Hydrography Dataset (NHD)

*Copied directly from the website and reprinted here for background information only. It is suggested that users refer to the original source:*

“The NHD is a digital vector dataset (USGS). It contains features such as lakes, ponds, streams, rivers, canals, and ditches. These data are designed to be used in general mapping and in the analysis of surface-water systems. NHD data are continuously being updated.


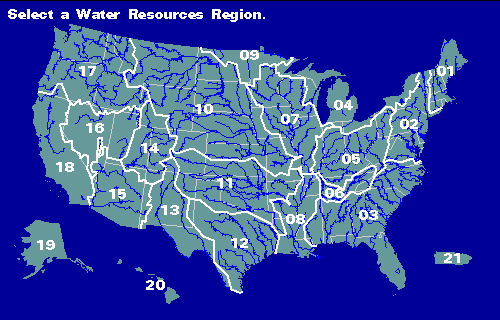


**Figure S12. Map of USGS delineated Water Resources Regions [6].**

The United States are divided and sub-divided into successively smaller hydrologic units, which are classified into four levels: regions, sub-regions, accounting units, and cataloging units. The hydrologic units are arranged or nested within each other, from the largest geographic area (regions) to the smallest geographic area (cataloging units). Each hydrologic unit is identified by a unique hydrologic unit code (HUC) consisting of two to eight digits based on the four levels of classification in the hydrologic unit system.

The first level of classification divides the Nation into 21 major geographic areas, or regions (Figure S12). These geographic areas contain either the drainage area of a major river, such as the Missouri region, or the combined drainage areas of a series of rivers, such as the Texas-Gulf region, which includes a number of rivers draining into the Gulf of Mexico. Eighteen of the regions occupy the land area of the conterminous United States. Alaska constitutes region 19, the Hawaii Islands are region 20, and Puerto Rico and other outlying Caribbean areas are region 21.

The second level of classification divides the 21 regions into 221 sub-regions. A sub-region [HUC4] includes the area drained by a river system, a reach of a river and its tributaries in that reach, a closed basin(s), or a group of streams forming a coastal drainage area.

The third level of classification subdivides many of the sub-regions into accounting units. These 378 hydrologic accounting units [HUC6] are nested within, or can be equivalent to the sub-regions.

The fourth level of classification is the cataloging unit [HUC8], the smallest element in the hierarchy of hydrologic units. A cataloging unit is a geographic area representing part of all of a surface drainage basin, a combination of drainage basins, or a distinct hydrologic feature. These units subdivide the sub-regions and accounting units into smaller areas. There are 2264 Cataloging Units in the Nation. Cataloging Units sometimes are called 'watersheds'.

In addition to the hierarchical region classifications, the NHD groups water bodies into several types of geometric features with linked attributes. The most important include the NHDWaterbody polygons which portray the geometry of lakes and ponds, the NHDArea polygons, which include stream and river features, and finally the NHDFlowline feature class, which models the direction of water flow in stream, river, and artificial path features [1,5,6].

# LAGOS: Definition of a 'lake'

A 'lake' in LAGOS is a perennial body of relatively still water. We include lakes and reservoirs that range from being completely natural to highly modified: lake basins can be entirely natural, modified natural (i.e., a water control structure on a natural lake), or a fully impounded stream or river (i.e., a reservoir). We explicitly exclude the following water bodies: sewage treatment ponds, aquaculture ponds, or other such detention ponds that are known to contain basins that are entirely artificial and were built for high-intensity human use. The operational minimum lake size in our database is 4 ha. Although LAGOS_LIMNO_ does include lakes between 1 and 4 ha for which we have a lake location, lake area, perimeter, and lake connectivity class, there is no LAGOS_GEO_ data associated with these lakes. There are very large errors in the NHD (see below for details). Therefore, we recommend caution in analyzes, interpretation, and inference for lakes < 4 ha in size that depend on NHD's spatial representation of water bodies.

The above definition of 'lake' for LAGOS has been developed only for the purpose of this database and its applications (e.g., to answer questions about lake water quality). The intent of LAGOS is not to document and measure the total number of water bodies in our study area, although we are able to perform this calculation for lakes ≥ 4 ha. Other approaches (e.g., remote sensing) and other sources are preferred to answer such questions.

## NHD Background

Although the NHD is a national dataset, it is updated and edited regionally (often at the state level) by local practitioners familiar with each study region. As a result, there are differences in the resolution and digitization of water bodies, particularly for small water bodies. As a result, it is difficult to quantify or document even nominal error rates, or rather, the minimum lake size that is well-represented in the NHD. It has been documented previously that the NHD may not do a good job of identifying small water bodies due to the resolution of the underlying data used to digitize water bodies for the NHD database (e.g., [7–9]

Because of these documented issues, some programs have set minimum lake area cutoffs for sampling lakes. Most notable is the EPA-National Lake Survey of 2007, which chose a minimum size of 4 ha. To determine an appropriate size cutoff for our purposes, we have conducted an analysis in an attempt to identify the lakes that are best represented by the NHD across the LAGOS study area.

## Consequences of errors in incomplete digitization of small water bodies

Because of their small size, small lakes can be incompletely digitized (i.e., by not being digitally recorded when in fact they are present, or by being incorrectly classified, such as when a swimming pool is erroneously identified as a lake). Incomplete digitization of small water bodies is problematic for two important features of LAGOS. The first is the fact that we define lakes as perennial. However, seasonality can play a role in whether or not a feature is detected. If the lakes are digitized based on data acquired in a wet season or year, there will be many small features added to the NHD water bodies that are actually intermittent. The NHD's classification system allows for features to be categorized as intermittent, but few intermittent features are actually assigned to this class. To do so requires the editor to perform a comparative analysis using multiple images that are close in time from both dry and wet times of the year, which is impractical and in many cases impossible. Thus many of the smaller water bodies in the NHD are classified as perennial by default when in reality they are intermittent. The second important feature relates to watershed delineation. Incomplete representation of small lakes in the NHD may result in biased watershed delineation. For example, imagine that lakes between 1 and 4 ha are only partially digitized, but watersheds are still delineated for any lake > 1 ha. As shown in Table S16, if small lakes are only partially digitized, there are two possible scenarios that seem to share a roughly equal probability of occurrence for lakes in the small end of the size range (e.g., 1 to 4 ha). Errors in the watersheds of these small lakes would be compounded when watersheds are aggregated upstream.

**Table S16. Results from two possible scenarios in which lakes are incorrectly digitized**

| **Scenarios** | **Results** |
| --- | --- |
| Lake A has undigitized neighboring lakes | Watershed of Lake A will be too big |
| Lake A has neighboring non-lake polygons | Watershed of Lake A will be too small |

The consequences of incomplete digitization of small waterbodies call for selection of an optimum size cutoff. Choosing a larger lake size cutoff will not solve all of the problems associated with incomplete digitization however. For example, a larger lake minimum size for delineating watersheds will not necessarily increase the accuracy of the watershed delineations; only the complete digitization and verification of the smaller water bodies would achieve this. However, choosing a larger minimum lake size provides three primary advantages:

1. If small, undigitized, or erroneously digitized features are removed from consideration, each lake watershed delineation becomes comparable relative to the other lake watershed delineations of that size range. This means that values from one lake watershed can be more safely extrapolated to another lake watershed in the same size class.
2. Coarser 'seeding' (using fewer lakes in the delineation process) may provide better watersheds for the purposes of this study. These slightly larger lake watersheds would provide more reliable zonal statistics of geographical features such as land use, geology, etc. This is because a larger polygon will overlay more cells in a raster dataset. Each cell in a raster can be considered to be a sample. More sampling provides higher confidence in the summarized values. Additionally, the smallest lake watershed features could be completely covered by a single raster cell when analyzing coarser environmental datasets. Attributing summary data based on a single sample cell may cause relatively poor attribution of small features as it renders them vulnerable to statistical outliers.
3. By choosing a larger minimum lake size, many of the intermittent and non-lake features that are incorrectly defined as 'lake/pond' in the NHD are removed from the water bodies included.

## Error rates for lakes by minimum lake size

### Methods to calculate NHD error rates:

Below are the steps that were taken to quantify nominal error rates by state for four states in LAGOS for lakes ≥ 1 ha (WI, MI, IA, ME), and then for seven states in LAGOS for lakes ≥ 4 ha (WI, MI, IA, ME, MO, NH, OH).

1. Choose a minimum lake size, select all lakes that are greater than or equal to that minimum size.
2. Randomly choose 3 rectangles (100 km^2^) from each state to be tested.
3. Estimate the number of lakes within each rectangle through visual interpretation of the best available aerial imagery from ESRI, DigitalGlobe, GeoEye, i-cubed, USDA, USGS, AEX, Getmapping, Aerogrid, IGN, IGP, and swisstopo. (Compiled as a web-served base map in ArcGIS [10]).
4. Count the number of lakes in the NHD coverage.
5. Calculate the percentage of missing lakes in the NHD for each rectangle.
6. Calculate a state average as well as the four- or seven-state average.
7. The above steps were performed for minimum lake size of 1 and 4 ha (different random rectangles selected for each analysis).

### Results of calculated error rates

Figure S13 shows the percentage of lakes that were digitized in the NHD, averaged by state, and for the two different threshold values. For all lakes ≥ 1 ha, only 7 to 70% of lakes were correctly represented in the NHD, with an average of 42% (Figure S13, top panel). The percentage of lakes correctly represented appears to be substantially better for all lakes ≥ 4 ha for which the success rate increased to a range of 67 to 100 %, with an average of 87% (Figure S13, bottom panel). Based on these results, it is recommended that we use a 4 ha threshold for identification of lakes in LAGOS.


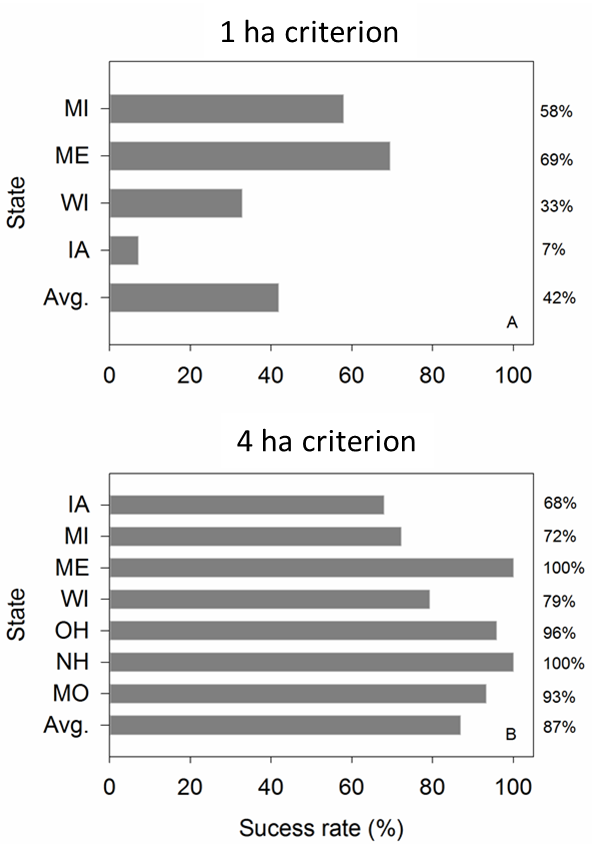


**Figure S13. The success rates for lakes (A) ≥ 1 ha and (B) ≥ 4 ha.**

## Summary of decisions regarding minimum lake size and LAGOS (Table S17)

- Lakes with limnological data (LAGOS_LIMNO_) that are < 1 ha will be deleted from LAGOS.
- All lakes ≥ 1 ha will be maintained in LAGOS_LIMNO_ with unique identifiers, but with no data in LAGOS_GEO_.
- All lakes ≥ 4 ha will have topographically based lake watersheds delineated for them.
- It is recommended that all extrapolations of results of the limnological data be applied to lakes ≥ 4 ha.

**Table S17. Summary of lake size implications for LAGOS_GEO_ calculations (i.e., land use/cover, climate, geology, lake type, etc.) for lakes in the 17-state study area**

| **Lake surface area** | **In LAGOS_GEO_** | **In LAGOS_LIMNO_** | **Watersheds delineated** | **Recommended population for extrapolation** |
| --- | --- | --- | --- | --- |
| Lakes < 1 ha | no | no | no | no |
| Lakes ≥ 1 ha and  Lakes ≤ 4 ha | no | yes | no | no |
| Lakes ≥ 4 ha | yes | yes | yes | yes |

# Lake classification

Lakes are classified in LAGOS_GEO_ following two different classification schemes: Lake hydrologic class and Lake order. Because LAGOS_GEO_ contains ~50,000 lakes, an automated tool was developed to categorize lakes by their hydrologic connectivity according to the above categories (see Additional file 8).

## Lake hydrologic class

This classification scheme uses information from the NHD on lake water bodies and stream flow direction to characterize lake landscape position and the relationship to upstream lakes and streams (Table S18). Lakes are classified by hydrologic class using the Lake Connectivity Classification tool (see Additional file 8 for more information).

## Lake order

In addition to Lake hydrologic type*,* we have also determined Lake order (Table S18). Lake order also classifies lake landscape position and uses similar methods as the Lake hydrologic type classification, but is based on slightly different relationships using the methods described in [11]. We used the NHD data on lake water bodies and stream flow direction and the NWI wetland data to classify lakes by Lake order. Lakes are classified by Lake order using the Lake Order Classification tool (see Additional file 8 for more information).

**Table S18. The two lake classification schemes and the class types based on connections between lakes and streams (and sometimes wetlands)**

| **Lake Classification** | **Classes** | **Definition** | **GIS Layer** |
| --- | --- | --- | --- |
| *Lake hydrologic class* | *Isolated* | Lakes with no inflow or outflow permanent streams* | NHD |
|  | *Headwater* | Lakes at the headwater of a stream network (i.e. no inflowing streams, but by definition, they DO have at least one outflowing stream) |  |
|  | *Drainage* | Lakes connected to surface water through inflowing streams, with no upstream lakes (≥10 ha), and may or may not have outflowing streams |  |
|  | *Drainage-UPLK* | Lakes connected to surface water through inflowing streams, with at least one upstream lake (≥10 ha), and may or may not have outflowing streams |  |
| *Lake order* | *Order -3* | Isolated (i.e., lakes with no inflow or outflow streams) | NHD |
|  | *Order -2* | Lakes with wetlands along lake perimeter | NWI |
|  | *Order -1* | Lakes connected by intermittent streams |  |
|  | *Order 0* | Headwater (i.e., no inflowing streams, but does have at least one outflowing stream) |  |
|  | *Order 1* | Lakes drained by a Strahler Order 1 stream |  |
|  | *Order X* | Lakes drained by a Strahler Order X stream (with X = 2 to the maximum stream order in study site) |  |

*Hereafter, all 'stream' indicate permanent streams, unless noted otherwise

### Results of error analysis of lake hydrological classification:

We estimated the error of the lake hydrologic classification by manually testing lakes within three different HUC8 regions in Michigan. We found very low error rates that were due primarily to errors in NHD digitization.

**Table S19. Results of the error analysis within 3 HUC8 regions in Michigan**

| **Watershed Evaluated** | **Watershed Code** | **#Lakes Evaluated** | **Errors Found** | **% Error** | **Source of Error** |
| --- | --- | --- | --- | --- | --- |
| Pere Marquette River Watershed | 409005 | 106 | 1 | 0.94% | Abnormal NHD Flowline Digitizing |
| Huron River Watershed | 4020202 | 102 | 1 | 0.98% | Abnormal NHD Flowline Digitizing |
| Tahquamenon River Watershed | 4060101 | 100 | 0 | 0.00% |  |
|  |  |  |  |  |  |
|  | Totals | 308 | 2 | 0.65% |  |

### Sources of Error:

Both errors that were found were ones in which lakes were incorrectly classified as 'Headwater' rather than 'Stream Drainage' or 'Lake-Stream Drainage'. Both errors were the result of incorrect digitization of the artificial flow lines by the NHD. These flow lines maintain hydrologic network connectivity as the network traverses a lake polygon. If there is a break or 'dangle' in the artificial flow line that should not be there, this tool can give a false positive for a Headwater lake classification. Because there are too many lakes to double check, we have accepted this very low error rate and will not fix any misclassifications as they are inherent in the base layer (i.e., the NHD) rather than errors of the tool. Thus, we argue that future updates to the NHD base layers should further improve accuracy in determining lake hydrologic types.

# References

1. US Geological Survey National Hydrography Dataset. http://nhd.usgs.gov/. Accessed 4 June 2015.

2. Zhang T, Soranno PA, Cheruvelil KS, Kramer DB, Bremigan MT, Ligmann-Zielinska A. Evaluating the effects of upstream lakes and wetlands on lake phosphorus concentrations using a spatially-explicit model. *Landsc Ecol.* 2012; 27:1015–1030.

3. Soranno PA. Spatial Variation among Lakes within Landscapes: Ecological Organization along Lake Chains. *Ecosystems.* 1999; 2:395–410.

4. Martin SL, Soranno PA. Lake landscape position: relationships to hydrologic connectivity and landscape features. *Limnol Oceanogr.* 2006; 51:801–814.

5. Seaber P, Kapinos F, Knapp G. Hydrologic unit maps: US Geological Survey water-supply paper 2294. 1987. http://water.usgs.gov/GIS/huc.html. Accessed 4 June 2015.

6. US Geological Survey water resources of the United States. http://water.usgs.gov/GIS/huc.html. Accessed 4 June 2015.

7. US Environmental Protection Agency: EPA launches national lake survey. http://water.epa.gov/type/lakes/assessmonitor/lakessurvey/outreach/wisconsin.cfm. Accessed 4 June 2015.

8. US Environmental Protection Agency: National lakes assessment fact sheet. 2010. http://water.epa.gov/type/lakes/upload/nla_survey_fact_sheet.pdf. 4 June 2015. Accessed 4 June 2015

9. US Environmental Protection Agency: National lakes assessment 2012: a fact sheet for communities. 2012. http://water.epa.gov/type/lakes/assessmonitor/lakessurvey/upload/NLA-2012-Fact-Sheet-for-Communities.pdf. Accessed 4 June 2015.

10. ArcGIS Map Service: World Imagery. 2014. http://goto.arcgisonline.com/maps/World_Imagery. 4 June 2015.

11. Riera JL, Magnuson JJ, Kratz TK, Webster KE. A geomorphic template for the analysis of lake districts applied to the Northern Highland Lake District, Wisconsin, US. *Freshw Biol.* 2000; 43:301–318.

# Appendix 1

**Table S20. NHD Geodatabase Download Dates**

| **Download Date** | **NHD Pre-staged High Resolution Geodatabse** |
| --- | --- |
| 3/27/2013 | NHDH0102_931v210.zip |
| 3/27/2013 | NHDH0103_931v210.zip |
| 3/27/2013 | NHDH0104_931v210.zip |
| 3/27/2013 | NHDH0105_931v210.zip |
| 3/27/2013 | NHDH0401_931v210.zip |
| 3/27/2013 | NHDH0402_931v210.zip |
| 3/27/2013 | NHDH0403_931v210.zip |
| 3/27/2013 | NHDH0404_931v210.zip |
| 3/27/2013 | NHDH0405_931v210.zip |
| 3/27/2013 | NHDH0406_931v210.zip |
| 3/27/2013 | NHDH0407_931v210.zip |
| 3/27/2013 | NHDH0408_931v210_sdeGridIdx.zip |
| 3/27/2013 | NHDH0409_931v210.zip |
| 3/27/2013 | NHDH0410_931v210.zip |
| 3/27/2013 | NHDH0508_931v210.zip |
| 3/27/2013 | NHDH0512_931v210.zip |
| 3/27/2013 | NHDH0514_931v210.zip |
| 3/27/2013 | NHDH0703_931v210.zip |
| 3/27/2013 | NHDH0704_931v210.zip |
| 3/27/2013 | NHDH0705_931v210.zip |
| 3/27/2013 | NHDH0706_931v210.zip |
| 3/27/2013 | NHDH0707_931v210.zip |
| 3/27/2013 | NHDH0708_931v210.zip |
| 3/27/2013 | NHDH0709_931v210.zip |
| 3/27/2013 | NHDH0712_931v210.zip |
| 4/5/2013 | NHDH0411_931v210.zip |
| 4/5/2013 | NHDH0414_931v210.zip |
| 4/5/2013 | NHDH0503_931v210.zip |
| 4/5/2013 | NHDH0504_931v210.zip |
| 4/5/2013 | NHDH0506_931v210.zip |
| 4/5/2013 | NHDH0507_931v210.zip |
| 4/5/2013 | NHDH0509_92v200.zip |
| 4/5/2013 | NHDH0701_931v210.zip |
| 4/5/2013 | NHDH0702_931v210.zip |
| 4/5/2013 | NHDH0710_931v210.zip |
| 4/5/2013 | NHDH0711_931v210.zip |
| 4/5/2013 | NHDH0713_931v210.zip |
| 4/5/2013 | NHDH0714_931v210.zip |
| 4/5/2013 | NHDH0802_931v210.zip |
| 4/5/2013 | NHDH0902_931v210.zip |
| 4/5/2013 | NHDH0903_931v210.zip |
| 4/5/2013 | NHDH1017_931v210.zip |
| 4/5/2013 | NHDH1023_931v210.zip |
| 4/5/2013 | NHDH1024_931v210.zip |
| 4/5/2013 | NHDH1027_931v210.zip |
| 4/5/2013 | NHDH1028_92v200.zip |
| 4/5/2013 | NHDH1029_931v210.zip |
| 4/5/2013 | NHDH1030_931v210.zip |
| 4/5/2013 | NHDH1101_931v210.zip |
| 4/5/2013 | NHDH1107_931v210.zip |
| 4/8/2013 | NHDH0501_931v210.zip |
| 4/8/2013 | NHDH0502_931v210.zip |
| 4/8/2013 | NHDH0509_931v210.zip |
| 4/9/2013 | NHDH0106_931v210.zip |
| 4/9/2013 | NHDH0107_931v210.zip |
| 4/9/2013 | NHDH0108_931v210.zip |
| 4/9/2013 | NHDH0109_931v210.zip |
| 4/9/2013 | NHDH0110_931v210.zip |
| 4/9/2013 | NHDH0111_92v200.zip |
| 4/9/2013 | NHDH0202_931v210.zip |
| 4/9/2013 | NHDH0203_931v210.zip |
| 4/9/2013 | NHDH0204_931v210.zip |
| 4/9/2013 | NHDH0205_931v210.zip |
| 4/9/2013 | NHDH0206_931v210.zip |
| 4/9/2013 | NHDH0207_931v210.zip |
| 4/9/2013 | NHDH0412_931v210_sdeGridIdx.zip |
| 4/9/2013 | NHDH0413_931v210.zip |
| 4/9/2013 | NHDH0415_931v210_sdeGridIdx.zip |
| 4/9/2013 | NHDH0801_931v210.zip |
| 4/15/2013 | NHDH0101_931v210.zip |
| 4/15/2013 | NHDH0415_92v200_sdeGridIdx.zip |
