# Supplementary material for: Building a multi-scaled geospatial temporal ecology database from disparate data sources: fostering open science and data reuse
Source: Gigascience. 2015 Jul 1;4:28. doi: 10.1186/s13742-015-0067-4 (PMC4488039; doi:10.1186/s13742-015-0067-4)
Supplement: Additional file 10: — Data sources, definition, and classification of rivers and streams. Detailed description of the sources, definitions, and classifications that were used to describe rivers and streams in LAGOS. [file 13742_2015_67_MOESM10_ESM.docx]

Additional file 10

**Data sources, definition, and classification of rivers and streams**

Nicole Smith, Patricia Soranno, Scott Stopyak, Ed Bissell

### **OVERVIEW**

This document describes how we created the stream data layer that we used to calculate lake connectivity metrics, stream density, and wetland order for the LAGOS database. The stream GIS layer was created to represent riverine features as recorded in the NHDFlowline data layer [1]. Stream order classifications were assigned using the Strahler stream order. There are approximately 2.7 million km of streams in the 17 state study area of LAGOS. The data layers created with this process can be used for mapping and for density calculations, but for any tool requiring network analysis (e.g., Upstream Lakes, Lake Connectivity Classification, Wetland Order, and all of the watershed tools calling for flowline data), it will be necessary to perform the analysis for each subregion using that subregion’s NHDFlowline feature class.

**Data sources**

See NHD documentation (Additional file 9). The fields NHDFlowline and NHDArea were used during the processing of these data.

**Definition of a 'Stream'**

Six classes of features were retained in our Streams layer (Table S21).

**Table S21. Total length of streams by feature type contained in our Streams layer**

| **FType** | **Feature type** | **Total length (km)** |
| --- | --- | --- |
| 460 | Stream/River | 2,376,958 |
| 558 | Artificial Path | 187,754 |
| 336 | Canal/Ditch | 147,490 |
| 334 | Connector | 15,823 |
| 428 | Pipeline | 3,478 |
| 420 | Underground Conduit | 1,394 |
|  | TOTAL | 2,732,897 |

We excluded coastlines (FType = 566) and artificial paths that did not spatially intersect NHDArea polygons with the StreamRiver FType. NHDArea includes polygon-based representations of large rivers that have two banks digitized. Artificial lines contained in these polygons are centerlines that are line-based representations of these rivers. Our tools rely on a line-based representation of rivers and streams in order to calculate total length inside the regions of interest (i.e., the spatial extents for which we summarize the data, which we call zones and which include lake watersheds, HU12, HU4, and other extents).

**Stream classification: Strahler stream order**

Strahler stream order is a stream-reach classification scheme based on methods developed by Strahler [2]. Stream order uses information from the NHD on stream flow direction to characterize the position of the stream reach within the stream network. In general, stream order increases as one moves down a stream network starting from the headwater stream to the terminal point. More specifically Strahler order 1 streams are defined as "the smallest finger-tip tributaries" [2]. Order 2 streams are where two order 1 streams intersect. Order 3 streams are where two order 2 streams intersect, etc.

Stream order classifications were assigned using the Strahler numbers calculated by the RivEx 10.6 GIS tool [3]. We grouped the stream order classes into three categories: headwater, mid-reach, and river. This was done because there is uncertainty in the identification of low order streams derived from the NHD data (based on expert opinion).

- Headwater streams = 1st to 3rd order streams.
- Mid-reach streams = 4th to 6th order streams.
- River = > 6th order streams.

### **Data processing steps to create the Streams data layer**

***1. Project and save NHDFlowline data***

NHDFlowlines were projected into the USGS Albers coordinate system and saved into shapefiles.

***2. Assign Strahler Stream Order to NHDFlowline data***

We used the RivEX extension for ArcGIS 10.1 to calculate the Strahler stream order number for each feature of the NHDFlowline shapefiles. We chose to calculate Strahler numbers before excluding features in order to maintain network connectivity.

RivEX software, a river network tool designed to run within ESRI ArcGIS, is proprietary, reasonably priced, and available for download [3]. RivEX requires the following specifications of the data, which are met by the NHDFlowline layers:

• Your dataset must be a vector dataset.

• Your vector dataset must be of polyline (arcs) feature type.

• Polylines must be connected to each other only at their ends (nodes).

• Polylines must be digitized in a source (From) to mouth (To) direction.

• The polyline represents the center line of the river and not the banks.

• Lakes and reservoirs should be reduced to center lines so that a connected network is formed.

The RivEX manual describes the algorithm to calculate Strahler order:

RivEX implements a fast recursive algorithm to calculate Strahler stream order, stream segments and can deal with multi-channeled networks. The algorithm that RivEX implements was developed by Gleyzer *et al.* [4].

Fields added to the NHDFlowline layer as a result of the Strahler order calculation:

- TNode, FNode, Enabled: Fields added while establishing the river network input to RivEX.
- Strahler: The Strahler stream order number of the digitized line segment. This value varies from 1 (low order) to 8 (high order), with 0 reserved for undetermined value as a result of the stream segment being flipped (polyline erroneously points from mouth to source direction).
- Segment: Each Strahler reach is assigned a new, unique segment number by the tool. Because our streams were processed at the subregion level, segment numbers do not repeat within a given subregion, but they do repeat within the master national layer.

***3. Merge data spatially for entire study area***

a. Following the Strahler order calculation, streams were merged into a study-wide layer. Two selections were performed to produce the final master Streams layer based on FCodes (see Table S22 for a complete list):

1. Exclude coastlines (FCode 566). The NHD includes coastlines as a part of the flow network; however, we choose to represent only riverine features in our final layer.
2. Exclude some artificial paths (FCode 558). The NHD uses artificial paths in a variety of situations to maintain flow through the network. First, it uses artificial paths to represent flow through lakes, reservoirs, and other features also represented in NHDWaterbody. Therefore, these paths were excluded from the Streams layer. It also uses artificial paths to represent the centerline of large, double-banked streams that are also represented by polygons in NHDArea. We use the line-based representations of these streams in our total length and stream density calculations, so we retained the latter category of artificial paths. This was performed using the following steps:
3. Merge all the NHDArea layers in our study region.
4. Select only NHDArea polygons with StreamRiver FType ('StreamRiver Areas ONLY').
5. Split Streams into NOT FCode = 558 ('NO Artificial Paths') and FCode = 558 ('Artificial Paths ONLY').
6. Select 'Artificial Paths ONLY' that INTERSECT 'StreamRiver Areas ONLY.
7. Merge 'NO Artificial Paths' and the result of step (d). The final result is the Streams layer.

b. The Streams layer was used to create several subset layers.

1. First, Canal_Ditches_ONLY was created by selecting the Streams features with FCodes 33600, 33601, 33603 ('Canal/Ditch'). We used this layer as the input in the creation of values for the [zone]_canalditchdensity_sum_ lengthm and [zone]_canalditchdensity_densitymperha fields.
2. Second, Strahler order values were consolidated into three classes: 'Headwaters' (1-3 order); 'Mid-reaches' (4-6 order), and 'Rivers' (>6 order). The justifications for reducing the number of classes are the following based on expert opinion:
   1. Reducing the number of classes to three categories saves GIS processing time.
   2. By grouping stream order we should not lose important geomorphological information because (a) it is recognized that Strahler Stream Order is not precise in characterizing stream reaches (e.g., a 2nd order stream in WI may look very different from a 2nd order stream somewhere else) and (b) the resolution of the NHD may lead to uncertainty in what was identified as a '1st order' stream.

**Error analysis of the streams geodata**

***Resolution of NHD Flowline data***

The NHDFlowLine data exhibit uneven digitization resolution for the lowest-order streams (Strahler order number = 1). In Figure S14, the boundaries at the HU8 level are very obvious in the streams layer due to more intensive digitization in some HU8s compared to others.

**
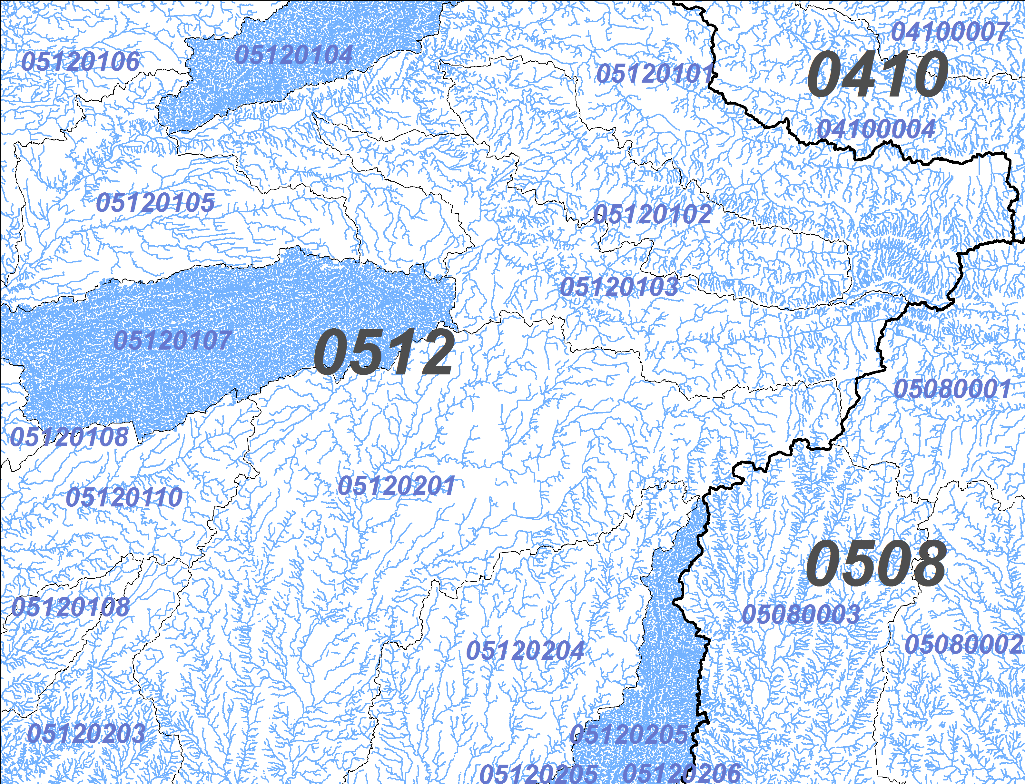
**

**Figure S14. Example of uneven digitization of low-order streams (Strahler Order 1) across HU8 areal extents.** Note the high density in the HUs: 05120107, 05120104 and 05120205.

***Unassigned Strahler order stream reaches (Order 0)***

Approximately 1,142 km of stream length within the LAGOS Study_Extent area have Strahler order 0 assigned because the polylines were digitized backwards in the NHD and not corrected by hand within our project. This is a small source of error, about 0.04% of the overall stream length. This error causes the sum of Headwaters + Midreaches + Rivers statistics to not exactly equal the total Streams based statistics.

***Coastlines were assigned orders***

Coastlines received a range of Strahler order values. Coastlines were almost always the highest-order part of a network so their removal prior to Strahler number calculation did not often change the Strahler number of other segments. Visual inspection was used to confirm that this situation was rare.

***Uncertainty with assigning Strahler order to artificial channels***

RivEX notes a warning: "To apply stream ordering on artificial grid-like channels can produce some very strange results and should be interpreted with extreme caution." Channels such as these are found in the NHD and were not removed.

***Strahler order numbering restarts at subregion boundaries***

Because the RivEX Strahler order calculations were performed using individual subregion files,

the numbering 'restarts' at subregion boundaries. In other words, along boundaries, Strahler number 8 streams can flow into Strahler number 1 streams that border boundaries (Figure S15).


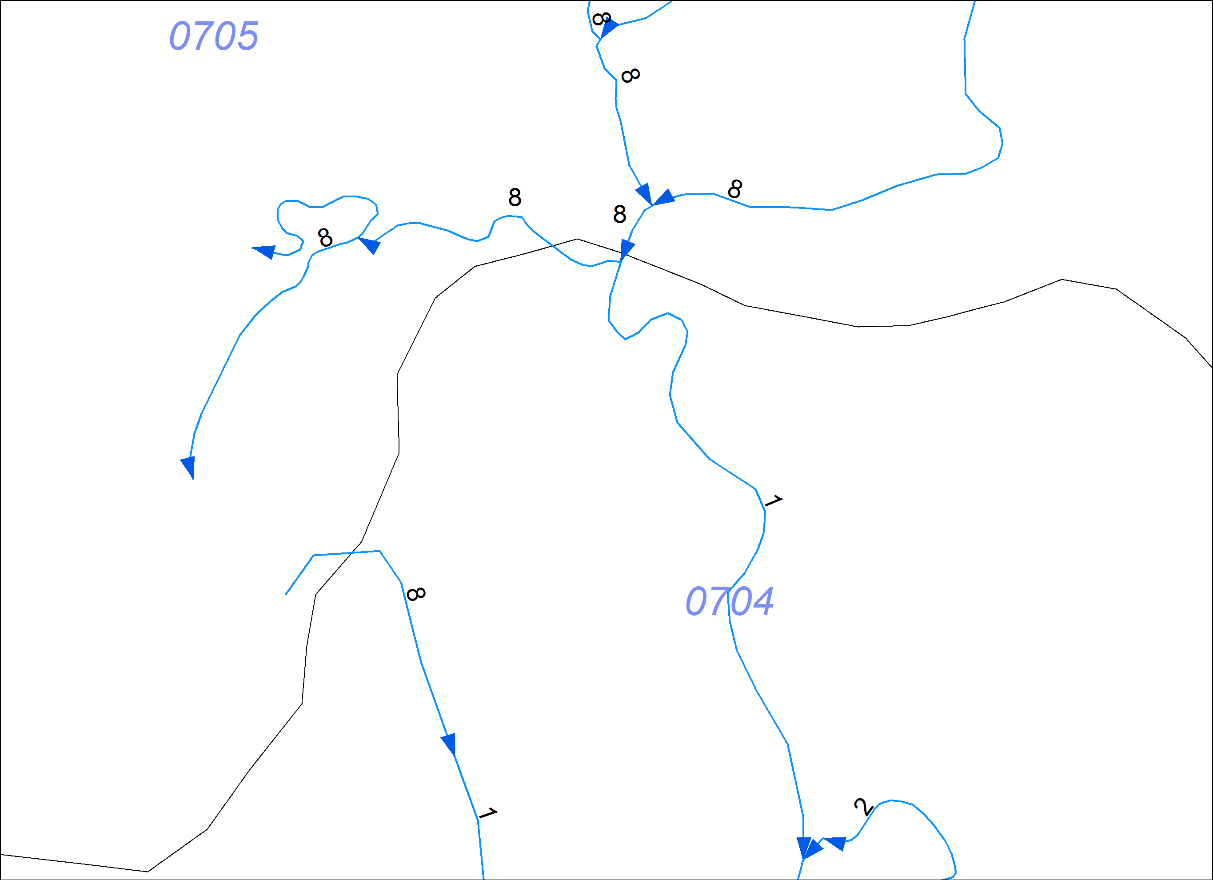


**Figure S15. An example showing potential problems with the RivEX tool at region boundaries.**

### **Additional warnings:**

The Streams layer does not represent a complete flow network chiefly due to the removal of some types of artificial paths. It cannot, for instance, be used as an input to RivEX. If a complete flow network is required, the analyst should start with the NHD geodatabases.

### **Table S22. NHDFlowline FCodes (from [5])**

| **Feature Type** | **FCode** | **Description** |
| --- | --- | --- |
| ARTIFICIAL PATH | 55800 | feature type only: no attributes |
| CANAL/DITCH | 33600 | feature type only: no attributes |
| CANAL/DITCH | 33601 | Canal/Ditch Type\|aqueduct |
| CANAL/DITCH | 33603 | Canal/Ditch Type\|stormwater |
| COASTLINE | 56600 | feature type only: no attributes |
| CONNECTOR | 33400 | feature type only: no attributes |
| PIPELINE | 42800 | feature type only: no attributes |
| PIPELINE | 42801 | Product\|water; Pipeline Type\|aqueduct; Relationship to Surface\|at or near |
| PIPELINE | 42802 | Product\|water; Pipeline Type\|aqueduct; Relationship to Surface\|elevated |
| PIPELINE | 42803 | Product\|water; Pipeline Type\|aqueduct; Relationship to Surface\|underground |
| PIPELINE | 42804 | Product\|water; Pipeline Type\|aqueduct; Relationship to Surface\|underwater |
| PIPELINE | 42805 | Product\|water; Pipeline Type\|general case; Relationship to Surface\|at or near |
| PIPELINE | 42806 | Product\|water; Pipeline Type\|general case; Relationship to Surface\|elevated |
| PIPELINE | 42807 | Product\|water; Pipeline Type\|general case; Relationship to Surface\|underground |
| PIPELINE | 42808 | Product\|water; Pipeline Type\|general case; Relationship to Surface\|underwater |
| PIPELINE | 42809 | Product\|water; Pipeline Type\|penstock; Relationship to Surface\|at or near |
| PIPELINE | 42810 | Product\|water; Pipeline Type\|penstock; Relationship to Surface\|elevated |
| PIPELINE | 42811 | Product\|water; Pipeline Type\|penstock; Relationship to Surface\|underground |
| PIPELINE | 42812 | Product\|water; Pipeline Type\|penstock; Relationship to Surface\|underwater |
| PIPELINE | 42813 | Product\|water; Pipeline Type\|siphon; Relationship to Surface\|unspecified |
| PIPELINE | 42814 | Product\|water; Pipeline Type\|general case |
| PIPELINE | 42815 | Product\|water; Pipeline Type\|penstock |
| PIPELINE | 42816 | Product\|water; Pipeline Type\|aqueduct |
| STREAM/RIVER | 46000 | feature type only: no attributes |
| STREAM/RIVER | 46003 | Hydrographic Category\|intermittent |
| STREAM/RIVER | 46006 | Hydrographic Category\|perennial |
| STREAM/RIVER | 46007 | Hydrographic Category\|ephemeral |
| UNDERGROUND CONDUIT | 42000 | feature type only: no attributes |
| UNDERGROUND CONDUIT | 42001 | Positional Accuracy\|definite |
| UNDERGROUND CONDUIT | 42002 | Positional Accuracy\|indefinite |
| UNDERGROUND CONDUIT | 42003 | Positional Accuracy\|approximate |

**References**

1. US Geological Survey National Hydrography Dataset. http://nhd.usgs.gov/.

2. Strahler AN. Hypsometric (area-altitude) analysis of erosional topography. *Geol Soc Am Bull.* 1952; 63:1117.

3. Hornby D. RivEx (Version 10.6). http://www.rivex.co.uk/.

4. Gleyzer A, Denisyuk M, Rimmer A, Salingar Y. A fast recursive GIS algorithm for computing Strahler stream order in braided and nonbraided networks1. *JAWRA J Am Water Resour Assoc.* 2004; 40::937–946.

5. US Geological Survey: Complete FCode list for NHD hydrography features. http://nhd.usgs.gov/userGuide/Robohelpfiles/NHD_User_Guide/Feature_Catalog/Hydrography_Dataset/Complete_FCode_List.htm.
