# Supplementary material for: Building a multi-scaled geospatial temporal ecology database from disparate data sources: fostering open science and data reuse
Source: Gigascience. 2015 Jul 1;4:28. doi: 10.1186/s13742-015-0067-4 (PMC4488039; doi:10.1186/s13742-015-0067-4)
Supplement: Additional file 11: — Data sources, definition, and classification of wetlands. Detailed description of the sources, definitions, and classifications that were used to describe wetlands in LAGOS. [file 13742_2015_67_MOESM11_ESM.docx]

Additional file 11

**Data sources, definition, and classification of wetlands**

Nicole Smith, Emi Fergus, Nicholas Skaff, Patricia Soranno, Scott Stopyak, Ed Bissell

**OVERVIEW**

This document describes the process and definitions used to classify wetlands in the LAGOS_GEO_ database. We used wetland data from the National Wetlands Inventory (NWI), which was created from image analysis of high altitude imagery and on-the-ground verification to create vector-based wetland delineations. NWI classifies each wetland on the basis of local vegetation, visible hydrology, and geography according to the federal standard for wetland classifications developed by Cowardin *et al.* [1]. They also demarcate both traditional wetlands and deep-water habitats. However, we omitted deep-water systems (riverine and lacustrine systems) because they are already represented in the NHD and further, we used a GIS overlay analysis to erase lake regions from the wetlands that we retained. We also omitted several classes of wetlands, keeping only palustrine systems, which are defined as non-tidal wetlands dominated by trees, shrubs, and persistent emergent vegetation. We then developed new metrics describing wetland connectivity to streams and lakes by combining information from both the NWI and NHD databases (see Additional files 9 and 10). Finally, we simplified the wetland vegetation and wetland water regime classifications in NWI to more specifically suit the needs of our project. As a results of these modifications, LAGOS_GEO_ wetlands are classified according to: 1) wetland spatial-connection with streams (Table S23) and lakes (Table S24); and 2) wetland vegetation composition (Table S25).

**National Wetlands Inventory Dataset**

*Download date*: August 29, 2014

*Download URL*: [2]

**Wetland Classifications**

**Wetland spatial connectivity classification**

Wetland polygons (NWI palustrine systems only) were classified based on their spatial relationship with NHD streams using the 'Wetland Order tool a' (LAGOS GIS Toolbox, Additional file 8). Wetland polygons were classified as connected, singly connected, or isolated from NHD streams (Table S23). Wetlands were considered connected to streams if they were within a 30 m buffer surrounding the stream reach. The 30 m buffers were used to alleviate limitations associated with GIS resolution and misalignment between the different GIS layers (NHD and NWI). Stream flow direction was not taken into consideration in the classification.

**Table S23.** Wetland-stream connected classification where NWI wetland polygons are classified based on their spatial configuration with NHD streams

| **Wetland-Stream Connected Classes** | **Definition** |
| --- | --- |
| *Isolated* | Wetlands with no intersecting streams within a 30 m buffer of the wetland |
| *Single* | Wetlands intersected within a 30 m buffer by a single first order stream |
| *Connected* | Wetlands intersected within a 30 m buffer by a higher order stream or by multiple streams |

**Wetland vegetation composition classification**

In the NWI, wetland polygons are classified into fine-scale vegetation classes (e.g., Scrub-shrub Broad-leaved Deciduous wetland) based on the dominant vegetation structure interpreted from aerial imagery. Many of these classes are not explicitly linked to ecological processes of interest and there is a question regarding the accuracy of these classes given the limitations of aerial imagery interpretation. Therefore, we applied the higher-level NWI vegetation classes to classify all wetland polygons as one of three possible categories: *Open water wetland*, *Scrub-shrub wetland*, or *Forested wetland* (Table S24).

**Table S24.** Wetland vegetation classifications based on NWI Cowardin *et al.* classification [1]

| **Wetland Vegetation Classification** | **Definition** | | **Original NWI attributes** |  |
| --- | --- | --- | --- | --- |
| *Open water wetland* | Wetlands with open water habitat | Combines 'Aquatic bed' and 'Emergent wetland' classes | | |
| *Scrub-shrub wetland* | Wetlands dominated by woody vegetation < 6 m tall | All 'Scrub-shrub' wetland classes | | |
| *Forested wetland* | Wetlands dominated by woody vegetation 6 m or taller | All 'Forested' wetland classes | | |

**Wetland water regime classification**

In the NWI, wetland polygons are assigned water regime modifiers that aim to provide "a gross description of a site's water regime, the frequency and duration of surface water inundation or soil saturation" [3]. We focus on the modifiers that fall within the Non-tidal Water Regime category, which include: *temporarily flooded, saturated, seasonally flooded, seasonally flooded/saturated,* *semi-permanently flooded, intermittently exposed, and permanently flooded* (Table S25) [4].

**Table S25. Wetland (non-tidal) water regime classifications based on NWI Cowardin classification**

| **Wetland Water Regime Classification** | **Definition** |
| --- | --- |
| 1. *Temporarily flooded* | Surface water is present for brief periods during the growing season, but the water table usually lies well below the soil surface for most of the season. Plants that grow both in uplands and wetlands are characteristic of this water regime. |
| 1. *Seasonally flooded* | Surface water is present for extended periods especially early in the growing season but is absent by the end of the season in most years. The water table after flooding ceases is variable, extending from saturated to the surface to a water table well below the ground surface. |
| 1. *Semi-permanently flooded* | Surface water persists throughout the growing season in most years. When surface water is absent, the water table is usually at or very near the land surface. |
| 1. *Intermittently exposed* | Surface water is present throughout the year except in years of extreme drought. |
| 1. *Permanently flooded* | Water covers the land surface throughout the year in all years. Vegetation is composed of obligate hydrophytes. |

**Accuracy**

Based on the currently available aerial imagery, NWI analysts were able to identify wetlands as small as 202 m^2^, although the database is more accurate at larger sizes and identifies at least 98% of wetlands greater than 2,023 m^2^ (0.05 acres; [5]). Finally, according to NWI quality standards, at least 85% of wetlands are classified correctly according to the Cowardin *et al.* [1]. However, there are questions regarding the accuracy of water regime classifications in some regions. One study evaluating NWI classifications using field surveys found water regime designation to be relatively accurate for the wettest and driest regimes in California with 81% agreement for 'permanently flooded' sites and 56% agreement for 'temporarily flooded' sites [5]. We are not aware of any accuracy analyzes in the Midwestern or Northeastern United States.

**Additional sources of information**

USGS. Federal Standards and Procedures for the National Watershed Boundary Dataset (WBD). Chapter 3 of Section A, Federal Standards, Book 11, Collection and Delineation of Spatial Data. *USGS Techniques and Methods. 4th e).* 2013; 11–A3.

US Fish and Wildlife Service, Division of Habitat and Resource Conservation**.** [National Standards and Quality Components for Wetlands, Deepwater and Related Habitat Mapping](http://www.fws.gov/stand/standards/dl_wetlands_National%20Standards.doc). *Branch of Habitat Assessment, Arlington, VA* 2004.

**References**

1. Cowardin LM, Carter V, Golet FC, LaRoe ET. *Classification of Wetlands and Deepwater Habitats of the United States*. Washington, D. C.: US Department of Interior, Fish and Wildlife Service, Office of Biological Services. 1979; FWS/OBS-79/31

2. US Fish and Wildlife Service National Wetlands Inventory: Download Seamless Wetlands Data. http://www.fws.gov/wetlands/data/Data-Download.html.

3. Cowardin LM, Golet FC. US Fish and Wildlife Service 1979 Wetland Classification: A Review. *Vegetatio.* 1995; 118:139–152.

4. US Fish and Wildlife Service: Wetland mapping training. www.fws.gov/habitatconservation/nwi/wetlands_mapping_training.

5. Werner HW. Accuracy assessment of National Wetland Inventory maps at Sequoia and Kings Canyon National Parks. *Park Sci.* 2004; 23:19–22.
