# Supplementary material for: Building a multi-scaled geospatial temporal ecology database from disparate data sources: fostering open science and data reuse
Source: Gigascience. 2015 Jul 1;4:28. doi: 10.1186/s13742-015-0067-4 (PMC4488039; doi:10.1186/s13742-015-0067-4)
Supplement: Additional file 12: — Freshwater connectivity and composition metrics. Detailed description and justification of the freshwater connectivity and composition metrics that we developed for LAGOS. The metrics were designed to quantify the spatial heterogeneity in lake, stream, and wetland abundance and surface hydrologic connectivity at a range of spatial extents. [file 13742_2015_67_MOESM12_ESM.docx]

# Additional file 12

# **Freshwater connectivity and composition metrics**

Emi Fergus, Patricia Soranno, Kendra Cheruvelil, Samantha Oliver, Nick Skaff, Katherine Webster, Mary Tate Bremigan, Nicole Smith, Scott Stopyak, Ed Bissell

**OVERVIEW**

Our two main goals in creating freshwater spatial metrics for LAGOS were: 1) to develop landscape metrics that quantify spatial heterogeneity both in freshwater feature abundance (including classes of lakes, wetlands, and streams) and surface hydrological connections among freshwater features in the landscape at a range of spatial extents, and 2) to evaluate the performance of the metrics in capturing variation in lake response variables, such as lake nutrients. We classified freshwater features and developed our abundance and connectivity metrics using geographic information systems (GIS) and layers on lakes, streams, wetlands, and topography compiled at the US national and state level. Using these base GIS layers, we first classified individual lakes, streams, and wetlands using classification schemes based on hydrologic-type attributes that we hypothesized to be relevant to freshwater ecological processes. We then quantified composite metrics of several spatial extents (see Additional file 7) that characterize the abundance and connectivity of freshwater features in the landscape. More specifically, we first used a patch-based approach to calculate freshwater abundance metrics for groups of lakes, rivers, and wetlands. Second, we used a hybrid approach (integrating patch-based and graph-based approaches) to calculate freshwater configuration (i.e., connectivity) measures for the entire freshwater feature population (e.g., for all lakes), for each hydrologic class (e.g., for isolated, headwater, and drainage lakes), and for connections between features types (e.g., between lakes and streams). This document describes each of the freshwater connectivity and composition metrics that we developed for LAGOS_GEO_.

**Background**

Landscape metrics (also called indices) are spatial representations that quantify landscape heterogeneity and allow one to relate pattern to process, a central tenet of landscape ecology [1]. There are many examples of studies that have developed relationships between landscape metrics and freshwater response variables, such as water chemistry and biotic community indices. These studies have often focused on landscape metrics that 1) quantify features that only represent the terrestrial landscape (e.g., forest and agriculture) and 2) quantify the bulk composition of the landscape, such as the area of a particular land use near a water body. However, these traditional metrics do not account for the spatial arrangement of freshwater features, and thus ignore major functional components of the landscape.

The spatial arrangement and connections among networks of freshwater features, encompassing lakes, wetlands, streams, and groundwater, are important to consider when examining ecological flows and processes in freshwater systems. Detailed measures of hydrologic connectivity, largely developed in the fields of hydrology and engineering, are often site specific, data intensive, and based on quantifying connectivity at fine spatial scales (e.g., hillslopes) with the result that the connectivity measures cannot be easily scaled up to broader spatial extents. There is a need for general freshwater connectivity metrics that explicitly recognize freshwater features in the landscape and that characterize their spatial connections (at multiple and broad spatial extents when possible). General freshwater connectivity metrics provide the means to compare and characterize hydrological connectivity across different landscapes, recognize how freshwater features interact with one another and the surrounding terrestrial landscape to affect lake response variables, and more generally, better understand the influence of connectivity on ecological processes.

**Approaches to quantify landscape heterogeneity**

There are three common approaches used to characterize spatial heterogeneity in the landscape that have been developed for terrestrial systems: 1) patch-mosaic metrics, 2) spatial graphs, and 3) point-based geostatistics [1]. We developed our freshwater connectivity metrics using the patch-mosaic approach and a hybrid approach that combines the patch-mosaic and spatial-graph approaches (e.g., graph-based patch metrics) [2]. Table S26 summarizes these three approaches and our hybrid approach.

**Table S26. Summary of approaches to characterize spatial heterogeneity of the landscape**

| **Approach** | **Description and Assumptions** | **Pros and cons** |
| --- | --- | --- |
| *Common* |  |  |
| Patch-based metrics | Traditional view of the landscape where spatial heterogeneity is viewed as discrete, relatively homogenous patches that exhibit relatively abrupt transitions to adjacent areas [1]. | Pros: Conceptual view is intuitive, easy to interpret, and well suited to apply to management actions.  Cons: Patch-based metrics may not be well linked to ecological phenomena. Patch boundaries may be arbitrarily defined. Patch metrics ignore spatial variation within the spatial units. |
| Spatial graphs | Spatial graphs are mathematical constructs of space using nodes (points) and links (lines) to model topology and connectivity. Nodes and links can be spatially explicit, where nodes have location and links have distance and direction assigned to them [2]. | Pros: A network-based framework that explicitly quantifies connections among landscape components.  Cons: Spatial graphs are sensitive to missing nodes. The conceptual foundations of such tools are somewhat abstract and not well linked to ecological phenomena to date. |
| Point-based geostatistics | Variation in landscape features is represented by continuous gradients across space rather than discrete patches. With continuous values one can calculate geostatistics, such as quantifying spatial autocorrelation and identifying scales of interest. | Pros: Does not require classification of patches or defining boundary lines. Well suited to variables such as ecosystem rates.  Cons: Requires frequent point sampling across space. The density of sample points affects spatial properties, and so are less suited to landscape features that are not well distributed spatially, such as freshwater systems. |
| *Hybrid* |  |  |
| Graph-based patch metrics | Hybrid of patch-mosaic metrics and spatial graphs that explicitly incorporates topology to characterize landscape patches. | Pros: Identifies patches based on underlying topology of freshwater features that explicitly takes into account the arrangement and flow direction of freshwater features.  Cons: Metrics are subject to the same assumptions of patch-based mosaic metrics. Patches have to be classified, which can be difficult to define. |

**GIS base layers for metric development**

The GIS base layers used to develop the freshwater connectivity metrics come from national coverages that are freely available for download online: the National Hydrography Dataset (NHD), the National Wetlands Inventory (NWI), and the National Elevation Dataset (NED) (see Additional File 5 for characteristics of these GIS layers).

**LAGOS_GEO_ freshwater classification schemes**

We first classified lakes, streams, and wetlands in LAGOS_GEO_ to group these freshwater features by attributes we hypothesize to be important to lake ecological processes. Some of these classification schemes are based on established classifications (e.g., Strahler stream order) while we developed other schemes to address specific research questions. Freshwater features in LAGOS_GEO_ are categorized by their spatial arrangement with one another (e.g., Strahler stream order), their spatial arrangement with other freshwater features (e.g., lake hydrologic type, lake order, stream-connected wetland, and lake-connected wetland), and/or by their vegetation composition (e.g., wetland vegetation).

The freshwater classifications in LAGOS_GEO_ are intended to capture specific aspects of hydrology relevant to the system being characterized and range conceptually from those that are explicit in their relationship with hydrology to those that are implicit. For example, *wetland water regime* is a hydrologically-explicit class that groups wetlands based on duration of water inundation. A less explicit scheme would be *lake hydrologic type* that groups lakes based on the presence or absence of surface-stream connections and upstream lakes. This latter classification recognizes spatial connections among lakes and streams but does not take into account the magnitude of water flow among these connections. A hydrologically implicit class is *wetland vegetation* that classifies wetland polygons by their dominant vegetation structure. Although this class does not directly recognize hydrologic characteristics, wetland vegetation is sensitive to water inundation duration and, therefore, acts as an indirect indicator of wetland hydrology.

***Lake classification***

Lakes are classified in LAGOS_GEO_ following two different classification schemes: *Lake hydrologic class* and *Lake order*. See Additional file 9 for details.

***Stream classification***

Stream reaches are classified in LAGOS_GEO_ following Strahler stream order numbers. See Additional file 10 for details.

***Wetland classification***

Wetlands are classified in LAGOS_GEO_ following three different classification schemes: 1) wetland spatial connectivity to lakes and streams, 2) wetland vegetation composition, and 3) wetland water regime. See Additional file 11 for detailed information on wetland classification.

**LAGOS_GEO_** **freshwater composition and connectivity metrics**

The metrics we developed quantified the abundance of lakes, streams, and wetlands in the landscape and the spatial connectivity among and between feature type(s). Individual metrics were developed following the appropriate approach from those described in Table S26. We quantified each of the metrics of the spatial extents described in Additional file 7. Tables S27-S32 define metric groups, organized by approach, for lakes, streams, and wetlands.

**Patch-based metrics: freshwater composition**

**Table S27A. Lake composition metrics**

| **LAGOS_GEO_ metric name** | **Units** | **Definition** |
| --- | --- | --- |
| Lakes4ha_Overlapping_AREA_pct | % | Percent lake area for all lakes ≥ 4 ha within boundaries defined by each spatial extent with lake area confined to the boundaries of that spatial extent |
| Lakes4ha_AvgSize_ha | ha | Average lake size for lakes ≥ 4 ha in a spatial extent |
| Lakes4ha_Count | count | Count of lakes ≥ 4 ha in a spatial extent |
| Lakes4to10ha_Overlapping_AREA_pct | % | Percent lake area for all lakes > 4 ha and < 10 ha in a spatial extent with lake area confined to the boundaries of that spatial extent |
| Lakes4to10ha_AvgSize_ha | ha | Average lake size for lakes > 4 ha and < 10 ha in a spatial extent |
| Lakes4to10ha_Count | count | Count of lakes > 4 ha and < 10 ha in a spatial extent |
| Lakes10ha_Overlapping_AREA_pct | % | Percent lake area for all lakes ≥ 10 ha in a spatial extent with lake area confined to the boundaries of that spatial extent |
| Lakes10ha_AvgSize_ha | ha | Average lake size for lakes ≥ 10 ha in a spatial extent |
| Lakes10ha_Count | count | Count of lakes ≥ 10 ha in a spatial extent |

**Table S27B. Stream composition metrics**

| **LAGOS_GEO_ metric name** | **Units** | **Definition** |
| --- | --- | --- |
| Streams_SUM_LengthM | m | Sum of stream length in a spatial extent |
| Streams_Density_MperHA | m/ha | Total stream density in a spatial extent |

**Table S27C. Wetland composition metrics**

| **LAGOS_GEO_ metric name** | **Units** | **Definition** |
| --- | --- | --- |
| AllWetlandsUndissolved_Overlapping_AREA_pct | % | Percent wetland area for all wetland types in a spatial extent with wetland area confined to the boundaries of that spatial extent |
| AllWetlandsUndissolved_AvgSize_ha | ha | Average wetland size for all wetland types in a spatial extent |
| AllWetlandsUndissolved_Count | count | Count of all wetland-type patches in a spatial extent |
| ForestedWetlandsUndissolved_Overlapping_AREA_pct | % | Percent forested wetland area in a spatial extent with wetland area confined to the boundaries of that spatial extent |
| ForestedWetlandsUndissolved_AvgSize_ha | ha | Average forested wetland area in a spatial extent |
| ForestedWetlandsUndissolved_Count | count | Count of forested wetland patches in a spatial extent |
| ScrubShrubWetlandsUndissolved_Overlapping_AREA_pct | % | Percent scrub-shrub wetland area in a spatial extent with wetland area confined to the boundaries of that spatial extent |
| ScrubShrubWetlandsUndissolved_AvgSize_ha | ha | Average scrub-shrub wetland area in a spatial extent |
| ScrubShrubWetlandsUndissolved_Count | count | Count of scrub-shrub wetland patches in a spatial extent |
| OpenWaterWetlandsUndissolved_Overlapping_AREA_pct | % | Percent open water wetland area in a spatial extent with wetland area confined to the boundaries of that spatial extent |
| OpenWaterWetlandsUndissolved_AvgSize_ha | ha | Average open water wetland area in a spatial extent |
| OpenWaterWetlandsUndissolved_Count | count | Count of open water wetland patches in a spatial extent |
| Regime<**X>***WetlandsUndissolved_Overlapping_AREA_pct | % | Percent wetland area by water regime in a spatial extent with wetland area confined to the boundaries of that spatial extent |
| Regime<**X**>*WetlandsUndissolved_AvgSize_ha | ha | Average wetland area by water regime in a spatial extent |
| Regime<**X**>*WetlandsUndissolved_Count | count | Count of wetland patches by water regime in a spatial extent |

* **X** = Water regime codes include A, C, F, G, and H

**Graph-based patch metrics: freshwater connectivity**

**Table S28. Lake-stream connectivity metrics**

| **LAGOS_GEO_ Metric Name** | **Units** | **Definition** |
| --- | --- | --- |
| Lakes4ha_Isolated_Overlapping_AREA_pct | % | Percent lake area for isolated lakes ≥ 4 ha in a spatial extent with overlapping lake area clipped to the boundaries of the spatial extent |
| Lakes4ha_Isolated_AvgSize_ha | ha | Average lake size for isolated lakes ≥ 4 ha in a spatial extent |
| Lakes4ha_Isolated_Count | count | Count of isolated lakes ≥ 4 ha in a spatial extent |
| Lakes4ha_Headwater_Overlapping_AREA_pct | % | Percent lake area for headwater lakes ≥ 4 ha in a spatial extent with overlapping lake area clipped to the boundaries of the spatial extent |
| Lakes4ha_Headwater_AvgSize_h | ha | Average lake size for headwater lakes ≥ 4 ha in a spatial extent |
| Lakes4ha_Headwater_Count | count | Count of headwater lakes ≥ 4 ha in a spatial extent |
| Lakes4ha_DRStream_Overlapping_AREA_pct | % | Percent lake area for drainage lakes ≥ 4 ha in a spatial extent with overlapping lake area clipped to the boundaries of the spatial extent |
| Lakes4ha_DRStream_AvgSize_ha | ha | Average lake size for drainage lakes ≥ 4 ha in a spatial extent |
| Lakes4ha_DRStream_Count | count | Count of drainage lakes ≥ 4 ha in a spatial extent |
| Lakes4ha_DRLakeStream_Overlapping_AREA_pct | % | Percent lake area for upstream lake drainage lakes ≥ 4 ha in a spatial extent with overlapping lake area clipped to the boundaries of the spatial extent |
| Lakes4ha_DRLakeStream_AvgSize_ha | ha | Average lake size for upstream lake drainage lakes ≥ 4 ha in a spatial extent |
| Lakes4ha_DRLakeStream_Count | count | Count of upstream lake drainage lakes ≥ 4 ha in a spatial extent |

Note: Each of the lake connectivity metrics was calculated for each of the lake size classes: ≥ 4 ha; 4 ha < lakes < 10 ha; ≥ 10 ha

**Table S29. Lake-lake connectivity metrics**

| **LAGOS_GEO_ Metric Name** | **Units** | **Definition** |
| --- | --- | --- |
| Upstream_Lakes_4ha_Count | count | Number of upstream ≥ 4 ha lakes to a focal lake |
| Upstream_Lakes_4ha_Area_ha | ha | Sum of area of upstream ≥ 4 ha lakes to a focal lake |
| Upstream_Lakes_10ha_Count | count | Number of upstream ≥ 10 ha lakes to a focal lake |
| Upstream_Lakes_10ha_Area_ha | ha | Sum of area of upstream ≥ 10 ha lakes to a focal lake |

**Table S30. Lake-wetland connectivity metrics**

| **LAGOS_GEO_ Metric Name** | **Units** | **Definition** |
| --- | --- | --- |
| AllWetlands_Contributing_AREA_ha | ha | Total area of wetlands that intersect/touch a lake |
| AllWetlands_Count | count | Count of all wetlands that intersect/touch a lake |
| AllWetlands_Shoreline_Km | km | Length of lake shoreline that intersects all wetlands |
| ForestedWetlands_Contributing_AREA_ha | ha | Total area of forested wetlands that intersect/touch a lake |
| ForestedWetlands_Count | count | Count of forested wetlands that intersect/touch a lake |
| ForestedWetlands_Shoreline_Km | km | Length of lake shoreline that intersects forested wetlands |
| ScrubShrubWetlands_Contributing_AREA_ha | ha | Total area of scrub-shrub wetlands that intersect/touch a lake |
| ScrubShrubWetlands_Count | count | Count of scrub-shrub wetlands that intersect/touch a lake |
| ScrubShrubWetlands_Shoreline_Km | km | Length of lake shoreline that intersects scrub-shrub wetlands |
| OpenWaterWetlands_Contributing_AREA_ha | ha | Total area of open water wetlands that intersect/touch a lake |
| OpenWaterWetlands_Count | count | Count of open water wetlands that intersect/touch a lake |
| OpenWaterWetlands_Shoreline_Km | km | Length of lake shoreline that intersects open water wetlands |

**Table S31. Wetland-stream connectivity metrics**

| **LAGOS_GEO_ Metric Name** | **Units** | **Definition** |
| --- | --- | --- |
| IsolatedWetlandsUndissolved_Overlapping_AREA_pct | % | Percent wetland area for isolated wetlands in a spatial extent with overlapping wetland area clipped to the boundaries of the spatial extent |
| IsolatedWetlandsUndissolved_AvgSize_ha | ha | Average wetland size for isolated wetlands in a spatial extent |
| IsolatedWetlandsUndissolved_Count | count | Count of isolated wetlands in a spatial extent |
| SingleWetlandsUndissolved_Overlapping_AREA_pct | % | Percent wetland area for single wetlands in a spatial extent with overlapping wetland area clipped to the boundaries of the spatial extent |
| SingleWetlandsUndissolved_AvgSize_ha | ha | Average wetland size for single wetlands in a spatial extent |
| SingleWetlandsUndissolved_Count | count | Count of single wetlands in a spatial extent |
| ConnectedWetlandsUndissolved_Overlapping_AREA_pct | % | Percent wetland area for connected wetlands in a spatial extent with overlapping wetland area clipped to the boundaries of the spatial extent |
| ConnectedWetlandsUndissolved_AvgSize_ha | ha | Average wetland size for connected wetlands in a spatial extent |
| ConnectedWetlandsUndissolved_Count | count | Count of connected wetlands in a spatial extent |

**Table S32. Stream connectivity metrics**

| **LAGOS_GEO_ Metric Name** | **Units** | **Definition** |
| --- | --- | --- |
| Headwaters_SUM_LengthM | m | Sum headwater stream length |
| Headwaters_Density_MperHA | m/ha | Headwater stream density in a spatial extent |
| Midreaches_SUM_LengthM | m | Sum midreach stream length |
| Midreaches_Density_MperHA | m/ha | Midreach stream density in a spatial extent |
| Rivers_SUM_LengthM | m | Sum river stream length |
| Rivers_Density_MperHA | m/ha | River stream density in a spatial extent |

### References

1. Gustafson, EJ: **Quantifying landscape spatial pattern: what is the state of the art?** *Ecosystems* 1998, **1**: 143-156.

2. Eros T, Olden JD, Schick RS, Schmera D, and Fortin MJ: **Characterizing connectivity relationships in freshwaters using patch-based graphs***. Landscape Ecology* 2012, **27**: 303 – 317.
