# Supplementary material for: Building a multi-scaled geospatial temporal ecology database from disparate data sources: fostering open science and data reuse
Source: Gigascience. 2015 Jul 1;4:28. doi: 10.1186/s13742-015-0067-4 (PMC4488039; doi:10.1186/s13742-015-0067-4)
Supplement: Additional file 13: — Database export formats for LAGOSGEO. This file contains a description of the strategies for exporting the heterogeneous and large volume of data from LAGOSGEO. [file 13742_2015_67_MOESM13_ESM.docx]

Additional file 13

**Database export formats for LAGOS_GEO_**

Ed Bissell, Patricia Soranno

**Overview**

The export strategy for the LAGOS_GEO_ database was somewhat simpler than that for the LAGOS_LIMNO_ database because LAGOS_GEO_ has a horizontal format whereas LAGOS_LIMNO_ has a vertical format but needed horizontally formatted exports. However, certain features of LAGOS_GEO_ presented challenges when developing its export strategy. Most notably, LAGOS_GEO_ contained a great many variables (>500), collectively representing a variety of data sources and a variety of spatial extents at which particular variables could be calculated. Therefore, we grouped sub-sets of geographic variables into five themes, based on the nature of their content, their data sources, and the specific spatial extents at which variables could be calculated. We generated an export table for each unique combination of geographic theme and spatial extent. The spatial extent of calculation is represented by the variable *ZoneID*, which is also included for each lake in LAGOS_LIMNO_, allowing for easy linkage between any geographic and lake nutrient export tables.

**Defining the data to be exported in LAGOS_GEO_**

Most statistical software programs or scripting packages are optimized for data tables that are in horizontal format (i.e., data values for each variable are stored in separate columns). For LAGOS_GEO_, all of the data tables are stored horizontally, which makes the mechanics of exporting the data easier than those for the vertically formatted LAGOS_LIMNO_ database. However, despite the relative ease of export mechanics for the LAGOS_GEO_ database, the sheer number of geographic variables poses its own challenges. There are over 500 GEO variables, measured at a range of spatial extents (therefore resulting in far more than 500 columns). Although it might be technically possible to have a data table with over 500 columns, doing so introduces analytical and data management challenges related to working with such a wide dataset in many statistical packages. Additionally, it is unlikely that, for any one analysis, a user would need or want all 500 variables. Therefore, we developed a data export strategy for the LAGOS_GEO_ database that aimed to reduce the large volume and heterogeneity of the GEO variables into a variety of export tables that were designed to be appropriate for a range of anticipated analyses.

In some ways, the geographic data to export from LAGOS were simpler than the limnological data because: (a) in LAGOS_GEO_ there were no issues related to sample depth,; (b) for any individual GEO variable, the data for all lakes originated from the same dataset (i.e., land use/cover for all lakes come from the same national dataset); (c) there were relatively few missing values; and, (d) the data were already stored horizontally in LAGOS. However, each GEO variable (for the most part) originated from a different dataset. Such multi-thematic data can be challenging to integrate. Additionally, the sheer number of geographic variables required us to think through and anticipate the specific subsets of variables (i.e., export tables) that would be most conducive to subsequent analyses. Our approach was to create and import GEO variables in separate tables (for the most part), and then to integrate those separate tables into five main themes (see below). Many of the GEO variables were calculated at a wide range of spatial extents (see below). Therefore, we created export tables for each specific combination of theme and spatial extent.

Defining spatial extents for GEO data measurement: We identified nine possible spatial extents for measuring at least some of the GEO data (see Additional file 7 and columns in Table S33 below). Not all of the GEO variables could be quantified at all of the spatial extents. The spatial extents were chosen based on their documented or hypothesized ecological importance to lake nutrients. As a result, many of the extents are hydrologically based (e.g., individual lake watersheds or large river watersheds in which multiple lakes occur) to delineate regions that might be ecologically important for understanding lake variation across a range of spatial extents.

Defining GEO themes to export data into separate tables: We combined data tables that were from similar themes (i.e., climate data) or had similar data types (e.g., data that are text, numeric, proportional, etc.) and for which specific metrics could be meaningfully calculated at the same spatial extents (Table S33). This process resulted in the following five themes: 1) climate, hydrology, atmospheric and geology data could be measured at six spatial extents and are collectively referred to as CHAG; 2) variables related to land surface features, such as land use/land cover, slope, and density of roads and dams are measured at nine spatial extents and are referred to as LULC; 3) variables that represent surface water connections past (glaciation) and present are referred to as CONN; 4) temperature and precipitation data analyzed at an annual time scale across six spatial extents are referred to as CLIM_ANN; and, 5) temperature and precipitation data analyzed at a monthly time scale are referred to as CLIM-MON. This theme also contains spatially invariant climate metrics that are calculated at monthly time scale. Additionally, some geographic variables were measured at the individual lake scale; especially those that characterize a lake’s unique position in a surface water flowpath (see the bottom section of Table S33). A table containing the lake-specific geographic variables was exported after LAGOS_GEO_ and LAGOS_LIMNO_ data were combined.

Linking LAGOS_GEO_ and LAGOS_LIMNO_: LAGOS_GEO_ and LAGOS_LIMNO_ data are linked through the variable *ZoneID*. For LAGOS_GEO_, each export table contains the value for *ZoneID* indicating the spatial extent at which the GEO variables contained in that export table were calculated. For LAGOS_LIMNO_, each of the spatial extent *ZoneIDs* is provided for each lake in the dataset. Therefore, the lake and geographic data can be easily combined, for any desired spatial extent, through the *ZoneID.*

**Specific steps to export geographic data (LAGOS_GEO_)**

As stated above, our export strategy for the GEO data was to create tables specific to each combination of theme and spatial extent. This resulted in our exporting a relatively large number of tables that users could then integrate with the LAGOS_LIMNO_ database as needed by lake ID or by spatial extent ID (*ZoneID)*. The steps are as follows:

1. Using PERL scripts, load the text files that were created from the CSI-LAGOS geoprocessing toolbox:
   1. The PERL script generates the necessary SQL statements to load the 1,300+ GEO tables with minimal user intervention.
   2. Standardize and uniquely name the primary and foreign keys based on table names.
2. Combine tables for variables contained within each of the five listed thematic categories into separate exports for each relevant spatial extent (summarized in Table S33). The five thematic categories and the variable types that each one contains are as follows:
   1. CHAG - 30-year normal **C**limate (TMIN, TMAX, TMEAN, PPT), coarse **H**ydrology measures (runoff, baseflow, recharge, saturated overland flow), **A**tmospheric deposition (TN, NO3, SO4 for 6 years), and surficial **G**eology.
   2. LULC - land use/land cover for 4 years, impervious cover for three years, canopy cover for 1 year, slope and TRI, road density, dam density.
   3. CONN - stream density (total stream and by stream order), lake connectivity metrics, wetland connectivity metrics, and glaciations.
   4. CLIM_ANN - annual climate data for each year for TMIN, TMAX, TMEAN, PPT for 42 years also with year as a column.
   5. CLIM_MON - monthly climate data for TMIN, TMAX, TMEAN, PPT for 42 years, and spatially invariant monthly climate metrics such as ENSO and NAO.

**Table S33. A summary table of data exports from the LAGOS_GEO_ database**

Each cell with an ‘x’ represents a combination of geographic variables and spatial extent of their calculation for which one or more export tables were created. See Additional File 7 for explanation of each spatial extent. Spatial extents are represented by the variable ‘*ZoneID*’ in the database. Geographic variables come from several sources, represent a wide variety of landscape and climate properties, and vary in terms of the spatial extent at which they can be calculated. Therefore, geographic variables were subdivided into five theme areas (GEO theme codes CHAG, LULC, CONN, CLIM_ANN and CLIM_MON). Geographic variables that are lake specific (GEO them code = LAKE) were also calculated and exported, after linking LAGOS_GEO_ and LAGOS_LIMNO_ databases.
