# Supplementary material for: Building a multi-scaled geospatial temporal ecology database from disparate data sources: fostering open science and data reuse
Source: Gigascience. 2015 Jul 1;4:28. doi: 10.1186/s13742-015-0067-4 (PMC4488039; doi:10.1186/s13742-015-0067-4)
Supplement: Additional file 14: — QAQC protocol for LAGOSGEO. The protocols that we used to QAQC the data after all of it had been loaded into and then exported from LAGOS. [file 13742_2015_67_MOESM14_ESM.docx]

Additional file 14

**QAQC protocol for LAGOS_GEO_**

Caren Scott, Sarah Collins, C. Emi Fergus, Nick Skaff, Kendra Spence Cheruvelil, Nicole Smith, Patricia Soranno

**Overview**

The spatial lake and landscape data that populate LAGOS_GEO_ come from multiple data sources, including field studies, remotely sensed imagery, and geographic information systems (GIS) data layers. The original data layers capture multiple data categories that include lake geomorphic attributes, topography, terrestrial land cover/land use, freshwater landscape features (i.e. lakes, streams, and wetlands), hydrology, atmospheric deposition and climate. The spatial data in LAGOS_GEO_ were created by developing project-specific GIS tools in the ArcGIS environment, which are referred to as the LAGOS GIS Toolbox. The toolbox outputs multiple individual data tables of calculated values organized by spatial themes (see below for definition) that are then imported into LAGOS_GEO_ for different spatial extents, including values calculated at the level of the individual lake, the watershed, and several measures of region.

The overall goal of the LAGOS_GEO_ quality assurance/quality control (QAQC) process is to identify possible errors in the extensive GIS data processing that creates the LAGOS_GEO_ spatial metrics data and to correct these problems. We expect that the original data layers have gone through extensive QAQCing and thus any errors detected would likely reflect problems with LAGOS GIS data processing. We define errors and egregious values to be: 1) values that do not make ecological sense; 2) values that are well beyond what has been observed in previous studies; 3) values that are not technically feasible; or, 4) null values that indicate an absence of data, when in fact data exist. Note that it is not our intention to remove statistical outliers that may or may not be real/true values.

**Phase I: Summary of Procedure for First Round of LAGOS_GEO_ QAQC**

Phase I of LAGOS_GEO_ QAQC examines the first GIS data table exports (individual, non-integrated tables) from the LAGOS GIS Toolbox. In this phase, we seek to identify problems or errors in the original data or errors resulting from the GIS Toolbox processing. When problems are identified with the GIS-exported data tables, they are brought to the attention of the GIS analyst and fixed. In particular we developed a strategy to detect the following potential errors in the spatial metric data: 1) errors in the original source data (e.g., measurement error, missing data that are misclassified); 2) data that were imported into our database incorrectly (e.g., unit conversion errors, importing errors such as null values that are supposed to be a value of zero, and vice-versa); and, 3) other errors in the output tables originating from the LAGOS tools themselves. Some of the approaches that we used to evaluate the spatial metric data are entirely objective and quantitative (e.g., technically impossible values), while other approaches rely on expert opinion (e.g., distribution of a variable) or established knowledge (e.g., the relationship between land cover types). An overview of steps in Phase I of QAQC is outlined below. Steps 3-9 are described in detail later in the document.

1. **Determine variables to retain for LAGOS_GEO_ from the GIS-exported data tables**. Eliminate extraneous or repetitive variables. Because the LAGOS GIS Tools automatically create multiple spatial metric data columns, we first eliminated repetitive or ecologically meaningless variables and did not import them into LAGOS. For example, range is a metric that is automatically created but, because range can be calculated based on minimum and maximum values, we eliminated it and minimum and maximum columns were retained.
2. **Clearly identify the variable column headers and units**. Verify variable column heading names and units of measurement.
3. **Map the GIS-exported data and evaluate study extent boundaries**. Verify that the tabular GIS-exported data tables pertain to spatial features within the LAGOS study extent boundaries by creating maps of the data points.
4. **Quantify the number of polygons (called zones, hereafter) that cover the data:** Count the number of zones for each spatial extent. We use these values to verify that the number of zones are equal to the number of rows in the data table at the respective spatial extent (e.g., HU4 = 65 and all HU4 data exports should have 65 rows).
5. **Identify values that are missing (NA), impossible, or zero:** Calculate summary statistics for each spatial metric to be sure there are no NAs or negative values, and to confirm that the minimum value is 0 for each metric (where appropriate). Create maps of NA observations to investigate possible problems with original data source or GIS tool outputs.
6. **Plot data distributions**: Check the frequency distributions to look for unusual patterns, such as bimodal distributions where normality is expected.
7. **Perform a proportion/percentage check**: Confirm that metrics expressed as a proportion or percentage sum to 1 or 100%, respectively.
8. **Perform a spatial check**: Make maps of selected metrics to visualize broad spatial patterns (e.g., land use/land cover, geology, and climate) and examine spatial extent boundaries.
9. **Perform a metric assumption** **check**: Create bi-plots of selected metrics to look for nonsensical relationships and check for expected relationships.

**Phase II: A summary of the procedure for the second round of LAGOS_GEO_ QAQC**

After problems were identified in the first round of QAQC, LAGOS GIS tools were revised and new data exports were produced. The database administrator compiled the GIS-export tables into integrated LAGOS spatial metric tables that are organized by spatial theme (e.g., CHAG) and spatial extent (e.g., HU12). We performed a second phase of LAGOS_GEO_ QAQC on these integrated tables. This phase focused on identifying possible problems with data import and export to the LAGOS_GEO_ database and it rechecked for possible errors in the LAGOS GIS tools outputs. We used many of the same strategies as the first QAQC effort to achieve these goals. Specifically, we repeated steps 3-9 from Phase I QAQC; we provide specific examples of these steps below.

**Detailed description of steps 3-9 used in QAQC procedures:**

**Step 3. Map GIS-exported data and evaluate study extent boundaries**

Mapping the tabular spatial data provides quick and useful visualizations of the data that we can QAQC for coarse spatial accuracy. In particular we can verify that the data points are within the study extent boundaries (Figure S16).

**Step 4. Quantify the numbers of zones for each spatial extent**

The LAGOS study area was divided into different spatial extents (Additional file 7). For each spatial extent, we calculated the total number of polygons or zones (Table 1) to make sure that each data table export had the correct number of rows. Even if a value from one of the tools is null, each zone should have its own row in all tables and unexpected values would indicate a problem.

1. **Make zone table:** We calculated the total number of zones for each spatial extent (Table 1)
2. **Make sure the number of zones is correct in subsequent tables:** For each table at each extent, we checked to make sure that the number of rows matched the correct number of zones in Table S34.

**Step 5. Missing (NA), impossible, or zero values**

1. **Negative values:** As negative values would be nonsensical across most of the metrics, we confirmed that none were present. For the few metrics where a negative value would be meaningful, we accepted negative values.
2. **NA and zero values:** If NA values existed, then we created a map to examine where they occurred (Figure S17). For example, if NA values existed on a coast or border or other region where it was reasonable for them to occur, then we considered them to be valid. However, if NA values occurred in a region where there was no obvious reason for missing data, then it suggested a potential problem with the LAGOS GIS tools or gaps in the original GIS data. We also mapped zero values to evaluate the possibility of NA values being incorrectly transformed to zeros during input to the database.

**Step 6. Data distributions**

1. **Typical distribution**: Most of the spatial metrics have multiple zero values and are right skewed, even when log transformed (typical distributions are shown in Figure S18).
2. **Atypical distributions**: We evaluated distributions that were atypical (e.g*.*, bimodal distribution or with outliers falling far beyond the curve) based on expert opinion and scientific knowledge, and sought secondary evidence to indicate whether problems existed with these data.

**Step 7. Proportion/percent check**

For variables that are expressed as a proportion or a percent (e.g., land use/land cover categories), it is expected that no single category will exceed 1 (for proportion data) or 100% (for percent data), and that the sum of all of the categories will be roughly equal to 1 (for proportion data) or 100% (for percent data). We checked to confirm that all of the proportion/percent data met these criteria. Moreover, the sum of all the areas for land use/land cover should equal the total area of the watershed. Note: due to rounding and approximations, these do not have to be exact but they should be relatively very close.

**Step 8. Spatial check**

When possible, we created maps of selected metrics to visualize metric quartile values (e.g., land use/land cover, climate) and to see if they matched expected patterns. For example, there are known gradients in the study extent for variables such as precipitation, temperature, and N deposition. In addition, we mapped NA observations to determine where there was missing data. Conducting a spatial check allowed us to determine whether patterns generally matched the patterns from original source data, and to confirm that GIS tools and the import-export process did not distort the data or result in NA values.

**Step 9. Check of metric assumptions**

We evaluated metric assumptions visually using bi-plots to check for nonsensical metric values and to check for expected relationships among variables. Outliers in the spatial metric values show up as points that fall far away from the regression between the two variables or in additional group(s) within the data. Nonsensical metric values indicated a possible problem with the implementation of the GIS tool and were addressed by the GIS analyst. Metric values that did not follow expected trends were checked to confirm that outliers were reasonable based on expert knowledge and secondary data. Some examples of bi-plots used in the LAGOS_GEO_ QAQC process are given below.

1. **Bi-plots of count versus area**: For spatial metrics that quantify the same spatial feature (e.g., total lake area and total lake count), there should never be a zero for one metric without a corresponding zero for the other metric. For example, zones with zero lake area should have a zero lake count (Figure S19).
2. **Bi-plots of lake size classes versus all lakes**: Lakes in LAGOS are grouped by size: lakes with surface area ≥ 4 ha and less than 10ha and lakes with surface area ≥ 10 ha. We expect that lakes within these size classes should never have metric values that are greater than the total lake metrics. For example, there should be a positive relationship between lakes ≥ 10 ha and the total lake dataset, and there should never be more observations in 10ha lakes size class than the total lakes in the full dataset. We confirmed that the total number of lakes was greater than the number of lakes in the 10ha lakes size class by visually checking that all of the points were above the 1:1 line in a regression (Figure S20). Deviation from this expected pattern would indicate a problem with the LAGOS GIS tools.
3. **Bi-plots of freshwater classes versus all lakes**: Lakes in LAGOS are grouped by hydrologic class (Additional file 12. Similar to the lake size class, we expect that lakes within hydrologic classes should never have metric values greater than the total lake metrics. This was evaluated visually by plotting individual lake hydrologic class metrics versus total lake metrics and making sure that all of the points were above the 1:1 line (Figure S21). Deviation from this expected pattern would indicate a problem with the LAGOS GIS tools.
4. **Bi-plots of overlapping versus contributing lake area**: Lake polygons do not always nest completely within the spatial extents such that some lake polygons overlap multiple zones. LAGOS_GEO_ includes two metrics of lake area: 1) an overlapping lake area, in which lake polygons were clipped at the outside of a zone so the partial area of a lake is included when it spans multiple zones; and, 2) a contributing lake area, which includes the total area of all lakes that exist in a zone, even if some of the area is actually in another zone. We checked to confirm that contributing lake area was always greater than or equal to the overlapping lake area.
5. **Bi-plots of two types of land cover**: We expect certain coarse trends among land use/land cover classes. We created bi-plots of land use/land cover percent for certain classes to visually assess whether these assumptions were being met. For example, the percentages of deciduous forest and agricultural land use are expected to be negatively related (Figure S22).


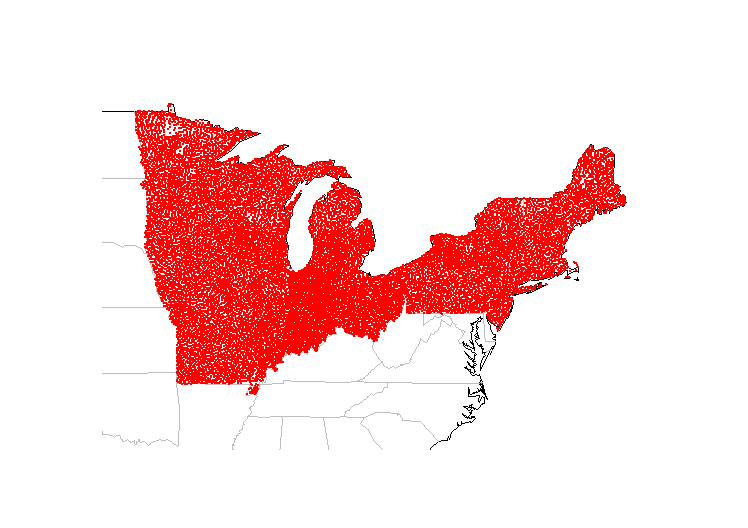


**Figure S16. Example map of LAGOS_GEO_ data points.** This particular figure maps of HU12 tabular data so that we can verify that the data are contained within the boundaries of the State study extent.


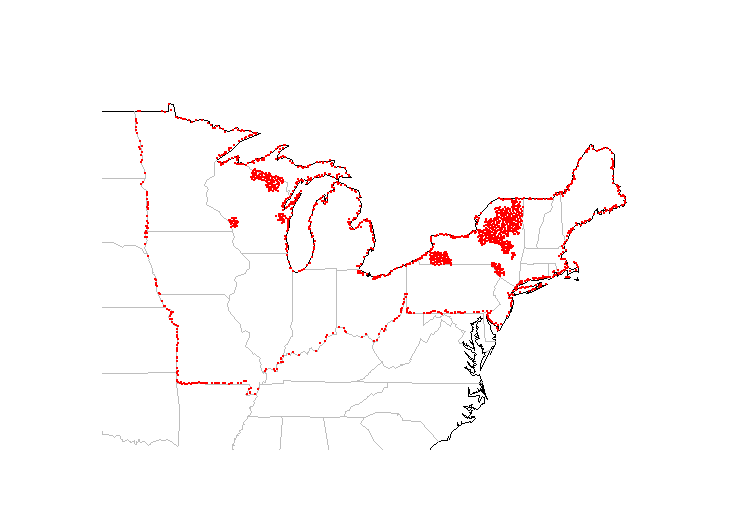


**Figure S17**. **Example map of NA observations.** This particular figure maps HU12 with missing wetland data (from an early version of LAGOS_GEO_). It is common to have missing data along the study extent boundaries. Patches of missing wetland data in Wisconsin and New York were populated when updated wetland source data was made available.


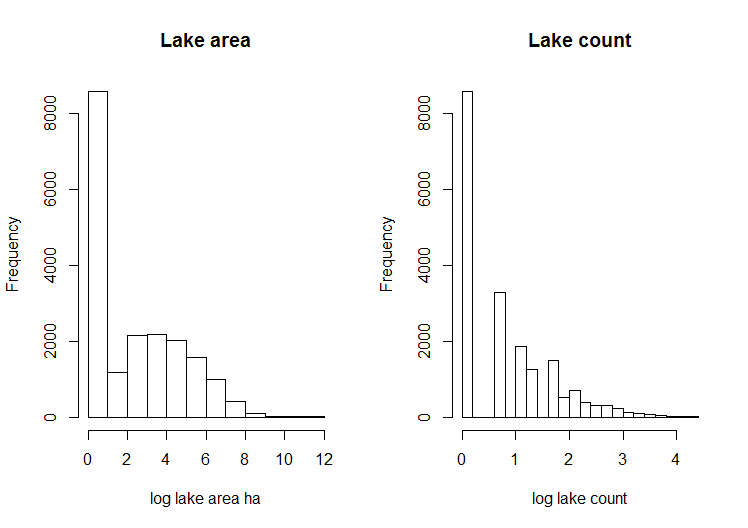


**Figure S18. Example of typical distributions for lake area and lake count metrics at the HU12 spatial extent.** Note the high frequency of zeros and the right skew in the data.


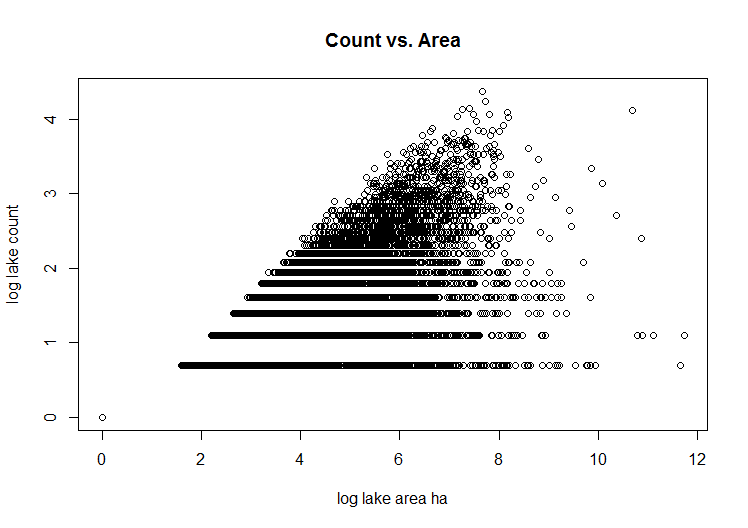


**Figure S1**9. Example of bi-plot of lake count versus lake area. Any points that fall in the red boxes would indicate a problem (i.e., a zero value for one metric but not the other).


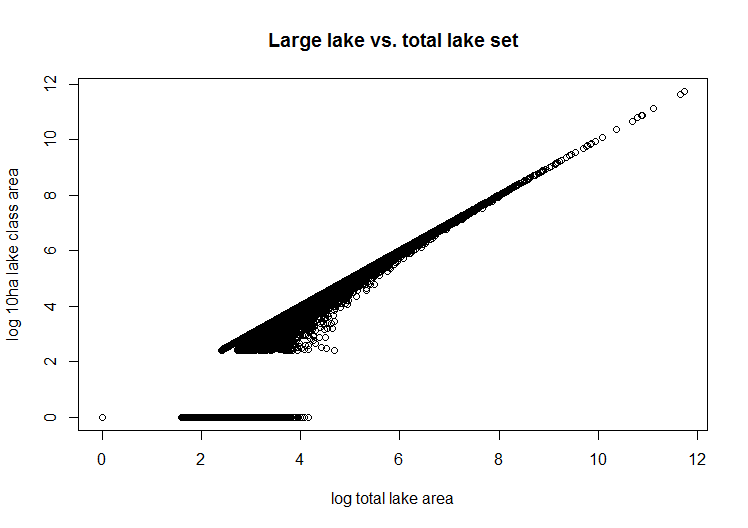


**Figure S20. Example bi-plot of lake size class (lakes ≥10 ha) area versus total lake area.** The total number of lakes includes all lakes ≥ 10 ha and thus all points should fall below the 1:1 line.


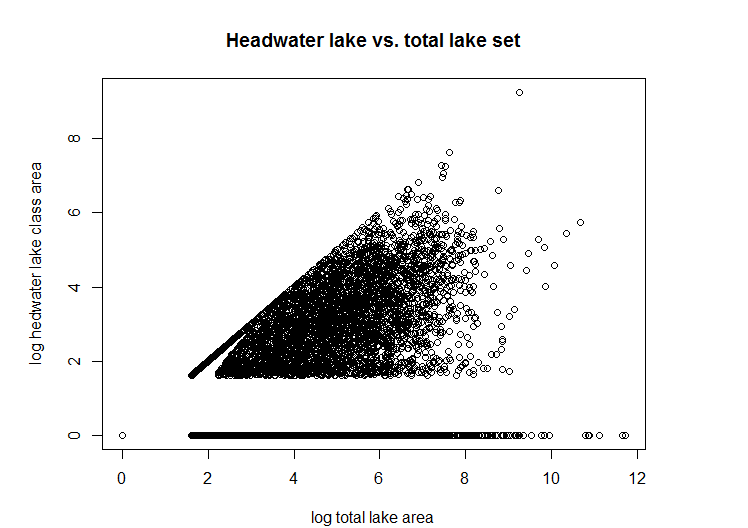


**Figure S21. Example bi-plot of lake hydrologic class area versus total lake area.** The total number of lakes includes all headwater designated lakes and thus all points should fall below the 1:1 line.


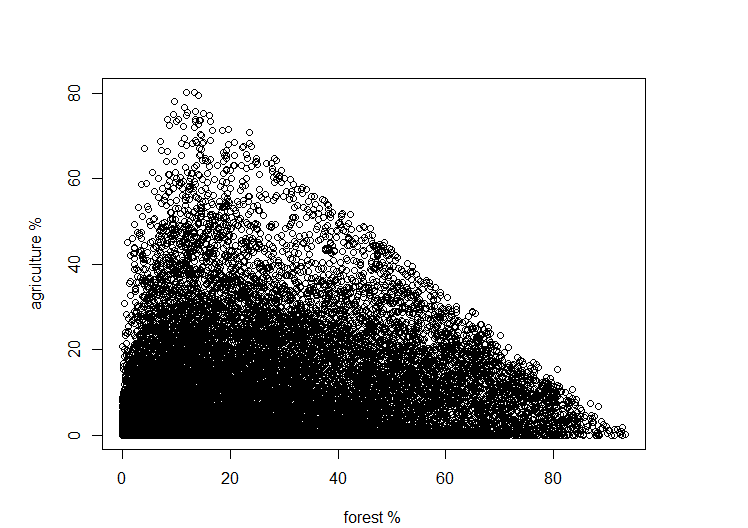


**Figure S22**. **Example bi-plot of agricultural land use percentage versus forested land cover percentage.** We expect a negative reciprocal trend between percent agriculture and forest within a spatial extent.

**Table S3**4. Number of zones per spatial extent

| **Spatial extent** | **Number of zones** |
| --- | --- |
| IWS | 51,054 |
| State | 17 |
| County | 955 |
| EDU | 91 |
| HU4 | 65 |
| HU8 | 511 |
| HU12 | 20,257 |
