# Supplementary material for: Building a multi-scaled geospatial temporal ecology database from disparate data sources: fostering open science and data reuse
Source: Gigascience. 2015 Jul 1;4:28. doi: 10.1186/s13742-015-0067-4 (PMC4488039; doi:10.1186/s13742-015-0067-4)
Supplement: Additional file 15: — Georeferencing the lake sampling locations to lake polygons using GIS. A description of the challenges and solutions to georeference each lake in our study area and connect that location to lake sample events. [file 13742_2015_67_MOESM15_ESM.docx]

Additional file 15

**Georeferencing the lake sampling locations to lake polygons using GIS**

Ed Bissell, Patricia Soranno

**Overview**

Identifying the spatial location and uniquely identifying each lake in LAGOS required us to georeference the lake sampling locations to lake polygons in a GIS data layer. We used the National Hydrography Dataset (NHD) Waterbody Feature Class as a basemap, which allowed us to spatially join lake sampling locations to NHD lake features. This document describes the specific steps to do this. Although the process can be somewhat automated in the GIS environment, it required substantial manual processing to assure that data were correctly referenced to lakes in the NHD. Specific issues that required manual checking included sampling datasets with problematic latitude/longitude information, and occasional incorrect assignments of a lake to nearby wetlands or other water bodies in the NHD.

**Selection of a basemap for lake polygons for LAGOS: The National Hydrography Dataset**

The National Hydrography Dataset (NHD) is a geographic representation of surface water within the United States developed by the USGS, other federal agencies, and local partner agencies (see Additional files 9-10). It is available at a relatively high resolution (1:24,000) for the conterminous US. The NHD includes features such as rivers, streams, canals, lakes, and ponds that represent surface water bodies and flow networks. Thus, the NHD, specifically the NHD Waterbody Feature Class, was selected to serve as the basemap used to locate (i.e., spatially join) the sample locations for the lake sampling datasets in a uniform fashion across the 17 states in our study area.

**Joining sample locations to the NHD Waterbody Feature Class**

A critical feature of integrated geospatial-temporal databases is to spatially join the data from the sampling programs that provided the lake chemistry data on a given lake to the geospatial data for that lake that is obtained from a different source. When there are ~50,000 lakes in our study area, and ~100 datasets for lake chemistry, this joining is challenging because 1) each of the 100 datasets have different unique identifiers to identify lakes, 2) few if any of the 100 datasets provided their lake sampling data in a GIS database, 3) there are many lakes with the same name, and so lake name cannot be used as an identifier, and 4) the sampling programs rarely use the NHD dataset to locate their lakes. To address these challenges, we determined that using a lake’s latitude/longitude (which was almost always provided by the lake sampling program data providers) was the best way to minimize errors that link a lake’s sampling data to its location in the NHD dataset. Nevertheless, this strategy also had potential problems because a lake’s latitude/longitude values also sometimes had problems, since these values signified either the sampling location, the center point of the lake, the location of the lake access point, some point on the lake shoreline, or sometimes a location on land near the lake. For almost all of our sampling programs, we were able to acquire the latitude/longitude of a lake, but some had one of the above problems associated with them. Therefore, although there is a way to automate this procedure within the GIS environment, it required manual checking and manipulation for many lakes, and so was fairly labor intensive. We, thus, consider this approach as 'semi-automated.'

Major steps in the semi-automated approach to georeferencing the lake sample locations from an individual lake sampling dataset to the NHD in GIS:

Before georeferencing any datasets:

1. All analyses are performed using ArcGIS for Desktop (ESRI, Version 10.1).

A feature class of NHD waterbody features for the entire 17-state study area was created by appending together each NHD waterbody feature class in the subregion pre-staged subregions geodatabases (downloaded from the US Geological Survey [1]). This resulting feature class was also projected to a common coordinate system (Albers USGS version) and duplicate waterbodies from adjacent subregions were removed.

For each lake sampling dataset:

1. In ArcMap, create a *lake sampling dataset point feature class* that contains all lakes in the lake sampling dataset using the lakes’ latitude/longitude by converting the latitude/longitude values to a GIS point coverage representing the lake or sampling locations.
2. Perform a spatial join between the *lake sampling dataset point feature class* and the *NHD waterbody features* in ArcMap. A spatial join assigns attributes from 1 spatially coincident feature to another, in this case from the *NHD waterbody features* to the *lake sampling dataset point feature class*.
3. Transfer the NHD field 'Permanent_Identifier,' which uniquely identifies each NHD waterbody to the *lake sampling dataset point feature class*, thus establishing the link from the NHD to the sample locations in the lake nutrient dataset. This value will become the unique identifier that LAGOS uses to identify lakes.
4. Determine where a lake in a sampling program did not overlap a lake polygon, and so was determined to be 'un-linked.' Investigate all un-linked lakes manually:
5. In cases where a significant percentage of lakes were not matched, a small tolerance (not more than 10 m) was applied to improve results.
6. Using additional information, such as aerial photographs or topographic maps, we attempted to find the best possible match for any un-linked lakes.
7. In some cases, the sample location fell just outside of the *NHD waterbody features* polygon and both shared a common lake name. In these cases the sample location was attributed with the corresponding *NHD* *waterbody features* polygon’s 'Permanent_Identifier' and was flagged in the database flag that we created for this purpose (Table S35) and given the value of 'Linked by common name and location.'
8. All other cases of un-linked lakes, in which additional information allowed us to link a lake sampling event to a lake location, were also flagged, and the 'Permanent_Identifier' was only transferred if it was obviously for the correct lake.
9. In some rare cases, un-linked lakes were manually assigned a 'Permanent_Identifier' by the database administrator based on a preponderance of evidence (other GIS datasets, other LAGOS_LIMNO_ datasets for the same area, fishing websites, Google Maps, ESRI basemaps, etc.) Note also that the population of *NHD waterbody features* used in the georeferencing process included ALL lakes and wetlands (i.e., lakes were not filtered out by FCODE as was done later for the LIMNO_GEO_ processing). Ultimately, some lakes that we were able to join to a waterbody polygon in the NHD were later dropped from LAGOS based on how we defined lakes for this integrated database. For example, there were 56 lakes that were joined to wetland polygons rather than a lake polygon. However, when there were sampling events that did fall within a wetland that was adjacent to a nearby lake, these sampling events were joined to the nearby lake.

**Table S35. The controlled vocabulary and the descriptions of the flag that we created for the georeferencing process called POSFLAG**

| **POSFLAG controlled vocabulary value** | **Description** |
| --- | --- |
| NULL | The sample lake’s latitude/longitude fell within a lake polygon automatically. |
| Linked by common location | The sample lake was linked because the *NHD waterbody feature* was the only lake in the area of the sample lake, even though the latitude/longitude of the sample lake did not fall within the lake polygon. |
| Linked by common name | The sample lake was linked because it and the *NHD waterbody feature* shared the same name and the name appeared unique enough to assume it was the correct lake. |
| Linked by common name and county | The sample lake was linked because it and the *NHD waterbody feature* shared the same name and the *NHD waterbody feature* was in the same county based on a county column supplied by the sample dataset. |
| Linked by common name and location | The sample lake was linked because it and the *NHD waterbody feature* were close to each other and shared the same name. |
| Point location off | The latitude/longitude coordinates of the sample lake fell outside of the *NHD waterbody feature.* The lake was initially un-linked, but later manually linked due to the use of various sources confirming location that were not based on any of the above lines of evidence. |
| Point location off NHD and limno names conflict | The latitude/longitude coordinates of the sample lake fell outside of the  *NHD waterbody feature* and the sample lake and *NHD waterbody feature* names conflicted. The lake was initially un-linked, but later manually linked due to the use of various sources confirming the name and location of the sample lake. |

**References**

1. U.S. Geological Survey: **Complete FCode list for NHD hydrography features**. http://nhd.usgs.gov/. Accessed 4 June 2015.
