# Supplementary material for: Building a multi-scaled geospatial temporal ecology database from disparate data sources: fostering open science and data reuse
Source: Gigascience. 2015 Jul 1;4:28. doi: 10.1186/s13742-015-0067-4 (PMC4488039; doi:10.1186/s13742-015-0067-4)
Supplement: Additional file 16: — Strategy for discovering and acquiring lake water quality datasets. The approach that we used for discovering, requesting and acquiring lake water quality datasets from a variety of data providers, focusing on datasets already in the public domain. [file 13742_2015_67_MOESM16_ESM.docx]

Additional file 16

**Strategy for discovering and acquiring lake water quality datasets**

Patricia Soranno, Emily Stanley

**Overview**

We developed a strategy to discover, request, and acquire lake water quality datasets from a range of sources, focusing on datasets in the public domain such as from state agency datasets or citizen monitoring programs. We developed protocols, form letters, and documentation to describe the data sources and metadata and to ensure reproducibility of our final database that are described in later supplemental files. Here, we describe our strategy for discovering and acquiring lake water quality datasets.

**Measuring lake water quality in the United States**

In the US, states are legally required to monitor water quality of the water bodies of the state (see Appendix for the history of the US Clean Water Act). In addition, the methods for measuring water quality have been fairly well standardized through such publications as ***Standard Methods for the Examination of Water and Wastewater*** (published since 1905, with 20 editions thereafter), with many of its methods approved by the EPA in later editions. Consequently, we expected that many states would use similar methods for determining measures of lake water quality (total nutrient concentrations, chlorophyll concentration, Secchi depth, etc.) and thus would provide a rich source of data across many states that could be compiled into an integrated sub-continental lake water quality database. This database would be a potent resource for answering research questions at these broad geographic scales.

In addition, given the establishment of such methods at the state and federal levels, it is likely that individual researchers would also use similar methods, and so we also sought datasets from a wider range of sources, including individual researchers, other federal or state programs, tribal programs and citizen monitoring programs.

Therefore, our strategy was to collect water quality data from as many sources as possible, focusing on programs that provided samples from many lakes across broad geographic areas (priority) or samples from fewer lakes, but across long time periods (also a priority). We assume that water quality likely would be measured similarly in other countries, but because all of our GIS data sources provided data that were within US national borders, we restricted our study extent to US lakes.

**Strategy for discovering and acquiring water quality datasets**

We sought datasets in 17 NE and Midwest US states that make up much of the glaciated, lake-rich area of the US. We had 13 limnologists on our project (students, post-doctoral researchers, faculty, and staff scientists) who each searched for datasets within different states. We used our professional networks to identify existing sampling programs or datasets. Once a dataset or program was identified, we identified the appropriate contact person (typically identified on a web page) and sent him/her a data request using a standard memo (Additional file 18). Along with the request for data, we asked data providers for any metadata or documentation that described field and laboratory methods, detection limits, quality assurance practices, data processing, etc. Because we expected each data provider to document their data in different ways, we created our own metadata form for each dataset using the EML standard metadata format for ecological datasets (see Additional file 3).

Another key component of a lake dataset is the information about each lake, including the coordinates of the lake itself, the lake name, the unique identifier of the lake if relevant, and the depth of the lake and/or the sample depth. Such data are often challenging to acquire and for some lakes, we had to search for additional sources of information for the depth of the lake in particular, which is a key lake characteristic. We conducted web-searches to identify additional sources of lake depth data from lake associations, fishing maps and resources, and other state databases.

**Data sharing**

A key component to the success of a project such as this is data sharing. Where possible, we chose data in the public domain (e.g. state databases), which should, in theory, be readily accessible. Nevertheless, for all datasets that we requested, we offered data providers the option to collaborate with our team if they provided data, were interested in a particular research question, and if they agreed to the policies established by the NSF and our research group (see Additional file 18). We are happy to report that our requests for data were denied in very few cases.

**APPENDIX**

The Clean Water Act (CWA) provided that the discharge of pollutants to the waters of the US be regulated. Under the CWA the Environmental Protection Agency (EPA) implemented programs and have set water quality standards for contaminants in surface waters (<http://www2.epa.gov/laws-regulations/summary-clean-water-act>). Since the CWA was first passed in 1972, many other laws have changed parts of it. In addition, by the early 1980’s it became clear that greater attention was needed on water quality-based approach to pollution (<http://water.epa.gov/scitech/swguidance/standards/history.cfm>). In addition, the Water Quality Standards Regulation was amended in 1991 to expand the standards program to include Indian Tribes by letting them apply for the ability to set their own standards. Consequently, US states (and tribes, where applicable) have recognized the need to collect nutrient samples on their water bodies to monitor status and to help in setting standards in the first place. In addition, EPA required that state water quality standards be approved by the EPA on or after 2000 to be usable for regulatory purposes. States also are required to submit an integrated water quality monitoring and assessment report every even-numbered year to the EPA.

Based on the above legislation and reporting requirements, most US states have implemented some form of monitoring of the water bodies of the state. In doing so, states are required to carefully document their methods through the submission of a Quality Assurance Project Plan (QAPP).
