# Supplementary material for: Building a multi-scaled geospatial temporal ecology database from disparate data sources: fostering open science and data reuse
Source: Gigascience. 2015 Jul 1;4:28. doi: 10.1186/s13742-015-0067-4 (PMC4488039; doi:10.1186/s13742-015-0067-4)
Supplement: Additional file 18: — Example memo sent to a potential data provider requesting a water quality dataset. This file contains the memo that we sent to potential data providers requesting data that described the purpose of the study as well as the short- and long-term plans for the data. [file 13742_2015_67_MOESM18_ESM.docx]

Additional file 18

**Example memo sent to a potential data provider requesting a water quality dataset**

**MEMORANDUM**

**TO:**  *To be filled in*

**FROM**: PIs on NSF Macrosystem Biology Project (P.A. Soranno, K.S. Cheruvelil, E.H. Stanley, J.A. Downing, N.R. Lottig, P-N Tang)

**RE**: Lake data and metadata request

**DATE**: *To be filled in*

We are part of a project funded by the National Science Foundation studying large-scale and long-term dynamics of lakes. To this end, we are compiling lake chemistry, nutrient, and clarity data from a large geographic region that includes your state (please see the last page for the project description and map of the study area). We are hoping to obtain data for any lakes for which you have electronic records (and for all associated sampling dates). Although we recognize that some of these data are archived in Storet, we require fairly detailed metadata and thus are asking for data (and metadata) from you directly.

We greatly appreciate your help in these efforts and we would like to thank you in advance for your time and effort in helping us to develop one of the largest and detailed lake databases in the US. This project would not be possible without the contributions of individual agencies and personnel responsible for lake sampling and database maintenance. We also recognize the amount of work necessary to compile databases for distribution and are willing to help in any way that we can. We view this compilation effort as a great opportunity for these high quality databases to be put to additional uses and be made more accessible for future managers and researchers at the state level and beyond.

Please see the details below for the description of our database request and our policies related to data sharing, publishing and collaborations. One of the most critical components of your databases that we are asking for is **metadata associated with each database** so that we can determine the best approaches for data migration/integration and quality assurance/quality control protocols. We recognize that there are a variety of ways to handle metadata and will accept it in any form, be it in tables, reports that you have provided to EPA, etc. Because of NSF requirements, we will likely need to talk with you over the phone to fill in additional required metadata fields. Our goal is to make it as easy as possible for you to provide us with this important information.

Below is an overview of the database that we are requesting. We can accept data in almost any format (e.g. *excel, csv, txt, dbf, or any existing database*)*.* Please note that we require sampled (raw) data as opposed to seasonal or annual averages per lake. We are looking for ANY available sampling data from approximately 1975 – present, from weekly, monthly, or annual sampling. We will also gladly accept data from single sampling efforts (i.e., lakes for which only one sample has been taken across the entire time period).

Overview of requested database

**Lake chemistry/nutrient/clarity database**

*Description: Any available water quality variables by date and unique lake identifier. Extra variables are fine to include as well.*

**Lake information table**

*Description: A table for all lakes in your state for which you have chemistry/nutrient/water clarity data, for all sampling dates and years that you have electronic records. Included in this table should be a location field such as lat/lon, lake depth if available, and other distinguishing variables you might have.*

Data sharing, publishing, and new collaborations

As the database is created and for the duration of this project (from 2011-2016), the database will only be available to project personnel and closely affiliated collaborators. The National Science Foundation requires that all data associated with this study be made available to the public and scientific community upon completion of the project. We agree with such policies as they ensure that this large and potentially very useful database will be available to future researchers and lake managers in the hopes of improved understanding and management of lakes. At the end of the project, in 2016, the database will be deposited for online access in the North Temperate Lakes Long Term Ecological Research site’s database (http://lter.limnology.wisc.edu). However, for datasets that are not already in the public domain and for which data providers do not want the data to be distributed, we can choose to withhold such data. Please let us know if any of the data you share with us should not be made public and why.

We are interested in collaborations with personnel who know the data well. If there are particular aspects of our project that interest you, we welcome new collaborations. We have a working document that describes our manuscript co-authorship policy and would be happy to send it to you if you are interested in collaboration. Although providing data alone is not sufficient for co-authorship, we welcome those interested in contributing more to project products. Please let us know if you are interested in such possibilities or would like more information about our research efforts.

**Project Description**

The effect of cross-scale interactions on freshwater ecosystem state across space and time

PI’s: P.A. Soranno, K.S. Cheruvelil, E.H. Stanley, J.A. Downing, N.R. Lottig, P-N. Tang

*National Science Foundation, Macrosystems Biology Program, Emerging Frontiers Division.* 2011-2016.

**Summary**: We live in a rapidly changing environment, yet scientists’ understanding of the ecological consequences of wholesale changes in climate and land use is in its infancy. So too is the incorporation of this knowledge into environmental management and policy, which is so critical because both climate and land use strongly affect ecosystems and the services that they provide to society. The main goal of this research is to develop tools to measure and understand how climate and land use by themselves and as interacting factors affect lake ecosystems across scales of time and space (cross-scale interactions), even as these factors are themselves, changing. A cross-scale interaction occurs when a factor at one scale, such as agricultural land use around a lake, interacts with a factor at another scale, such as the climate of the region the lake is located within. Such interactions can lead to situations where lakes in different climatic zones respond differently to agricultural land use in their watersheds, all else being equal. Without an understanding of such interactions, it is challenging to develop and apply models that are effective in different regions. Unfortunately, to date, very few cross-scale interactions have been measured so that they can be incorporated into models relevant to ecosystems and policy. This project will identify and measure the most important cross-scale interactions that control lake nutrients and water quality. The research will be guided by a landscape limnology conceptual framework. Although the study focuses on lake nutrients, the models, tools, and knowledge will be useable to study cross-scale interactions in other important ecosystems. This collaborative team from three universities will collect an unprecedented dataset on lakes, nutrients, and watersheds, including ~6,000 lake ecosystems in 17 U.S. states spanning up to 30 years. Several new and innovative statistical modeling approaches will be used to tackle these important problems. For example, Bayesian hierarchical modeling (a robust statistical method for learning and modeling complex relationships in data) will be used to detect and model cross-scale interactions and to communicate these complex dynamics to other researchers and policy-makers.

Managing ecosystems as society intensifies changes in them requires new approaches, models, information, and skill sets. Identifying the conditions or the environments prone to cross-scale interactions is needed to forecast, manage, and repair damages from environmental change at local to regional scales. This project will help to develop these much-needed strategies and will change the way people view and conduct research on large-scale, living systems because of the project’s geographical scope, its foundation in a reliable conceptual framework, and its use of innovative statistical and numerical methods. The results will provide insight into these important ecosystems and problems beyond the lakes under study. This collaborative project across three institutions will also train a new generation of biologists who will know how to tackle broad-scaled research and policy problems. In addition, because the researchers will use commonly-measured lake water quality variables that are used to set water resources policy, results will directly inform the state and federal agencies responsible for lake and water management. Inclusion of several undergraduate, graduate, and post-doctoral researchers, along with state and federal agency partners will ensure the project’s success and improve the world’s capacity to manage our changing ecosystems.


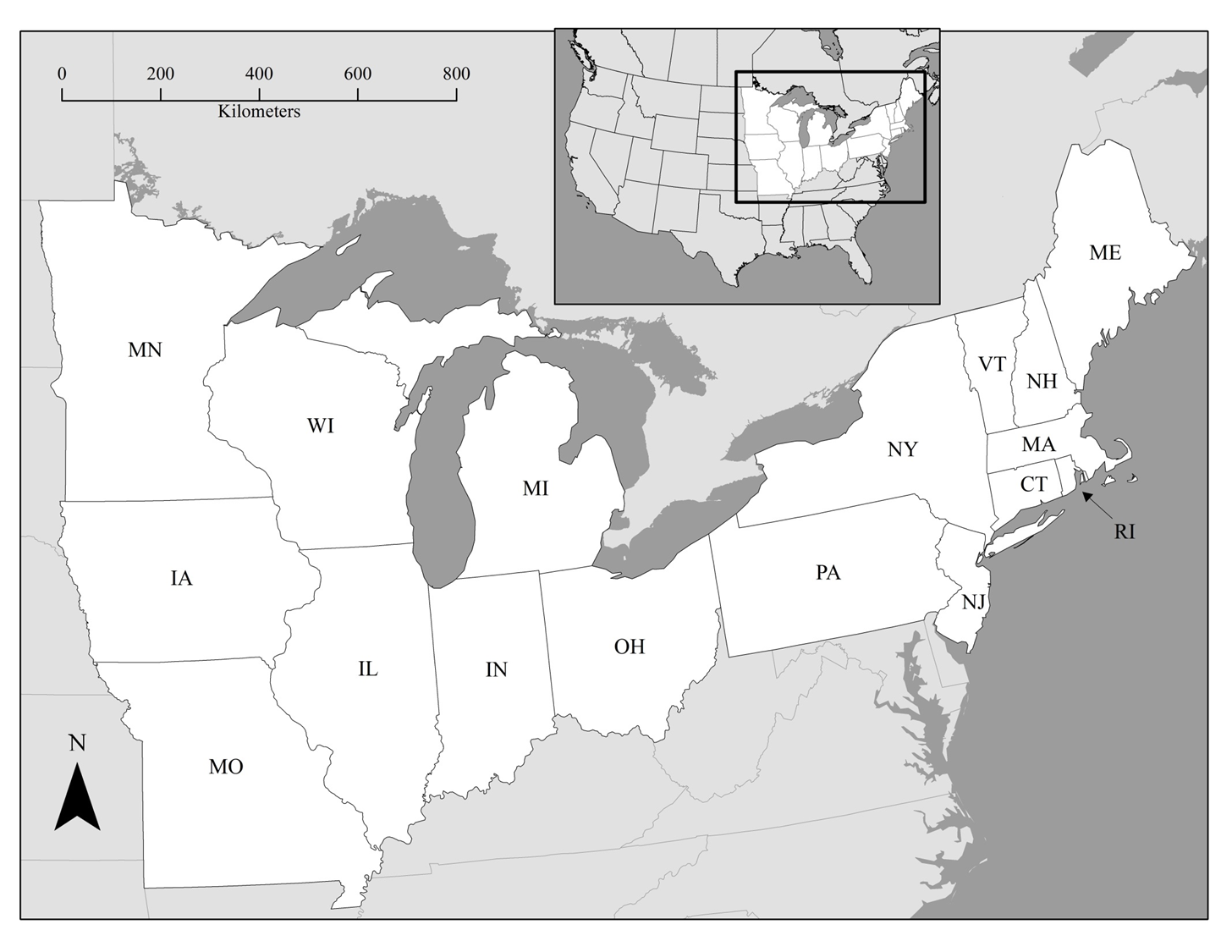


The 17 states shown in white will be included in this study.
