# Supplementary material for: Building a multi-scaled geospatial temporal ecology database from disparate data sources: fostering open science and data reuse
Source: Gigascience. 2015 Jul 1;4:28. doi: 10.1186/s13742-015-0067-4 (PMC4488039; doi:10.1186/s13742-015-0067-4)
Supplement: Additional file 19: — Procedure for converting individual water quality datasets into the LAGOSLIMNO schema. The steps that used to convert the heterogeneous water quality datasets into a common format to eventually import into LAGOS. We include an example script and log file created for each dataset prior to loading into LAGOSLIMNO. [file 13742_2015_67_MOESM19_ESM.docx]

Additional file 19

**Procedure for converting individual water quality datasets into the LAGOS_LIMNO_ schema**

Samuel Christel, Corinna Gries, Ed Bissell

**Overview**

Producing LAGOS_LIMNO_ required that all datasets we collected be manipulated into a single common format or 'dataset schema'. We used the scripting languages R and Python because they ensure data provenance. A script was written for each dataset. Each script documents all data manipulation steps that were taken to transform the source dataset (i.e., an individual dataset obtained from a unique source outside of our project) into the format required for the design of LAGOS. In addition, we recorded any decisions that were made during the data manipulation step (that could not be documented in the R or Python script) in a separate text log file. Finally, after converting the format of the source dataset to match the schema of LAGOS_LIMNO_, we saved the file in 'comma separated value' (csv) format'. Therefore, the data manipulation step produced three data products from the source dataset: (1) an R or Python script documenting all changes made to the source dataset, (2) a word processor document with all decisions made during data manipulation, but not detailed in the script, and (3) a csv file with the dataset manipulated to match the schema of LAGOS (Figure S24).


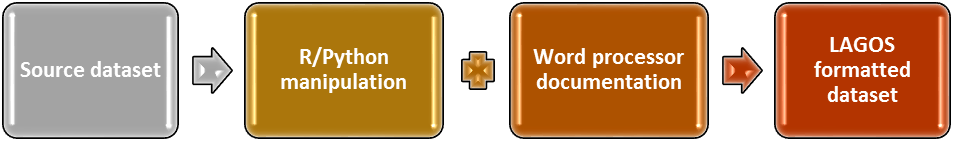


**Records for data provenance**

Figure S24. Overview of the steps to convert individual dataset formats into the LAGOS schema.

**Introduction**

Each individual dataset that we acquired had different formats, units, naming conventions and dataset structure. Therefore, a key component of our workflow was to convert all of the different individual datasets that we acquired into individual datasets with uniform formats, units, naming conventions, and dataset structure that could then be imported into LAGOS_LIMNO_. In other words, the datasets were converted into a common ontology (e.g., the concepts and relationships of the data, the units, and the naming conventions of the dataset) and a common dataset schema (e.g., the structure of the dataset itself). In addition, in many cases, we had to add information from the metadata files into the datasets themselves if they were missing, such as sample position or depth, if known, or sample type (e.g., grab or integrated). Because we sought to retain data provenance to the original data sources, we did the majority of any dataset manipulation using a scripting language (in most cases, we used R) so as to reproduce the datasets that were loaded into LAGOS_LIMNO_ and also to minimize the introduction of errors. Although this step could technically be performed by many members on the team, the majority of the datasets to be imported into LAGOS_LIMNO_ were manipulated by a single individual which had the advantage of making the process even more consistent given the highly non-standardized nature of the datasets.

**Definitions** (please see the Glossary for additional definitions)

- *Controlled vocabulary* -- A controlled vocabulary is an established list of standardized terminology for use in indexing and retrieval of information. An example of a controlled vocabulary is subject headings used to describe library resources (definition obtained from http://stats.oecd.org)
- *Data provenance* - a record that details how a dataset was produced, all changes that were made to a dataset, and any other details required to analyze a dataset.
- *Dataset schema* -- the structure of a dataset, i.e., characteristic arrangement of columns and rows that comprise a dataset.
- *Observations data model (ODM)* - A relational database design produced by the CUAHSI community: “The observations data model is designed to store hydrologic observations and sufficient ancillary information (metadata) about the data values to provide traceable heritage from raw measurements to usable information allowing them to be unambiguously interpreted and used. A relational database format is used to provide querying capability to allow data retrieval supporting diverse analyses.” Definition obtained from [1].
- *Ontology* -- A formal representation or classification of concepts and their relationships within a domain of interest

**Files**

*Dataset input, metadata, and controlled vocabulary*

1. A csv or Excel file of the original dataset - The original dataset to be converted into the needed format and ontology.
2. Individual metadata file (text document; see Additional file 3) - Detailed metadata for each individual dataset in EML format.
3. Controlled vocabulary (text document; see Additional file 4) **-** Provides the ontology that is needed for each individual dataset including the needed data columns that should be populated, and the allowed values for each column (i.e., the controlled vocabulary)
4. Integrated metadata file (spreadsheet; see Additional file 3) **-** Metadata is compiled for each dataset into a single Excel file to help fill in gaps in the datasets.

*Outputs of the file conversion procedure*

1. Data import log file (text document; see Appendix 1) **-** a word document that describes any decisions that were made in processing the datasets (see Appendix 1 for an example log entry).
2. R script for each dataset (text document; see Appendix 2) - The code that converts the dataset into the appropriate format, structure and ontology.
3. A csv file of the converted dataset (see 'converted dataset description' below) - The file that contains the converted individual datasets to be imported into LAGOS.

**LAGOS_LIMNO_ Data import procedures**

For many situations, individual datasets contained some variables that were not needed for import into the larger database. A list of 'priority' variables was therefore provided in the controlled vocabulary file above, and only those were loaded into the database. An R script was written that carried out all of the steps below using the raw dataset (in either csv or Excel formats) as the input; the final output of these steps was a .csv file conforming to the column definitions and ontologies described in the above 'ontology and controlled vocabulary file'. R script steps included:

1. Standardize the source data into the needed schema.
2. Identify and load only the priority variables.
3. Populate the values of the priority variables based on a combination of information in the source data file(s), the metadata (in a text file), and the 'integrated metadata file'.
4. Convert all units (including detection limit units) to the standardized units using formulas in the 'integrated metadata file'**.** Make a note in the 'comments' column when detection limits and/or specific standard methods are not provided in the data file itself but the metadata file indicates that a standard method was used.
5. Document all decisions in the data import logs (see Appendix 1 for an example log).

**Additional steps in the conversion procedure**

After many of the datasets were processed using the above steps, we identified two problems: duplicate observations and issues with designating the sample depth and position of observations. Therefore, we had to revisit our procedures, revise our strategy, and reprocess the datasets. We developed procedures to deal with these issues as follows:

Duplicate observations: We realized that for both the database schema and for statistically analyzing the database, duplicate observations would be challenging to adequately represent in LAGOS. Therefore, we decided to develop tools to remove duplicate observations. The definition of duplicate observations extends beyond simply filtering out those observations that are replicates (the original data import documentation already mandated that replicate observations be filtered out). We decided that an observation is 'unique' if it has unique values for programid, lagoslakeid, date, sample depth, sample position, lagosvariableid, and datavalue as compared to other observations in the dataset (See 'Converted dataset description' below for an explanation of these variable names). We wrote R code (Appendix 2) to determine whether or not an observation is unique based on the aforementioned criteria. Specifically, we created a new column called, 'Dup' that indicates whether or not an observation is duplicate, where NA= not duplicate and 1= duplicate. Thus, the original import code had to be updated and re-run to make these changes. As a final step, we recorded the total number of duplicates for each dataset in the word processor documentation.

Sample depth or position: We observed that a large number of datasets contained observations that had no information for *SamplePosition* and *SampleDepth*. Because sample depth is important for lake nutrient concentrations, we decided to put extra effort into addressing the absence of these data. We added code to the data conversion script to check for observations that were NA (null) for *SamplePosition* and *SampleDepth*. Then, for lakes with NA in both *SamplePosition* and *SampleDepth*, we developed criteria and strategies for assigning sample position to lakes using additional information after all data were loaded into LAGOS.

Controlled vocabulary values

1. ALL CAPS
2. Use an underscore ( _ ) instead of a space.
3. Column names with an asterisk are columns that are linked to a controlled vocabulary, i.e. only the provided values are allowed.
4. All values in a controlled vocabulary column should conform to the allowed data values and data types.
5. For Secchi measurements, *SamplePosition* should always be 'SPECIFIED', *SampleType* should always be INTEGRATED, SampleDepth should be NULL (NA).
6. *LabMethodName* should only be populated with specific laboratory method names from a well-known document describing common methods, e.g. APHA_4500PH, using the ALL CAPS no spaces convention.

Rules for filtering out data that we did not want to import into LAGOS:

1. Do not include data records that are NULL for the variable in question.
2. Do not include data records for variables that are not currently listed as a priority variable.
3. Do not include any data records without sample dates.
4. Do not include duplicate or replicate data values. The definition of a unique or non-duplicate record is: A row in a horizontally structured (every variable has its own column) table that is unique for these columns programid, lagoslakeid, date, sampledepth, sampleposition, lagosvariableid, datavalue.
5. Do not include data values that indicate that they are unfit for use.

**Converted dataset description**

Below are the columns that are in the final converted dataset for each limnological dataset. A '*' after the column name indicates a required column (i.e., the value cannot be NULL). In parentheses, we provide the most likely source for the information that populates this column.

- **LakeID*** (from the original data file) **-** unique lake identifier (or in some cases basin or station); this column is absolutely necessary and will be used to relate the limnology sample data to locational information in the GIS dataset.
- **Lakename** (from the original data file) **-** name of lake (not technically required, but it is useful to have it as a backup to locate the lake).
- **SourceVariableName*** (from the original data file or the integrated metadata file) **-** name of sampled variable/parameter in the source dataset.
- **SourceVariableDescription*** (from the integrated metadata file) **-** name of sampled variable/parameter in source dataset.
- **SourceFlags** (from the original data file) **-** these columns indicate issues related to the measurement/analysis of a specific sample and are too difficult to standardize across datasets. Therefore, they are retained for future use if necessary. If a particular data value has multiple flags, then all are specified in a single cell separated by commas. The explanations of the flags (if available) are provided in the comments column or provided in the log file. If a source dataset includes indications of data quality in a comments column but does not use an actual flag, then those comments are standardized as much as possible and populated in this column.
- **LagosVariableID*** (from the integrated metadata file) **-** unique integer identifier for LagosVariableName.
- **LagosVariableName*** (from the integrated metadata file) **-** full text name of the variable that was measured, observed, modeled, etc. from the LAGOS controlled vocabulary.
- **Value*** (from the original data file) **-** numeric data value.
- **Units*** (from the integrated metadata file) **-** standardized units for the 'value'.
- **CensorCode** (from the original data file or the integrated metadata file) **-** code indicating observation is censored ('greater than', 'less than', or 'not censored' use GT, LT, or NC, respectively).
- **DetectionLimit** (from the original data file or the integrated metadata file) **-** if applicable, the detection limit of the method used to quantify the value; if this value is null, then it is assumed to be not applicable or unknown. This value must be populated for each row if the information is available in the integrated metadata file.
- **Date*** (from the original data file) **-** date at which the sample was collected, stored in date format <YYYY-MM-DD> (no time).
- **LabMethodName** (from the integrated metadata file) **-** if available, the name of the laboratory method. If no named laboratory method is provided, but there is pertinent information about the method, then that is recorded in the next variable. If multiple laboratory methods were listed and it is unknown which method was used, then MULTIPLE is specified here and the names of laboratory methods separated by commas are provided in the LabMethodInfo
- **LabMethodInfo** (from the integrated metadata file) **-** used to store information related to the laboratory method; or additional named methods; or descriptive text that cannot be standardized in LabMethodName. This variable may contain information regarding type of probe or specific analysis equipment used.
- **SampleType*** (from the original data file or the integrated metadata file) **-** method of sample collection.
- **SamplePosition*** (from the original data file or the integrated metadata file) **-** the position in the water column that the sample was taken. Either SamplePosition or SampleDepth is required, but both can be provided if available.
- **SampleDepth*** (from the original data file or the integrated metadata file) **-** numeric depth at which the sample was taken if known. For an integrated sample, the deepest depth is used; but if both depths are provided, both depths are included.
- **MethodInfo** (from the integrated metadata file) **-** variable specific tag that differentiates distinct characteristics of a variable but does not warrant storage as a separate variable. There is only one of these values per variable; for example, currently, there is only one variable that has this column populated (SECCHI_VIEW or SECCHI_VIEW_UNKNOWN). This column functions like a flag indicating a potential issue with a data value. It should only be populated if a data value needs to be flagged, i.e. it will be NULL most of the time. For example, if it is known that a viewscope was NOT used for a Secchi value, then this column should be NULL.
- **SubProgram -** name of a distinct subproject, study, or sampling effort contained within a single dataset.
- **Comments** - descriptive textual information that describes the sampling event that cannot be standardized into a discrete set of terms.

**References**

1. Tarbonton, D.G., Horsburgh, J.S., Maidment, D.R., Whiteaker, T., Zaslavsky, I., Piasecki, M., Goodall, J., Valentine, D., Whitenack, T: **Development of a community hydrologic information system.** *18^th^ World IMACS/MODSIM Congress.* 2009.

**APPENDIX 1: Example data import log**

Data Import Log: MA_DEP_2005_2010

Update 10/25/2013🡪 I found that 30 observations out of 1802 are null for sample depth and position.

Update 10/21/2013🡪 In the initial import effort, I had used the source variable “Relative Sample Depth” to assign the lagos sample position (see notes below) of either “epi” or “hypo.” Note that this may not be entirely true because “surface” and “near bottom” do not necessarily correspond to “epi” and “hypo.” However, many observations are null for sample depth, and so I decided to use this approximation in the interest of having information on the sample position (rather than having a bunch of observations that are sample depth =NA and sample position= unknown­­). Note that in some cases this still resulted in observations NA for sample depth and unknown for sample position because there was no info in “Relative Sample Depth.”

There were a large number of secchi observations that I had originally filtered out. I went back and kept those observations because I had made in error in thinking they needed to be filtered out. The final number of secchi observations= 1304.

I also exported the definition of the source data flags to the comments field.

Duplicate observations were removed from the processed version of this dataset. A unique observation is defined as an observation that has unique values for programid, lagoslakeid, date, sample depth, sample position, lagosvariableid, and datavalue (for each row). I created a new column, “Dup” which determines whether or not an observation is duplicate, where NA= not duplicate and 1= duplicate. 1119 OBSERVATIONS WERE FLAGGED AS DUPLICATE OUT OF 1802.

1. General Notes

These data were collected by the MA Department of Environmental Protection = WQ monitoring of lakes to ensure compliance with the FWPCA. Lake typically sampled during the summer sampling (water recreational) season. Missing values represented by blank cells (NULL).

Metadata specifies that all SampleType are “GRAB” unless the variable is chlorophyll-a which may have 'range' specified (this is “INTEGRATED” for SampleType). Observations in “Water Body” were exported to lagos “LakeName” and observations in “Unique ID” to the lagos LakeID column.

“QC” indicates whether a sample is duplicate, routine, or a field blank, we are not interested in duplicate samples or field blanks—consequently, these were filtered out. Note that “Sample Depth” is in meters but sometimes is reported as a range (if integrated as for chlorophyll-a observations). In the case where “Sample Depth” is reported as a range, the lower depth was exported to the lagos SampleDepth and SampleType specified as “INTEGRATED.”

The “Station Description” field was used to specify the lagos BasinType. If “deep hole” or some variant was specified in “Station Description” then “PRIMARY” was exported to the lagos BasinType. If not then “UNKNOWN” was specified for BasinType.

“Relative Sample Depth” contains info on the lagos SamplePosition, where “surface” corresponds to the lagos “epi” and “near bottom” corresponds to the lagos “hypo.” Values of “**” in “Relative Sample Depth” indicate that the SamplePosition is unknown (null) for which obs. were assigned a value of “UNKNOWN” for SamplePosition. Observations with “--“in “Relative Sample Depth” corresponded to observations of SampleType “INTEGRATED.” For those observations “SPECIFIED” was exported to the lagos SamplePosition.

“Result Qualifiers” contains info on SourceFlags and CensorCode. Observations with “Result Qualifiers” of ##, **, ^^, and -- were filtered out before processing for lagos. Secchi disk observations have their own column of qualifiers entitled “Secchi Depth Qualifiers.”

The “Result” field also contains special characters such as “<” attached to the reported result. These specify the lagos CensorCode. See variable specific notes for further details.

“Result Qualifiers”=

## Censored data (i.e., data that has been discarded as unusable. Check qualifiers for explanation).

** Missing data (i.e., data that should have been reported, but are missing for some reason, e.g., probe malfunction, probe not used).

^^ No water (i.e., a special case of missing data due to dry/no water conditions).

-- No data (i.e., data not taken/not required/not calibrated for/otherwise not available)

a Accuracy as estimated at the lab via matrix spikes, PT sample recoveries, internal check standards and lab-fortified blanks did not meet project data quality objectives identified for program or in QAPP. Also used for Secchi depths where disk contacts lake bottom.

b Blank contamination in lab reagent blanks and/or field blank samples (indicating possible bias high and false positives)

d Precision of field duplicates (as RPD) did not meet project data quality objectives identified for program or in QAPP. Batched samples may also be affected.

e Reported data value not theoretically possible. Specifically used for bacteria data where colonies per unit volume for E. coli bacteria > fecal coliform bacteria, for lake Secchi and station depth data where a specific Secchi depth is greater than the reported station depth, for dissolved fractions greater than total (same parameter from the same sample bottle) , and for other incongruous or conflicting results

f Frequency of quality control duplicates did not meet data quality objectives identified for program or in QAPP

h Holding time violation (usually indicating possible bias low)

j 'Estimated' value; can be used for lab-related issues where certain lab QC criteria are not met and re-testing is not possible (as identified by the lab).

m Method SOP not followed, field and/or lab methodology only partially implemented or not implemented at all, due to complications with sample matrix (e.g. sediment in sample, floc formation), lab error (e.g. cross-contamination between samples), additional steps taken by the lab to deal with matrix complications, lost/unanalyzed samples, use of expired reagents, samples collected by others (non-DWM), misc. field errors, etc.

p Samples not preserved per SOP or analytical method requirements.

r Data may not be representative due to circumstances and/or conditions at the time of sampling. Statistical outliers are also qualified using "r". Note: This qualifier is NOT automatically applied to data collected under stagnant flow conditions.

s Field sheet recorded data were used in place of electronic data (i.e., no electronic records available).

t Tidal influence likely (not indicative of freshwater conditions) .

2. NH4

All steps in the general notes section apply to NH4. Not detection limit is specified in the metadata, and there are only grab samples here (no integrated). One observation had to be discarded because there was no info on sample depth or sample position.

3. Apparent Color

There were only 8 observations and after the filtering steps only 3 observations! These were still processed for lagos.

4. Chlorophyll a

Initially there were about 203 observations, but after filtering out the duplicates, field blanks, null observations, and values that were flagged in such a way that made them irrelevant… only 61 observations remained. After filtering out values for which there was no info about the depth or position—only 53 remained!! Note that in “Relative Sample Depth” the value “--“indicates that the sample is “INTEGRATED” rather than “GRAB.” All observations were assigned a SampleType of “INTEGRATED” as this was specified in the metadata. However, “Relative Sample Depth” actually specifies SamplePosition so where “--“ was observed in “Relative Sample Depth” “UNKNOWN” was exported to the lagos SamplePosition. The bottom depth of the depth range reported in the source data was specified for the lagos SampleDepth.

5. Dissolved Reactive Phosphorus

After filtering out observations that were null for the numeric result, and for which there was no information on the lagos sample depth or position, there were only three values remaining. All three of these observations were actually censored as being below what is probably the detection limit. After filtering only one unique LabMethodName remained, as opposed to the two unique methods specified in the metadata.

6. Nitrite + nitrate

After filtering out all observations per general notes there were only 17 observations to be processed for lagos. All other steps listed in general notes were followed in importing the observations.

7. Total nitrogen and Total Phosphorus

After filtering out all observations per general notes there were only 122 and 208 remaining observations for tn and tp; respectively. Note that total phosphorus has two unique analytical methods attached to observations.

8. Secchi disk

Secchi disk obs. have their own data flag column in this data set. The data in this column were used to specify the censor code: where “Secchi Depth Qualifiers” = “a” the disk hit the bottom so “GT” was exported to the lagos CensorCode. Otherwise “NA” was exported to the lagos CensorCode. The data flags in “Secchi Depth Qualifiers” were exported to the lagos SourceFlags column as well. The meaning of these flags is listed below in bold.

“a” = disk hit bottom

“e”= value not possible

Note that the other column with information on SourceFlags, “Results Qualifiers” contained flags irrelevant to secchi disk obs. so these were disregarded.

“Relative Sample Depth Meters” contained “--“for several obs. This specifies that the obs. is “INTEGRATED.” For the secchi disk data, all observations were assigned a value of “INTEGRATED” for the lagos SampleType. However, “Relative Sample Depth Meters” was used to specify sample position (epi or hypo). Those observations with a “Relative Sample Depth Meters” of “—“were assigned a sample position of “unknown”.

**APPENDIX 2: Example R code to convert a dataset into LAGOS_LIMNO_ format**

LAGOS_Template = data.frame(LakeID=character(0),

LakeName=character(0),

SourceVariableName=character(0),

SourceVariableDescription=character(0),

SourceFlags=character(0),

LagosVariableID=integer(0),

LagosVariableName=character(0),

Value=numeric(0),

Units=character(0),

CensorCode=character(0),

DetectionLimit=numeric(0),

Date=character(0),

LabMethodName=character(0),

LabMethodInfo=character(0),

SampleType=character(0),

SamplePosition=character(0),

SampleDepth=numeric(0),

MethodInfo=character(0),

BasinType=character(0),

Subprogram=character(0),

Comments=character(0),

Dup=numeric(0))

#################################### General Notes ###########################################

#QC.type contains "duplicates" that should be filtered out

#Sample.Depth...meters. may contain obs. reported as a range = "INTEGRATED"

#values should be filtered by Analyte since this is an ODM formatted db

#Result.Qualifers contains flags which signify data to be filtered out, DataFlag, and CensorCode

#look at special notes also

#

################################### Ammonia-N ################################

data=MA_DEP_2005_2010

names(data)#looking at data

#filter out columns required for this data import effort

data=data[,c(3:6,11:12,15:16,19:24)] #pulled out columns of iterest based on metadata and looking at unique value

names(data)

length(data$Analyte[which(data$Analyte=="Ammonia-N")]) #45 should remain after filtering

data=data[which(data$Analyte=="Ammonia-N"),]

unique(data$QC.type) #filter out "Duplicate"

length(data$QC.type[which(data$QC.type=="Duplicate")]) #18

#filter out duplicates

45-18 #27 should remain

data=data[which(data$QC.type!="Duplicate"),]

#looking at data to understand it

unique(data$Result.Qualifers) #no unique qualifers that suggest data needs to be filtered out

unique(data$Result) #note that their are unique characters specifying CensorCode

unique(data$Analysis.Method) #only one unique method

unique(data$Sample.Depth...meters.) # ** represents not reported

length(data$Sample.Depth...meters.[which(data$Sample.Depth...meters.=="**")]) #21 do not have a depth specified

unique(data$Relative.Sample.Depth..meters.) #reveals sample position

length(data$Relative.Sample.Depth..meters.[which(data$Relative.Sample.Depth..meters.=="Near bottom")]) #8

length(data$Relative.Sample.Depth..meters.[which(data$Relative.Sample.Depth..meters.=="Surface")]) #18

length(data$Relative.Sample.Depth..meters.[which(data$Relative.Sample.Depth..meters.=="**")]) #1 is null

18+8+1

###start populating the lagos template

data.Export= LAGOS_Template

data.Export[1:nrow(data),]=NA

data.Export$LakeID = data$Unique.ID

data.Export$LakeName = data$Water.Body

data.Export$SourceVariableName = "Ammonia-N"

data.Export$SourceVariableDescription = "Ammonia"

#populate SourceFlags

unique(data$Result.Qualifers) #export to source flags

data.Export$SourceFlags=as.character(data.Export$SourceFlags)

data.Export$SourceFlags=data$Result.Qualifers

data.Export$SourceFlags[which(data.Export$SourceFlags=="")]= NA

unique(data.Export$SourceFlags)

#MAKE SURE ADDS TO TOTAL

length(data.Export$SourceFlags[which(is.na(data.Export$SourceFlags)==TRUE)])

length(data.Export$SourceFlags[which(is.na(data.Export$SourceFlags)==FALSE)])

25+1 #adds up

#continue populating other lagos variables

data.Export$LagosVariableID = 19

data.Export$LagosVariableName="Nitrogen, NH4"

#populate CensorCode

data.Export$CensorCode=as.character(data.Export$CensorCode)

unique(data$Result)

length(data$Result[which(data$Result=="<0.02")]) #14 are "LT"

data.Export$CensorCode[which(data$Result=="<0.02")]="LT"

data.Export$CensorCode[which(is.na(data.Export$CensorCode)==TRUE)]= "NC"

unique(data.Export$CensorCode)

#overwrite special characters

data$Result[which(data$Result=="<0.02")]=0.02

unique(data$Result) #check to make sure "<" gone

#continue with others

#note that several conversions had to be made with data type (below) in order to get the right values to export

data.Export$Value=as.character(data.Export$Value)

data$Result=as.character(data$Result)

names(data)

data.Export$Value = data[,11] #export ammonia obs.

unique(data.Export$Value)

data.Export$Value=as.numeric(data.Export$Value)

unique(data.Export$Value)

data.Export$Value = (data.Export$Value)*1000 #convert from mg/l to ug/l, the preff. units

unique(data.Export$Value)

typeof(data.Export$Value)

#continue with other columns

data.Export$Date = data$Sample.Date #date already in correct format

data.Export$Units="ug/L"

#prepare to populate sampletype

data.Export$SampleType=as.character(data.Export$SampleType)

data.Export$SampleType="GRAB" #specied in metadata table,

length(data.Export$SampleType[which(data.Export$SampleType=="GRAB")]) #all obs. populated

#populate sampelposition

data.Export$SamplePosition=as.character(data.Export$SamplePosition)

#first determine number of obs. of each sample position type

unique(data$Relative.Sample.Depth..meters.)

length(data$Relative.Sample.Depth..meters.[which(data$Relative.Sample.Depth..meters.=="Near bottom")])#set these as hypo = 8

length(data$Relative.Sample.Depth..meters.[which(data$Relative.Sample.Depth..meters.=="Surface")])#set these as epi=18

#populate sample position

data.Export$SamplePosition[which(data$Relative.Sample.Depth..meters.=="Surface")]= "EPI"

data.Export$SamplePosition[which(data$Relative.Sample.Depth..meters.=="Near bottom")]= "HYPO"

#check to make sure numbers add up

length(data.Export$SamplePosition[which(data.Export$SamplePosition=="EPI")])

length(data.Export$SamplePosition[which(data.Export$SamplePosition=="HYPO")])

#numbers add up proceed to populating sample depth

#assign sampledepth

unique(data$Sample.Depth...meters.)

data$Sample.Depth...meters.[which(data$Sample.Depth...meters.=="**")]=NA #set null to NA

unique(data$Sample.Depth...meters.)

data.Export$SampleDepth=data$Sample.Depth...meters.

#continue populating other lagos fields

data.Export$BasinType=as.character(data.Export$BasinType)

unique(data$Station.Description)

data.Export$BasinType[grep("deep",data$Station.Description,ignore.case=TRUE)]="PRIMARY"

data.Export$BasinType[which(is.na(data.Export$BasinType)==TRUE)]="UNKNOWN"

unique(data.Export$BasinType)

#check to make sure all obs. assigned a basin type

length(data.Export$BasinType[which(data.Export$BasinType=="PRIMARY")])

length(data.Export$BasinType[which(data.Export$BasinType=="UNKNOWN")])

#continue with other fields

data.Export$MethodInfo = as.character(data.Export$MethodInfo)

data.Export$MethodInfo = NA

data.Export$LabMethodName= as.character(data.Export$LabMethodName)

data.Export$LabMethodName= "EPA_350.1" #per emi's metadata

data.Export$LabMethodInfo=as.character(data.Export$LabMethodInfo)

data.Export$LabMethodInfo=NA

data.Export$DetectionLimit= NA #no info in meta

unique(data$Special.Notes)

data.Export$Comments=as.character(data.Export$Comments)

unique(data.Export$SourceFlags)

data.Export$Comments[which(data.Export$SourceFlags=="f")]="f=Frequency of quality control duplicates did not meet data quality objectives identified for program or in QAPP"

unique(data.Export$Comments)

nh4.Final = data.Export

rm(data.Export)

rm(data)

rm(temp.df)

################################### Apparent Color ################################

data=MA_DEP_2005_2010

names(data)#looking at data

#filter out columns required for this data import effort

data=data[,c(3:6,11:12,15:16,19:24)] #pulled out columns of iterest based on metadata and looking at unique value

names(data)

length(data$Analyte[which(data$Analyte=="Apparent Color")]) #8 should remain after filtering

data=data[which(data$Analyte=="Apparent Color"),]

unique(data$QC.type) #filter out "Duplicate"

length(data$QC.type[which(data$QC.type=="Duplicate")]) #4

#filter out duplicates

data=data[which(data$QC.type!="Duplicate"),]

#looking at data to understand it

unique(data$Result.Qualifers) #no unique qualifers that suggest data needs to be filtered out

unique(data$Result) #note that their are unique characters specifying CensorCode

length(data$Result[which(data$Result=="**")])#filter that out

length(data$Result[which(data$Result=="")]) #none are null

data=data[which(data$Result!="**"),]

unique(data$Analysis.Method) #only one unique method

unique(data$Sample.Depth...meters.) # ** represents not reported

length(data$Sample.Depth...meters.[which(data$Sample.Depth...meters.=="**")]) #all 3 do not have a depth specified

unique(data$Relative.Sample.Depth..meters.) #reveals sample position

#ignore warning message

###start populating the lagos template

data.Export= LAGOS_Template

data.Export[1:nrow(data),]=NA

data.Export$LakeID = data$Unique.ID

data.Export$LakeName = data$Water.Body

data.Export$SourceVariableName = "Apparent Color"

data.Export$SourceVariableDescription = "Apparent color"

#populate SourceFlags

unique(data$Result.Qualifers) #no unique qualifiers

data.Export$SourceFlags=NA

unique(data.Export$SourceFlags)

#continue populating other lagos variables

data.Export$LagosVariableID = 11

data.Export$LagosVariableName="Color, apparent"

#populate CensorCode

data.Export$CensorCode=as.character(data.Export$CensorCode)

unique(data$Result) #none are censored

data.Export$CensorCode= NA

unique(data.Export$CensorCode)

#continue with others

names(data)

data$Result=as.character(data$Result)

data.Export$Value=as.character(data.Export$Value)

data.Export$Value= data[,11] #export obs.alreadyi n PCU = preff. units

typeof(data.Export$Value)

unique(data.Export$Value)

data.Export$Value=as.numeric(data.Export$Value)

unique(data.Export$Value)

#continue with other columns

data.Export$Date = data$Sample.Date #date already in correct format

data.Export$Units="PCU"

#prepare to populate sampletype

data.Export$SampleType=as.character(data.Export$SampleType)

data.Export$SampleType="GRAB" #specied in metadata table,

length(data.Export$SampleType[which(data.Export$SampleType=="GRAB")]) #all obs. populated

#populate sampelposition

data.Export$SamplePosition=as.character(data.Export$SamplePosition)

#first determine number of obs. of each sample position type

unique(data$Relative.Sample.Depth..meters.)

length(data$Relative.Sample.Depth..meters.[which(data$Relative.Sample.Depth..meters.=="Surface")])#set these as epi=18

#populate sample position

data.Export$SamplePosition[which(data$Relative.Sample.Depth..meters.=="Surface")]= "EPI"

#check to make sure numbers add up

length(data.Export$SamplePosition[which(data.Export$SamplePosition=="EPI")])

#numbers add up proceed to populating sample depth

#assign sampledepth

unique(data$Sample.Depth...meters.) #no depth specified here, all NA

data.Export$SampleDepth=NA

#continue populating other lagos fields

data.Export$BasinType=as.character(data.Export$BasinType)

unique(data$Station.Description)

data.Export$BasinType[grep("deep",data$Station.Description,ignore.case=TRUE)]="PRIMARY"

data.Export$BasinType[which(is.na(data.Export$BasinType)==TRUE)]="UNKNOWN"

unique(data.Export$BasinType)

#check to make sure all obs. assigned a basin type

length(data.Export$BasinType[which(data.Export$BasinType=="PRIMARY")])

length(data.Export$BasinType[which(data.Export$BasinType=="UNKNOWN")])

#continue with other fields

data.Export$MethodInfo = as.character(data.Export$MethodInfo)

data.Export$MethodInfo = NA

data.Export$LabMethodName= as.character(data.Export$LabMethodName)

data.Export$LabMethodName= "SM_2120B" #per emi's metadata

data.Export$LabMethodInfo=as.character(data.Export$LabMethodInfo)

data.Export$LabMethodInfo=NA

data.Export$DetectionLimit= NA #no info in meta

unique(data$Special.Notes)

data.Export$Comments=as.character(data.Export$Comments)

data.Export$Comments= NA

acolor.Final = data.Export

rm(data.Export)

rm(data)

################################### Chlorophyll a ################################

data=MA_DEP_2005_2010

names(data)#looking at data

#filter out columns required for this data import effort

data=data[,c(3:6,11:12,15:16,19:24)] #pulled out columns of iterest based on metadata and looking at unique value

names(data)

length(data$Analyte[which(data$Analyte=="Chlorophyll a")]) #203 should remain after filtering

data=data[which(data$Analyte=="Chlorophyll a"),]

unique(data$QC.type) #filter out "Duplicate"

length(data$QC.type[which(data$QC.type=="Duplicate")]) #100 are dupicates

length(data$QC.type[which(data$QC.type=="Field Blank")]) #25 are field blanks

203-100-25 #ony 78 obs. should remain

#filter out duplicates and field blanks

data=data[which(data$QC.type!="Duplicate"),]

data=data[which(data$QC.type!="Field Blank"),]

#looking at data to understand it

unique(data$Result.Qualifers) #no unique qualifers that suggest data needs to be filtered out

length(data$Result.Qualifers[which(data$Result.Qualifers==" --")]) #15 need to be filtered out

78-15 #should be left with 63 obs.

data=data[which(data$Result.Qualifers!=" --"),]

unique(data$Result) #note that their are unique characters specifying CensorCode

length(data$Result[which(data$Result=="##")]) #filter these out, null

data=data[which(data$Result!="##"),]

63-2 #should be left with 61

unique(data$Analysis.Method) #only one unique method

unique(data$Sample.Depth...meters.) # ** represents not reported

length(data$Sample.Depth...meters.[which(data$Sample.Depth...meters.=="**")]) #13 do not have a depth specified

#most but not all have a depth reported as a range

unique(data$Relative.Sample.Depth..meters.) #reveals sample position

length(data$Relative.Sample.Depth..meters.[which(data$Relative.Sample.Depth..meters.=="Near bottom")]) #1

length(data$Relative.Sample.Depth..meters.[which(data$Relative.Sample.Depth..meters.=="Surface")]) #8

length(data$Relative.Sample.Depth..meters.[which(data$Relative.Sample.Depth..meters.=="--")]) #44 are integrated thats what "--" signifies

length(data$Relative.Sample.Depth..meters.[which(data$Relative.Sample.Depth..meters.==" --")]) #another 8 integrated

data$Sample.Depth...meters.=as.character(data$Sample.Depth...meters.)

unique(data$Sample.Depth...meters.)

data$Relative.Sample.Depth..meters.=as.character(data$Relative.Sample.Depth..meters.)

unique(data$Relative.Sample.Depth..meters.)

temp.df=data[which(data$Sample.Depth...meters.=="**" & data$Relative.Sample.Depth..meters.=="--"),] #these observations must be filtered out

#remove these at the end.

#no other filtering

###start populating the lagos template

data.Export= LAGOS_Template

data.Export[1:nrow(data),]=NA

data.Export$LakeID = data$Unique.ID

data.Export$LakeName = data$Water.Body

data.Export$SourceVariableName = "Chlorophyll a"

data.Export$SourceVariableDescription = "Chlorophyll a"

#populate SourceFlags

unique(data$Result.Qualifers) #export to source flags

data.Export$SourceFlags=as.character(data.Export$SourceFlags)

data.Export$SourceFlags=data$Result.Qualifers

data.Export$SourceFlags[which(data.Export$SourceFlags=="")]= NA

#check to make sure adds up to total

length(data.Export$SourceFlags[which(is.na(data.Export$SourceFlags)==TRUE)])

length(data.Export$SourceFlags[which(is.na(data.Export$SourceFlags)==FALSE)])

40+13 #adds up to totall

unique(data.Export$SourceFlags)

#continue populating other lagos variables

data.Export$LagosVariableID = 9

data.Export$LagosVariableName="Chlorophyll a"

#populate CensorCode

data.Export$CensorCode=as.character(data.Export$CensorCode)

unique(data$Result)

length(data$Result[which(data$Result=="<1.0")]) #1 obs is "LT"

data.Export$CensorCode[which(data$Result=="<1.0")]="LT"

data.Export$CensorCode[which(is.na(data.Export$CensorCode)==TRUE)]= "NC"

unique(data.Export$CensorCode)

#overwrite special characters

data$Result[which(data$Result=="<1.0")]=1.0

unique(data$Result) #check to make sure "<" gone

#continue with others

#note that several conversions had to be made with data type (below) in order to get the right values to export

unique(data$Units) #note that mg/m3 is equal to the preff. units of ug/L.

data.Export$Value=as.character(data.Export$Value)

data$Result=as.character(data$Result)

data.Export$Value = data[,11] #export chla obs.

unique(data.Export$Value)

data.Export$Value=as.numeric(data.Export$Value)

#continue with other columns

data.Export$Date = data$Sample.Date #date already in correct format

data.Export$Units="ug/L"

#prepare to populate sampletype

data.Export$SampleType=as.character(data.Export$SampleType)

data.Export$SampleType="INTEGRATED" #specied in metadata table & metadata

length(data.Export$SampleType[which(data.Export$SampleType=="INTEGRATED")]) #all obs. populated

#populate sampelposition

data.Export$SamplePosition=as.character(data.Export$SamplePosition)

#first determine number of obs. of each sample position type

unique(data$Relative.Sample.Depth..meters.)

length(data$Relative.Sample.Depth..meters.[which(data$Relative.Sample.Depth..meters.=="Near bottom")])#set these as hypo = 8

length(data$Relative.Sample.Depth..meters.[which(data$Relative.Sample.Depth..meters.=="Surface")])#set these as epi=18

length(data$Relative.Sample.Depth..meters.[which(data$Relative.Sample.Depth..meters.=="--")]) #unknown

#populate sample position

unique(data$Relative.Sample.Depth..meters.)

data.Export$SamplePosition[which(data$Relative.Sample.Depth..meters.=="Surface")]= "EPI"

data.Export$SamplePosition[which(data$Relative.Sample.Depth..meters.=="Near bottom")]= "HYPO"

data.Export$SamplePosition[which(data$Relative.Sample.Depth..meters.=="--")]= "UNKNOWN"

data.Export$SamplePosition[which(data$Relative.Sample.Depth..meters.==" --")]= "UNKNOWN"

#check to make sure numbers add up

length(data.Export$SamplePosition[which(data.Export$SamplePosition=="EPI")])

length(data.Export$SamplePosition[which(data.Export$SamplePosition=="HYPO")])

length(data.Export$SamplePosition[which(data.Export$SamplePosition=="UNKNOWN")])

52+8+1 #adds up to total

#numbers add up proceed to populating sample depth

#assign sampledepth

unique(data$Sample.Depth...meters.)

length(data$Sample.Depth...meters.[which(data$Sample.Depth...meters.=="**")]) #13 are NA

#the following code pulls out the number and ignores the "0.0-"...so only the lower depth is exported to the lagos sample depth

data.Export$SampleDepth[which(data.Export$SampleDepth=="**")]="" #export the null obs. as NA

data.Export$SampleDepth[grep("7.5",data$Sample.Depth...meters.,ignore.case=TRUE)]="7.5"

data.Export$SampleDepth[grep("6.0",data$Sample.Depth...meters.,ignore.case=TRUE)]="6.0"

data.Export$SampleDepth[grep("4.0",data$Sample.Depth...meters.,ignore.case=TRUE)]="4.0"

data.Export$SampleDepth[grep("3.5",data$Sample.Depth...meters.,ignore.case=TRUE)]="3.5"

data.Export$SampleDepth[grep("5.0",data$Sample.Depth...meters.,ignore.case=TRUE)]="5.0"

data.Export$SampleDepth[grep("2.2",data$Sample.Depth...meters.,ignore.case=TRUE)]="2.2"

data.Export$SampleDepth[grep("2.0",data$Sample.Depth...meters.,ignore.case=TRUE)]="2.0"

data.Export$SampleDepth[grep("1.8",data$Sample.Depth...meters.,ignore.case=TRUE)]="1.8"

data.Export$SampleDepth[grep("8.5",data$Sample.Depth...meters.,ignore.case=TRUE)]="8.5"

data.Export$SampleDepth[grep("4.2",data$Sample.Depth...meters.,ignore.case=TRUE)]="4.2"

data.Export$SampleDepth[grep("3.6",data$Sample.Depth...meters.,ignore.case=TRUE)]="3.6"

data.Export$SampleDepth[grep("3.7",data$Sample.Depth...meters.,ignore.case=TRUE)]="3.7"

data.Export$SampleDepth[grep("3.4",data$Sample.Depth...meters.,ignore.case=TRUE)]="3.4"

data.Export$SampleDepth[grep("3.2",data$Sample.Depth...meters.,ignore.case=TRUE)]="3.2"

data.Export$SampleDepth[grep("3.8",data$Sample.Depth...meters.,ignore.case=TRUE)]="3.8"

data.Export$SampleDepth[grep("4.5",data$Sample.Depth...meters.,ignore.case=TRUE)]="4.5"

data.Export$SampleDepth[grep("3.1",data$Sample.Depth...meters.,ignore.case=TRUE)]="3.1"

data.Export$SampleDepth[grep("2.3",data$Sample.Depth...meters.,ignore.case=TRUE)]="2.3"

data.Export$SampleDepth[grep("2.5",data$Sample.Depth...meters.,ignore.case=TRUE)]="2.5"

data.Export$SampleDepth[grep("0.9",data$Sample.Depth...meters.,ignore.case=TRUE)]="0.9"

data.Export$SampleDepth[grep("7.0",data$Sample.Depth...meters.,ignore.case=TRUE)]="7.0"

data.Export$SampleDepth[grep("6.6",data$Sample.Depth...meters.,ignore.case=TRUE)]="6.6"

data.Export$SampleDepth[grep("6.3",data$Sample.Depth...meters.,ignore.case=TRUE)]="6.3"

data.Export$SampleDepth[grep("3.0",data$Sample.Depth...meters.,ignore.case=TRUE)]="3.0"

data.Export$SampleDepth[grep("1.2",data$Sample.Depth...meters.,ignore.case=TRUE)]="1.2"

data.Export$SampleDepth[grep("5.4",data$Sample.Depth...meters.,ignore.case=TRUE)]="5.4"

data.Export$SampleDepth[grep("2.4",data$Sample.Depth...meters.,ignore.case=TRUE)]="2.4"

data.Export$SampleDepth[grep("8.5",data$Sample.Depth...meters.,ignore.case=TRUE)]="8.5"

unique(data.Export$SampleDepth)

length(data.Export$SampleDepth[which(is.na(data.Export$SampleDepth)==TRUE)]) #13 are NA as expected

data.Export$SampleDepth=as.numeric(data.Export$SampleDepth)

#continue populating other lagos fields

data.Export$BasinType=as.character(data.Export$BasinType)

unique(data$Station.Description)

data.Export$BasinType[grep("deep",data$Station.Description,ignore.case=TRUE)]="PRIMARY"

data.Export$BasinType[which(is.na(data.Export$BasinType)==TRUE)]="UNKNOWN"

unique(data.Export$BasinType)

#check to make sure all obs. assigned a basin type

length(data.Export$BasinType[which(data.Export$BasinType=="PRIMARY")])

length(data.Export$BasinType[which(data.Export$BasinType=="UNKNOWN")])

#continue with other fields

data.Export$MethodInfo = as.character(data.Export$MethodInfo)

data.Export$MethodInfo = NA

data.Export$LabMethodName= as.character(data.Export$LabMethodName)

data.Export$LabMethodName= "EPA_445" #per emi's metadata

data.Export$LabMethodInfo=as.character(data.Export$LabMethodInfo)

data.Export$LabMethodInfo=NA

data.Export$DetectionLimit= NA #no info in meta

unique(data$Special.Notes)

unique(data.Export$SourceFlags)

data.Export$Comments=as.character(data.Export$Comments)

unique(data.Export$SourceFlags)

data.Export$Comments[which(data.Export$SourceFlags=="f")]="f=Frequency of quality control duplicates did not meet data quality objectives identified for program or in QAPP"

data.Export$Comments[which(data.Export$SourceFlags=="j")]="j='Estimated' value; can be used for lab-related issues where certain lab QC criteria are not met and re-testing is not possible (as identified by the lab)."

data.Export$Comments[which(data.Export$SourceFlags=="d")]="d=Precision of field duplicates (as RPD) did not meet project data quality objectives identified for program or in QAPP. Batched samples may also be affected."

data.Export$Comments[which(data.Export$SourceFlags=="m")]="m=Method SOP not followed, field and/or lab methodology only partially implemented or not implemented at all, due to complications with sample matrix (e.g. sediment in sample, floc formation), lab error (e.g. cross-contamination between samples), additional steps taken by the lab to deal with matrix complications, lost/unanalyzed samples, use of expired reagents, samples collected by others (non-DWM), misc. field errors, etc."

data.Export$Comments[which(data.Export$SourceFlags=="h")]="h=Holding time violation (usually indicating possible bias low)"

unique(data.Export$Comments)

chla.Final = data.Export

rm(data.Export)

rm(data)

################################### Dissolved Reactive Phosphorus#############################################################################################################################

data=MA_DEP_2005_2010

names(data)#looking at data

#filter out columns required for this data import effort

data=data[,c(3:6,11:12,15:16,19:24)] #pulled out columns of iterest based on metadata and looking at unique value

names(data)

length(data$Analyte[which(data$Analyte=="Dissolved Reactive Phosphorus")]) #19 should remain after filtering

data=data[which(data$Analyte=="Dissolved Reactive Phosphorus"),]

unique(data$QC.type) #filter out "Duplicate"

length(data$QC.type[which(data$QC.type=="Duplicate")]) #8 are dupicates

length(data$QC.type[which(data$QC.type=="Field Blank")]) #0 are field blanks

19-8 #only 11 obs. should remain

#filter out duplicates and field blanks

data=data[which(data$QC.type!="Duplicate"),]

data=data[which(data$QC.type!="Field Blank"),]

#looking at data to understand it

unique(data$Result.Qualifers) #no unique qualifers that suggest data needs to be filtered out

unique(data$Result) #note that their are unique characters specifying CensorCode

length(data$Result[which(data$Result=="<0.005")]) #specify censor code for these

length(data$Result[which(data$Result=="**")]) #filter these out

data=data[which(data$Result!="**"),]

unique(data$Analysis.Method) #only one unique method

unique(data$Sample.Depth...meters.) # ** represents not reported

length(data$Sample.Depth...meters.[which(data$Sample.Depth...meters.=="**")]) #all obs. do not have a depth specified

#most but not all have a depth reported as a range

unique(data$Relative.Sample.Depth..meters.) #reveals sample position

length(data$Relative.Sample.Depth..meters.[which(data$Relative.Sample.Depth..meters.=="Near bottom")]) #1

length(data$Relative.Sample.Depth..meters.[which(data$Relative.Sample.Depth..meters.=="Surface")]) #3

length(data$Relative.Sample.Depth..meters.[which(data$Relative.Sample.Depth..meters.=="**")]) #2 are null thats what "**" signifies

#no other filtering

###start populating the lagos template

data.Export= LAGOS_Template

data.Export[1:nrow(data),]=NA

data.Export$LakeID = data$Unique.ID

data.Export$LakeName = data$Water.Body

data.Export$SourceVariableName = "Dissolved Reactive Phosphorus"

data.Export$SourceVariableDescription = "Dissolved reactive phosphorus"

#populate SourceFlags

unique(data$Result.Qualifers) #export to source flags

data.Export$SourceFlags=as.character(data.Export$SourceFlags)

data.Export$SourceFlags=data$Result.Qualifers

data.Export$SourceFlags[which(data.Export$SourceFlags=="")]= NA

#check to make sure adds up to total

length(data.Export$SourceFlags[which(is.na(data.Export$SourceFlags)==TRUE)])

length(data.Export$SourceFlags[which(is.na(data.Export$SourceFlags)==FALSE)])

3+2 #adds up to totall

unique(data.Export$SourceFlags)

#continue populating other lagos variables

data.Export$LagosVariableID = 26

data.Export$LagosVariableName="Phosphorus, soluable reactive orthophosphate"

#populate CensorCode

data.Export$CensorCode=as.character(data.Export$CensorCode)

unique(data$Result)

length(data$Result[which(data$Result=="<0.005")]) #3 obs is "LT"

data.Export$CensorCode[which(data$Result=="<0.005")]="LT"

data.Export$CensorCode[which(is.na(data.Export$CensorCode)==TRUE)]= "NC"

unique(data.Export$CensorCode)

#overwrite special characters

data$Result[which(data$Result=="<0.005")]=0.005

unique(data$Result) #check to make sure "<" gone

#continue with others

#note that several conversions had to be made with data type (below) in order to get the right values to export

unique(data$Units) #convert to ug/L (lagos preff. units)

data.Export$Value=as.character(data.Export$Value)

data$Result=as.character(data$Result)

names(data)

data.Export$Value = data[,11] #export drp obs.

unique(data.Export$Value)

data.Export$Value=as.numeric(data.Export$Value)

data.Export$Value=(data.Export$Value)*1000

#continue with other columns

data.Export$Date = data$Sample.Date #date already in correct format

data.Export$Units="ug/L"

#prepare to populate sampletype

data.Export$SampleType=as.character(data.Export$SampleType)

data.Export$SampleType="GRAB" #specied in metadata table & metadata

length(data.Export$SampleType[which(data.Export$SampleType=="GRAB")]) #all obs. populated

#populate sampelposition

data.Export$SamplePosition=as.character(data.Export$SamplePosition)

#first determine number of obs. of each sample position type

unique(data$Relative.Sample.Depth..meters.)

length(data$Relative.Sample.Depth..meters.[which(data$Relative.Sample.Depth..meters.=="Near bottom")])#set these as hypo = 0

length(data$Relative.Sample.Depth..meters.[which(data$Relative.Sample.Depth..meters.=="Surface")])#set these as epi=3

length(data$Relative.Sample.Depth..meters.[which(data$Relative.Sample.Depth..meters.=="--")]) #0 are integrated, set these as "Unknown" for sample position

length(data$Relative.Sample.Depth..meters.[which(data$Relative.Sample.Depth..meters.=="**")])#set to unknown

#populate sample position

data.Export$SamplePosition[which(data$Relative.Sample.Depth..meters.=="Surface")]= "EPI"

data.Export$SamplePosition[which(data$Relative.Sample.Depth..meters.=="Near bottom")]= "HYPO"

data.Export$SamplePosition[which(data$Relative.Sample.Depth..meters.=="**")]= "UNKNOWN"

#check to make sure numbers add up

length(data.Export$SamplePosition[which(data.Export$SamplePosition=="EPI")])

length(data.Export$SamplePosition[which(data.Export$SamplePosition=="HYPO")])

length(data.Export$SamplePosition[which(data.Export$SamplePosition=="UNKNOWN")])

3+0+0 #adds up to total

#numbers add up proceed to populating sample depth

#assign sampledepth

unique(data$Sample.Depth...meters.)

length(data$Sample.Depth...meters.[which(data$Sample.Depth...meters.=="**")])

data.Export$SampleDepth=data$Sample.Depth...meters.

data.Export$SampleDepth[which(data.Export$SampleDepth=="**")]=NA

length(data.Export$SampleDepth[which(is.na(data.Export$SampleDepth)==TRUE)]) #5 are NA as expected

#continue populating other lagos fields

data.Export$BasinType=as.character(data.Export$BasinType)

unique(data$Station.Description)

data.Export$BasinType[grep("deep",data$Station.Description,ignore.case=TRUE)]="PRIMARY"

data.Export$BasinType[which(is.na(data.Export$BasinType)==TRUE)]="UNKNOWN"

unique(data.Export$BasinType)

#check to make sure all obs. assigned a basin type

length(data.Export$BasinType[which(data.Export$BasinType=="PRIMARY")])

length(data.Export$BasinType[which(data.Export$BasinType=="UNKNOWN")])

#continue with other fields

data.Export$MethodInfo = as.character(data.Export$MethodInfo)

data.Export$MethodInfo = NA

data.Export$LabMethodName= as.character(data.Export$LabMethodName)

unique(data$Analysis.Method)

data.Export$LabMethodName= "SM_4500PF" #per emi's metadata

data.Export$LabMethodInfo=as.character(data.Export$LabMethodInfo)

data.Export$LabMethodInfo=NA

data.Export$DetectionLimit= NA #no info in meta

unique(data$Special.Notes)

unique(data.Export$SourceFlags)

data.Export$Comments=as.character(data.Export$Comments)

unique(data.Export$SourceFlags)

data.Export$Comments[which(data.Export$SourceFlags=="f")]="f=Frequency of quality control duplicates did not meet data quality objectives identified for program or in QAPP"

data.Export$Comments[which(data.Export$SourceFlags=="j")]="j='Estimated' value; can be used for lab-related issues where certain lab QC criteria are not met and re-testing is not possible (as identified by the lab)."

data.Export$Comments[which(data.Export$SourceFlags=="d")]="d=Precision of field duplicates (as RPD) did not meet project data quality objectives identified for program or in QAPP. Batched samples may also be affected."

data.Export$Comments[which(data.Export$SourceFlags=="m")]="m=Method SOP not followed, field and/or lab methodology only partially implemented or not implemented at all, due to complications with sample matrix (e.g. sediment in sample, floc formation), lab error (e.g. cross-contamination between samples), additional steps taken by the lab to deal with matrix complications, lost/unanalyzed samples, use of expired reagents, samples collected by others (non-DWM), misc. field errors, etc."

data.Export$Comments[which(data.Export$SourceFlags=="h")]="h=Holding time violation (usually indicating possible bias low)"

unique(data.Export$Comments)

drp.Final = data.Export

rm(data.Export)

rm(data)

################################### Nitrite + nitrate ################################

data=MA_DEP_2005_2010

names(data)#looking at data

#filter out columns required for this data import effort

data=data[,c(3:6,11:12,15:16,19:24)] #pulled out columns of iterest based on metadata and looking at unique value

names(data)

length(data$Analyte[which(data$Analyte=="Nitrate/Nitrite-N")]) #29 should remain after filtering

data=data[which(data$Analyte=="Nitrate/Nitrite-N"),]

unique(data$QC.type) #filter out "Duplicate"

length(data$QC.type[which(data$QC.type=="Duplicate")]) #9 are dupicates

length(data$QC.type[which(data$QC.type=="Field Blank")]) #0 are field blanks

29-9 #ony 20 obs. should remain

#filter out duplicates and field blanks

data=data[which(data$QC.type!="Duplicate"),]

data=data[which(data$QC.type!="Field Blank"),]

#looking at data to understand it

unique(data$Result.Qualifers) #no unique qualifers that suggest data needs to be filtered out

unique(data$Result) #note that their are unique characters specifying CensorCode

unique(data$Analysis.Method) #only one unique method

unique(data$Sample.Depth...meters.) # ** represents not reported

length(data$Sample.Depth...meters.[which(data$Sample.Depth...meters.=="**")]) #11 do not have a depth specified

#most but not all have a depth reported as a range

unique(data$Relative.Sample.Depth..meters.) #reveals sample position

length(data$Relative.Sample.Depth..meters.[which(data$Relative.Sample.Depth..meters.=="Near bottom")]) #10

length(data$Relative.Sample.Depth..meters.[which(data$Relative.Sample.Depth..meters.=="Surface")]) #7

length(data$Relative.Sample.Depth..meters.[which(data$Relative.Sample.Depth..meters.=="**")]) #3 are integrated thats what "--" signifies

#no other filtering

###start populating the lagos template

data.Export= LAGOS_Template

data.Export[1:nrow(data),]=NA

data.Export$LakeID = data$Unique.ID

data.Export$LakeName = data$Water.Body

data.Export$SourceVariableName = "Nitrate/Nitrite-N"

data.Export$SourceVariableDescription = "Nitrite + nitrate"

#populate SourceFlags

unique(data$Result.Qualifers) #no source flags

data.Export$SourceFlags=as.character(data.Export$SourceFlags)

data.Export$SourceFlags=NA

#check to make sure adds up to total

length(data.Export$SourceFlags[which(is.na(data.Export$SourceFlags)==TRUE)])

length(data.Export$SourceFlags[which(is.na(data.Export$SourceFlags)==FALSE)])

20+0 #adds up to totall

unique(data.Export$SourceFlags)

#continue populating other lagos variables

data.Export$LagosVariableID = 18

data.Export$LagosVariableName="Nitrogen, nitrite (NO2) + nitrate (NO3)"

#populate CensorCode

data.Export$CensorCode=as.character(data.Export$CensorCode)

unique(data$Result)

length(data$Result[which(data$Result=="<0.02")]) #12 obs are "LT"

data.Export$CensorCode[which(data$Result=="<0.02")]="LT"

data.Export$CensorCode[which(is.na(data.Export$CensorCode)==TRUE)]= "NC"

unique(data.Export$CensorCode)

#overwrite special characters

data$Result[which(data$Result=="<0.02")]=0.02

unique(data$Result) #check to make sure "<" gone

#continue with others

#note that several conversions had to be made with data type (below) in order to get the right values to export

unique(data$Units) #convert from mg/l to preff. units of ug/l

data.Export$Value=as.character(data.Export$Value)

data$Result=as.character(data$Result)

data.Export$Value = data[,11] #export nitrate

unique(data.Export$Value)

data.Export$Value=as.numeric(data.Export$Value)

data.Export$Value=(data.Export$Value)*1000 #convert to ug/l

#continue with other columns

data.Export$Date = data$Sample.Date #date already in correct format

data.Export$Units="ug/L"

#prepare to populate sampletype

data.Export$SampleType=as.character(data.Export$SampleType)

data.Export$SampleType="GRAB" #specied in metadata table & metadata

length(data.Export$SampleType[which(data.Export$SampleType=="GRAB")]) #all obs. populated

#populate sampelposition

data.Export$SamplePosition=as.character(data.Export$SamplePosition)

#first determine number of obs. of each sample position type

unique(data$Relative.Sample.Depth..meters.)

length(data$Relative.Sample.Depth..meters.[which(data$Relative.Sample.Depth..meters.=="Near bottom")])#set these as hypo = 10

length(data$Relative.Sample.Depth..meters.[which(data$Relative.Sample.Depth..meters.=="Surface")])#set these as epi=7

length(data$Relative.Sample.Depth..meters.[which(data$Relative.Sample.Depth..meters.=="**")]) #0 are integrated, set these as "Unknown" for sample position

#populate sample position

data.Export$SamplePosition[which(data$Relative.Sample.Depth..meters.=="Surface")]= "EPI"

data.Export$SamplePosition[which(data$Relative.Sample.Depth..meters.=="Near bottom")]= "HYPO"

data.Export$SamplePosition[which(data$Relative.Sample.Depth..meters.=="**")]= "UNKNOWN"

#check to make sure numbers add up

length(data.Export$SamplePosition[which(data.Export$SamplePosition=="EPI")])

length(data.Export$SamplePosition[which(data.Export$SamplePosition=="HYPO")])

length(data.Export$SamplePosition[which(data.Export$SamplePosition=="UNKNOWN")])

7+10+3 #adds up to total

#numbers add up proceed to populating sample depth

#assign sampledepth

unique(data$Sample.Depth...meters.)

length(data$Sample.Depth...meters.[which(data$Sample.Depth...meters.=="**")]) #11 are NA

#it is okay that these are NA all have a position specified

data$Sample.Depth...meters.[which(data$Sample.Depth...meters.=="**")]=NA

data.Export$SampleDepth=data$Sample.Depth...meters.

unique(data.Export$SampleDepth)

#continue populating other lagos fields

data.Export$BasinType=as.character(data.Export$BasinType)

unique(data$Station.Description)

data.Export$BasinType[grep("deep",data$Station.Description,ignore.case=TRUE)]="PRIMARY"

data.Export$BasinType[which(is.na(data.Export$BasinType)==TRUE)]="UNKNOWN"

unique(data.Export$BasinType)

#check to make sure all obs. assigned a basin type

length(data.Export$BasinType[which(data.Export$BasinType=="PRIMARY")])

length(data.Export$BasinType[which(data.Export$BasinType=="UNKNOWN")])

#continue with other fields

data.Export$MethodInfo = as.character(data.Export$MethodInfo)

data.Export$MethodInfo = NA

data.Export$LabMethodName= as.character(data.Export$LabMethodName)

data.Export$LabMethodName= "EPA_353.1" #per emi's metadata

data.Export$LabMethodInfo=as.character(data.Export$LabMethodInfo)

data.Export$LabMethodInfo=NA

data.Export$DetectionLimit= NA #no info in meta

unique(data$Special.Notes)

unique(data.Export$SourceFlags)

data.Export$Comments=as.character(data.Export$Comments)

unique(data.Export$SourceFlags)

data.Export$Comments[which(data.Export$SourceFlags=="f")]="f=Frequency of quality control duplicates did not meet data quality objectives identified for program or in QAPP"

data.Export$Comments[which(data.Export$SourceFlags=="j")]="j='Estimated' value; can be used for lab-related issues where certain lab QC criteria are not met and re-testing is not possible (as identified by the lab)."

data.Export$Comments[which(data.Export$SourceFlags=="d")]="d=Precision of field duplicates (as RPD) did not meet project data quality objectives identified for program or in QAPP. Batched samples may also be affected."

data.Export$Comments[which(data.Export$SourceFlags=="m")]="m=Method SOP not followed, field and/or lab methodology only partially implemented or not implemented at all, due to complications with sample matrix (e.g. sediment in sample, floc formation), lab error (e.g. cross-contamination between samples), additional steps taken by the lab to deal with matrix complications, lost/unanalyzed samples, use of expired reagents, samples collected by others (non-DWM), misc. field errors, etc."

data.Export$Comments[which(data.Export$SourceFlags=="h")]="h=Holding time violation (usually indicating possible bias low)"

unique(data.Export$Comments)

no3no2.Final = data.Export

rm(data.Export)

rm(data)

################################### Total nitrogen ################################

data=MA_DEP_2005_2010

names(data)#looking at data

#filter out columns required for this data import effort

data=data[,c(3:6,11:12,15:16,19:24)] #pulled out columns of iterest based on metadata and looking at unique value

names(data)

length(data$Analyte[which(data$Analyte=="Total Nitrogen")]) #178 should remain after filtering

data=data[which(data$Analyte=="Total Nitrogen"),]

unique(data$QC.type) #filter out "Duplicate"

length(data$QC.type[which(data$QC.type=="Duplicate")]) #53 are dupicates

length(data$QC.type[which(data$QC.type=="Field Blank")]) #0 are field blanks

178-53#only 125 obs. should remain

#filter out duplicates and field blanks

data=data[which(data$QC.type!="Duplicate"),]

data=data[which(data$QC.type!="Field Blank"),]

#looking at data to understand it

unique(data$Result.Qualifers) #no unique qualifers that suggest data needs to be filtered out

unique(data$Result)

length(data$Result[which(data$Result=="**")]) #filter these out, null, 3 values

data=data[which(data$Result!="**"),]

125-3 #should be left with 122

unique(data$Analysis.Method) #only one unique method

unique(data$Sample.Depth...meters.) # ** represents not reported

length(data$Sample.Depth...meters.[which(data$Sample.Depth...meters.=="**")]) #75 do not have a depth specified

#most but not all have a depth reported as a range

unique(data$Relative.Sample.Depth..meters.) #reveals sample position

length(data$Relative.Sample.Depth..meters.[which(data$Relative.Sample.Depth..meters.=="Near bottom")]) #53

length(data$Relative.Sample.Depth..meters.[which(data$Relative.Sample.Depth..meters.=="Surface")]) #63

length(data$Relative.Sample.Depth..meters.[which(data$Relative.Sample.Depth..meters.=="**")]) # 6 are null

53+63+6 #adds up to total

#no other filtering

###start populating the lagos template

data.Export= LAGOS_Template

data.Export[1:nrow(data),]=NA

data.Export$LakeID = data$Unique.ID

data.Export$LakeName = data$Water.Body

data.Export$SourceVariableName = "Total Nitrogen"

data.Export$SourceVariableDescription = "Total nitrogen"

#populate SourceFlags

unique(data$Result.Qualifers) #export to source flags

data.Export$SourceFlags=as.character(data.Export$SourceFlags)

data.Export$SourceFlags=data$Result.Qualifers

data.Export$SourceFlags[which(data.Export$SourceFlags=="")]= NA

#check to make sure adds up to total

length(data.Export$SourceFlags[which(is.na(data.Export$SourceFlags)==TRUE)])

length(data.Export$SourceFlags[which(is.na(data.Export$SourceFlags)==FALSE)])

111+11 #adds up to total

unique(data.Export$SourceFlags)

#continue populating other lagos variables

data.Export$LagosVariableID = 21

data.Export$LagosVariableName="Nitrogen, total"

#populate CensorCode

data.Export$CensorCode=as.character(data.Export$CensorCode)

unique(data$Result) #none are censored

data.Export$CensorCode= "NC"

unique(data.Export$CensorCode)

#continue with others

#note that several conversions had to be made with data type (below) in order to get the right values to export

unique(data$Units) #convert from mg/l to preff. ug/l

data.Export$Value=as.character(data.Export$Value)

data$Result=as.character(data$Result)

data.Export$Value = data[,11] #export tn obs.

unique(data.Export$Value)

data.Export$Value=as.numeric(data.Export$Value)

data.Export$Value=(data.Export$Value)*1000

#continue with other columns

data.Export$Date = data$Sample.Date #date already in correct format

data.Export$Units="ug/L"

#prepare to populate sampletype

data.Export$SampleType=as.character(data.Export$SampleType)

data.Export$SampleType="GRAB" #specied in metadata table & metadata

length(data.Export$SampleType[which(data.Export$SampleType=="GRAB")]) #all obs. populated

#populate sampelposition

data.Export$SamplePosition=as.character(data.Export$SamplePosition)

#first determine number of obs. of each sample position type

unique(data$Relative.Sample.Depth..meters.)

length(data$Relative.Sample.Depth..meters.[which(data$Relative.Sample.Depth..meters.=="Near bottom")])#set these as hypo = 8

length(data$Relative.Sample.Depth..meters.[which(data$Relative.Sample.Depth..meters.=="Surface")])#set these as epi=18

length(data$Relative.Sample.Depth..meters.[which(data$Relative.Sample.Depth..meters.== "**" )]) #6 are integrated, set these as "Unknown" for sample position

#populate sample position

data.Export$SamplePosition[which(data$Relative.Sample.Depth..meters.=="Surface")]= "EPI"

data.Export$SamplePosition[which(data$Relative.Sample.Depth..meters.=="Near bottom")]= "HYPO"

data.Export$SamplePosition[which(data$Relative.Sample.Depth..meters.=="**")]= "UNKNOWN"

data.Export$SamplePosition[which(is.na(data.Export$SamplePosition)==TRUE)]= "UNKNOWN"

#check to make sure numbers add up

length(data.Export$SamplePosition[which(data.Export$SamplePosition=="EPI")])

length(data.Export$SamplePosition[which(data.Export$SamplePosition=="HYPO")])

length(data.Export$SamplePosition[which(data.Export$SamplePosition=="UNKNOWN")])

63+53+6 #adds up to total

#numbers add up proceed to populating sample depth

#assign sampledepth

unique(data$Sample.Depth...meters.)

length(data$Sample.Depth...meters.[which(data$Sample.Depth...meters.=="**")]) #75 are NA

data$Sample.Depth...meters.[which(data$Sample.Depth...meters.=="**")]= NA

data.Export$SampleDepth=data$Sample.Depth...meters.

length(data.Export$SampleDepth[which(is.na(data.Export$SampleDepth)==TRUE)]) #75 are NA as expected

unique(data.Export$SampleDepth)

#continue populating other lagos fields

data.Export$BasinType=as.character(data.Export$BasinType)

unique(data$Station.Description)

data.Export$BasinType[grep("deep",data$Station.Description,ignore.case=TRUE)]="PRIMARY"

data.Export$BasinType[which(is.na(data.Export$BasinType)==TRUE)]="UNKNOWN"

unique(data.Export$BasinType)

#check to make sure all obs. assigned a basin type

length(data.Export$BasinType[which(data.Export$BasinType=="PRIMARY")])

length(data.Export$BasinType[which(data.Export$BasinType=="UNKNOWN")])

#continue with other fields

data.Export$MethodInfo = as.character(data.Export$MethodInfo)

data.Export$MethodInfo = NA

data.Export$LabMethodName= as.character(data.Export$LabMethodName)

data.Export$LabMethodName= "USGS_I465003" #per emi's metadata

data.Export$LabMethodInfo=as.character(data.Export$LabMethodInfo)

data.Export$LabMethodInfo=NA

data.Export$DetectionLimit= NA #no info in meta

unique(data$Special.Notes)

unique(data.Export$SourceFlags)

data.Export$Comments=as.character(data.Export$Comments)

unique(data.Export$SourceFlags)

data.Export$Comments[which(data.Export$SourceFlags=="f")]="f=Frequency of quality control duplicates did not meet data quality objectives identified for program or in QAPP"

data.Export$Comments[which(data.Export$SourceFlags=="j")]="j='Estimated' value; can be used for lab-related issues where certain lab QC criteria are not met and re-testing is not possible (as identified by the lab)."

data.Export$Comments[which(data.Export$SourceFlags=="d")]="d=Precision of field duplicates (as RPD) did not meet project data quality objectives identified for program or in QAPP. Batched samples may also be affected."

data.Export$Comments[which(data.Export$SourceFlags=="m")]="m=Method SOP not followed, field and/or lab methodology only partially implemented or not implemented at all, due to complications with sample matrix (e.g. sediment in sample, floc formation), lab error (e.g. cross-contamination between samples), additional steps taken by the lab to deal with matrix complications, lost/unanalyzed samples, use of expired reagents, samples collected by others (non-DWM), misc. field errors, etc."

data.Export$Comments[which(data.Export$SourceFlags=="h")]="h=Holding time violation (usually indicating possible bias low)"

data.Export$Comments[which(data.Export$SourceFlags=="f, p")]="f=Frequency of quality control duplicates did not meet data quality objectives identified for program or in QAPP, p=Samples not preserved per SOP or analytical method requirements"

unique(data.Export$Comments)

tn.Final = data.Export

rm(data.Export)

rm(data)

################################### Total phosphorus ################################

data=MA_DEP_2005_2010

names(data)#looking at data

#filter out columns required for this data import effort

data=data[,c(3:6,11:12,15:16,19:24)] #pulled out columns of iterest based on metadata and looking at unique value

names(data)

length(data$Analyte[which(data$Analyte=="Total Phosphorus")]) #401 should remain after filtering

data=data[which(data$Analyte=="Total Phosphorus"),]

unique(data$QC.type) #filter out "Duplicate"

length(data$QC.type[which(data$QC.type=="Duplicate")]) #119 are dupicates

length(data$QC.type[which(data$QC.type=="Field Blank")]) #26 are field blanks

401-119-26 #256 should remain after filtering

#filter out duplicates and field blanks

data=data[which(data$QC.type!="Duplicate"),]

data=data[which(data$QC.type!="Field Blank"),]

unique(data$Result) #note there are obs. that will have to be censored

length(data$Result[which(data$Result=="**")]) #filter these out, null, 6 values

data=data[which(data$Result!="**"),]

214-6 #should be left with 208

unique(data$Analysis.Method) #two unique analytical methods

unique(data$Sample.Depth...meters.) # ** represents not reported

length(data$Sample.Depth...meters.[which(data$Sample.Depth...meters.=="**")]) #107 do not have a depth specified

#most but not all have a depth reported as a range

unique(data$Relative.Sample.Depth..meters.) #reveals sample position

length(data$Relative.Sample.Depth..meters.[which(data$Relative.Sample.Depth..meters.=="Near bottom")]) #135

length(data$Relative.Sample.Depth..meters.[which(data$Relative.Sample.Depth..meters.=="Surface")]) #106

length(data$Relative.Sample.Depth..meters.[which(data$Relative.Sample.Depth..meters.=="**")]) #8 are null

length(data$Relative.Sample.Depth..meters.[which(data$Relative.Sample.Depth..meters.==" --")])

135+106+8+1#adds up to total

#no other filtering

###start populating the lagos template

data.Export= LAGOS_Template

data.Export[1:nrow(data),]=NA

data.Export$LakeID = data$Unique.ID

data.Export$LakeName = data$Water.Body

data.Export$SourceVariableName = "Total Phosphorus"

data.Export$SourceVariableDescription = "Total phosphorus"

#populate SourceFlags

unique(data$Result.Qualifers) #export to source flags

data.Export$SourceFlags=as.character(data.Export$SourceFlags)

data.Export$SourceFlags=data$Result.Qualifers

data.Export$SourceFlags[which(data.Export$SourceFlags=="")]= NA

#check to make sure adds up to total

length(data.Export$SourceFlags[which(is.na(data.Export$SourceFlags)==TRUE)])

length(data.Export$SourceFlags[which(is.na(data.Export$SourceFlags)==FALSE)])

166+84 #adds up to total

unique(data.Export$SourceFlags)

#continue populating other lagos variables

data.Export$LagosVariableID = 27

data.Export$LagosVariableName="Phosphorus, total"

#populate CensorCode

data.Export$CensorCode=as.character(data.Export$CensorCode)

unique(data$Result) #deal with censored obs.

length(data$Result[which(data$Result=="<0.005")])

data.Export$CensorCode[which(data$Result=="<0.005")]="LT"

data.Export$CensorCode[which(is.na(data.Export$CensorCode)==TRUE)]="NC"

unique(data.Export$CensorCode)

#overwrite special character signs

data$Result[which(data$Result=="<0.005")]=0.005

unique(data$Result)

#continue with others

#note that several conversions had to be made with data type (below) in order to get the right values to export

unique(data$Units) #convert from mg/l to preff. ug/l

data.Export$Value=as.character(data.Export$Value)

data$Result=as.character(data$Result)

data.Export$Value = data[,11] #export tp obs.

unique(data.Export$Value)

data.Export$Value=as.numeric(data.Export$Value)

data.Export$Value=(data.Export$Value)*1000

#continue with other columns

data.Export$Date = data$Sample.Date #date already in correct format

data.Export$Units="ug/L"

#prepare to populate sampletype

data.Export$SampleType=as.character(data.Export$SampleType)

data.Export$SampleType="GRAB" #specied in metadata table & metadata

length(data.Export$SampleType[which(data.Export$SampleType=="GRAB")]) #all obs. populated

#populate sampelposition

data.Export$SamplePosition=as.character(data.Export$SamplePosition)

#first determine number of obs. of each sample position type

unique(data$Relative.Sample.Depth..meters.)

length(data$Relative.Sample.Depth..meters.[which(data$Relative.Sample.Depth..meters.=="Near bottom")])#set these as hypo = 107

length(data$Relative.Sample.Depth..meters.[which(data$Relative.Sample.Depth..meters.=="Surface")])#set these as epi=93

length(data$Relative.Sample.Depth..meters.[which(data$Relative.Sample.Depth..meters.=="--")]) #0 are integrated, set these as "Unknown" for sample position

#populate sample position

data.Export$SamplePosition[which(data$Relative.Sample.Depth..meters.=="Surface")]= "EPI"

data.Export$SamplePosition[which(data$Relative.Sample.Depth..meters.=="Near bottom")]= "HYPO"

data.Export$SamplePosition[which(data$Relative.Sample.Depth..meters.==" --")]= "UNKNOWN"

data.Export$SamplePosition[which(is.na(data.Export$SamplePosition)==TRUE)]= "UNKNOWN"

#check to make sure numbers add up

length(data.Export$SamplePosition[which(data.Export$SamplePosition=="EPI")])

length(data.Export$SamplePosition[which(data.Export$SamplePosition=="HYPO")])

length(data.Export$SamplePosition[which(data.Export$SamplePosition=="UNKNOWN")])

106+135+9 #adds up to total

#numbers add up proceed to populating sample depth

#assign sampledepth

unique(data$Sample.Depth...meters.)

length(data$Sample.Depth...meters.[which(data$Sample.Depth...meters.=="**")]) #107 are NA

length(data$Sample.Depth...meters.[which(data$Sample.Depth...meters.==" --")]) #another NA

data$Sample.Depth...meters.[which(data$Sample.Depth...meters.=="**")]= NA

data$Sample.Depth...meters.[which(data$Sample.Depth...meters.==" --")]= NA

data.Export$SampleDepth=data$Sample.Depth...meters.

length(data.Export$SampleDepth[which(is.na(data.Export$SampleDepth)==TRUE)]) #108 are NA as expected

unique(data.Export$SampleDepth)

#continue populating other lagos fields

data.Export$BasinType=as.character(data.Export$BasinType)

unique(data$Station.Description)

data.Export$BasinType[grep("deep",data$Station.Description,ignore.case=TRUE)]="PRIMARY"

data.Export$BasinType[which(is.na(data.Export$BasinType)==TRUE)]="UNKNOWN"

unique(data.Export$BasinType)

#check to make sure all obs. assigned a basin type

length(data.Export$BasinType[which(data.Export$BasinType=="PRIMARY")])

length(data.Export$BasinType[which(data.Export$BasinType=="UNKNOWN")])

#continue with other fields

data.Export$MethodInfo = as.character(data.Export$MethodInfo)

data.Export$MethodInfo = NA

data.Export$LabMethodName= as.character(data.Export$LabMethodName)

unique(data$Analysis.Method)

data.Export$LabMethodName[which(data$Analysis.Method=="SM 4500-P E")]="SM_4500PE"

data.Export$LabMethodName[which(data$Analysis.Method=="USGS I-4650-03")]="USGS_I465003"

length(data.Export$LabMethodName[which(is.na(data.Export$LabMethodName)==TRUE)]) #check to make sure none are null

data.Export$LabMethodInfo=as.character(data.Export$LabMethodInfo)

data.Export$LabMethodInfo=NA

data.Export$DetectionLimit= NA #no info in meta

unique(data$Special.Notes)

unique(data.Export$SourceFlags)

data.Export$Comments=as.character(data.Export$Comments)

unique(data.Export$SourceFlags)

data.Export$Comments[which(data.Export$SourceFlags=="f")]="f=Frequency of quality control duplicates did not meet data quality objectives identified for program or in QAPP"

data.Export$Comments[which(data.Export$SourceFlags=="j")]="j='Estimated' value; can be used for lab-related issues where certain lab QC criteria are not met and re-testing is not possible (as identified by the lab)."

data.Export$Comments[which(data.Export$SourceFlags=="d")]="d=Precision of field duplicates (as RPD) did not meet project data quality objectives identified for program or in QAPP. Batched samples may also be affected."

data.Export$Comments[which(data.Export$SourceFlags=="m")]="m=Method SOP not followed, field and/or lab methodology only partially implemented or not implemented at all, due to complications with sample matrix (e.g. sediment in sample, floc formation), lab error (e.g. cross-contamination between samples), additional steps taken by the lab to deal with matrix complications, lost/unanalyzed samples, use of expired reagents, samples collected by others (non-DWM), misc. field errors, etc."

data.Export$Comments[which(data.Export$SourceFlags=="h")]="h=Holding time violation (usually indicating possible bias low)"

unique(data.Export$Comments)

tp.Final = data.Export

rm(data.Export)

rm(data)

################################### True color ################################

data=MA_DEP_2005_2010

names(data)#looking at data

#filter out columns required for this data import effort

data=data[,c(3:6,11:12,15:16,19:24)] #pulled out columns of iterest based on metadata and looking at unique value

names(data)

length(data$Analyte[which(data$Analyte=="True color")]) #18 should remain after filtering

data=data[which(data$Analyte=="True color"),]

unique(data$QC.type) #filter out "Duplicate"

length(data$QC.type[which(data$QC.type=="Duplicate")]) #8 are dupicates

length(data$QC.type[which(data$QC.type=="Field Blank")]) #0 are field blanks

18-8 #10 should remain after filtering

#filter out duplicates and field blanks

data=data[which(data$QC.type!="Duplicate"),]

data=data[which(data$QC.type!="Field Blank"),]

#looking at data to understand it

unique(data$Result.Qualifers) #unique qualifers that suggest data needs to be filtered out per meta

length(data$Result.Qualifers[which(data$Result.Qualifers==" --")]) #0need to be filtered out

unique(data$Result) #note there are obs. that will have to be censored

unique(data$Analysis.Method) #one analytical method

unique(data$Sample.Depth...meters.) # ** represents not reported

length(data$Sample.Depth...meters.[which(data$Sample.Depth...meters.=="**")]) #all obs. null for depth

#most but not all have a depth reported as a range

unique(data$Relative.Sample.Depth..meters.) #reveals sample position

length(data$Relative.Sample.Depth..meters.[which(data$Relative.Sample.Depth..meters.=="Near bottom")]) #0

length(data$Relative.Sample.Depth..meters.[which(data$Relative.Sample.Depth..meters.=="Surface")]) #10

length(data$Relative.Sample.Depth..meters.[which(data$Relative.Sample.Depth..meters.=="**")]) #0are null

#no other filtering

###start populating the lagos template

data.Export= LAGOS_Template

data.Export[1:nrow(data),]=NA

data.Export$LakeID = data$Unique.ID

data.Export$LakeName = data$Water.Body

data.Export$SourceVariableName = "True Color"

data.Export$SourceVariableDescription = "True color"

#populate SourceFlags

unique(data$Result.Qualifers) #none

data.Export$SourceFlags=as.character(data.Export$SourceFlags)

data.Export$SourceFlags=NA

#check to make sure adds up to total

length(data.Export$SourceFlags[which(is.na(data.Export$SourceFlags)==TRUE)])

length(data.Export$SourceFlags[which(is.na(data.Export$SourceFlags)==FALSE)])

10+0 #adds up to total

unique(data.Export$SourceFlags)

#continue populating other lagos variables

data.Export$LagosVariableID = 12

data.Export$LagosVariableName="Color, true"

#populate CensorCode

data.Export$CensorCode=as.character(data.Export$CensorCode)

unique(data$Result) #deal with censored obs.

length(data$Result[which(data$Result=="<15")])

data.Export$CensorCode[which(data$Result=="<15")]="LT"

data.Export$CensorCode[which(is.na(data.Export$CensorCode)==TRUE)]="NC"

unique(data.Export$CensorCode)

#overwrite special character signs

data$Result[which(data$Result=="<15")]=15

unique(data$Result)

#continue with others

unique(data$Units) #pcu

typeof(data.Export$Value)

data$Result=as.character(data$Result)

data.Export$Value=as.character(data.Export$Value)

data.Export$Value= data[,11] #export obs.alreadyi n PCU = preff. units

typeof(data.Export$Value)

unique(data.Export$Value)

data.Export$Value=as.numeric(data.Export$Value)

unique(data.Export$Value)

#continue with other columns

data.Export$Date = data$Sample.Date #date already in correct format

data.Export$Units="PCU"

#prepare to populate sampletype

data.Export$SampleType=as.character(data.Export$SampleType)

data.Export$SampleType="GRAB" #specied in metadata table & metadata

length(data.Export$SampleType[which(data.Export$SampleType=="GRAB")]) #all obs. populated

#populate sampelposition

data.Export$SamplePosition=as.character(data.Export$SamplePosition)

#first determine number of obs. of each sample position type

unique(data$Relative.Sample.Depth..meters.)

length(data$Relative.Sample.Depth..meters.[which(data$Relative.Sample.Depth..meters.=="Near bottom")])#set these as hypo = 0

length(data$Relative.Sample.Depth..meters.[which(data$Relative.Sample.Depth..meters.=="Surface")])#set these as epi=10

length(data$Relative.Sample.Depth..meters.[which(data$Relative.Sample.Depth..meters.=="--")]) #0 are integrated, set these as "Unknown" for sample position

#populate sample position

data.Export$SamplePosition[which(data$Relative.Sample.Depth..meters.=="Surface")]= "EPI"

data.Export$SamplePosition[which(data$Relative.Sample.Depth..meters.=="Near bottom")]= "HYPO"

data.Export$SamplePosition[which(data$Relative.Sample.Depth..meters.=="--")]= "UNKNOWN"

data.Export$SamplePosition[which(is.na(data.Export$SamplePosition)==TRUE)]= "UNKNOWN"

#check to make sure numbers add up

length(data.Export$SamplePosition[which(data.Export$SamplePosition=="EPI")])

length(data.Export$SamplePosition[which(data.Export$SamplePosition=="HYPO")])

length(data.Export$SamplePosition[which(data.Export$SamplePosition=="UNKNOWN")])

10+0+0#adds up to total

#numbers add up proceed to populating sample depth

#assign sampledepth

unique(data$Sample.Depth...meters.) #ALL NA

data.Export$SampleDepth=NA

#continue populating other lagos fields

data.Export$BasinType=as.character(data.Export$BasinType)

unique(data$Station.Description)

data.Export$BasinType[grep("deep",data$Station.Description,ignore.case=TRUE)]="PRIMARY"

data.Export$BasinType[which(is.na(data.Export$BasinType)==TRUE)]="UNKNOWN"

unique(data.Export$BasinType)

#check to make sure all obs. assigned a basin type

length(data.Export$BasinType[which(data.Export$BasinType=="PRIMARY")])

length(data.Export$BasinType[which(data.Export$BasinType=="UNKNOWN")])

#continue with other fields

data.Export$MethodInfo = as.character(data.Export$MethodInfo)

data.Export$MethodInfo = NA

data.Export$LabMethodName= as.character(data.Export$LabMethodName)

unique(data$Analysis.Method)

data.Export$LabMethodName="SM_2120B"

data.Export$LabMethodInfo=as.character(data.Export$LabMethodInfo)

data.Export$LabMethodInfo=NA

data.Export$DetectionLimit= NA #no info in meta

unique(data$Special.Notes)

data.Export$Comments=as.character(data.Export$Comments)

unique(data.Export$SourceFlags)

data.Export$Comments= NA

tcolor.Final = data.Export

rm(data.Export)

rm(data)

################################### Secchi Depth (meters) ################################

data=MA_DEP_2005_2010

names(data)#looking at data

#filter out columns required for this data import effort

data=data[,c(3:6,11:12,15:18,20,22:24)] #pulled out columns of iterest based on metadata and looking at unique value

names(data)

unique(data$Secchi.Depth...meters.)

data=data[which(is.na(data$Secchi.Depth...meters.)==FALSE),] #none are NA anyway

length(data$Secchi.Depth...meters.[which(data$Secchi.Depth...meters.=="**")]) #89

length(data$Secchi.Depth...meters.[which(data$Secchi.Depth...meters.==" --")])#1

length(data$Secchi.Depth...meters.[which(data$Secchi.Depth...meters.=="** ")])#13

length(data$Secchi.Depth...meters.[which(data$Secchi.Depth...meters.=="")])#13

#filter out those obs

data=data[which(data$Secchi.Depth...meters.!="**"),]

data=data[which(data$Secchi.Depth...meters.!=" --"),]

data=data[which(data$Secchi.Depth...meters.!="** "),]

data=data[which(data$Secchi.Depth...meters.!=""),]

1422-90-13-15 #1304 should remain

#looking at data to understand it

unique(data$Secchi.Depth.Qualifiers) #note that a is where the disk hit the bottom, e means value not possible

#no other filtering

###start populating the lagos template

data.Export= LAGOS_Template

data.Export[1:nrow(data),]=NA

data.Export$LakeID = data$Unique.ID

data.Export$LakeName = data$Water.Body

data.Export$SourceVariableName = "Secchi Depth (meters)"

data.Export$SourceVariableDescription = "Secchi"

#populate SourceFlags

unique(data$Result.Qualifers) #ignore these

unique(data$Secchi.Depth.Qualifiers)

data.Export$SourceFlags=as.character(data.Export$SourceFlags)

data.Export$SourceFlags=data$Secchi.Depth.Qualifiers

data.Export$SourceFlags[which(data.Export$SourceFlags=="")]= NA

unique(data.Export$SourceFlags)

#check to make sure adds up to total

length(data.Export$SourceFlags[which(is.na(data.Export$SourceFlags)==TRUE)])

length(data.Export$SourceFlags[which(is.na(data.Export$SourceFlags)==FALSE)])

1246+58 #adds up to total

unique(data.Export$SourceFlags)

#continue populating other lagos variables

data.Export$LagosVariableID = 30

data.Export$LagosVariableName="Secchi"

#populate CensorCode

data.Export$CensorCode=as.character(data.Export$CensorCode)

unique(data$Secchi.Depth.Qualifiers)

length(data$Secchi.Depth.Qualifiers[which(data$Secchi.Depth.Qualifiers=="e")]) #6 value not possible

length(data$Secchi.Depth.Qualifiers[which(data$Secchi.Depth.Qualifiers=="a")]) #47 disk hit bottom

length(data$Secchi.Depth.Qualifiers[which(data$Secchi.Depth.Qualifiers=="a, e")]) #5 both

data.Export$CensorCode[which(data$Secchi.Depth.Qualifiers=="a")]="GT"

data.Export$CensorCode[which(data$Secchi.Depth.Qualifiers=="a, e")]="GT"

data.Export$CensorCode[which(is.na(data.Export$CensorCode)==TRUE)]="NC"

unique(data.Export$CensorCode)

#continue with others

names(data)

typeof(data.Export$Value)

data$Secchi.Depth...meters.=as.character(data$Secchi.Depth...meters.)

data.Export$Value=as.character(data.Export$Value)

names(data)

data.Export$Value= data[,9] #export obs.alreadyi n PCU = preff. units

typeof(data.Export$Value)

unique(data.Export$Value)

data.Export$Value=as.numeric(data.Export$Value)

unique(data.Export$Value)

length(data.Export$Value[which(data.Export$Value=="")])

#continue with other columns

data.Export$Date = data$Sample.Date #date already in correct format

data.Export$Units="m"

#prepare to populate sampletype

data.Export$SampleType=as.character(data.Export$SampleType)

data.Export$SampleType="INTEGRATED" #specied in metadata table & metadata

length(data.Export$SampleType[which(data.Export$SampleType=="INTEGRATED")]) #all obs. populated

#populate sampelposition

data.Export$SamplePosition=as.character(data.Export$SamplePosition)

data.Export$SamplePosition="SPECIFIED"

unique(data.Export$SamplePosition)

#numbers add up proceed to populating sample depth

#assign sampledepth

data.Export$SampleDepth=NA

#continue populating other lagos fields

data.Export$BasinType=as.character(data.Export$BasinType)

unique(data$Station.Description)

data.Export$BasinType[grep("deep",data$Station.Description,ignore.case=TRUE)]="PRIMARY"

data.Export$BasinType[which(is.na(data.Export$BasinType)==TRUE)]="UNKNOWN"

unique(data.Export$BasinType)

#check to make sure all obs. assigned a basin type

length(data.Export$BasinType[which(data.Export$BasinType=="PRIMARY")])

length(data.Export$BasinType[which(data.Export$BasinType=="UNKNOWN")])

#continue with other fields

data.Export$MethodInfo = as.character(data.Export$MethodInfo)

data.Export$MethodInfo = "SECCHI_VIEW_UNKNOWN"

data.Export$LabMethodName= as.character(data.Export$LabMethodName)

data.Export$LabMethodName=NA

data.Export$LabMethodInfo=as.character(data.Export$LabMethodInfo)

data.Export$LabMethodInfo=NA

data.Export$DetectionLimit= NA #no info in meta

unique(data$Special.Notes)

unique(data.Export$SourceFlags)

data.Export$Comments=as.character(data.Export$Comments)

data.Export$Comments[which(data.Export$SourceFlags=="e")]="e=value not possible"

data.Export$Comments[which(data.Export$SourceFlags=="a")]="a=disk hit bottom"

data.Export$Comments[which(data.Export$SourceFlags=="a, e")]="a=disk hit bottom, e=value not possible"

unique(data.Export$Comments)

secchi.Final = data.Export

rm(data.Export)

rm(data)

########################################### final export #######################################

Final.Export = rbind(acolor.Final,chla.Final,drp.Final,nh4.Final,no3no2.Final, secchi.Final,tcolor.Final,tn.Final,tp.Final)

###########################################################################################

##Duplicates check #################################

#an observation is defined as duplicate if it is NOT unique for programid, lagoslakeid, date, sampledepth, sampleposition, lagosvariableid, datavalue

names(Final.Export)

library(data.table)

data1=data.table(Final.Export,key=c('LakeID','Value','Date','LagosVariableID','SampleDepth','SamplePosition'))

data1=data1[,Dup:=duplicated(.SD),.SDcols=c('LakeID','Value','Date', 'LagosVariableID', 'SampleDepth', 'SamplePosition')]

head(data1)#look at a snapshot of the data

data1$Dup[which(data1$Dup==FALSE)]=NA

data1$Dup[which(data1$Dup==TRUE)]=1

unique(data1$Dup)

#check to see if they add up to the total

length(data1$Dup[which(data1$Dup=="1")])

length(data1$Dup[which(is.na(data1$Dup)==TRUE)])

1119+683#adds up to total

##write table

Final.Export1=data1

typeof(Final.Export1$Value)

length(Final.Export1$Value[which(Final.Export1$Value<0)])

nosamplepos=Final.Export1[which(is.na(Final.Export1$SampleDepth)==TRUE & Final.Export1$SamplePosition=="UNKNOWN"),]

write.table(Final.Export1,file="DataImport_MA_DEP_2005_2010.csv",row.names=FALSE,sep=",")

save.image("C:/Users/schristel/Dropbox/CSI-LIMNO_DATA/DATA-lake/MA data/MassDEPData-2005-2010(Pat Done)/DataImport_MA_DEP_2005_2010/DataImport_MA_DEP_2005_2010.RData")
