# Supplementary material for: Building a multi-scaled geospatial temporal ecology database from disparate data sources: fostering open science and data reuse
Source: Gigascience. 2015 Jul 1;4:28. doi: 10.1186/s13742-015-0067-4 (PMC4488039; doi:10.1186/s13742-015-0067-4)
Supplement: Additional file 20: — QAQC protocol for LAGOSLIMNO. The protocols that we used to QAQC the data after all of it had been loaded into and then exported from LAGOSLIMNO. [file 13742_2015_67_MOESM20_ESM.docx]

Additional file 20

**QAQC protocol for LAGOS_LIMNO_**

Noah Lottig, Patricia Soranno

**Overview**

There are two major phases in the quality assurance/quality control (QAQC) procedure for LAGOS_LIMNO_. For the first major version of LAGOS_LIMNO_ database (Ver 1.040.0), both QAQC phases were conducted. For all subsequent major versions of LAGOS_LIMNO_ (in which at least 8 new datasets are loaded), all data, including data from prior versions of LAGOS_LIMNO_, will be assessed again using both phases of the QAQC procedure. Because many of the QAQC analyses outlined here make use of all information from an individual lake or variable, incorporating new data may result in a better assessment of the data than was feasible before the addition of new data. Thus, existing flags will be discarded and new flags will be generated for each subsequent major version of LAGOS_LIMNO_.

**Phase I – Program-level analysis: All limnological data in LAGOS_LIMNO_ to detect systematic import and program-level errors**

In Phase I, we analyze limnological data at the level of the individual program and variable. For each program, we examine individual variables, and, when possible, combinations of two variables between which we expect a relationship based on known limnological relationships. In this phase, we seek to identify problems or errors at the program level or at various steps in the data import. When problems are identified, they are fixed at the central LAGOS_LIMNO_ database level and the data are re-exported before proceeding to the next QAQC phase. In general, this phase does not result in assigning flags to data; rather, it is used as a diagnostic step to identify major problems in data import/export.

The steps are as follows:

1. Import data: Read in all LAGOS_LIMNO_ data (for all lakes and lake depths)
   1. We exclude data that are flagged by the original data source (in the censorcode field) as being greater than or less than a reported value, and remove them from the QAQC process. This is done because we do not know what the true value is, only that it is greater than or less than some reported number.
2. Variable diagnostics: For each Program/Variable combination, generate general diagnostic plots of the data that include histograms, boxplots, the number of observations, and the variance of those observations. Because ecological data are often not normally distributed and characterized by fat tails, we use robust boxplots that account for the skewness of the data [1]. Histograms and boxplots provide similar types of information in that they are both representations of the distribution of the data, but provide slightly different representations of those distributions.
   1. *Distribution plots (shape)*: Examine the histogram shapes for each program and for each variable. For each variable, we compare across programs by visually assessing whether the distribution diverges from the general patterns observed in other programs. Specifically, we target bimodal distributions, extremely skewed data, or other patterns that diverge drastically from the other programs. In general, these divergent patterns can indicate that variables were recorded in more than one measurement unit (e.g., feet and meters; or mg/L and µg/L). See Figure S25 for an example of Secchi depth data that contained values reported in both feet and meters, even though the metadata suggested the data was all reported in meters.
   2. *Distribution plots (mode and range)*: Compare mode and range of distribution plots (both histograms and robust boxplots) to determine whether program level unit conversion issues exist. For example, units of a variable were thought to be mg/L and converted to µg/L when in fact the units were originally in µg/L. Typically, this results in distributions being shifted by approximately three orders of magnitude for variables measured as concentrations. See Figure S26 for an example of datasets that demonstrate order-of-magnitude decreases and increases in the distribution modes and ranges relative to the other programs loaded into LAGOS owing to incorrect unit conversions.
   3. *Variance*: Very low variance in the data is suggestive of constant values (e.g., all values the same, such as 10.4) and may indicate a data import problem. This occurred twice in the QAQC process for two separate variables in different programs. In both cases, the reported values were below the detection limit because at a program level, no measurement was ever made that was above the detection limit. This observation was one of the driving factors for Phase I-1a.
3. Limnological relationships: Many ecological variables tend to change in predictable ways, such as the positive relationship between chlorophyll a (Chla) and total phosphorus (TP). At the program level, we generate all combinations of bivariate plots (e.g., TP vs Chla plots) for the variables included in LAGOS_LIMNO_. For each bivariate plot, we use quantile regression to estimate the slope of the relationship between the two variables. Slopes from the bivariate plots are examined to assess whether a program does not follow known ecological relationships or varies substantially relative to the other programs. See Figure S27 for an example of two bivariate plots that do not follow known ecological relationships. Relationship errors typically occurred as a result of errors in the source data or in the data importing stage.
4. Strategy to deal with data problems: If problems are identified in steps 2-3, then the data managers examine the data beginning with the original source raw data, then the data-import step, and finally the export step to identify the possible problem(s). The data manager fixes the problem and re-exports a new version of LAGOS_LIMNO_ for the remaining QAQC steps.
5. We repeat steps 1-3 to ensure the problems identified are fixed.

**Phase II – Analysis of selected data in LAGOS_LIMNO_**

Phase II of the QAQC procedure identifies anomalies in the data at the variable level, in contrast to Phase I, which identified program level issues. Here we take a variety of approaches, including: combining information from the LAGOS_LIMNO_ data, professional knowledge, and published literature to determine realistic ranges in values for parameters; comparing different forms of parameters such as total and dissolved phosphorus to determine if values are inconsistent with known relationships (e.g., dissolved cannot be greater than total fractions); identifying observations using multiple different parameters that are inconsistent with limnological relationships (e.g., Chla/TP relationship); identifying observations within a lake that are inconsistent with the historical record in the lake. A significant amount of effort was placed in generating robust approaches that automatically identify and flag outliers so as to remove observer bias and ensure that the process was repeatable regardless of who was processing the data and across different sampling programs.

**A. Import and clean up data for detailed QAQC analysis**

1. Read in all LAGOS_LIMNO_ data (all lakes and sample depths) after they have passed Phase I of the QAQC process.
   1. Exclude values censored by the source program for Phase II QAQC process (see Phase I-1a)
   2. Exclude observations with reported zero values for Phase II QAQC process (except Secchi). Zero values that are reported likely represent below detection limit (BDL) values (very few analytical methods can actually measure a true zero value).

**B. Egregious Threshold (ET) and Maximum Allowable Value (MAV)**

1. Delete all values that exceed the Egregious Threshold values and flag all values that exceed Maximum Allowable Value as 'MAV' (Table S36).

2. Generate table of flagged values for the database administrator to integrate into the lagosflag field of LAGOS database.

3. Exclude 'MAV' flagged values from current QAQC dataset. Data values are no longer part of the QAQC process for remaining Phase II steps.

**C. Analysis of bounded relationships for select variables**

The bounded relationship (RATIO) QAQC step assumes that species of nutrients cannot be greater than the TOTAL measures of those nutrients (TP and TN).

1. Calculate nitrogen and phosphorus ratios such as total dissolved phosphorus (TDP)/TP, total Kjeldahl nitrogen (TKN)/total nitrogen (TN) when known quantities are bounded by another known parameter (e.g., TDP can never be greater than TP). See Table S37 for a list of all calculated ratios.
2. Flag values that have ratios >1.1 as 'RATIO'.
3. Generate table of flagged values for the database administrator to integrate into the lagosflag field of LAGOS_LIMNO_ database.
4. Exclude 'RATIO' flagged values from current QAQC dataset. Data values are no longer part of the QAQC process for remaining Phase II steps.

**D. Distance-based outlier test**

The distance-based outlier (BIPLOT) QAQC step assumes that every variable is related in some systematic way to the other variables for all of LAGOS_LIMNO_ data.

1. Generate bi-plot matrixes of all combinations of variables in LAGOS_LIMNO_ using data from all programs at any depth.
2. Calculate the Euclidian distances between all bi-plot points when more than 20 points exist and extract the Euclidian distance to the 5th nearest point (k value). K values between 3 and 20 were compared and we determined that k = 5 appears to provide an optimal balance of identifying points that appeared to be outliers in bi-plot space while retaining points that are not egregious.
3. Calculate boxplots of the Euclidian distances and identify outlier biplot points that have Euclidian distances greater than 15 times the IQR of the boxplot. These points were then given a flag value of ‘BIPLOT’ for both variables (i.e., the X and the Y variable from the original biplot). We determined that 15 times the IQR was a conservative estimate in retaining data. It excludes only those values whose Euclidian distances are extreme relative to all other values in a given bi-plot space.
4. Generate table of flagged values for the database administrator to integrate into the lagosflag field of LAGOS_LIMNO_ database.
5. Exclude 'BIPLOT' flagged values from current QAQC dataset. Data values are no longer part of the QAQC process for remaining Phase II steps.

**E. Analysis of variables by depth through time (time-test)**

The time-test (TIME) QAQC step assumes that lake-level variables are temporally related to one another from year to year.

1. Split the dataset into two separate datasets. A near surface (EPI) dataset is composed of 1) all samples designated as 'EPI', and 2) samples from depth < 10 m when the stratum is not designated but depth is, and 3) any sample with unknown sample depth or stratum (based on the assumption that if a sampling program collects samples without depth specified that it is likely taken from the surface). The second dataset is a hypolimnion (HYPO) dataset and contains all data that are identified as 'HYPO' samples, and, when the stratum is not identified but the depth is, those samples that have a depth of ≥ 10m. This step is conducted only for this analysis and is driven by the fact that hypolimnion TP concentrations may, for example, be very different from epilimnion concentrations during stratified periods. Consequently, within a lake, the distributions of data might be very different and thus should be considered separately when identifying outliers using discrete time-series of data without other co-varying information.
2. Identify lakes that have had an individual variable measured on at least five different dates over the entire sampling record for that specific lake in LAGOS_LIMNO_. Thus, each lake must have at least five observations for a given variable (but the number of observations could be greater if multiple depths within each stratum were measured on the same date).
   1. For each identified lake, analyze variables for outliers using the EPI and HYPO datasets separately. We exclude data from lakes with time series data that have median absolute deviations equal to zero due to analytical difficulties (e.g., TP values of 1.2, 1.2, 1.2, 1.2, 0.5, 1.2, 1.2, 1.5) would result in both 0.5 and 1.5 being identified as outliers).
   2. Outliers were identified when the generalized extreme studentized deviate test indicated an outlier at p = 0.001 and the outlier is greater than eight times the IQR value using robust boxplots.
   3. Values are flagged as 'TIME'.
3. Generate table of flagged values for the database administrator to integrate into the lagosflag field of LAGOS_LIMNO_ database.
4. Exclude 'TIME' flagged values from current QAQC dataset. Data values are no longer part of the QAQC process for remaining Phase II steps.

**Integrating QAQC results into LAGOS_LIMNO_ database**

The QAQC team integrates with the database administrator at two stages during the QAQC process:

Phase I: The QAQC is tightly coupled with the database administrator. Potential program-level issues are communicated in near real-time with the database administrator so that those issues can be investigated by the appropriate individuals in order for the QAQC process to proceed. Each time the database administrator resolves a potential issue, a new export is generated for the QAQC process, after which Phase I starts over until all issues are resolved and the QAQC team concludes that no systematic program-level errors remain.

Phase II: The results of the QAQC steps are managed by the QAQC team, with the final result being a table of flagged values (defined in Table S38) that is provided to the database administrator. Table S39 show the number of flagged values in LAGOS_LIMNO_ Ver 1.040The database administrator then imports the table into LAGOS_LIMNO_ and generates a fully QAQC’d dataset for research users. Starting with Phase II, the QAQC process proceeds sequentially as outlined above. At each step, when observations are identified and flagged, those observations are integrated into a separate flagged value table (for eventual export to the database) and the values are removed from the QAQC dataset. For example, the first set of values that are removed from the QAQC dataset are censored values (Phase II-A1a). Following this step these censored and flagged values identified in Phase II are no longer included in the QAQC dataset for all future QAQC steps.

After Phase II is completed, the resulting data table of flagged values is sent to the database administrator to import into the LAGOS_LIMNO_ database to populate the LAGOS-QAQC flag field.

**References**

1. Vanderviere E, Huber M. An adjusted boxplot for skewed distributions. COMPSTAT’2004 Symposium. 2004. https://wis.kuleuven.be/stat/robust/papers/2004/boxplotCOMPSTAT04.pdf. Accessed 27 May 2015.

**Table S36. Maximum allowable values (MAVs) and egregious thresholds (ETs) that are used in the QAQC process**

| **Variable** | **Units** | **Maximum Allowable Value** | **Egregious Thresholds** |
| --- | --- | --- | --- |
| Chla | µg/L | 800 | 1,600 |
| Colora | PCU | 600 | 1,200 |
| Colort | PCU | 600 | 1,200 |
| DKN | µg/L | 21,000 | 40,000 |
| DOC | mg/L | 100 | 165 |
| NH4 | µg/L | 21,000 | 40,000 |
| NO2 | µg/L | 21,000 | 40,000 |
| NO2NO3 | µg/L | 21,000 | 40,000 |
| Secchi | m | 32 | 32 |
| SRP | µg/L | 2,000 | 10,000 |
| TDN | µg/L | 21,000 | 40,000 |
| TDP | µg/L | 2,000 | 10,000 |
| TKN | µg/L | 21,000 | 40,000 |
| TN | µg/L | 21,000 | 40,000 |
| TOC | mg/L | 332 | 332 |
| TON | µg/L | 21,000 | 40,000 |
| TP | µg/L | 2,000 | 10,000 |

Egregious threshold values represent the maximum limit for a variable value that will remain in LAGOS_LIMNO_. Values above ET are permanently deleted from the database. Values between MAV and ETs represent values that are highly unlikely for the systems included in this study but are retained with flags in the dataset for users who might be interested in examining extremes. Limnological variables are defined in the Glossary (Additional file 1).

**Table S37. Bounded relationship ratios examined in the LAGOS_LIMNO_ QAQC process**

| **Major Nutrient** | |
| --- | --- |
| **Phosphorus** | **Nitrogen** |
| TDP:TP | TON:TN |
| SRP:TP | TKN:TN |
| SRP:TDP | TDN:TN |
|  | NO2NO3:TN |
|  | NO2:TN |
|  | NH4:TN |
|  | DKN:TN |
|  | NO2:NO2NO3 |
|  | DKN:TKN |
|  | NH4:TKN |
|  | NH4:DKN |
|  | TON:TKN |
|  | (NO2NO3+NH4):TKN |
|  | (NO2NO3+NH4):TDN |
|  | (TN-TKN):NO2NO3 |

These terms represent different forms of phosphorus and nitrogen referred to in the Glossary (Additional file 1).

**Table S38. LAGOS_LIMNO_ QAQC flag descriptions**

| **QAQC Flag CODE** | **Description** | **Limnological data type relevant to QAQC flag** | **LAGOS_LIMNO_ variables relevant to QAQC flag** |
| --- | --- | --- | --- |
| MAV | Maximum allowable value exceeded. Values are excluded from all LAGOS_LIMNO_ exports | All sample events | All LAGOS nutrient variables |
| RATIO | Measured ratio of N or P not feasible (e.g., TDP > 1.1 x TP). Values are excluded from all LAGOS_LIMNO_ exports | All sample events | Only nitrogen and phosphorus variables |
| TIME | Value was identified as outlier based on generalized extreme studentized deviate tests (p = 0.001) and > 8 x IQR using robust boxplots | Only individual lakes with at least 5 years of repeated measures | All LAGOS nutrient variables |
| BIPLOT | Value was identified as an outlier using distance-based outlier algorithm | All sample events | All LAGOS nutrient variables |

**Table S39. Number of observations flagged for each QAQC step by variable for LAGOS_LIMNO_ Ver 1.040.0**

|  |  | **QAQC Flags** | | | |  |  |
| --- | --- | --- | --- | --- | --- | --- | --- |
| **Variable** | **ETs** | **MAVs** | **RATIO** | **BIPLOT** | **TIME** | **Total Obs.** | **Total flagged** |
| Chla | 5 | 5 | NA | 34 | 88 | 128,269 | 132 |
| Colora | 0 | 3 | NA | 2 | 11 | 23,516 | 16 |
| Colort | 7 | 0 | NA | 20 | 10 | 24,892 | 37 |
| DKN | 0 | 8 | 104 | 0 | 6 | 4,411 | 118 |
| DON | 0 | 1 | NA | 2 | 7 | 5,712 | 10 |
| NH4 | 0 | 23 | 863 | 44 | 28 | 35,516 | 958 |
| NO2 | 0 | 0 | 87 | 1 | 2 | 7,241 | 90 |
| NO2NO3 | 1 | 4 | 799 | 47 | 78 | 63,428 | 929 |
| Secchi | 0 | 0 | NA | 9 | 90 | 656,663 | 99 |
| SRP | 1 | 59 | 250 | 1 | 21 | 14,298 | 332 |
| TDN | 0 | 0 | 0 | 0 | 0 | 14 | 0 |
| TDP | 0 | 26 | 249 | 5 | 18 | 4,878 | 298 |
| TKN | 3 | 16 | 1,306 | 61 | 21 | 40,789 | 1,407 |
| TN | 2 | 2 | 967 | 44 | 13 | 25,024 | 1,028 |
| TOC | 0 | 0 | NA | 0 | 0 | 278 | 0 |
| TON | 1 | 0 | 5 | 0 | 0 | 724 | 6 |
| TP | 1 | 205 | 404 | 50 | 378 | 192,269 | 1,038 |

Variable names are defined in the Glossary (Additional file 1).

**
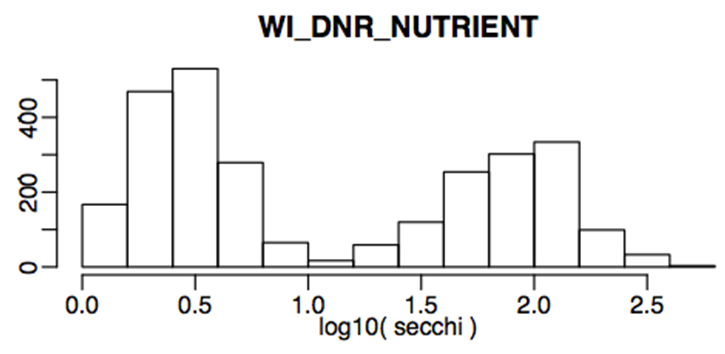
**

**Figure S25. Bimodal distribution observed in Wisconsin Secchi data (Y axis is the number of records) that ended up being the result of different Secchi values reported in either feet or meters when the associated metadata from the data source indicated values were in meters.**


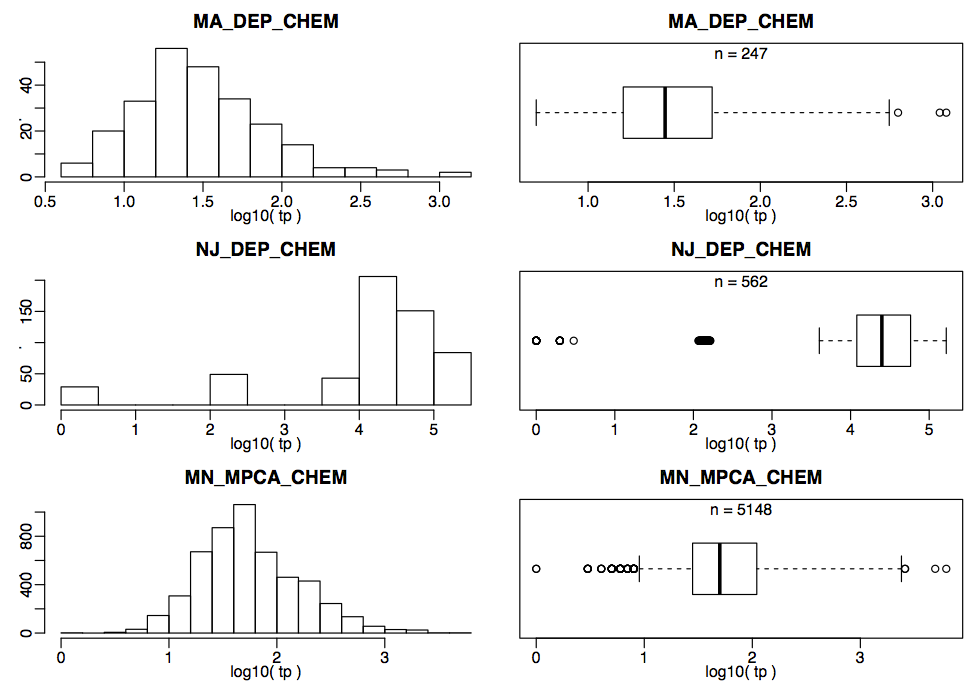


**Figure S26. Examples of unit conversion issues identified where histograms indicated the mode of the distribution was approximately three times greater or less than the values we typically observed in LAGOS_LIMNO_.** **Y axis is as for Figure S25.** These issues were resolved at the data import step after careful analysis by data managers.

**
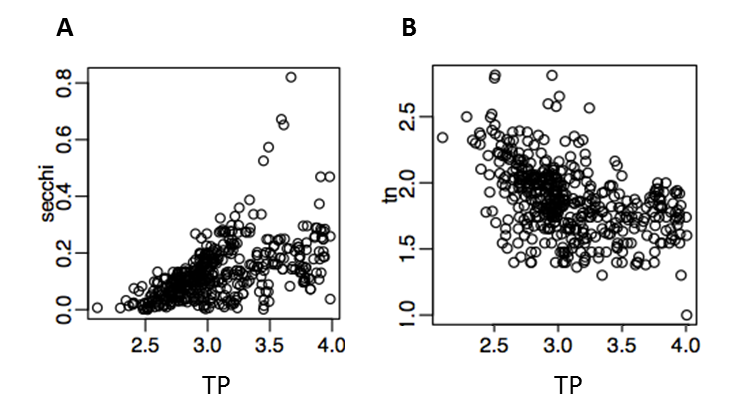
**

**Figure S27. Examples of erroneous bi-plots.** **(A)** Secchi depth increasing (*y*-axis) as total phosphorus (TP) concentrations increase (*x*-axis) and **(B)** total nitrogen (TN, *y*-axis) decreasing as total phosphorus (*x*-axis) increases. Both patterns are not consistent with ecological literature and data in LAGOS_LIMNO_. Resultant analysis by data managers determined that data import errors were associated with the inconsistencies and were fixed.
