# Supplementary material for: Building a multi-scaled geospatial temporal ecology database from disparate data sources: fostering open science and data reuse
Source: Gigascience. 2015 Jul 1;4:28. doi: 10.1186/s13742-015-0067-4 (PMC4488039; doi:10.1186/s13742-015-0067-4)
Supplement: Additional file 21: — Database export formats for LAGOSLIMNO. This file contains a description of the strategies for exporting the large volume of data from LAGOSLIMNO, and the challenges in dealing with sample depth or position in the lake. [file 13742_2015_67_MOESM21_ESM.docx]

Additional file 21

**Database export formats for LAGOS_LIMNO_**

Ed Bissell, Patricia Soranno

**Overview**

We faced two main challenges in creating database exports for LAGOS_LIMNO_. First, because of its vertical data format, the LAGOS_LIMNO_ database required transformation to produce horizontally structured export tables that are more amenable to statistical analysis. Second, the depth at which a sample is taken for water quality matters very much for limnological processes in lakes; and for studying eutrophication, often the surface water samples are the most commonly studied and analyzed. Therefore, to create data exports for later statistical analysis, we constrained the sampling events to those collected from the well-mixed epilimnion of lakes. Limnological variables such as nutrient concentrations are typically uniform throughout the epilimnion within a lake, but concentrations often differ between the epilimnion and deeper strata of lakes that are sufficiently deep to thermally stratify. Among our limnological data sources, there was wide variety in the ways in which they reported the depth of water samples from which nutrient concentrations and limnological variables were measured; sample depth information was also incomplete in some cases. Therefore, the LAGOS_LIMNO_ database includes information on the depth of water samples using two separate columns: *SamplePosition* (i.e., sample depth occurred within epilimnion, or elsewhere) and *SampleDepths* (i.e., actual depth from which water sample was collected). For situations in which *SamplePosition* and *SampleDepths* information was incomplete, we used the individual metadata files for the sampling program to infer, wherever possible, whether a water sample originated from the lake’s epilimnion.

We created two main exports of LAGOS_LIMNO_ that were used for subsequent statistical analysis. In the database as a whole, there are far more Secchi depth measurements than any other kind of measurement. Therefore, to minimize the number of null values in our data export, we created two different exports based on the following criteria: (1) 'All Secchi data export' (this export includes values for Secchi depths only); and (2) 'Any lake nutrient data export' (this export is created with the criterion that there must be at least one non-Secchi depth measurement taken on a sampling event. Then, for all sampling events that meet those criteria, all limnological data (including Secchi depth data if available) are exported. Because the ‘All Secchi data export’ requires few assumptions or criteria for export, the rest of this document describes the strategies to export the ‘Any lake nutrient data export’.

**Defining the data to be exported in LAGOS_LIMNO_**

Most statistical software programs or scripting packages are optimized for data tables that are in horizontal format, i.e. in which data values for each variable are stored in separate columns. However, a horizontal format is not the optimal orientation for data storage in a relational database because of the lack of flexibility, the large number of null values, and the extra columns required, which is why the LAGOS_LIMNO_ database module is stored in a vertical format (see Additional file 6 for details on the database design of LAGOS). Therefore, we needed to develop a process for exporting data from LAGOS_LIMNO_ tables, which are stored vertically, into exported tables that are stored horizontally so that they could be used for statistical analysis. For most of our analyses of LAGOS, we chose to analyze nutrient samples taken from the epilimnion, and so exported only those samples. Next we describe the decisions that needed to be made to create a database of epilimnetic samples.

Defining a lake sampling event: On the basis of our research questions (see Additional file 2), we decided that we should create a database that contained individual sampling events per lake and per day (i.e., no multiple samples for a single lake within a single lake basin or from multiple basins within the same lake).

**Sample event** (i.e. a single row in LAGOS_LIMNO_ =

*Programid, Lagoslakeid, Date, SamplePosition, SampleDepth*, …all water quality data of interest

We deleted sample events that were deemed to be 'duplicate' in situations for which sampling of the same lake on the same date was reported by different sampling programs/entities. This duplication happened for some lakes for which programs share data because the lakes are on state borders, and for some lakes for which different sampling programs just happened to sample the same lake on the same day. When a determination was made to delete a sample event, we kept the sample event from a) the original collector of the data (in the case of shared data) or b) a state agency (in the case of multiple sampling programs).

Defining an epilimnetic sample: Because all of the research questions that we identified related to understanding lake nutrients and primary productivity in lake epilimnion zones (or the near-surface sample, if the lake is unstratified), which are considered to be well-mixed, we sought samples from anywhere in the lake epilimnion zone. Unfortunately, the different sampling programs from which LAGOS data originated did not always specify the sample depth and/or sample position (whether the sample was taken from the epilimnion, metalimnion, or hypolimnion zone) in similar ways (and sometimes, not at all). Therefore, we needed to establish explicit criteria to create a database of epilimnetic samples. We defined an epilimnetic sample as a sample taken from any of the following: the surface, just below the surface, an integrated zone of the entire epilimnion, or any single depth from within the epilimnion. Because of the variety of ways in which sample depth information was reported across LIMNO data sources (and because depth information was often incomplete), the LAGOS_LIMNO_ database schema includes information on the depth of the sample using two separate columns:

1. *SamplePosition* - the location in the water column from which a sample is taken, with possible values EPI, META, HYPO, SPECIFIED, and UNKNOWN). EPI is the epilimnion; META is the metalimnion; HYPO is the hypolimnion; SPECIFIED means that an exact depth is given in the next column; and UNKNOWN means that there is no information on sample position or sample depth.
2. *SampleDepths* - the numeric depth in meters below the water surface (if known) this value corresponds to a SamplePosition of SPECIFIED).

**The criteria for establishing lake epilimnion (EPI) samples**

Storing *SampleDepth* and *SamplePosition* allowed us to retain as much information as possible from each source dataset during LAGOS_LIMNO_ development, so that a subsequent decision on how to identify and export EPI samples could be made in a consistent way. Ideally, the source dataset included sample position information such that we could clearly and easily select a single epilimnetic sample for each sampling event. However, in some cases of incomplete sample information, we manually examined any metadata associated with the program to be able to infer sample depth or position, and ultimately to identify epilimnetic samples. Next, we describe the specific problems we encountered associated with identifying epilimnetic samples; then we describe the approach that we used to identify a single epilimnion sample per date as well as edits that we made in LAGOS for programs that had lake water quality data with unknown *SampleDepth* and *SamplePosition*.

**Specific problems associated with the lack of sample depth or position information in LAGOS_LIMNO_**

- For samples taken using an integrated tube sampler:
  - The source dataset provided no information for *SampleDepth* and *SamplePosition.* For example, in LAGOS Ver 1.011, there were 109 out of 8,343 tube sampler total phosphorus (TP) samples that fell into this category.
- For samples taken using a grab sampler of some sort:
  - The source dataset provides no information for *SampleDepth* and *SamplePosition.* For example, in LAGOS Ver 1.011, there were 6,047 out of 88,530 grab sampler TP samples (which all came from two programs) that fell into this category
  - Source dataset provides *SampleDepth* but no *SamplePosition*

For example, in LAGOS Ver 1.011, there were 25,127 out of 88,530 grab sampler TP samples that fell into this category.

In summary, of the 101,562 sample events that include measurements for TP in LAGOS Ver 1.011, there were 29,687 sample events that had incomplete information regarding the sample depth and/or position (29% of the samples). Because of the large number of samples with this problem, we developed a strategy to infer sample position with sufficiently high confidence to accurately assign a sample position to samples that we believed were taken from the epilimnion zone. Because of the importance of sample position to lake water quality measurements, we were conservative in our criteria for inferring the range of lake depths that would correspond to an epilimnetic sample in each lake for which complete sample depth and/or position information was lacking.

**Approach to identify EPI samples when depth/position information was incomplete**

Below is our approach to assign a sample position to samples that have UNKNOWN *SamplePosition*, but known *SampleDepth.* The general goal of this strategy was to reduce the number of sample events for which we could not immediately identify an EPI sample because of incomplete information.

1. A single INTEGRATED sample of UNKNOWN *SamplePosition* per *program, lake, date, variable* combination was exported as EPI. We assume that most sampling programs that only take a single sample and that also use an integrated tube sampler do so to sample the epilimnion rather than the entire water column or rather than a lower zone, which is difficult to do with an integrated sampler.
2. A single GRAB sample of UNKNOWN *SamplePosition* per *program, lake, date, variable* combination, was exported as EPI. We assume that most sampling programs that sample ONLY one depth, typically sample the epilimnion.
3. For multiple GRAB samples of UNKNOWN SamplePosition per *program, lake, date, variable* combination, we only exported the sample closest to the surface as EPI.
4. For records in LAGOS in which it is not possible to determine an appropriate position or depth, then no values were exported as EPI, but the data were retained in the LAGOS database for further evaluation (see below).

Note, the above process occurs separately for each lake variable (e.g., TP, TN, Chl, Secchi) and data for all of the variables are eventually located on the same row and all are assumed to be taken from the same (or nearly the same) depth in the epilimnion. This means that samples for different variables could potentially be taken at different depths within the epilimnion; however, based on a test of a subset of the data, such discrepancies appear to be minimal. For example, for the first 11 datasets loaded into LAGOS (many of the largest state databases), only 1,000 out of 20,500 sample events had any difference at all in sample depths across four variables within a row (i.e., record), and the vast majority of differences were in the range of 0.5-2.0 meters, which could arguably be well within the epilimnion for the vast majority of lakes.

**Approach to identify EPI samples when depth information was lacking entirely**

We generally deleted records that had a null sampledepth and an unknown sampleposition. However, we manually checked situations where this was the case for large numbers of lakes to see if we could infer sample position or depth. Below is our approach to do this for some lake programs. The general goal of this strategy was to reduce the number of sample events for which we could not immediately identify an EPI sample because of a complete absence of sample depth and position information. For this step, all decisions were reviewed and made by a project limnologist on the team who reviewed the source data and metadata to determine the likelihood of epilimnetic sample depths.

1. For samples in LAGOS for which it is determined that it is likely to be from the eplilimnion based on review of the sampling program description and metadata (i.e., the sampling program only sampled at one depth in the epilimnion as stated in the metadata, but not the dataset itself), then the sample was given a *SamplePosition* of ‘EPI’ and a flag was created (*SamplePositionUncertain*) that identifies these samples by being given a value of ‘1’ for this flag.
2. Special case: We deleted data from LAGOS for data from very large and deep lakes in which samples were most likely taken at multiple depths (particularly when sampled on the same date), but the samples had UNKNOWN values for both *SamplePosition* and *SampleDepth*. We determined that these data had insufficient metadata to include in LAGOS. This only happened for one lake in one program, i.e., Lake Champlain.

**Example decisions made for some programs for which depth information was lacking in LAGOS Version 1.040.0**

1. WI_DNR_NUTRIENT - UNKNOWN *SampleDepth* and *SamplePosition* records (2,764 out of 9,364) collected from a citizen monitoring program were classified as EPI because we reasonably assumed that citizen monitoring programs collect surface samples.
2. VT_DWQ_NUTRIENTS - Some samples with UNKNOWN *SampleDepth* and *SamplePosition* were collected with a Hose or TygonHose which is an INTEGRATED sampler (these records were mistakenly attributed as GRAB). These were changed to INTEGRATED and attributed as EPI and *SamplePositionUncertain* = 1.
3. All UNKNOWN *SampleDepth* and *SamplePosition* samples for Lake Champlain were deleted (1,422) because it is an extremely deep lake with a long-term record so it did not make sense to have samples with absolutely no depth information when there is a considerable amount of data with depth information on this lake.
4. IL_ALMP - We removed 2,552 records that were NULL for *SampleDepth* and UNKNOWN for *SamplePosition*. There was not enough information in metadata to indicate where samples were taken in the water column, and multiple samples were recorded per date (and so likely from multiple depths that were not recorded), so there was a large potential to misclassify. *SamplePosition*.
5. PA_DEP - We removed 1,120 records that were NULL for *SampleDepth* and UNKNOWN for *SamplePosition*. There was not enough information in the metadata to indicate where samples were taken in the water column and multiple samples per date were recorded, so there was a potential to misclassify *SamplePosition*.

**Specific steps to export limnological data (LAGOS_LIMNO_)**

To export LAGOS_LIMNO_ data, we pivoted the data from a vertical orientation to a horizontal orientation using the Crosstab function in PostgreSQL. We also used a series of UPDATE statements that optimizes the number of EPI samples that can be exported from LAGOS_LIMNO_. Using the criteria that we established above, we implemented the following specific steps using SQL in PostgreSQL, the database management system for LAGOS.

1. Determine sample events:
   1. A sample event is defined as a unique combination of *Programid, Lagoslakeid, Date, SamplePosition,* and *SampleDepth*
   2. A sample event must have at least 1 *datavalue* (that is not a Secchi value)
2. Identify shallowest depth per sample event:
   1. The minimum sample depth for each ‘GRAB’ or ‘PROBE’ sample event is calculated if the variable sampled was not Secchi and had an ‘UNKNOWN’ *SamplePosition*
3. Select *datavalues* to be exported based on the criteria in the following order of preference:
   1. Pick each non-Secchi value with *SamplePosition* = 'EPI' OR *SampleDepth* = 0
   2. If no values in 1) pick an INTEGRATED sample
   3. If no values in 1) and 2) pick the sample with shallowest depth (if depth available)
4. Assign values for special flag columns (*qualifier, methodinfo*)
5. Use PostgreSQL Crosstab function to pivot vertical variable-specific columns (*censorcode, labmethodname, detectionlimit*) to horizontal orientation
6. Join *datavalue* and supporting columns from 1, 3, 4, and 5 to create the horizontal export.
