# Supplementary material for: Building a multi-scaled geospatial temporal ecology database from disparate data sources: fostering open science and data reuse
Source: Gigascience. 2015 Jul 1;4:28. doi: 10.1186/s13742-015-0067-4 (PMC4488039; doi:10.1186/s13742-015-0067-4)
Supplement: Additional file 22: — Example user documentation for LAGOSLIMNO. This file contains the user documentation that is provided to project participants for each new version of LAGOSLIMNO. [file 13742_2015_67_MOESM22_ESM.docx]

Additional file 22

**Example user documentation for LAGOS_LIMNO_**

Ed Bissell, Patricia Soranno

**LAGOS_LIMNO_ Version 1.040.0 User Documentation**

Ed Bissell and Patricia Soranno, *Michigan State University*

11/10/2014

**What’s New in Version 1.040.0**

- Significant changes to how LAGOS exports are structured: @ Version 1.040.2 limno (nutrients & secchi) are provided along with a lakeinfo table, program info table, and sources info table
- List of programs included in this version: CT_DEEP_CHEM, CT_DEEP_CHEM_GRANT_PROGRAM, CT_DEEP_SECCHI, IA_CHEMISTRY, IL_ALMP_1999_2000, IL_ALMP_2001_2004, IL_ALMP_2004_2005, IL_ALMP_2006_2008, IL_ALMP_2009, IL_ALMP_CHMPG_2006, IL_ALMP_SPRNGFLD_2004_2006, IN_chemistry, MA_DEP_CHEM, ME_ANP_post_2006, ME_ANP_pre_2006, ME_DEP_CHEM, MI_CORPS_CHEM, MI_DEQ_COLOR, MI_DEQ_LWQA, MI_LEELANAU_CHEM, MI_LTBB_CHEM, MI_MSU_CHERUVELIL, MI_MSU_LNDSCP_CHEM, MI_TIP_MITT_CHEM, MI_TIP_MITT_VOLUNTEER, MN_MPCA_CHEM, MN_MPCA_SECCHI, MO_LMVP_CHEM, NH_LKTROPH, NH_SUNAPEE, NJ_DEP_CHEM, NY_CDEP_CATSKILL, NY_CSLAP, NY_ONEIDA_CHEM, OH_ODW_CHEM, PA_DEP_CHEM, RI_URIWW_CHEM, VT_DWQ_NUTRIENT, WI_DNR_NUTRIENT, WI_LTER_SECCHI
- The naming convention for a horizontal EPI export of nutrient data has been changed from VersionA to EPI_NUTR for clarity
- The naming convention for a horizontal Secchi export has been changed from VersionC to SECCHI for clarity
- Flagged *datavalues* (per our QAQC efforts) are not included in any exports
- Added greatlakes flag to LAGOS. Lakes that are one of the Great Lakes or closely connected to the Great Lakes will not be included in any exports (140 records in epi_nutr)
- Eventids for EPI_NUTR and Secchi exports were reset due to changes in how sample events were determined
- Deleted all records with sample dates that were anomalous, i.e. > 2013 for datasets with no sampling in 2014
- Removed mean lake depths that were greater than max lake depth
- Secchi values in nutrient export that didn’t have accompanying nutrient data have been removed
- Chla+b+c was dropped from LAGOS and lagosvariableid 10 (chla unfiltered) was merged with *lagosvariableid* (9) (chla) and "CHLA_UNFILTERED" was added to methodinfo column
- Deleted sample events that were deemed to be “duplicate” based on the fact that they were the same lake sampled on the same date but by different programs. This happens by coincidence for some lakes that are on state borders, when programs share data, and when different sampling programs just happen to sample the same lake on the same day. When a determination was made to delete a sample event the preferred sample event to be retained was from a) the original collector of the data or b) collected by a state agency.
- Removed all lakes less than 1 ha from LAGOS, and all lakes otherwise not in the Master Geodatabase (our master file that contains all lakes in our study extent). These were usually a FCODE that was previously filtered out.

**Overview:** The LAGOS (**LA**ke **G**e**OS**patial) database is a multi-scale spatial/temporal database of lake chemistry and landscape characterization for thousands of lakes in a 17 state study region in the Upper Midwest and North Eastern U.S. When complete, it is estimated that LAGOS will consist of nearly 10,000-15,000 lakes ≥ 4 ha with lake chemistry data (LAGOS_LIMNO_) and approximately 50,000 lakes ≥ 4 ha in surface area with delineated watersheds and multi-scale landscape characterizations (LAGOS_GEO_). These two components of the LAGOS database (LAGOS_LIMNO_ and LAGOS_GEO_) represent two separate but related efforts to create the largest known spatially-explicit lake water chemistry and landscape database at a sub-continental scale.

LAGOS_LIMNO_ currently consists of 17 previously identified LAGOS primary variables*.* Nutrient and Secchi exports include a column for each variable (<variableshortname>_qual) that provides qualifier information from the source dataset. This column may seem cryptic to users (explanations of flags are usually not provided in the data) and will be used to determine the fitness for use of individual data records as part of the QAQC process.

Users are advised to always be aware of the specific version of LAGOS they are using for a particular analysis and to always cite the version of LAGOS used when referencing their results.

For LAGOS 1.040.0 a single horizontal flat file containing relevant columns for analysis is no longer provided. Instead an EPI export of chemistry data and a Secchi export are provided with one lake information table and two sampling related “metadata” tables. GEO data will be provided as exports in a series of thematic tables (CHAG, LULC, CONN, CLIM_ANN, and CLIM_MON).

LAGOS database design: LAGOS is a relational database based on the ‘Observations Data Model’ (ODM) database design created by CUAHSI for the hydrologic sciences (<http://his.cuahsi.org/odmdatabases.html>). This design is "intended to facilitate the export of data and is designed to store observations and sufficient metadata about the data values to provide traceable heritage from raw measurements to usable information allowing them to be unambiguously interpreted and used". This database format stores values in a vertical structure (values for all limnology variables are stored within one column rather than separate columns).

**Introduction to Documentation**

This document is a guide that describes how the LAGOS database will be exported and made available to CSI-Limnology personnel for individual analysis. Data files that make up the export (for a particular version number) will be placed in a Dropbox directory for your access. Note that each version number will have its own folder for future reference, as well as its own documentation that describes the contents and updates specific to that LAGOS version.

**LAGOS Version 1.040.0**: This version of LAGOS includes 40 datasets (based on *programid* or *programname*) that is generally comprised of one larger state agency sampling program for each of the 17 states and additional representative datasets that have been prioritized based on filling data gaps within the datasets. QAQC has been performed on this version and egregious values have been removed and suspect values have been flagged and excluded from the export process. The folder contains joined tables only with two different formatting options, Tab-delimited (txt extension) and CSV (csv extension). All tables display NULL values as NA and include a header (column names) as the first line in the file. The data are exported in horizontal format (1 column for each variable + metadata columns).

**LAGOS Version 1.040.0** - **Data Directory**

Below is an outline of the contents of the Version 1.040.0 data directory including:

- Table S40: names of the text files that make up this LAGOS_LIMNO_ export with a brief description of the file
- Table S41: descriptions of LAGOS_LIMNO_ columns as they are stored in LAGOS
- Figure S28: diagram showing relationship between LAGOS_LIMNO_ database tables
- Figure S29: example graphic showing a common difference between definition of LAGOs lakes (NHD-based) and lakes from source datasets

**LAGOS Versioning Description**

The following LAGOS version notation is used to denote the LAGOS version.

<major><datasetnumber><minor>

- <major> Number indicates the major version which will be updated when a major revision to LAGOS occurs such as the inclusion of a QAQC process, expansion of the study area, inclusion of non-primary variables or inclusion of the GEO data variables
- <datasetnumber> Number indicates the number of datasets loaded
- <minor> Number indicates a minor revision to the database such as fixing minor data problems

Below are examples of possible LAGOS versions (underscores and leading/trailing 0’s added for clarity)

- *01_000_0 => initial LAGOS version 1.000.0 with 0 datasets (empty schema only)*
- *01_010_0 => LAGOS version 1.010.0 with 10 datasets*
- *01_011_0=> LAGOS version 1.011.0 with 11 datasets*
- *01_011_1=> LAGOS version 1.013.0 with 11 datasets and 1st minor revision*
- *01_022_2=> LAGOS version 1.022.2 with 22 datasets and 2nd minor revision*
- *02_049_0=> LAGOS version 2.049.0 with 49 datasets*

**Table S40. LAGOS Export Tables**

Below is a list of tables exported in this version of LAGOS_LIMNO_, but it is not necessarily an exhaustive list of tables in LAGOS_limno_. In some cases, to avoid confusion and minimize unnecessary columns, only the data from supporting data tables relevant to the actual datasets loaded into LAGOS at that version were exported.

| **ID** | **Exported Table File Name** | **Description** | **Column List** |
| --- | --- | --- | --- |
| **1** | **lagos_epi_nutr_10400.txt**  Record Count: 164,402  Column Count: 87 | EPI only chemistry data values + a corresponding Secchi sample, and the description of the sampling and laboratory methods, program name, qualifiers, etc. | eventida10400, lagoslakeid, programname, programtype, lagosversion, sampledate, chla, colora, colort, dkn, doc, nh4, no2, no2no3, srp, tdn, tdp, tkn, tn, toc, ton, tp, secchi, colora_qual, colort_qual, dkn_qual, doc_qual, nh4_qual, no2_qual, no2no3_qual, srp_qual, tdp_qual, tkn_qual, toc_qual, tp_qual, secchi_qual, chla_censorcode, colora_censorcode, colort_censorcode, dkn_censorcode, nh4_censorcode,  no2_censorcode, no2no3_censorcode, secchi_censorcode, srp_censorcode, tdp_censorcode, tkn_censorcode, tn_censorcode, toc_censorcode,  ton_censorcode, tp_censorcode, chla_detectionlimit, colora_detectionlimit, colort_detectionlimit, dkn_detectionlimit, doc_detectionlimit, nh4_detectionlimit, no2_detectionlimit, no2no3_detectionlimit, secchi_detectionlimit, tdn_detectionlimit, tdp_detectionlimit, tkn_detectionlimit, tn_detectionlimit, toc_detectionlimit, ton_detectionlimit, tp_detectionlimit, chla_labmethodname, colora_labmethodname, colort_labmethodname, dkn_labmethodname, doc_labemthodname, nh4_labmethodname, no2_labmethodname, no2no3_labmethodname, srp_labmethodname, tdn_labmethodname, tdp_labmethodname, tkn_labmethodname, tn_labmethodname, toc_labmethodname, tp_labmethodname, chla_methodinfo, nh4_methodinfo, secchi_methodinfo, sampleyear, samplemonth |
| **2** | **lagos_secchi_10400.txt**  Record Count: 656,574  Column Count: 13 | Secchi-only data values | eventidc10400, lagoslakeid, programname, programtype, lagosversion, sampledate, secchi, secchi_censorcode, secchi_qual, secchi_methodinfo, greatlakes, sampleyear, samplemonth |
| **3** | **lagoslakes_10400.txt**  Record Count: 141,271  Column Count: 41 | All lakes in LAGOS Master Geodatabase (≥ 1 ha) with the zone identifier that each lake is within and any lake-specific info from sample programs for lakes that are in LAGOS_LIMNO_ | lagoslakeid, nhdid, nhd_lat, nhd_long, lake_area_ha, lake_perim_meters, fcode, ftype, greatlakes, lagosname1,meandepth, maxdepth, In_NWI, iws_zone_id, HU4_ZoneID, HU4_Name, HU6_ZoneID, HU6_Name, HU8_ZoneID, HU8_Name, HU12_ZoneID, HU12_Name, EDU_ZoneID, EDU_Name, COUNTY_ZoneID, COUNTY_Name, State_Name, STATE_ZoneID, HU4, HU6, HU12, HU8, STATE, IWS_Area_Ha, iws_perim_km, lakeconnectivity, Upstream_Lakes_4ha_Count, Upstream_Lakes_10ha_Count, Upstream_Lakes_4ha_Area_ha,  Upstream_Lakes_10ha_Area_ha, Glacial |
| **4** | **lagos_program_10400.txt**  Record Count: 40  Column Count: 15 | Information regarding the sampling program or effort that collected the limnological data sampling | programid, sourcename , programname, composite, programtype, fundingsource, programdescription, labtype, metadataid, sourceid, programlink, programstatus, datasharingpolicy, datasharingpolicydetails, comments |
| **5** | **lagos_source_10400.txt**  Record Count: 28  Column Count: 3 | Information regarding the agency or organization responsible for collecting the limno data or that provided the data to CSI-LIMNO | sourceid, organization, sourcedescription |

**Table S41: LAGOS Column Descriptions**

*Note: These are columns that physically stored in the LAGOS database and are a superset of what is generally exported at any particular version.*

**TABLE**: **lagosvalues**

**COLUMN: sampledate** **-** date at which the sample was collected, stored in date format (no time), changed from date to sampledate at version 1.032.0 because date is a reserved keyword for ESRI File Geodatabases (@ ArcGIS 10.1)

**COLUMN: lagosvariableid** **-** integer identifier that references the variable that was measured; links data values to their variable in the lagosvariables table

**COLUMN: sampledepth** **-** sample depth below the water surface in meters

**COLUMN**: **detectionlimit -** if applicable, the detection limit used by the particular sampling regime under which the sample is collected; if null this is assumed to be not applicable or unknown

**COLUMN**: **sampleposition** - name of sample position within water column (i.e. EPI, META, HYPO), if applicable

**COLUMN**: **programid** - integer id for the program under which the sampling effort was conducted

**COLUMN: qualifier** - added to retain diverse set of dataflags for use in QAQC process, Note: As of 12 September 2013, no QAQC has been conducted and the value of this column may indicate data values unfit for use, may be removed in later versions after QAQC

**COLUMN: datavalue** - numeric value of the observation, Note: As of 12 September 2013, values have not been checked for outliers and anomalies

**COLUMN**: **comments** - relevant text concerning sample value that cannot be standardized, may be removed in later versions after QAQC process

**COLUMN**: **legacyId** - identifying id from source dataset that can be used to trace provenance of datavalue; note that this should be a sampleid or similar column; will not exist for all source datasets

**COLUMN**: **censorcode** - identifies whether a particular datavalue is censored and if so what type of censored observation it is

**COLUMN**: **sampletype** - method of sample collection, i.e. grab, integrated, unknown

**COLUMN**: **labmethodname** - name of laboratory processing procedure, from a standards body if available

**COLUMN:lakeid -** internal lakeid to for relational purposes (not provided to users)

**COLUMN:basintype -** Boolean indicating status of sample basin as primary basin

**COLUMN:labmethodinfo -** descriptive information concerning analytical method, cannot be standardized

**COLUMN: sourcevariableid -** unique identifier that relates to variable information from the source dataset

**COLUMN: lagosvalueid -** unique identifier for data values

**COLUMN: methodinfo -** flag column used to differentiate variables that were sampled using methods that are important to consider but not numerous or significant enough to warrant creation of a new variable

**COLUMN: lagosversion -** current version of LAGOS that the data record belongs too; all records will be from the same version

**COLUMN: subprogram -** if source dataset was a composite (aggregation of multiple sampling programs) indicates the name of the sampling program under which the sample was collected

**COLUMN: lagoslakeid** - from nhdlakes table, populated in lagosvalues table for convenience

**COLUMN: samplepositionuncertain** - reflects uncertainty in the sampleposition column, denotes a record that was UNKNOWN for sampleposition and sampledepth and had its sampleposition permanently changed to EPI as a best guess (this last happened for version 1.011.1? and has not been changed since)

**COLUMN: newsampleposition** - indicates a record that was exported as an EPI sample in horizontal export version a because it was deduced to be the 'most EPI' record for that sample

**COLUMN: eventida -** unique combination of programid, lakeid, date; establishes concept of a unique surface or epilimnion sampling event facilitating the pivot of unique data records from a vertical to a horizontal database orientation (Horizontal Analysis Version A); not populated for Secchi observations

**COLUMN: eventidb -** unique combination of programid, lakeid, date, sampledepth, sampleposition; establishes concept of a unique sampling event facilitating the pivot of unique data records from a vertical to a horizontal database orientation (Horizontal Analysis Version B); populated for non-Secchi observations and samples at all depths

**COLUMN: eventidc - unique combination programid, lakeid, date;** establishes concept of a unique sampling event facilitating the pivot of unique data records from a vertical to a horizontal database orientation (Horizontal Analysis Version C); populated for uncensored Secchi observations

**TABLE**: **lagosvariables**

**COLUMN: lagosvariableid** - integer identifier for each limnology sample variable

**COLUMN: variablename** - full text name of the variable that was measured, observed, modeled, etc., from the lagosvariablecv controlled vocabulary table which is sourced from ODM

**COLUMN: variableunitsid** - integer identifier that references the record in the Units table giving the units of the data values associated with the variable

**COLUMN: status** - indicates whether the variable is one of the 17 standardized and grouped (lumped) variables to be initially loaded into LAGOS

**COLUMN: comments** - relevant text concerning sample limnovariable that cannot be standardized

**COLUMN: variableshortname** - abbreviated/shortened variable name for use in analysis versions

**TABLE**: **lagoslakes** - uniquely identified sample lake based on NHD

**COLUMN: lagoslakeid** - unique integer identifier for lake in LAGOS; this is the unique lake identifier to use for analysis as it is an integer, will never change and will provide a link to landscape data

**COLUMN: nhdid** - Permanent_Identifier from the NHD that the sample lake was linked to

**COLUMN: lagosname1** - name of lake from source dataset, this should be viewed as the authoritative lake name for LAGOS

**COLUMN: lagosname2** - secondary name of lake from source dataset; this is usually populated because a lake was sampled by multiple programs and each program had a different name for the lake.

**COLUMN: maxdepth** - maximum depth of lake (meters) provided by source program or other source

**COLUMN: meandepth** - mean depth of lake (meters) provided by source program or other source

**COLUMN: maxdepthsource** - source of information for maxdepth, if same as programname the information accompanied the limnology data otherwise it was compiled from another source such as bathymetry maps

**COLUMN: meandepthsource** - source of information for meandepth, if same as programname the information accompanied the limnology data otherwise it was compiled from another source such as bathymetry maps

**COLUMN: zmax** - boolean indicating whether or not maxdepth value is a zmax value, i.e. the deepest point in lake at sample location rather than the overall maxdepth of the lake; in cases where zmax values differ for different sampling events the deepest zmax value is always used. This column should serve as a flag indicating that maxdepth is an approximation and should be used with caution. 1 = maxdepth is zmax, 0 = maxdepth is an actual maxdepth for lake

**COLUMN: greatlakes** - 1 or 0 if a waterbody is one of the Great Lakes per the NHD

**TABLE: lagos_all_lakes_1ha -** attributes from NHD waterbody feature class within LAGOS study extent for lakes ≥ 1 ha that are of specific ftypes included in LAGOS_GEO_

**COLUMN: nhdid** - 40-char GUID value that uniquely identifies the occurrence of each feature in The National Map, basis for lagoslakeid

**COLUMN:ftype** - three-digit integer value; unique identifier of a feature type

**COLUMN**:**fcode** - five-digit integer value; comprised of the feature code and combinations of characteristics and values

**COLUMN: lake_area_ha** - surface area of lake (hectares) calculated from polygon in NHD Waterbody feature class projected to Albers USGS (wkid: 102039)

**COLUMN: lakeconnectivity** - Classifies lakes based on hydrologic connectivity determined by tracing the NHD network. Lake Connectivity Classifications are: Isolated - has no stream connectivity, Headwater - has one outlet and no upstream connectivity, DR_ Stream (Drainage Stream) - has upstream connectivity to streams and/or lakes < 10 ha in area, DR_LakeStream (Drainage Lake Stream) - has upstream connectivity to streams and lakes > 10 ha in area

**COLUMN: in_nwi** - yes/no if US Fish & Wildlife Service NWI (National Wetland Inventory) is available at location of lake

**COLUMN: hu4_zoneid** - unique zone identifier that links lake to LAGOS_GEO_ data calculated at HUC4 scale

**COLUMN: hu4_name** - text name of HUC4

**COLUMN: hu6_zoneid** - unique zone identifier that links lake to LAGOS_GEO_ data calculated at HUC4 scale

**COLUMN: hu6_name** - text name of HUC6

**COLUMN: hu8_zoneid** - unique zone identifier that links lake to LAGOS_GEO_ data calculated at HUC8 scale

**COLUMN: hu8_name** - text name of HUC8

**COLUMN: hu12_zoneid** - unique zone identifier that links lake to LAGOS_GEO_ data calculated at HUC12 scale

**COLUMN: hu12_name** - text name of HUC12

**COLUMN: edu_zoneid** - unique zone identifier that links lake to LAGOS_GEO_ data calculated at the EDU scale

**COLUMN: edu_name** - text name of EDU

**COLUMN: county _zoneid** - unique zone identifier that links lake to LAGOS_GEO_ data calculated at County scale

**COLUMN: county_name** - text name of County

**COLUMN: state_zoneid** - unique zone identifier that links lake to LAGOS_GEO_ data calculated at State scale

**COLUMN: hu4** - 4 digit HUC identifier

**COLUMN: hu12** - 12 digit HUC identifier

**COLUMN: hu8** - 8 digit HUC identifier

**COLUMN: state** - state name abbreviated (2 letters)

**COLUMN: lagoslakeid** - unique integer identifier for lake in LAGOS; this is the unique lake identifier to use for analysis as it is an integer; will never change and will provide a link to landscape data

**COLUMN: lake_lat** - latitude of lake centroid (in decimal degrees NAD83), calculated from NHD polygon geometry; for irregularly shaped polygons coordinate is not necessarily within NHD lake polygon

**COLUMN: lake_long** - longitude of lake centroid (in decimal degrees NAD83), calculated from NHD polygon geometry; for irregularly shaped polygons coordinate is not necessarily within NHD lake polygon

**COLUMN: lake_perim_meters** - perimeter of lake polygon from NHD projected to Albers USGS (wkid: 102039)

**TABLE: sourcevariables**

**COLUMN:sourcevariableid** - integer identifier for each unique source variable (on a source dataset basis)

**COLUMN: sourcevariablename** - what the source dataset calls a particular variable (as close as possible); retained for reasons of provenance and in case LAGOS variable groupings are to be changed in future

**COLUMN: sourcevariabledescription** - description of sampled variable derived from metadata at the level of the individual sample value

**TABLE**: **sampletypecv**

**COLUMN: term** - categorical description of sample depth type, e.g. EPI, META, HYPO

**COLUMN: definition** - definition/explanation of categorical description of sample depth within water column

**TABLE**: **units**

**COLUMN: unitsid** - unique integer identifier that identifies each unit

**COLUMN: unitsname** - full text name of the units

**COLUMN: unitstype** - text value that specifies the dimension of the units

**COLUMN: unitsabbreviation** - text abbreviation for the units names that with symbols and spaces removed

**TABLE**: **basintypecv**

**COLUMN: term** - from lagosvalues table, value indicating the status of the sample basin as the deepest (primary) and thus preferred sample basin to use if more than one is available for a lake, possible values are primary, not primary, and unknown. Note: the information content in source datasets regarding sampling basintype is very lacking. Use this column with caution.

**COLUMN: definition** - definition/explanation of basintype values

**TABLE**: **censorcodecv**

**COLUMN: term** - code for how a datavalue is censored if at all

**COLUMN: definition** - definition/explanation of censor codes

**TABLE**: **program**

**COLUMN: programid** - unique integer identifier that identifies each unit

**COLUMN: sourcename** - name of the organization that collected the data, in abbreviated form from metadata

**COLUMN: programname** - name of the sampling/monitoring program that collected the data, in abbreviated form from metadata

**COLUMN: composite** - indicates if a dataset consists of multiple aggregated datasets, 1 = yes

**COLUMN: programtype** - categorical description of the type of sampling/monitoring effort

**COLUMN: fundingsource** - categorical description of the entity that funded the program

**COLUMN: programdescription** - longer text description of program

**COLUMN: labtype** - categorical description of the type of laboratory that processed data samples

**COLUMN: metadataid** - unique integer identifier that identifies each metadata entry

**COLUMN: sourceid** - unique integer identifier that identifies each data source

**COLUMN: programlink** - link to specific program webpage or internet resource

**COLUMN: programstatus** - indicates whether a program is ongoing (at time of metadata compilation) or sampling has been completed

**COLUMN: datasharingpolicy** - categorical description of restrictions placed on the data by the owner

**COLUMN: datasharingpolicydetails** - specific details of how the data can be shared

**COLUMN: programcomments** descriptive information compiled during the metadata authoring process, usually not applicable to data user

**TABLE**: **sources**

**COLUMN: sourceid** - unique integer identifier that identifies each data source

**COLUMN: organization** - name of the organization that collected the data, in abbreviated form from metadata

**COLUMN: sourcedescription** - full text description of the source of the data

**TABLE**: **fundingsourcecv**

**COLUMN: term** - controlled vocabulary term used to identify funding source agency of sampling program for informational purposes and for cases in which data source owner requires funding source to be cited

**COLUMN: fundingsourcedefinition** - explanation /definition of funding source term

**TABLE**: **datasharingpolicycv**

**COLUMN: term** - controlled vocabulary for terms used to describe restrictions on data access and dissemination stipulated by data source owner

**COLUMN: datasharingpolicydefinition** - explanation /definition of data sharing policy term

**TABLE**: **metadata**

**COLUMN: metadataid** - unique integer identifier for each metadata entity

**COLUMN: title** - title of data from a specific data source

**COLUMN: abstract** - narrative describing the data source, program, sampling methodology, etc.

**COLUMN: metadatalink** - link to additional metadata information; will probably be replaced with a link to authoritative EML metadata file

**COLUMN: citation** - text string that indicates the citation to be used when referencing data from the program

**COLUMN: temporalscale** - approximate time period of sampling program; note that this is derived from metadata and may not accurately reflect the actual data in LAGOs due to lake linking issues, QAQC, selection of variables for LAGOS, and any other number of issues up to and including acts of God and random acts of kindness

**COLUMN: metadatacomments** - descriptive information compiled during the metadata authoring process, usually not applicable to data user

**LAGOS Database Table Relationships**

Below is an Entity Relationship Diagram (ERD) that provides a visual representation of the relationships between database tables in the LAGOS_LIMNO_ schema. Not all of these tables (or columns) are included in the LAGOS export process as some tables are used to maintain data provenance and are not necessary for analysis. Also, some tables were developed during the initial LAGOS database design but do not include enough data to warrant including in the export process at this time.


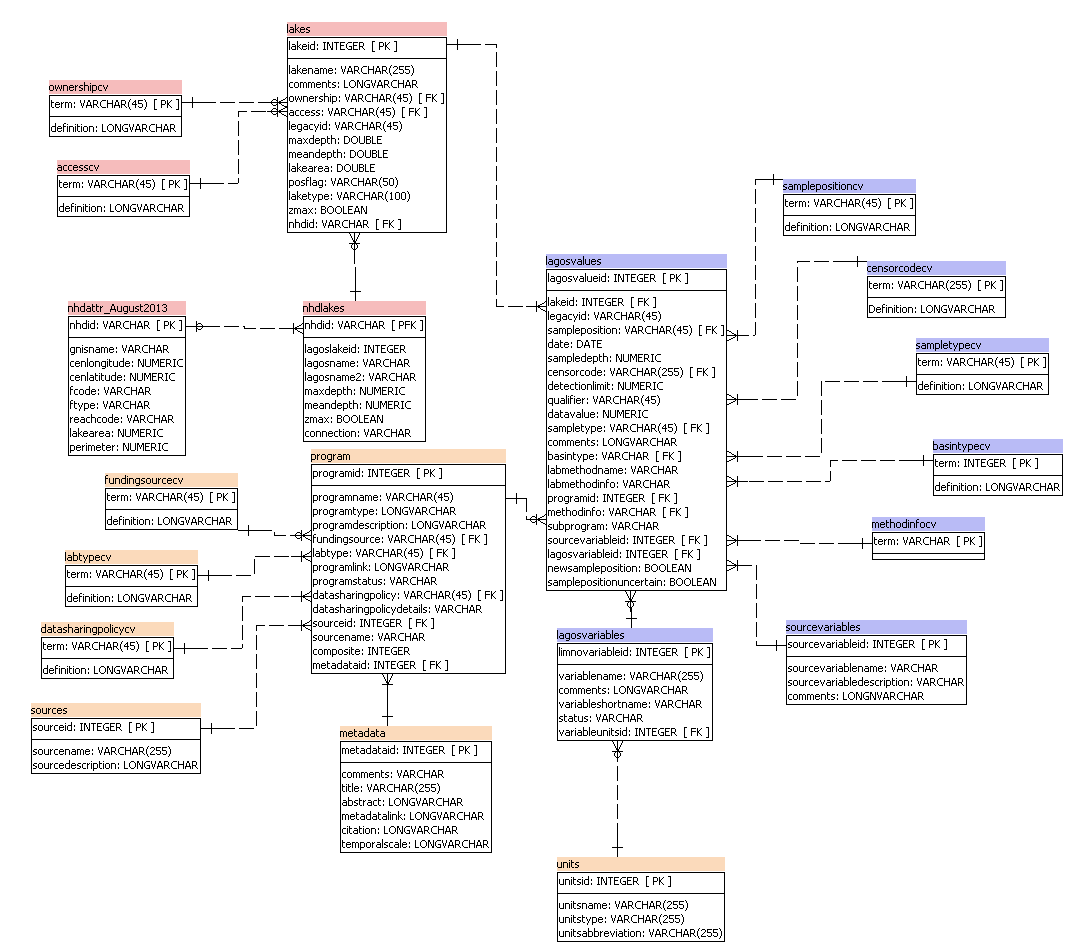


**Figure S28. Excerpt from LAGOS Entity Relationship Diagram showing tables that comprise the LAGOS_LIMNO_ component of LAGOS and how they are related.**

**Identifying unique lakes in LAGOS**

A lake in LAGOS_LIMNO_ must be unique as represented by the NHD Waterbody feature class with an assigned *Permanent_Identifier*. Lakes from source datasets are uniquely identified for data provenance reasons based on site or *basinid* information, *lakeid* or *lakename*, in that order of preference (increasing to decreasing specificity). The NHD frequently treats chains of lakes and multi-lobed lakes as a single waterbody, instead of multiple, individual lakes that the source datasets often represent them as; and multiple sample programs may overlap in the lakes that they sample. For these reasons, in order to maintain data provenance (the ability to trace the lineage of individual data values to their final representation in LAGOS) source-lake information is maintained within LAGOS, but actual lake-specific information is provided to data users at the level of the *nhdlake* only. Built into this process is the ability to identify duplicate lakes across sample programs and aggregate lake-specific characteristics (*maxdepth*, *meandepth*, *lakename*, etc.) accordingly.


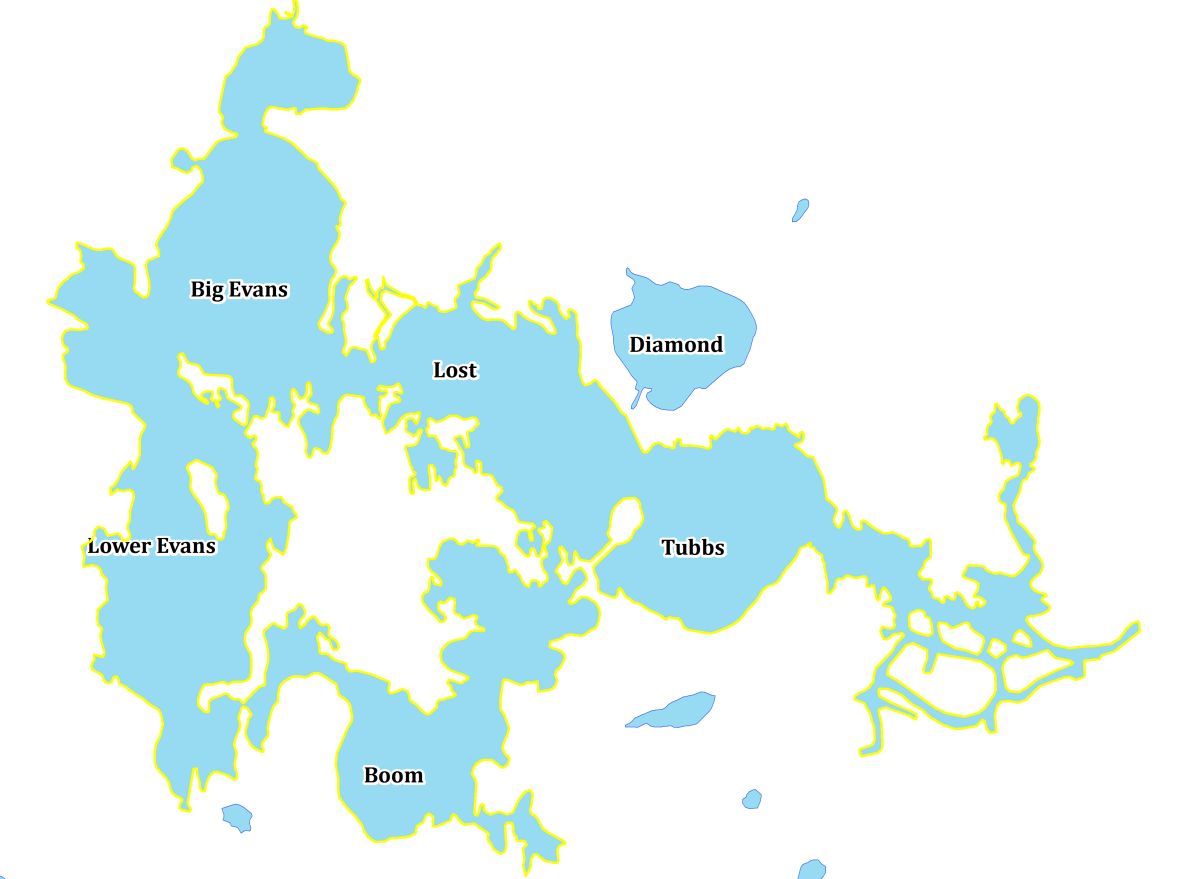
**Figure S29. Example of chain of lakes issue.** NHD treats the entire polygon highlighted in yellow as one lake while data from source program was collected for five distinctly named lakes (Big Evans, Lower Evans, Lost, Tubbs, and Boom Lakes).

**NHD Lakes vs. Source Lakes**

There are two levels of lake-specific information in LAGOS_LIMNO_, NHD Lakes, and Source Lakes. Because the information content in source lake datasets is so varied and has proven to be so low in terms of positional and morphometric characteristics, in Version 1.013.0 (and probably subsequent versions) the only source-lake-specific information made available to LAGOS users is the name of the lake, id of the lake from the source dataset and *maxdepth* and *meandepth* which are not part of the NHD. It is important to understand why we are linking all lakes to the NHD given that large NHD lakes often consist of multiple source lakes.

The reference GIS dataset that all LAGOS_LIMNO_ lakes are linked to is the 1:24,000 NHD. Linking to the NHD facilitates:

1. usage of a high resolution, consistent, seamless, national GIS waterbody dataset that locates all lakes on the landscape (not all source datasets include coordinates)
2. delineation of lake-specific watersheds and geometric buffers and subsequent landscape characterization
3. characterization of freshwater connectivity using the NHD flow network
4. identification of unique lakes across sampling programs
5. access to information contained in NHD attributes such as gnis_name, lakearea, perimeter, and ftype and fcode (characterization of lakes such as lake/pond vs. reservoir), info that is often missing in source datasets

In LAGOS_LIMNO_ all lake-specific characteristics that begin with “*nhd*_” (and the *nhdid*) are NHD GIS lake attributes. Source lake information in LAGOS_LIMNO_ includes *lakename*(s) (*lagosname1* and/or *lagosname2*), *lakeid* or *stationid* (*legacylakeid*) and *maxdepth* and/or *meandepth* information. In the example above (Figure S29), the NHD lake that comprises the five source lakes will be attributed with whatever the maximum *maxdepth* and *meandepth* from the source dataset. In the case of lakes that are sampled in multiple source programs, again the maximum value for *maxdepth* and *meandepth* from each program will be populated at the level of the NHD lake.

Users should consider *lagoslakeid* to be the lake identifier that facilitates the identification of unique lakes in LAGOS_LIMNO_ and LAGOS_GEO_ and should be used in analysis. *Lagoslakeid* is an integer identifier calculated from the *nhdid* (or *Permanent_Identifier*), which is a GUID that has characters that may not import into certain statistical packages readily.
